# Targeting LncRNA *LLNLR-299G3.1* with antisense oligonucleotide inhibits malignancy of esophageal squamous cell carcinoma cells *in vitro* and *in vivo*

**DOI:** 10.32604/or.2023.028791

**Published:** 2023-06-27

**Authors:** LI TIAN, YONGYI HUANG, BAOZHEN ZHANG, YI SONG, LIN YANG, QIANQIAN CHEN, ZHENG WANG, YILING WANG, QIHAN HE, WENHAN YANG, SHUYONG YU, TIANYU LU, ZICHEN LIU, KAIPING GAO, XIUJUN FAN, JIAN SONG, RIHONG ZHAI

**Affiliations:** 1 School of Public Health, International Cancer Center, Guangdong Key Laboratory for Genome Stability & Disease Prevention, Shenzhen University Medical School, Shenzhen, China; 2 Shenzhen Institute of Advanced Technology, Chinese Academy of Science, Shenzhen, China; 3 Department of Thoracic Surgery, Shenzhen People’s Hospital, Shenzhen, China; 4 Department of Gastroenterology, Hainan Tumor Hospital, Haikou, China; 5 Department of Gastroenterology, Southern University of Science and Technology Hospital, Shenzhen, China

**Keywords:** *LLNLR-299G3.1*, Chromatin, Esophageal squamous cell carcinoma (ESCC), Antisense oligonucleotide (ASO), Placental chondroitin sulfate A binding peptide (plCSA-BP)-coated nanoparticles

## Abstract

Accumulating evidence has indicated that long non-coding RNAs (lncRNAs) play critical roles in the development and progression of cancers, including esophageal squamous cell carcinoma (ESCC). However, the mechanisms of lncRNAs in ESCC are still incompletely understood and therapeutic attempts for *in vivo* targeting cancer-associated lncRNA remain a challenge. By RNA-sequencing analysis, we identified that *LLNLR-299G3.1* was a novel ESCC-associated lncRNA. *LLNLR-299G3.1* was up-regulated in ESCC tissues and cells and promoted ESCC cell proliferation and invasion. Silencing of *LLNLR-299G3.1* with ASO (antisense oligonucleotide) resulted in opposite effects. Mechanistically, *LLNLR-299G3.1* bound to cancer-associated RNA binding proteins and regulated the expression of cancer-related genes, including OSM, TNFRSF4, HRH3, and SSTR3. ChIRP-seq (chromatin isolation by RNA purification and sequencing) revealed that these genes contained enriched chromatin binding sites for *LLNLR-299G3.1*. Rescue experiments confirmed that the effects of *LLNLR-299G3.1* on ESCC cell proliferation were dependent on interaction with HRH3 and TNFRSF4. Therapeutically, intravenous delivery of placental chondroitin sulfate A binding peptide-coated nanoparticles containing antisense oligonucleotide (pICSA-BP-ANPs) strongly inhibited ESCC tumor growth and significantly improved animal survival *in vivo*. Overall, our results suggest that *LLNLR-299G3.1* promotes ESCC malignancy through regulating gene-chromatin interactions and targeting ESCC by pICSA-BP-ANPs may be an effective strategy for the treatment of lncRNA-associated ESCC.

## Introduction

Esophageal cancer (EC) is one of the most fatal malignancies with high mortality and morbidity worldwide [1,2]. EC consists of two major pathological subtypes: esophageal adenocarcinoma (EA) and esophageal squamous cell carcinoma (ESCC), with the latter accounting for 90% of all EC cases in the world [3,4]. Although significant progress has been achieved in the diagnosis and treatment for ESCC in the past decades, the five-year survival rate for patients with ESCC remains less than 30% [5,6]. Therefore, it is still necessary to explore the molecular mechanisms underlying ESCC pathogenesis in order to develop more effective therapy for ESCC.

Long noncoding RNAs (lncRNAs) are RNA transcripts with lengths exceeding 200 nucleotides (nt) but without protein coding potential [7]. It has been found that the expression of lncRNAs is strikingly cell- and tissue-type specific [8], indicating that lncRNAs may serve as sensitive biomarkers for different diseases. Importantly, lncRNAs play essential regulatory roles in proliferation, apoptosis, invasion, and differentiation in many cancers including breast, colon, liver, pancreatic, and lung cancers [9]. ESCC-related lncRNAs, such as TTN-AS1, LINC01503, and PCAT1 have also been identified *in vitro* and *in vivo* [10–12]. However, the regulatory mechanisms of lncRNAs in ESCC remains largely unclear. Moreover, while it has been proposed that targeting lncRNAs with specific antisense oligonucleotides (ASOs) may be a promising cancer therapeutics, several limitations, including tissue-specific delivery and the ability to penetrate into cancer cells, remain to be unsolved challenges for the clinical application of ASO-lncRNAs [13,14].

Previously, we have identified a novel lncRNA *LLNLR-299G3.1* (ENSG00000272448.1) that was up-regulated in exosomes from patients with early-stage ESCC (GSE104926) [15]. However, whether and how *LLNLR-299G3.1* may play a role in ESCC is unclear. The primary objective of the current study was to investigate the biological functions and the regulatory mechanisms of *LLNLR-299G3.1* in ESCC. The second aim of this study was to investigate whether a novel type of nanoparticles conjugated with glycosaminoglycan placental chondroitin sulfate A binding peptide (plCSA-BP) was able to deliver ASO-*LLNLR-299G3.1* for targeting inhibition of ESCC cell *in vivo*.

## Materials and Methods

### Patients and specimen

Tumor tissues and adjacent normal tissues were collected from patients with ESCC undergoing surgical resections in the Department of Thoracic Surgery at Shenzhen People’s Hospital, China [15]. No local or systemic treatments were administered to these patients before surgery. All collected tissue samples were immediately snap-frozen in liquid nitrogen and stored at −80°C. This study was approved by the Medical Ethics Committee of Shenzhen University Health Science Center (protocol no. 2016001). A written informed consent was obtained from each subject before participating in this study.

### Cell lines and cell culture

The normal esophageal epithelial cell line (NE3), originally developed at Hong Kong University, was obtained from Professor Li Fu (Department of Pharmacology, Shenzhen University). Other human ESCC cell lines, including Eca-109 (RRID: CVCL_6898), KYSE-30 (RRID: CVCL_1351), KYSE-70 (RRID: CVCL_1356), KYSE-180 (RRID: CVCL_1349), KYSE-450 (RRID: CVCL_1353), and TE1 (RRID: CVCL_1759) were purchased from the Cobioer Biosciences Co., Nanjing, China. NE3, Eca-109, KYSE30, KYSE70, KYSE180, KYSE450, and TE1 cells were maintained in 10% FCS-RPMI-1640 medium (Invitrogen, Shanghai, China). NE3 cells were cultured in EpiCM 4101 medium (ScienCell, USA). The cells were cultured in humidified atmosphere of 5% CO_2_ at 37°C. All cell lines were authenticated by matching the short-tandem repeat (STR) DNA profiles of the cell to the corresponding standard STR in the database of ATCC (the American Type Culture Collection) and DSMZ (Deutsche Sammlung von Mikroorganismen und Zellkulturen). All cells were routinely tested and found negative for mycoplasma.

### Construction of LLNLR-299G3.1-overexpression lentivirus vector

To generate lentiviruses for stably overexpression of *LLNLR-299G3.1*, full length cDNA of *LLNLR-299G3.1* was cloned into the pHBLV-CMV-MCS-3flag-EF1-ZsGreen-T2A-PURO vector system (Hanbio Biotech., Shanghai China). The vectors were verified by sequencing and electrophoresis after digesting by EcoRI and BamHI (Thermo Fisher, Shanghai, China). The lentiviral vectors were co-transfected into 293T cells with packaging plasmid pSPAX2 and envelope plasmid pMD2G using the Lipofiter^™^ transfection reagent (Hanbio Biotech., Shanghai, China). After 48 h of infection, lentiviral particles in the supernatant of cell culture were collected and concentrated by ultracentrifugation at 4°C. Concentrated viruses were used to infect 5 × 10^5^ cells in a 6-well plate, and then subjected to selection with 1.5 µg/ml puromycin in 10% FBS for 1 week. Stable overexpression cell lines or NC cell lines were identified by qRT-PCR assay.

### Antisense oligonucleotide (ASO) and cell transfection

Antisense oligonucleotide (ASO) against *LLNLR-299G3.1* was synthesized by RiboBio (Guangzhou, China). To determine the *in vitro* interference efficiency, ASO was transfected into TE1 cells using the Lipofectamine 3000 kit (Life Technologies, Shanghai, China) and the expression level of *LLNLR-299G3.1* was analyzed by qRT-PCR assay. For the *in vitro* ASO functional assays, cells were seeded in 96-well plates and transfected with 100 nM of the ASO using the Lipofectamine 3000 kit following the manufacturer’s instruction. At 48 h post transfection, cells were harvested for further analyses.

### 5′ and 3′ rapid amplification of cDNA ends (RACE) analysis

Total RNA was isolated from TE1 cells using Trizol reagent kit (Invitrogen, Shanghai, China), according to the manufacturer’s instructions. 3′RACE was performed using the 3′-Full RACE Core Set with PrimeScript Rtase (Code No.6106, Takara, Dalian, China), and 5’RACE was conducted with the SMARTer® RACE 5′/3′ Kit (Cat. No. 634860, Takara, Dalian, China), following the manufacturer’s protocols. All *cDNA* products were amplified by the TaKaRa Tks Gflex DNA Polymerase (Code No. R060A, Takara, Dalian, China). The RACE PCR products were separated on a 1.5% agarose gel using the TaKaRa MiniBEST Agarose Gel DNA Extraction Kit Ver.4.0 (Code No. 9762) and verified by RNA sequencing on ABI Prism 3730XL DNA Analyzer (Life Technologies, CA, USA). The gene-specific primers (5′RACE, 3′RACE) used for the PCR of the RACE analysis are listed in Suppl. Table S1.

### RNA extraction and qRT-PCR

Total RNA was extracted from either cells or tissues using the Trizol reagents (Invitrogen, CA, USA) according to the user’s guide. 1 µg of total RNA was reversely transcribed into cDNA in a final volume of 20 µl using the PrimeScript RT reagent kit (Takara, Tokyo, Japan). Real-time PCR was performed using the SYBR Premix RT-PCR kit (Takara, Tokyo, Japan) with quantitative fluorescence gene amplification instrument(qTOWER^3^ analytik jena, German) under the following conditions: 95°C for 5 min followed by 40 cycles at 95°C for 10 s, 60°C for 30 s, 95°C for 15 s, and then 60°C for 1 min. The results were normalized to the expression level of GAPDH (internal control) and the fold change of gene expression was calculated using the 2^−ΔΔCt^ method [16]. All primers used in this study were synthesized by RiboBio (Guangzhou, China) and are listed in Suppl. Table S1.

### Cell proliferation assay

Cell viability was evaluated using the cell counting kit-8 (CCK-8) (DojinDo, Tokyo, Japan) following the manufacturer’s guidelines. A total of 5000 cells per well were seeded in 96-well culture plates and incubated overnight at 37°C with 5% CO_2_. Subsequently, 10 µl CCK8 reagent was added into each well and incubated for 2h. Lastly, cell viability was measured every 24 h (0, 24, 48, 72, and 96 h) at the absorbance of 450 nm using the Synergy HTX multi-mode reader (BioTek, Germany).

### Transwell assay

For transwell assay, 1 × 10^5^ cells in fetal bovine serum (FBS)–free medium were seeded to the upper chamber of the Transwell insert coated with Matrigel (BD Biosciences, CA, USA). RPMI-1640 medium containing 10% FBS was added into the lower chamber. After incubation for 24 h, the cells remaining on the upper chamber were removed with cotton wool. Cells that had migrated or invaded through the membrane were fixed by cold methanol and stained by 0.1% crystal violet. The stained cells were counted under a microscope in six randomly selected areas and the number of migrated cells was presented relative to untreated controls. All experiments were performed three times in triplicate.

### Wound healing assay

The motility of cells was measured by wound healing assays. Cells were seeded in 6-well plates and cultured to 90–100% confluent for 48 h. Wounds were made in each well using pipette tips. The cell migration distance was observed at 0 and 48 h under a microscope. Then the relative percentage of the wound closure was calculated. The areas between scratches were measured using Image Pro Plus 6.0 software under the microscope (Nikon, ECLIPSE NI-U, Tokyo, Japan) and the relative percentage of the wound closure was calculated according to the formula (area of 0 h–area of 24 h)/area of 0 h.

### Apoptosis assay

Apoptosis of cells was analyzed using the Annexin V-FITC cell apoptosis kits (BD Biosciences, CA, USA) according to the manufacturer’s instruction. Briefly, ESCC cells were seeded into 6-well plates and cultured for 24 h. Cells were then stained with Annexin V-fluorescein isothiocyanate (FITC) and propidium iodide (PI). The apoptotic rates of cells were determined by flow cytometer (Beckman Coulter, Inc., IN, USA) equipped with CytExpert software (version 2.3).

### RNA pull-down and mass spectrometry assays

RNA pull-down analysis was carried out using the Pierce^™^ Magnetic RNA-Protein Pull-Down Kit (Thermo Fisher, Shanghai, China). Briefly, cells were firstly lysed in dilution buffer and RNA was extracted by Trizol reagent (Invitrogen, Shanghai, China). Then large Scale RNA Production System-T7 Kit (Promega, Beijing, China) was used to synthesize large quantities of RNA and RNA 3′ End Desthiobiotinylation Kit (Thermo Fisher, Shanghai, China) was applied to label the RNA with biotin. Thereafter, biotin-labeled RNAs were mixed with cell extracts and incubated with Pierce Nucleic-Acid Compatible Streptavidin Magnetic Beads (Thermo Fisher, Shanghai, China). The RNA-protein complex was isolated from magnetic beads using Biotin Elution buffer and boiled in SDS buffer. After RNA pull-down, equal amounts of samples pulled down by sense and anti-sense *LLNLR-299G3.1* were loaded on SDS-PAGE (sodium dodecyl sulphate polyacrylamide gel electrophoresis). Then the gel was stained with Fast silver stain kit (Beyotime, Shanghai, China) according to the manufacturer’s instructions. Specific bands were cut and analyzed by Q-Exactive mass spectrometer (Thermo Fisher Scientific, NH, USA). Protein identification was retrieved in the human RefSeq protein database (National Center for Biotechnology Information), using Mascot version 2.4.01 (Matrix Science, London, UK; http://www.matrixscience.com/).

### Subcellular fractionation

The nuclear and cytoplasmic RNA fractions were extracted from TE1 cells using the NE-PER^™^ Nuclear and Cytoplasmic Extraction Reagents (Cat: 78835, Thermo Fisher) according to the manufacturer’s protocols. *LLNLR-299G3.1* expression levels were quantified by qRT-PCR assay, using U1 gene as nuclear control and GAPDH as cytoplasmic control.

### Construction of ASO-loaded plCSA-BP nanoparticles (plCSA-ANPs)

The plCSA-conjugated biodegradable lipidnanoparticles were synthesized using our previously published EDC/NHS bioconjugate techniques [17,18] ASO-*LLNLR-299G3.1* was encapsulated in plCSA-BP-nanoparticles using our prior reported one-step sonication method [19] with minor modifications. Briefly, Dlin-MC3-DMA, DSPC, cholesterol and DSPE-PEG-COOH were solubilized in ethanol at a molar ratio of 50/10/38.5/1.5. The lipid mixture was added to an aqueous buffer (50 mM citrate, pH 4) and mixed to a final ethanol and lipid concentration of 30% (vol/vol) and 6.1 mg/ml, respectively. After mixing, the hydrated lipids were passed through an extrusion device (T&T scientific corporation, Knoxville, TN, USA) with an 80 nm diameter filter, following the vendor’s instructions. The ASO (solubilized in a 50 mM citrate, pH 4 aqueous solution containing 30% ethanol) was added to the nanoparticles (pre-equilibrated to 35°C) using the extrusion device at a rate of 1 ml/min and extruded with an 80 nm diameter filter for 21 times. The final ASO to total nanoparticles ratio were 0.06 (wt/wt). The ethanol was then removed, and the external buffer was replaced with PBS by dialysis.

### Animal experiments

Four-week-old female BALB/c nude mice were purchased from the Beijing Vital River Laboratory Animal Technology Co., Ltd. (Beijing, China) and housed under SPF (Specific Pathogen Free) conditions at the animal care facility of the Experimental Animal Center of Shenzhen Institute of Advanced Technology, Chinese Academy of Science (China).

For ASO *in vivo* treatment experiment, fluconazole (fluc) labeled-TE1 cells stably overexpressed *LLNLR-299G3.1* or the negative control cells (1 × 10^6^ cells/mouse) were subcutaneously inoculated into the right flank area of each nude mice. One week after injection, mice were randomly divided into 4 groups (n = 5/group), treated with PBS (control), the plCSA-NNPs (plCSA-coated lipid nanoparticles loading with ASO-NC, 80 mg/kg), the ANPs (lipid nanoparticles loading with ASO, 80 mg/kg), and the plCSA-ANPs (plCSA-coated lipid nanoparticles loading with ASO, 80 mg/kg), respectively. Treatments were administered via the tail vein injection once a week. The state of the nude mice was observed every day and tumors were measured twice a week, and tumor sizes were calculated using the equation V = length × width 2/2 (V, volume). Tumor growth was monitored by the bioluminescence intensity using the IVIS spectroscopic imaging system (Perkin-Elmer, Waltham, MA) [19]. To investigate tumor metastasis, 21 days after injection, 200 mg/kg D-luciferin potassium salt was injected into the peritoneal cavity of nude mice. The mice were sacrificed by using 30% urethane, and the primary tumor, lung, liver and lymph nodes were dissected for examination of metastasis by fluorescence imaging. All animal procedures were approved and performed in accordance with the Guidance Suggestions for the Care and Use of Laboratory Animals by the Animal Care and Use Committee from the Shenzhen Institute of Advanced Technology, Chinese Academy of Sciences (China).

### RNA-sequencing (RNA-seq)

Total RNA was extracted from ASO-treated TE1 cells using the TRIzol (Invitrogen, Shanghai, China) reagents. RNA quantity and quality were measured using the NanoDrop ND-2000 Bioanalyzer (Agilent, CA, USA). RNA libraries were prepared using the TruSeq Stranded mRNA LT Sample Prep Kit (Illumina CA, USA) with 500 ng of purified total RNA according to the manufacturer’s protocol. The libraries were sequenced on an Illumina HiSeq X Ten Platform at OeBiotech (Shanghai, China) and 150 bp paired-end reads were generated. Raw reads were processed using Trimmomatic software and the clean reads were aligned to the human genome database (GRCh38) using the HISAT2 software (2.0.5). Gene expression level were calculated by the FPKM (fragments per kilobase of transcript per million mapped reads) by HTseq-count softwares. Differential expression was analyzed using the DESeq R package (1.16.1) [15].

### ChIRP-seq

ChIRP-seq (Chromatin isolation by RNA purification sequencing) experiments were performed as previously described with minor modifications [20]. In brief, seveal DNA probes against the *LLNLR-299G3.1* full-length sequence were ordered from Dianxi Biotech (Shanghai, China) and were biotinylated at the 3′ end. TE1 cells were cross-linked, lysed sonicated, incubated with probe sets, and *LLNLR-299G3.1*-bound chromatin was retrieved. The ChIRPed RNA was purified with proteinase K buffer and then subjected for library construction using the NEBNext Ultra II RNA Library Prep kit (New England Biolabs, MA, USA). The purified DNA fragments were sequenced on an Illumina Novaseq PE150 system at Dianxi Biotech (Shanghai, China). Raw reads were mapped to human reference genome assembly (GRCh38) using the STAR (Spliced Transcripts Alignment to a Reference) version 2 software. Peaks were called using the MACS package. Motif enrichment analysis was performed using the online tool Homer (http://homer.ucsd.edu/homer/ngs/peakMotifs.html). The sequences of probes for *LLNLR-299G3.1* are listed in Suppl. Table S1.

### Bioinformatics analyses

All bioinformatics analyses in this study were conducted using the following online softwares and databases. RNA sequencing reads were counted for each gene using HTseq-count (https://pypi.org/project/HTSeq/). Coding potential calculation (CPC) for lncRNA was computed by Coding Potential Calculator at http://cpc2.gao-lab.org/. Gene Ontology (GO) was analyzed in GO knowledgebase (http://geneontology.org/). Molecular pathways of genes or proteins were estimated using the KEGG (Kyoto Encyclopedia of Genes and Genomes) database (https://www.genome.jp/kegg/). Gene Set Enrichment Analysis (GSEA) was performed based on the GSEA database (https://www.gsea-msigdb.org/gsea/index.jsp). Motif enrichment analysis was carried out using the HOMER database (http://homer.ucsd.edu/homer/ngs/peakMotifs.html).

### Statistical methods

Statistical analyses were performed using SAS v. 9.4 program (SAS Corp., NC, USA) or Prism GraphPad 8.0. Normality of data distribution was examined using the Shapiro-Wilk test. Normal distribution continuous variables between subgroups were analyzed using paired or unpaired student’s *t*-test. Wilcoxon rank sum test, and Wilcoxon signed-rank test were used for comparing continuous data that were not in normal distribution. The Mann-Whitney U test was performed for the comparison of the xenograft tumor weights and volumes. All statistical analyses were performed using two-tailed *p*-values. Statistical significance was defined as a *p*-value of less than 0.05.

## Results

### LLNLR-299G3.1 was up-regulated in ESCC

**FIGURE 1:**
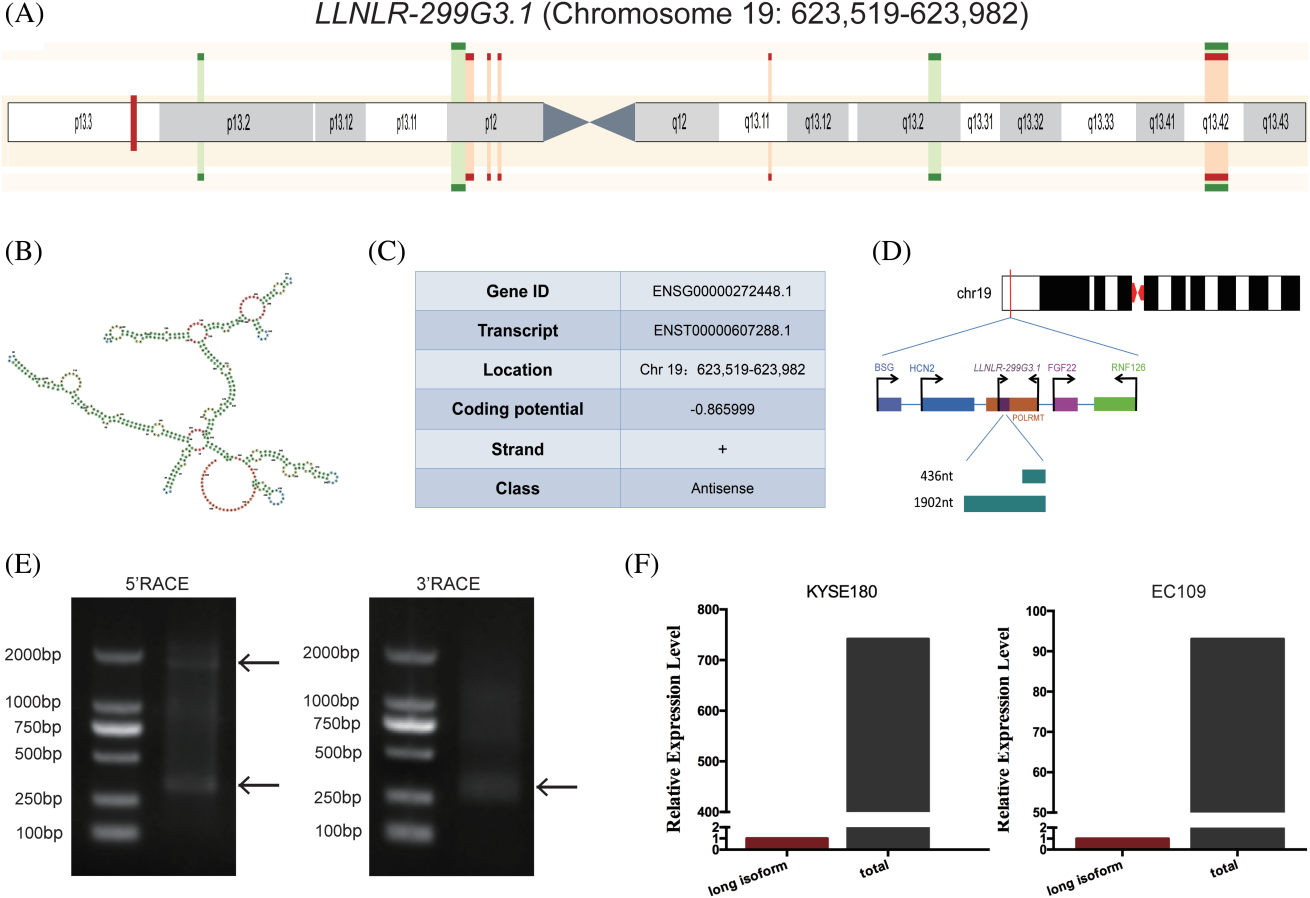
Characteristics of *LLNLR-299G3.1* gene. (A) Schematic view of the chromosomal location of *LLNLR-299G3.1*. (B) The secondary structure of *LLNLR-299G3.1* generated from Ensemble Genome Browser. (C) Genetic characteristics of *LLNLR-299G3.1*. (D) Schematic view of *LLNLR-299G3.1* isoforms. (E) Two isoforms of *LLNLR-299G3.1* were confirmed by 5′ and 3′ RACE-PCR. (F) Relative expression levels of 1902 nt isoform *LLNLR-299G3.1* and the total *LLNLR-299G3.1* in ESCC cells analyzed by RT-qPCR.

By conducting multiple comparison analyses, we have previously found that *LLNLR-299G3.1* was a novel lncRNA which differentially expressed in exosomes between esophagitis and ESCC, and between ESCC and healthy control [15]. The *LLNLR-299G3.1* gene is located in human chromosome 19: 623,519–623,982 (gene ID: ENSG00000272448) (Figs. 1A and 1B). Coding potential calculation (CPC) analysis showed that both transcripts have minimal coding potential (coding potential scores = −0.86599, −0.93685, respectively) (Fig. 1C). Following 5′ and 3′ RACE and PCR analysis, we identified two transcripts (1902-nt and 436-nt, respectively) of *LLNLR-299G3.1* (Figs. 1D and 1E). The expression level of 436-nt isoform is higher than that of 1902-nt isoform in ESCC cells (Fig. 1F), indicating that 436-nt isoform is the major type of *LLNLR-299G3.1* in ESCC cells. Thus, we focused on the 436-nt isoform of *LLNLR-299G3.1* in the subsequent studies.

**FIGURE 2:**
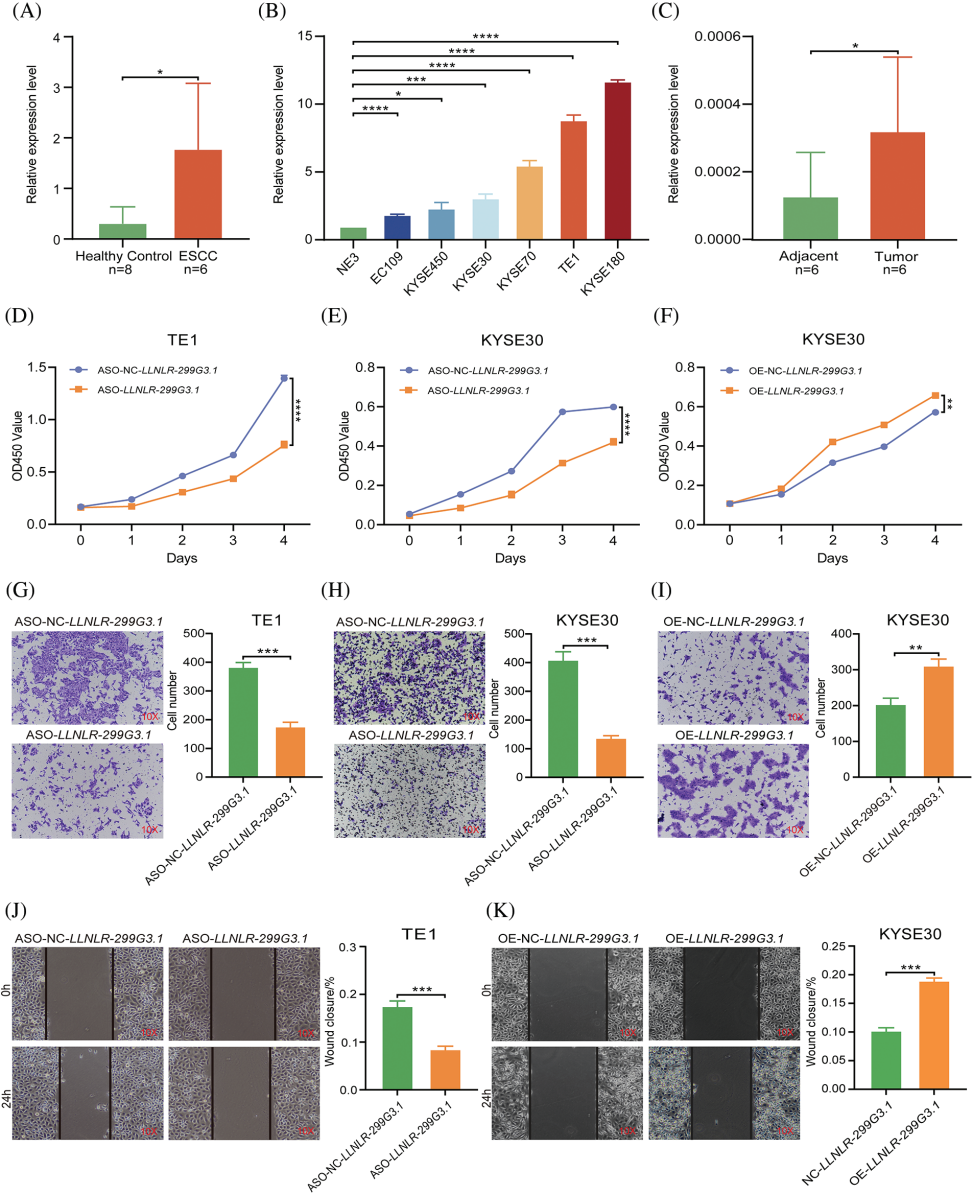
*LLNLR-299G3.1* was up-regulated in ESCC and promoted the malignancy of ESCC cells. (A) Expression levels of *LLNLR-299G3.1* in plasma exosomes from ESCC patients (n = 6) were higher than that from healthy controls (n = 8), detected by qRT-PCR. (B) Expression levels of *LLNLR-299G3.1* in ESCC cell lines (Eca-109, KYSE450, KYSE30, KYSE70, TE1, and KYSE180) compared with that in normal epithelial cell line NE3. Data were presented as expression fold changes relative to NE3 (n = 3, biological replicates). (C) Expression level of *LLNLR-299G3.1* in paired ESCC tumor tissues and adjacent normal tissue, (n = 6). (D) ASO-*LLNLR-299G3.1* suppressed proliferation rate of TE1 cells. (E) Knockdown of *LLNLR-299G3.1* with ASO inhibited cell proliferation of KYSE30 cells. (F) Overexpression of *LLNLR-299G3.1* promoted ESCC cell proliferation. (G) Knockdown of *LLNLR-299G3.1* inhibited migration rates of TE1 cells. (H) Inhibition of *LLNLR-299G3.1* with ASO reduced migration rate of KYSE30 cells. (I) Overexpression of *LLNLR-299G3.1* promoted migration capacity of KYSE30 cells. (J) *LLNLR-299G3.1* knockdown resulted in a shorter vertical migration distance compared with control cells. (K) Overexpression of LLNLR 299G3.1 increased the invasion ability of ESCC cells. Cell experiments were performed in triplicate. *p < 0.05. **p < 0.01. ***p < 0.001. ****p < 0.0001.

To verify the expression levels of *LLNLR-299G3.1* in ESCC, we used qRT-PCR to analyze its expression in exosomes. The results showed that *LLNLR-299G3.1* expression levels in exosomes from ESCC patients were significantly higher than that in healthy controls (Fig. 2A). *LLNLR-299G3.1* expression levels in several ESCC cell lines (KYSE180, KYSE30, KYSE70, and TE1) were also significantly higher than that in normal esophageal epithelial cells (NE3) (Fig. 2B). Moreover, the expression level of *LLNLR-299G3.1* in ESCC tumor tissues was significantly increased, compared to that in adjacent tissues (Fig. 2C). Collectively, these results consistently indicated that *LLNLR-299G3.1* was up-regulated in ESCC.

### LLNLR-299G3.1 promoted proliferation, migration, and invasion of ESCC cells

To assess the biological functions of *LLNLR-299G3.1* in ESCC cells, we performed loss- and gain-of-function experiments in ESCC cell lines. We synthesized *LLNLR-299G3.1* anti-sense oligonucleotides (ASOs) to specifically inhibit the expression of *LLNLR-299G3.1*. It was seen that knockdown of *LLNLR-299G3.1* by ASOs significantly reduced proliferation rates of both TE1 and KYSE30 cells (Figs. 2D and 2E). While stably over-expressed *LLNLR-299G3.1* significantly increased cell proliferation rate of ESCC cells (Fig. 2F). Furthermore, silencing of *LLNLR-299G3.1* suppressed migration capacities of ESCC cells (Figs. 2G and 2H). Conversely, over-expression of *LLNLR-299G3.1* increased migration rates of ESCC cells (Fig. 2I). Similarly, wound healing assay showed that up-regulation of *LLNLR-299G3.1* remarkably promoted ESCC cell invasion capacity and inhibition of *LLNLR-299G3.1* reduced the invasion ability of ESCC cells (Figs. 2J and 2K). The apoptosis rate of ESCC cells were not affected by *LLNLR-299G3.1* over-expression or by knockdown (data not shown). Taken together, these data revealed that *LLNLR-299G3.1* was an oncogenic lncRNA in ESCC cells.

### LLNLR-299G3.1 bound to cancer-associated proteins

**FIGURE 3:**
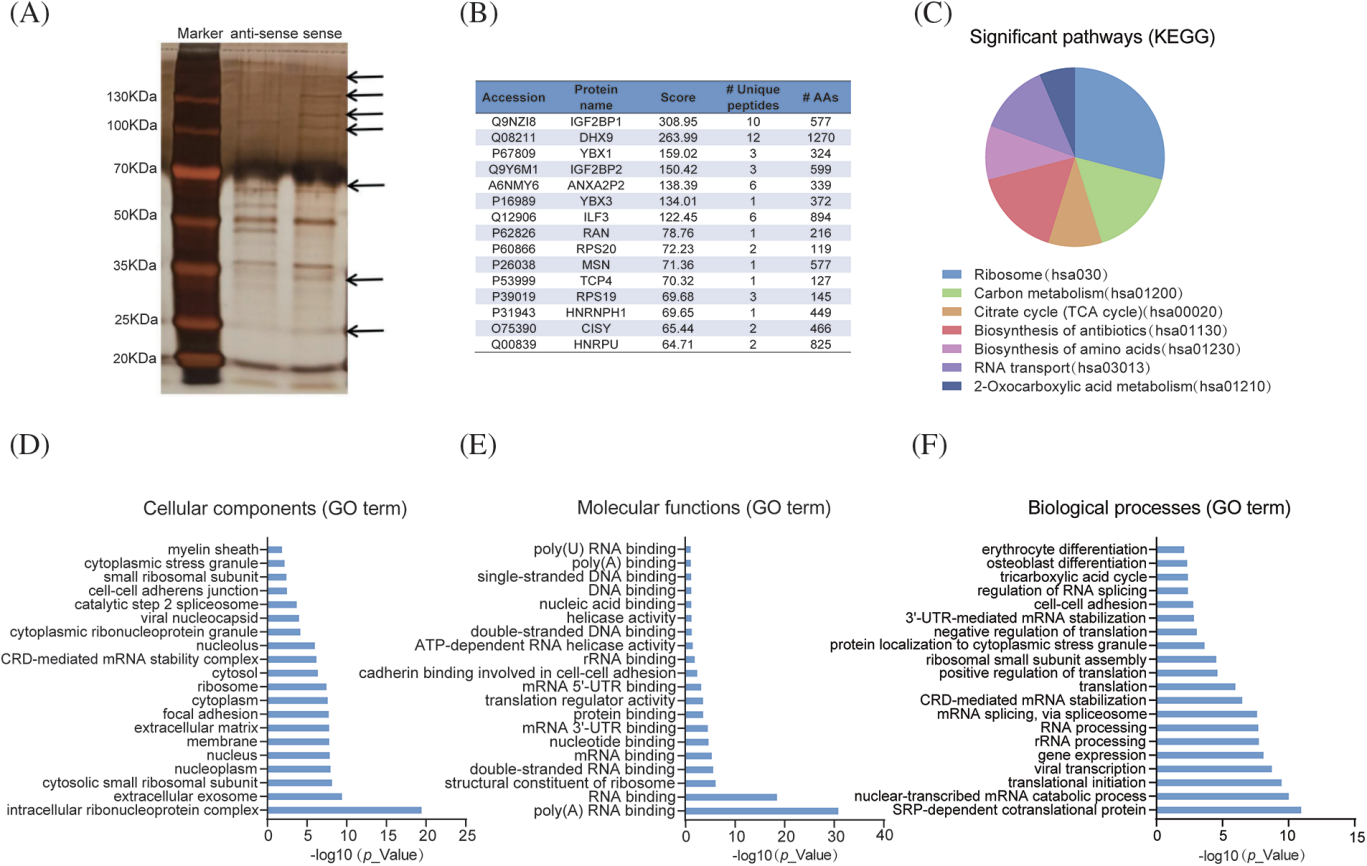
*LLNLR-299G3.1* interacted with cancer-associated proteins. (A) Identification of proteins associated with *LLNLR-299G3.1* using silver staining of RNA pull-down. (B) Top 15 binding proteins identified by mass spectrophotometry. Binding proteins were immunoprecipitated by *LLNLR-299G3.1* and its antisense RNA in TE1 cells. (C) KEGG (Kyoto Encyclopedia of Genes and Genomes) pathway analysis of the *LLNLR-299G3.1*-binding proteins. (D) GO (Gene Ontology) analysis showing cellular components of *LLNLR-299G3.1*-binding proteins. (E) GO analysis indicating the molecular functions of *LLNLR-299G3.1*-binding proteins. (F) GO analysis revealing the biological processes of *LLNLR-299G3.1*-binding proteins.

To explore the mechanisms by which *LLNLR-299G3.1* promotes ESCC cell proliferation and migration, RNA pull-down assays were performed to identify the protein partners binding to *LLNLR-299G3.1*. After silver staining (Fig. 3A), differential bands were subjected to mass spectrometry assays, revealing that *LLNLR-299G3.1* bound to a number of RNA binding proteins (Suppl. Table S2). Among the RNA binding proteins identified, 15 proteins that had the highest Mascot scores have been reported to be involved in the regulation of proliferation and migration in cancer cells, supporting the findings of *in vitro* analyses (Fig. 3B). KEGG Functional pathway analysis revealed that proteins derived from RNA pull-down assay were associated with cancer-related pathways such as ribosome composing (hsa03010), carbon metabolism (hsa01200), and citrate cycle (TCA cycle) (hsa00020) (Fig. 3C). Gene Ontology (GO) analysis showed that these proteins were enriched in cellular components of intracellular ribonucleoprotein complex (GO: 0030529), nucleus (GO: 0005634), and extracellular matrix (GO: 0031012) (Fig. 3D). Molecular functions were mainly related to poly (A) RNA binding (GO: 0044822), double-stranded RNA binding (GO: 0003725), and RNA binding (GO: 0003723) (Fig. 3E), supporting the validity of RNA pull-down experiment. Nevertheless, biological processes were more enriched in mRNA catabolic process (GO: 0000184), translational initiation (GO: 0006413), and gene expression (GO: 0010467) (Fig. 3F).

### LLNLR-299G3.1 regulated the expression of cancer-associated pathway genes

**FIGURE 4:**
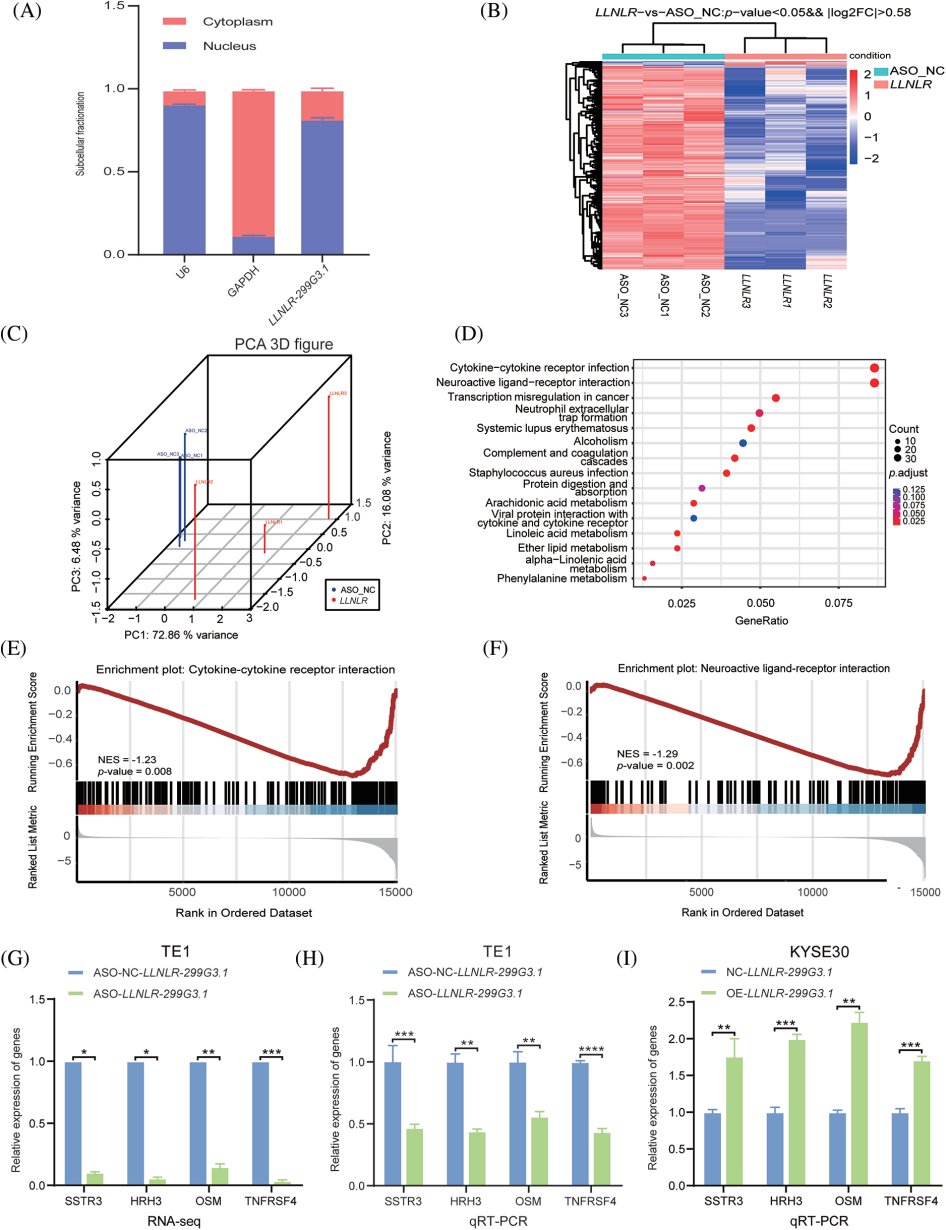
*LLNLR-299G3.1* was involved in the regulation of the cytokine-cytokine receptor interaction and neuroactive ligand-receptor interaction pathways in ESCC cells. (A) *LLNLR-299G3.1* was mainly localized in nucleus. (B) Heat map analysis illustrated genes differentially expressed between ASO-knockdown cells and control cells. (C) Principal component analysis (PCA) showed a clear difference between genetic components of *LLNLR-299G3.1*-knockdown cells and that of control cells. (D) KEGG Pathway analysis suggested that ASO-induced DE-genes were enriched in cancer-associated pathways, particularly cytokine-cytokine receptor interaction and neuroactive ligand-receptor interaction pathways. (E and F) Gene set enrichment analysis (GSEA) showed that inhibition of *LLNLR-299G3.1* with ASO was correlated to downregulation of cytokine-cytokine receptor interaction and neuroactive ligand-receptor interaction pathway genes. (G–I) qRT-PCR assay confirmed the effects of *LLNLR-299G3.1* knockdown or over expression on the expression of cytokine-cytokine receptor interaction pathway genes (TNFRSF4, OSM) and neuroactive ligand-receptor interaction pathway genes (HRH3, SSTR3). Cell experiments were performed in triplicate. *p < 0.05; **p < 0.01; ***p < 0.001; ****p < 0.0001.

To get a better understanding of the regulatory mechanisms of *LLNLR-299G3.1* in gene expression, we first determined the cellular localization of *LLNLR-299G3.1* in the cytoplasm and nucleus. We found that *LLNLR-299G3.1* was mainly expressed in the nucleus (Fig. 4A). Since nuclear lncRNAs are known to be mainly involved in regulating gene expression [21], we conducted RNA-seq to identify differentially expressed genes regulated by *LLNLR-299G3.1* in ESCC cells. After silencing *LLNLR-299G3.1* by ASO, both *LLNLR-299G3.1*-ASO and NC treated cells were subjected to RNA-seq analysis. Using a fold change ≧ 1.5 and *p*-value < 0.05 as cut-off, we found a total of 1039 differentially expressed (DE) genes on *LLNLR-299G3.1* silencing (Suppl. Table S3; Figs. 4B and 4C). Following the identification of DE genes, KEGG pathway analysis was carried out to identify pathways that were perturbed upon *LLNLR-299G3.1* knockdown. There were 38 signaling pathways significantly enriched in differentially expressed genes affected by ASO silencing (Suppl. Table S4; Fig. 4D). Among them, cytokine-cytokine receptor interaction and neuroactive ligand-receptor interaction pathways were the top two most significantly ones, suggesting that these two pathways may play important roles in the regulatory mechanisms of *LLNLR-299G3.1* in ESCC cells. In agreement with this, gene set enrichment analysis (GSEA) showed that there were significant correlations between knockdown of *LLNLR-299G3.1* and expression of these two pathway genes (Figs. 4E and 4F). QRT-PCR analyses on selected genes in these two pathways confirmed the results of RNA-seq (Figs. 4G–4I).

### LLNLR-299G3.1 regulated ESCC-associated genes through directly interacting with chromatins

**FIGURE 5:**
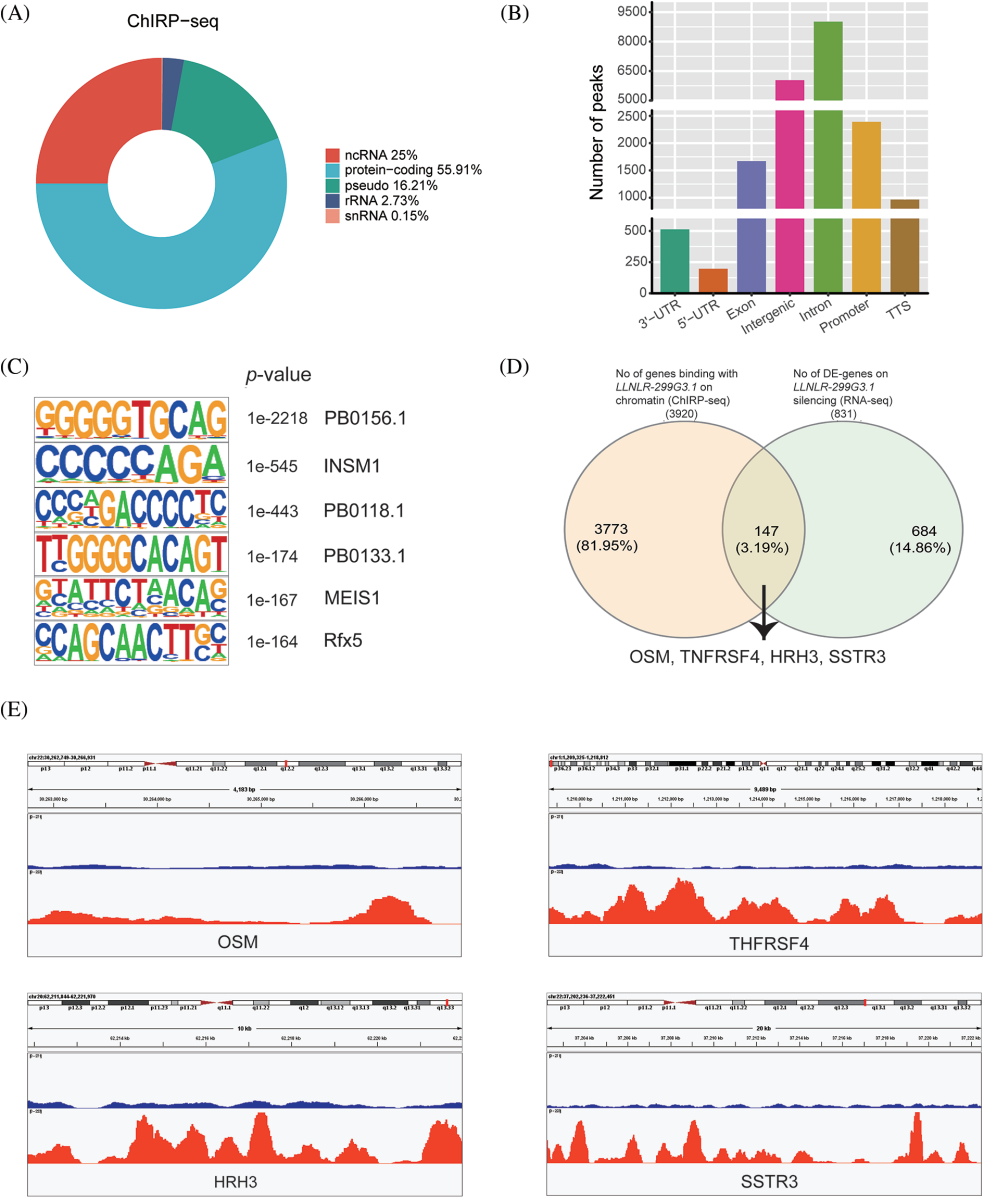
ChIRP-seq (Chromatin isolation by RNA purification sequencing) analysis revealed the enrichment of chromatin targets of *LLNLR-299G3.1*. (A) ChIRP-seq analysis showed the distribution of *LLNLR-299G3.1*-bound peaks in genic region. (B) *LLNLR-299G3.1* chromatin binding sites were enriched in protein-coding genes. (C) The top scoring motifs enriched in *LLNLR-299G3.1*-binding sites. (D) Venn diagram representing overlapped DE-genes (RNA-seq data, fold change ≧ 2.0, *p* < 0.05) with chromatin binding sites for *LLNLR-299G3.1* (ChIRP-seq data). (E) Enrichment of chromatin binding sites for *LLNLR-299G3.1* at the promoter and gene body regions of cytokine-cytokine receptor interaction pathway genes (OSM, TNFRSF4) and neuroactive ligand-receptor interaction pathway genes (HRH3, SSTR3) detected by ChIRP-seq.

Given that nuclear lncRNAs may regulate gene expression through chromatin interactions [22], we further examined whether *LLNLR-299G3.1* may modify target gene expression via chromatin-mediated transcriptional regulation. We performed chromatin isolation by RNA purification (ChIRP) assays in TE1 cells to retrieve chromatins that bound to *LLNLR-299G3.1*. Analyses of the retrieved DNA fragments by deep sequencing (ChIRP-seq) revealed that enriched *LLNLR-299G3.1* chromatin interactions were mainly (55.9%) among protein-coding genes (Fig. 5A). *LLNLR-299G3.1* chromatin interactions were genome-wide, and in genic regions. The most abundant *LLNLR-299G3.1*-chromatin enrichment was located in intronic regions (45.9%), followed by intergenic regions (35.5%) (Fig. 5B; Suppl. Table S5). The enrichment of *LLNLR-299G3.1* chromatin binding regions is highly suggestive of its functional consequences on gene expression.

Next, we performed motif enrichment analysis of these regions and found that the motifs, such as PB0156.1, INSM1. PB0118.1, PB0133.1, MEIS1, and Rfx5, were significantly enriched (Fig. 5C), suggesting that *LLNLR-299G3.1* might affect gene expression by regulating the transcriptional function of these motifs. By overlapping the DE genes (FC ≧ 1.5, *p* < 0.05) detected by RNA-seq with the directly bound genes (peak score ≧ 300) identified by ChIRP-seq, we found 186 overlapped genes between two sets of data (Suppl. Table S6). Among these overlapped genes, we found that several cytokine-cytokine receptor interaction pathway genes (OSM, TNFRSF4) and neuroactive ligand-receptor interaction pathway genes (HRH3, SSTR3) were physically occupied with *LLNLR-299G3.1* and also significantly dysregulated upon *LLNLR-299G3.1* silencing (Fig. 5D). As shown in Fig. 5E, *LLNLR-299G3.1* directly bound to the promoter and gene body regions of these genes, indicating an important function of *LLNLR-299G3.1*-chromatin interaction in gene regulation.

**FIGURE 6:**
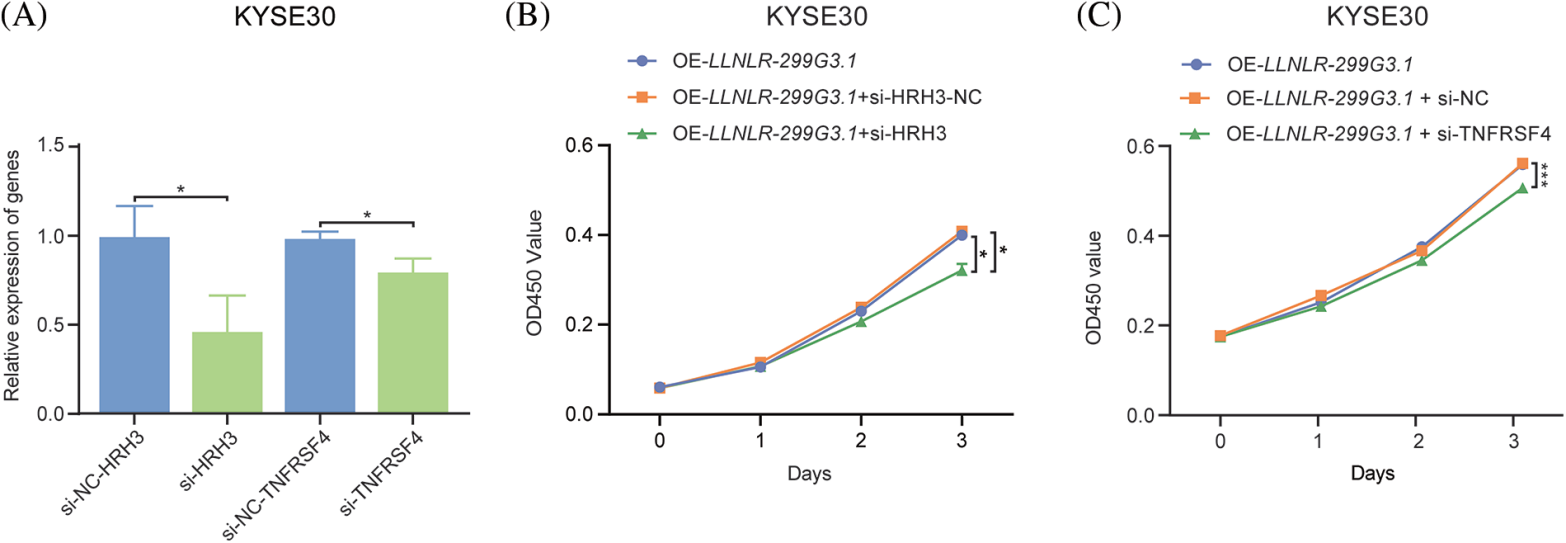
The promoting effect of *LLNLR-299G3.1* on ESCC cell proliferation was dependent on interaction with chromatin enriched genes. (A) Inhibitive effects of si-HRH3 and si-TNFRSF4 on HRH3 and TNFRSF4 gene expression in ESCC cells. (B) The promoting effect of *LLNLR-299G3.1*-overexpression on cell proliferation depended on interacting with si-HRH3. (C) Down-regulation of TNFRSF4 suppressed the promoting ability of *LLNLR-299G3.1* on cell proliferation of ESCC cells. Cell experiments were performed in triplicate. *p < 0.05; ***p < 0.001.

Since DE-genes regulated by *LLNLR-299G3.1* were enriched in cytokine-cytokine receptor interaction and neuroactive ligand-receptor interaction pathways, and ChIRP-seq analysis revealed that genes in these pathways had enriched chromatin binding sites for *LLNLR-299G3.1*, we speculated that *LLNLR-299G3.1* could regulate the malignant phenotypes of ESCC cells by inducing gene-chromatin interactions. To test this hypothesis, we conducted rescue experiments by treating *LLNLR-299G3.1*-overexpression ESCC cells with si-RNAs for cytokine-cytokine receptor interaction pathway gene (TNFRSF4) and neuroactive ligand-receptor interaction pathway gene (HRH3), respectively. As shown in Fig. 6A, si-TNFRSF4 and si-HRH3 significantly suppressed the expression of TNFRSF4 and HRH3, respectively. And over-expression of *LLNLR-299G3.1* increased proliferation of ESCC cells. However, the impacts of *LLNLR-299G3.1* on cell proliferation were abolished by co-transfection with si-TNFRSF4 and si-HRH3, respectively (Figs. 6B and 6C). These findings suggest that *LLNLR-299G3.1* may contribute to ESCC cell proliferation, at least in part, through interacting with cytokine-cytokine receptor interaction and neuroactive ligand-receptor interaction pathway genes. Since these genes contained enriched chromatin binding sites for *LLNLR-299G3.1*, our results also indicate that the effect of *LLNLR-299G3.1* on cell proliferation of ESCC cells were dependent on chromatin-gene interactions.

### Characterization and cellular uptake of the pICSA-ANPs

**FIGURE 7:**
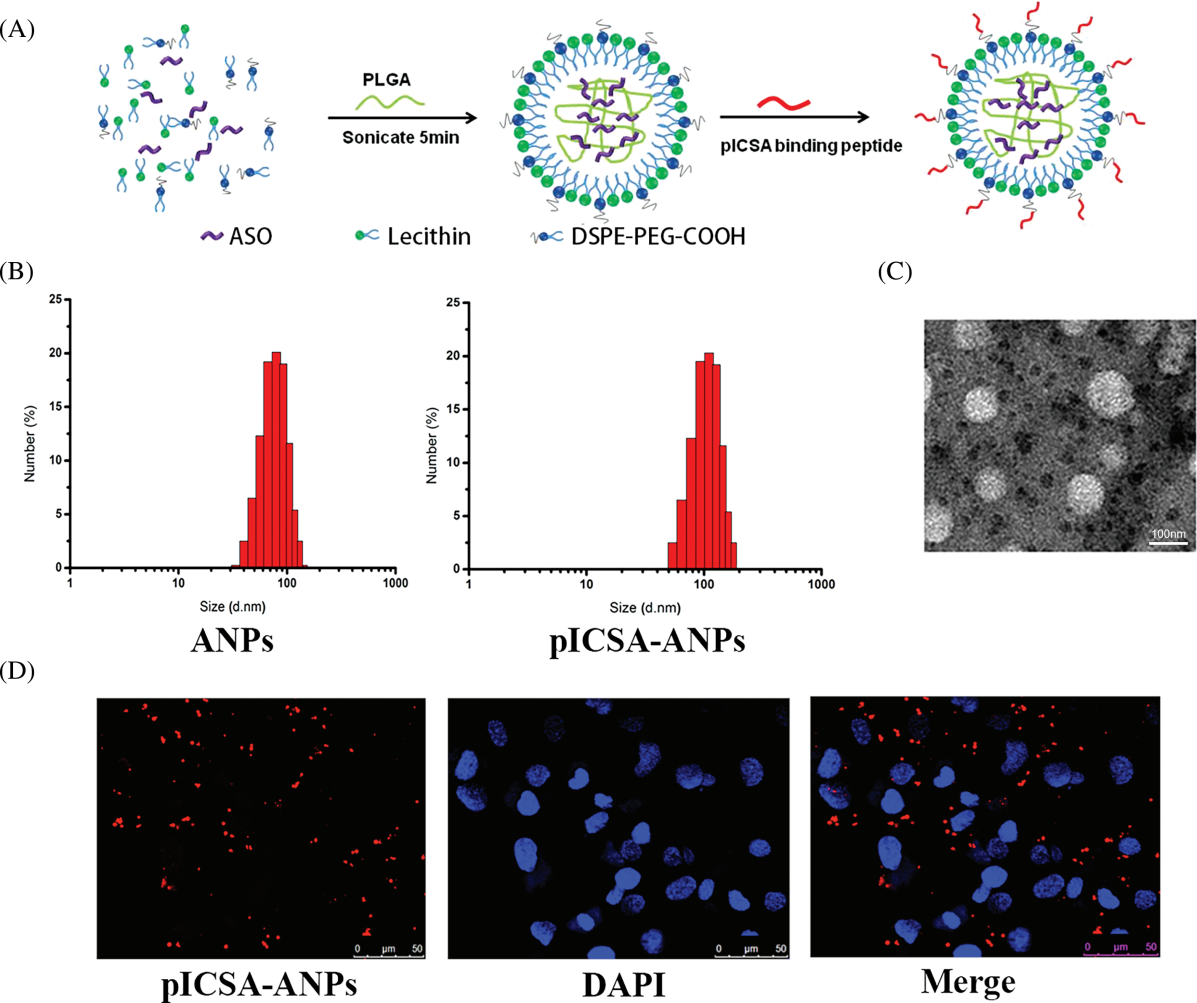
Synthesis and characterization of pICSA-binding protein nanoparticles (plCSA-NP). (A) Schematic illustration of the one-step sonication method to synthesize plCSA-NPs. (B) Size distribution of different nanoparticles. ANPs (ASO-loaded lipid nanoparticles); plCSA-ANPs (ASO-loaded nanoparticles conjugated with plCSA-binding peptide). (C) TEM (Transmission Electron Microscope) image of the plCSA-ANPs. (D) Subcellular localization of the plCSA-ANPs in TE1 cells after a 30min incubation with the plCSA-ANPs. Nuclei were stained with DAPI, and plCSA-ANPs were labeled with Cy5.5.

The pICSA-ANPs were assembled from PLGA, lecithin, DSPE-PEG-COOH, and ASO through a single-step sonication method (Fig. 7A). The mean diameters of the ANPs and pICSA-ANPs were 88.5 ± 5.6 and 113.4 ± 4.7, respectively, suggesting that nanoparticles peptide conjugation increased the size of the nanoparticles (Fig. 7B). TEM images of nanoparticles demonstrated that the particles had generally spherical morphologies (Fig. 7C). To evaluate the cellular uptake of nanoparticles, Cy5.5-labeled pICSA-ANPs were incubated with TE1 cells at 37°C for 30 min, and then the unbound nanoparticles were removed. Subcellular localization of pICSA-ANPs in the cells were observed using confocal laser scanning microscopy (TCS, SP5, Leica, Hamburg, Germany). Red fluorescence signals from pICSA-ANPs were detected in the cytoplasm of TE1 cells, suggesting that pICSA-ANPs were able to enter into TE1 cells (Fig. 7D).

### Intravenous delivery of pICSA-ANPs inhibited ESCC tumor growth in vivo

**FIGURE 8:**
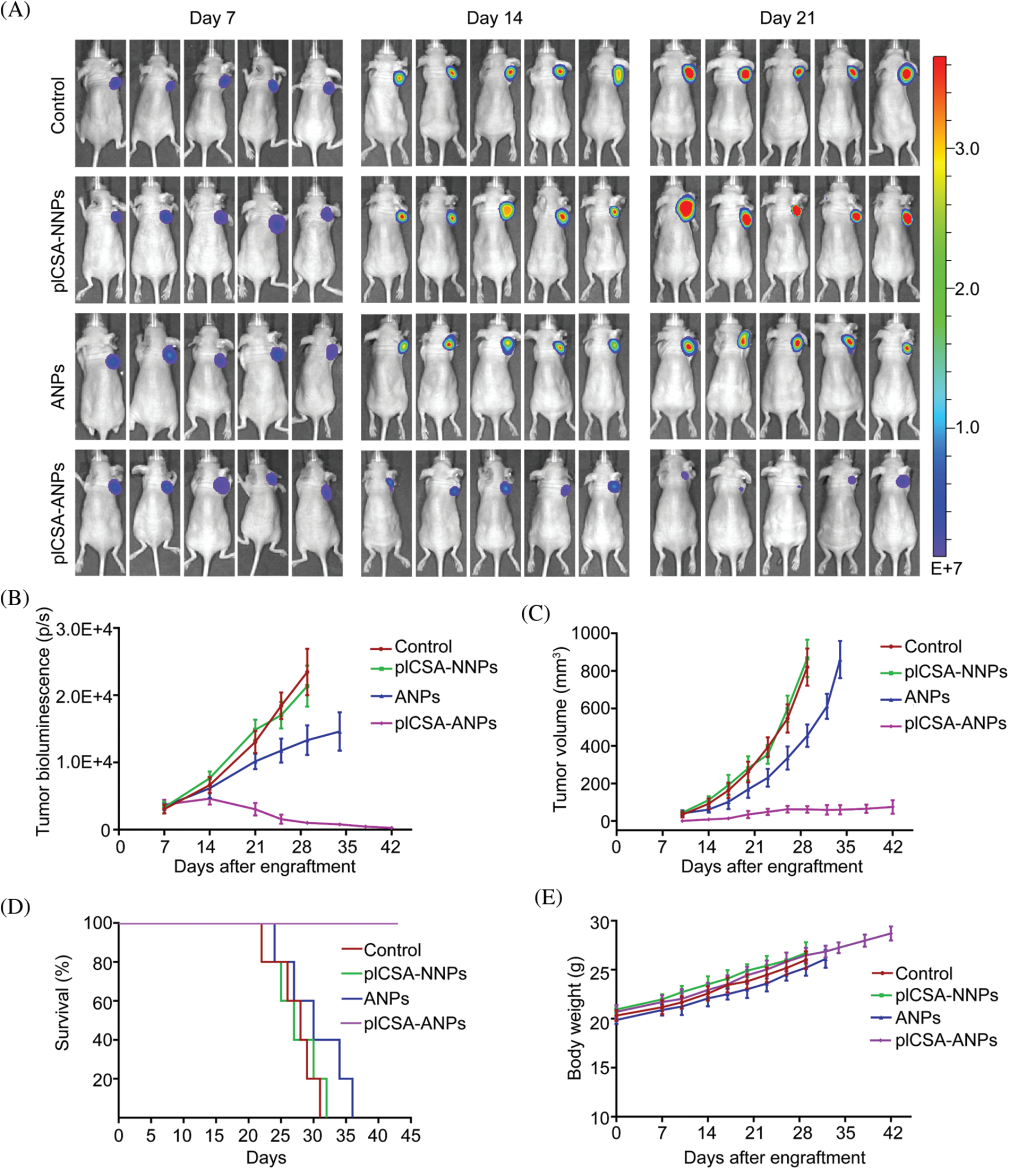
plCSA-ANPs suppressed tumor growth in mice after systemic administration. (A) IVIS analysis of Fluc-GFP-TE1 cell growth in mice receiving different treatments at the indicated time points. (B) The bioluminescence intensity of treatment groups, including pICSA-NNPs, pICSA-ANPs, ANPs and control (PBS). (C) Caliper measurements of the xenograft tumor volume in mice. (D) Kaplan–Meier’s survival curve comparison of tumor-bearing mice treated with pICSA-NNPs, ANPs, pICSA-ANPs and PBS. (E) Body weight changes of mice receiving different treatments

To assess the therapeutic effect of pICSA-ANPs on ESCC cell growth *in vivo*, fluc-TE1 cells stably overexpressed *LLNLR-299G3.1* were grafted into nude mice. The tumor burden was monitored using bioluminescence imaging and caliper measurement. As can be seen in Fig. 8A, the bioluminescence intensities of both pICSA-ANPs and ANPs groups were significantly lower than that of pICSA-NNPs and PBS groups, indicating inhibitive effect of ASOs on tumor growth. Interestingly, the bioluminescence signals from pICSA-ANPs treatment group were evidently weaker than that of ANPs treatment groups, indicating that pICSA-ANPs displayed stronger inhibitive effect on tumor growth than that of ANPs (Fig. 8B). Similarly, tumor volume of pICSA-ANPs treated group was significantly lower than that of ANPs group (Fig. 8C). Furthermore, survival analysis confirmed that pICSA-ANPs treatment resulted in longer survival probability than that of ANPs administration (Fig. 8D). No significant differences in body weight were found among the four nude mice groups during the entire period of animal experiments, suggesting the low toxicity of pICSA-ANPs, ANPs, pICSA-NNPs, and PBS (Fig. 8E). Together, these data demonstrated the effectiveness and safety of systemic administration of pICSA-ANPs for treatment of ESCC tumor growth in mice.

## Discussion

Through genome-wide profiling of lncRNA landscapes, we have previously identified a novel lncRNA *LLNLR-299G3.1* that was significantly enriched in exosomes from early-stage ESCC patients [15]. In the present study, we further verified that *LLNLR-299G3.1* was up-regulated in ESCC tumor tissues and in ESCC cells. We showed that higher expression of *LLNLR-299G3.1* enhanced the malignancy of ESCC cells. Whereas knockdown of *LLNLR-299G3.1* resulted in contrary effects. Our findings suggested that *LLNLR-299G3.1* was an oncogenic lncRNA in ESCC. Moreover, we showed that intravenous delivery of ASO-*LLNLR-299G3.1* by plCSA-BP-ANPs significantly suppressed ESCC tumor growth with high efficiency and low toxicity, highlighting the potential of plCSA-BP-ANPs for ASO-based therapies in ESCC.

Antisense oligonucleotides (ASOs) are synthetic single-strand DNAs that can specifically bind to target mRNA through Watson–Crick hybridization [23]. This DNA-RNA hybrid may recruit and activate the endonuclease RNase-H to cleave the RNA strand, degrade the mRNA, and prohibit protein translation [24,25]. Due to its ability to specifically inhibit transcription and translation of oncogenes, ASOs have been used for experimental therapy in some types of cancers [26]. However, several limitations, including low stability, weak affinity with cell membrane, and poor targeting restrict the translation of ASOs for clinical application [14]. Recent progressions in nanotechnology provide new opportunity for safer and more efficient delivery of anticancer agents to target cells or tissues [27]. However, results from *in vivo* treatment did not result in the desired therapeutic effects [28], partly due to low tissue specificity and varied tumor microenvironments [29]. It has been proposed that conjugate-mediated delivery might improve nanoparticle pharmacokinetics and efficacy in tissues [30]. For example, we previously developed a novel type of nanoparticles coated with glycosaminoglycan placental chondroitin sulfate A binding peptide (plCSA-BP), which could specifically bind to many types of cancer cells [17]. We demonstrated that plCSA-BP-conjugated nanoparticles (plCSA-NP) had higher specificity and efficiency than conventional nanoparticles for delivering chemotherapeutic drugs to cancer cells and tumor tissues [17,18]. However, whether plCSA-NP could also effectively deliver lncRNA-targeting ASOs to cancer tissues has not been characterized.

It has been suggested that lncRNA-targeting ASOs could regulate the biological functions of lncRNA in cancers. For instance, BCAR4-targeted ASOs were found to be able to effectively inhibit metastasis in breast cancer mouse models [31]. Intravenous delivery of ASO-LINC00673 by liposomes significantly suppressed breast cancer cell proliferation *in vivo* [32]. However, the actual translation of ASOs into clinical applications has been hampered by several difficulties, such as the need to prevent ASO from degradation by nucleases in biological fluids, the transportation of ASO to target tissues, and the intracellular delivery of ASO to the target cell organs [33]. To overcome these limitations, several RNAi delivery approaches have been developed. Among them, lipid-based nanoparticles showed more promising potential due to their low toxicity and high biocompatibility compared with inorganic nanoparticles or viral systems [34]. Nevertheless, the instability of lipid nanoparticles in the biological environment, and the low specificity to tumor cells, have restrained their application *in vivo* [35,36]. plCSA is a fragment of protein VAR2CSA that could specifically bind to 90% of known cancer cells, and drug-conjugated VAR2CSA could inhibit tumor growth in mouse models *in vivo* [37]. PlCSA-BP, similar to VAR2CSA, was able to bind to multiple human cancer cells that expressed plCSA on the cell surface [18]. Previously, we and others have demonstrated that plCSA-BP was a specific guiding peptide for delivery of nanoparticle-loaded chemotherapeutic drugs to target tumor xenografts [17–19]. However, no attempts have been made regarding the use of plCSA-BP to guide the ASO-loaded nanoparticles to tumors. In this study, we showed that ASOs-loaded lipid nanoparticles (ANPs) were able to deliver ASO-*LLNLR-299G3.1* to tumor xenografts and inhibited the growth of xenograft tumors. Moreover, we demonstrated that conjugation of plCSA-BP to ANPs significantly increased the inhibitive effects of ANPs on the tumor growth. To the best of our knowledge, this is the first report using plCSA-BP-nanoparticles to deliver lncRNAs-ASO to targeting tumors. Our results highlight the usefulness of plCSA-BP-nanoparticles as a novel delivery tool for ASO-targeted therapeutics in cancers.

Increasing evidence have suggested that RNA binding proteins (RBP) may play a role in modulating cancer initiation and progression through binding to RNAs and regulating their processing, stability, localization, modification or translation [38,39]. Our RNA pull-down analysis revealed that *LLNLR-299G3.1* could bind to several proteins, including IGF2BP1, DHX9, YBOX1, and ANXA2P2. Consistent with our hypotheses, these lncRNA binding proteins have been associated with cell proliferation and migration in multiple cancer cells. For instance, IGF2BP1 could post-transcriptionally regulate the expression of some essential genes required for the control of tumor cell proliferation, invasion, and chemo-resistance, and was associated with a poor overall survival and metastasis in various types of human cancers [40]. DHX9 has been implicated in many cellular processes, including regulation of DNA replication, transcription, translation, RNA processing and transport, tumor cell maintenance and drug response [41]. YBX1 promoted tumorigenesis and metastasis via interacting with c-Myc and linc02042 *in vitro* and *in vivo* in ESCC [42]. ANXA2P2 was up-regulated in hepatocellular carcinoma (HCC) tumor tissues and in HCC cells. Higher expression of ANXA2P2 was associated with worse prognosis of HCC patients [43]. ILF3 was overexpressed in gastric cancer and contributed to cell proliferation and poorer prognosis in patients with gastric cancer [44]. Our findings, together with results from previous studies by others, suggested an important mechanism by which *LLNLR-299G3.1* promoted ESCC cell proliferation and migration via its RNA-binding activities.

Our RNA sequencing analysis showed that knockdown of *LLNLR-299G3.1* led to the dysregulation of multiple genes in ESCC cells. KEGG pathway analysis revealed that differentially expressed genes regulated by *LLNLR-299G3.1* were significantly enriched in the cytokine-cytokine receptor interaction and neuroactive ligand-receptor interaction, pathways. The cytokine-cytokine receptor interaction pathway was involved in the initiation and progression of many types of cancers [45,46], including ESCC [47]. The neuroactive ligand-receptor interaction pathway has been associated with tumorigenesis and poor prognosis in cancers [48–50]. It has been found that lncRNAs contributed to cancer prognosis through a competing endogenous RNA (ceRNA) regulatory network with the “neuroactive ligand-receptor interaction” pathways [51].

It has been suggested that lncRNAs may regulate tumor progression through interacting with chromatin [52]. For example, HOTTIP promoted leukemogenesis by altering the chromatin signature at the FAS promoter and increasing its expression [53]. The oncogenic LINC00284 contains enriched chromatin binding sites for genes it regulated in breast cancer [54]. Meg3 was a cancer suppressor lncRNA which bound to chromatin on unique genomic regions in the c-Met gene in pancreatic neuroendocrine tumors (PNETs) [55]. Nevertheless, whether lncRNA and chromatin interaction may play a role in ESCC remains to be elucidated. Here, we showed that *LLNLR-299G3.1* was predominantly nuclear and had genomic interactions. Among genic regions, the *LLNLR-299G3.1*-chromatin interactions were most common in regulatory regions such as intronic and intergenic regions. It was noteworthy that although a large number of genome loci were found to be associated with *LLNLR-299G3.1* in ChIRP-seq analysis, only a small number of intersected genes were overlapped with corresponding DE-genes identified by RNA-seq. It could be attributed to the fact that binding of lncRNA to chromatin is a physical phenomenon while regulation of genes by lncRNA is a mechanical phenomenon [56]. These observations highlighted that a lncRNA might not regulate all the physically associated genomic region in every cell, but highly cell specific and context dependent. In RNA-seq analysis, *LLNLR-299G3.1*-dysregulated genes were significantly enriched in cytokine-cytokine receptor interaction and the neuroactive ligand-receptor interaction pathway genes, including OSM, TNFRSF4, HRH3, and SSTR3. The enrichment of chromatin interactions in these genes identified by further ChIRP-seq suggested that binding of *LLNLR-299G3.1* on chromatin had functional consequence. Indeed, in subsequent rescue experiments, it was confirmed that the impacts of *LLNLR-299G3.1* on ESCC cell proliferation were dependent on interacting with these genes. Collectively, our data suggest that *LLNLR-299G3.1* is a chromatin regulator of ESCC-associated genes. Future experiments will be required to determine if *LLNLR-299G3.1*-chromatin interaction may alter chromatin structure and the expression of *LLNLR-299G3.1* gene in ESCC cells.

## Conclusion

**FIGURE 9:**
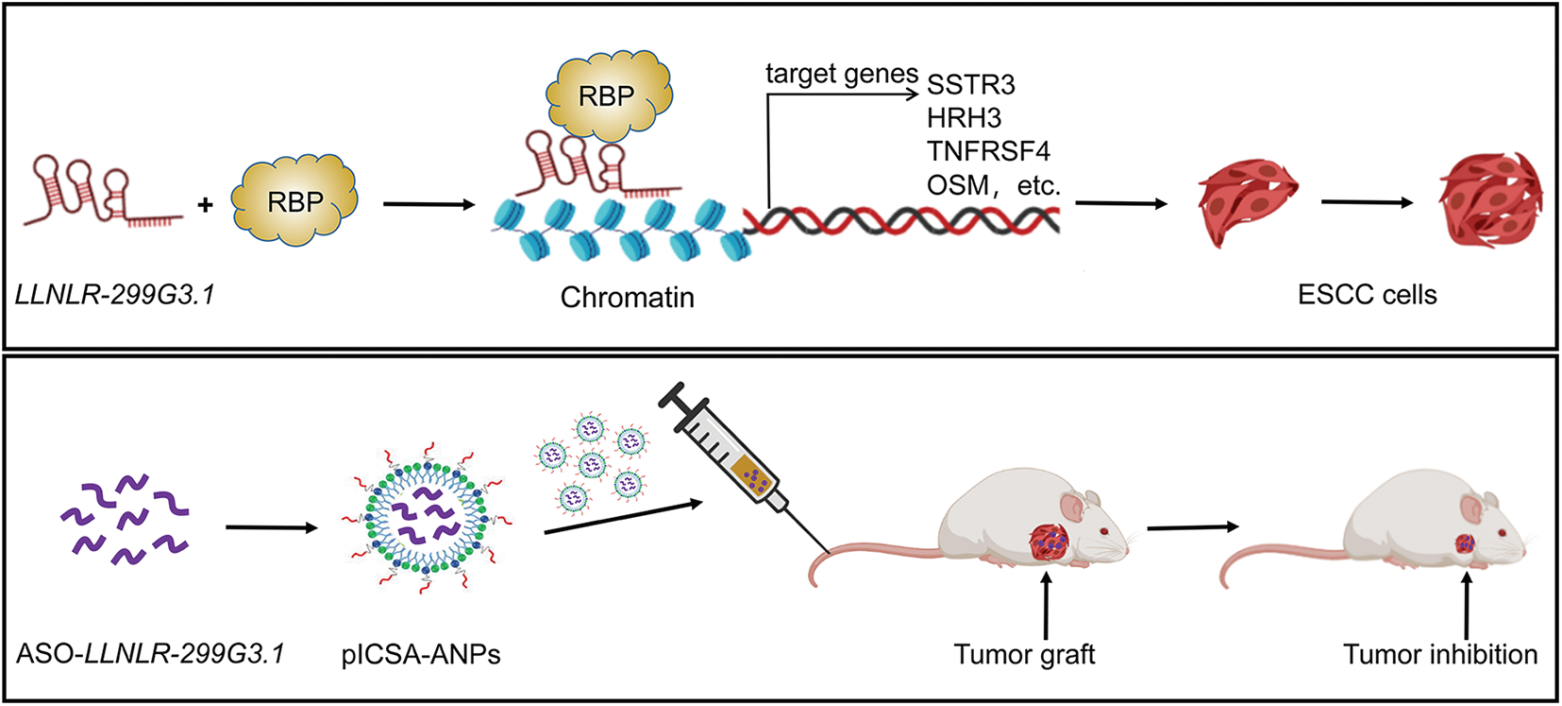
A schematic model depicting the mechanisms of *LLNLR-299G3.1* and the therapeutic effect of pICSA-BP-ANPs on ESCC progression.

In summary, we showed for the first time that *LLNLR-299G3.1* was an oncogenic lncRNA in ESCC. Moreover, we found that *LLNLR-299G3.1* may enhance ESCC progression through genome-wide interactions with chromatins among the genes it regulated. Finally, we demonstrated that plCSA-BP nanoparticle was an efficient tool for specific delivery of ASO-lncRNA to ESCC tumor tissues and may be a novel approach for targeted cancer therapy (Fig. 9).

Supplementary Materials

SUPPLEMENTARY TABLE S1 Primers, oligos, and probes used in this study

SUPPLEMENTARY TABLE S2 *LLNLR-299G3.1* binding proteins detected by RNA pull-down and MS

SUPPLEMENTARY TABLE S3 DE-genes regulated by ASO-*LLNLR-299G3.1*

SUPPLEMENTARY TABLE S4 Results of KEGG analysis on DE-genes

SUPPLEMENTARY TABLE S5 *LLNLR-299G3.1* directly bound targets obtained by ChIRP-seq

SUPPLEMENTARY TABLE S6 Overlapped genes obtained from intersection of RNA-seq and ChIRP-seq dataset

## Data Availability

All relevant data are available from the corresponding author upon request.

## References

[1] Sung, H., Ferlay, J., Siegel, R. L., Laversanne, M., Soerjomataram, I. et al. (2021). Global cancer statistics 2020: GLOBOCAN estimates of incidence and mortality worldwide for 36 cancers in 185 countries. CA: A Cancer Journal for Clinicians*,* 71 *(*3*),* 209–249. 10.3322/caac.21660; 33538338

[2] Abnet, C. C., Arnold, M., Wei, W. Q. (2018). Epidemiology of esophageal squamous cell carcinoma. Gastroenterology*,* 154 *(*2*),* 360–373. 10.1053/j.gastro.2017.08.023; 28823862 PMC5836473

[3] Smyth, E. C., Lagergren, J., Fitzgerald, R. C., Lordick, F., Shah, M. A. et al. (2017). Oesophageal cancer. Nature Reviews Disease Primers*,* 3 *(*1*),* 17048. 10.1038/nrdp.2017.48; 28748917 PMC6168059

[4] Bray, F., Ferlay, J., Soerjomataram, I., Siegel, R. L., Torre, L. A. et al. (2018). Global cancer statistics 2018: GLOBOCAN estimates of incidence and mortality worldwide for 36 cancers in 185 countries. CA: A Cancer Journal for Clinicians*,* 68 *(*6*),* 394–424. 10.3322/caac.21492; 30207593

[5] Morgan, E., Soerjomataram, I., Gavin, A. T., Rutherford, M. J., Gatenby, P. et al. (2021). International trends in oesophageal cancer survival by histological subtype between 1995 and 2014. Gut*,* 70 *(*2*),* 234–242. 10.1136/gutjnl-2020-321089; 32554620

[6] Kelly, R. J., Ajani, J. A., Kuzdzal, J., Zander, T., van Cutsem, E. et al. (2021). Adjuvant nivolumab in resected esophageal or gastroesophageal junction cancer. New England Journal Medicine*,* 384 *(*13*),* 1191–1203. 10.1056/NEJMoa2032125; 33789008

[7] Batista, P. J., Chang, H. Y. (2013). Long noncoding RNAs: Cellular address codes in development and disease. Cell*,* 152 *(*6*),* 1298–1307. 10.1016/j.cell.2013.02.012; 23498938 PMC3651923

[8] Iyer, M. K., Niknafs, Y. S., Malik, R., Singhal, U., Sahu, A. et al. (2015). The landscape of long noncoding RNAs in the human transcriptome. Nature Genetics*,* 47 *(*3*),* 199–208. 10.1038/ng.3192; 25599403 PMC4417758

[9] Schmitt, A. M., Chang, H. Y. (2016). Long noncoding RNAs in cancer pathways. Cancer Cell*,* 29 *(*4*),* 452–463. 10.1016/j.ccell.2016.03.010; 27070700 PMC4831138

[10] Lin, C., Zhang, S., Wang, Y., Wang, Y., Nice, E. et al. (2018). Functional role of a novel long noncoding RNA TTN-AS1 in esophageal squamous cell carcinoma progression and metastasis. Clinical Cancer Research*,* 24 *(*2*),* 486–498. 10.1158/1078-0432.CCR-17-1851; 29101304

[11] Xie, J. J., Jiang, Y. Y., Jiang, Y., Li, C. Q., Lim, M. C. et al. (2018). Super-enhancer-driven long non-coding RNA LINC01503, regulated by TP63, is over-expressed and oncogenic in squamous cell carcinoma. Gastroenterology*,* 154 *(*8*),* 2137–2151.e2131. 10.1053/j.gastro.2018.02.018; 29454790

[12] Huang, L., Wang, Y., Chen, J., Wang, Y., Zhao, Y. et al. (2019). Long noncoding RNA PCAT1, a novel serum-based biomarker, enhances cell growth by sponging miR-326 in oesophageal squamous cell carcinoma. Cell Death & Disease*,* 10 *(*7*),* 513. 10.1038/s41419-019-1745-4; 31273188 PMC6609620

[13] Crooke, S. T., Wang, S., Vickers, T. A., Shen, W., Liang, X. H. (2017). Cellular uptake and trafficking of antisense oligonucleotides. Nature Biotechnology*,* 35 *(*3*),* 230–237. 10.1038/nbt.3779; 28244996

[14] Moss, K. H., Popova, P., Hadrup, S. R., Astakhova, K., Taskova, M. (2019). Lipid nanoparticles for delivery of therapeutic RNA oligonucleotides. Molecular Pharmaceutics*,* 16 *(*6*),* 2265–2277. 10.1021/acs.molpharmaceut.8b01290; 31063396

[15] Tian, L., Yang, L., Zheng, W., Hu, Y., Ding, P. et al. (2020). RNA sequencing of exosomes revealed differentially expressed long noncoding RNAs in early-stage esophageal squamous cell carcinoma and benign esophagitis. Epigenomics*,* 12 *(*6*),* 525–541. 10.2217/epi-2019-0371; 32043367

[16] Livak, K. J., Schmittgen, T. D. (2001). Analysis of relative gene expression data using real-time quantitative PCR and the 2^−ΔΔCT^ method. Methods*,* 25 *(*4*),* 402–408. 10.1006/meth.2001.1262; 11846609

[17] Zhang, B., Zheng, M., Cai, L., Fan, X. (2018). Synthesis and characterization of placental chondroitin sulfate A (plCSA)-targeting lipid-polymer nanoparticles. Journal of Visualized Experiments*,* *(*139*),* 58209. 10.3791/58209; 30295666 PMC6235189

[18] Zhao, K., Cheng, G., Zhang, B., Li, D., Han, J. et al. (2020). Targeting delivery of partial VAR2CSA peptide guided N-2-hydroxypropyl trimethyl ammonium chloride chitosan nanoparticles for multiple cancer types. Materials Science & Engineering C-Materials for Biological Applications*,* 106 *(*10053*),* 110171. 10.1016/j.msec.2019.110171; 31753378

[19] Zhang, B., Cheng, G., Zheng, M., Han, J., Wang, B. et al. (2018). Targeted delivery of doxorubicin by CSA-binding nanoparticles for choriocarcinoma treatment. Drug Delivery*,* 25 *(*1*),* 461–471. 10.1080/10717544.2018.1435750; 29426237 PMC6058719

[20] Alfeghaly, C., Behm-Ansmant, I., Maenner, S. (2021). Study of genome-wide occupancy of long non-coding RNAs using chromatin isolation by RNA purification (ChIRP). Methods in Molecular Biology*,* 2300*,* 107–117. 10.1007/978-1-0716-1386-3 33792876

[21] Sun, Q., Hao, Q., Prasanth, K. V. (2018). Nuclear long noncoding RNAs: Key regulators of gene expression. Trends in Genetics*,* 34 *(*2*),* 142–157. 10.1016/j.tig.2017.11.005; 29249332 PMC6002860

[22] Rashid, F., Shah, A., Shan, G. (2016). Long non-coding RNAs in the cytoplasm. Genomics Proteomics Bioinformatics*,* 14 *(*2*),* 73–80. 10.1016/j.gpb.2016.03.005; 27163185 PMC4880952

[23] Jin, S. J., Jin, M. Z., Xia, B. R., Jin, W. L. (2019). Long non-coding RNA DANCR as an emerging therapeutic target in human cancers. Frontiers in Oncology*,* 9*,* 1225. 10.3389/fonc.2019.01225; 31799189 PMC6874123

[24] Gutschner, T., Hämmerle, M., Eissmann, M., Hsu, J., Kim, Y. et al. (2013). The noncoding RNA MALAT1 is a critical regulator of the metastasis phenotype of lung cancer cells. Cancer Research*,* 73 *(*3*),* 1180–1189. 10.1158/0008-5472.CAN-12-2850; 23243023 PMC3589741

[25] Zhang, X., Liu, N., Zhou, M., Zhang, T., Tian, T. et al. (2019). DNA nanorobot delivers antisense oligonucleotides silencing c-met gene expression for cancer therapy. Journal of Biomedical Nanotechnology*,* 15 *(*9*),* 1948–1959. 10.1166/jbn.2019.2828; 31387681

[26] MacLeod, A. R., Crooke, S. T. (2017). RNA therapeutics in oncology: Advances, challenges, and future directions. The Journal of Clinical Pharmacology*,* 57 *(*S10*),* S43–S59. 10.1002/jcph.957; 28921648

[27] Amreddy, N., Babu, A., Muralidharan, R., Panneerselvam, J., Srivastava, A. et al. (2018). Recent advances in nanoparticle-based cancer drug and gene delivery. Advances in Cancer Research*,* 137 *(*1*),* 115–170. 10.1016/bs.acr.2017.11.003; 29405974 PMC6550462

[28] Castanotto, D., Stein, C. A. (2014). Antisense oligonucleotides in cancer. Current Opinion in Oncology*,* 26 *(*6*),* 584–589. 10.1097/CCO.0000000000000127; 25188471

[29] Peng, Z. H., Kopeček, J. (2015). Enhancing accumulation and penetration of HPMA copolymer-doxorubicin conjugates in 2D and 3D prostate cancer cells via iRGD conjugation with an MMP-2 cleavable spacer. Journal of the American Chemical Society*,* 137 *(*21*),* 6726–6729. 10.1021/jacs.5b00922; 25963409 PMC4855854

[30] Osborn, M. F., Khvorova, A. (2018). Improving siRNA delivery *in vivo* through lipid conjugation. Nucleic Acid Therapeutics*,* 28 *(*3*),* 128–136. 10.1089/nat.2018.0725; 29746209 PMC5994667

[31] Xing, Z., Lin, A., Li, C., Liang, K., Wang, S. et al. (2014). lncRNA directs cooperative epigenetic regulation downstream of chemokine signals. Cell*,* 159 *(*5*),* 1110–1125. 10.1016/j.cell.2014.10.013; 25416949 PMC4266991

[32] Qiao, K., Ning, S., Wan, L., Wu, H., Wang, Q. et al. (2019). LINC00673 is activated by YY1 and promotes the proliferation of breast cancer cells via the miR-515-5p/MARK4/Hippo signaling pathway. Journal of Experimental & Clinical Cancer Research*,* 38 *(*1*),* 418. 10.1186/s13046-019-1421-7; 31623640 PMC6796384

[33] Leung, A. K., Tam, Y. Y., Cullis, P. R. (2014). Lipid nanoparticles for short interfering RNA delivery. Advanced Genetics*,* 88*,* 71–110. 10.1016/B978-0-12-800148-6.00004-3; 25409604 PMC7149983

[34] Kulkarni, J. A., Cullis, P. R., van der Meel, R., (2018). Lipid nanoparticles enabling gene therapies: From concepts to clinical utility. Nucleic Acid Therapeutics*,* 28 *(*3*),* 146–157. 10.1089/nat.2018.0721; 29683383

[35] Campani, V., Salzano, G., Lusa, S., de Rosa, G. (2016). Lipid nanovectors to deliver RNA oligonucleotides in cancer. Nanomaterials*,* 6 *(*7*),* 131. 10.3390/nano6070131; 28335259 PMC5224597

[36] Veiga, N., Goldsmith, M., Granot, Y., Rosenblum, D., Dammes, N. et al. (2018). Cell specific delivery of modified mRNA expressing therapeutic proteins to leukocytes. Nature Communications*,* 9 *(*1*),* 4493. 10.1038/s41467-018-06936-1; 30374059 PMC6206083

[37] Salanti, A., Clausen, T. M., Agerbaek, M. O., Al Nakouzi, N., Dahlback, M. et al. (2015). Targeting human cancer by a glycosaminoglycan binding malaria protein. Cancer Cell*,* 28 *(*4*),* 500–514. 10.1016/j.ccell.2015.09.003; 26461094 PMC4790448

[38] Hentze, M. W., Castello, A., Schwarzl, T., Preiss, T. (2018). A brave new world of RNA-binding proteins. Nature Reviews Molecular Cell Biology*,* 19 *(*5*),* 327–341. 10.1038/nrm.2017.130; 29339797

[39] McHugh, C. A., Chen, C. K., Chow, A., Surka, C. F., Tran, C. et al. (2015). The Xist lncRNA interacts directly with SHARP to silence transcription through HDAC3. Nature*,* 521 *(*7551*),* 232–236. 10.1038/nature14443; 25915022 PMC4516396

[40] Huang, X., Zhang, H., Guo, X., Zhu, Z., Cai, H. et al. (2018). Insulin-like growth factor 2 mRNA-binding protein 1 (IGF2BP1) in cancer. Journal of Hematology & Oncology*,* 11 *(*1*),* 88. 10.1186/s13045-018-0628-y; 29954406 PMC6025799

[41] Lee, T., Paquet, M., Larsson, O., Pelletier, J. (2016). Tumor cell survival dependence on the DHX9 DExH-box helicase. Oncogene*,* 35 *(*39*),* 5093–5105. 10.1038/onc.2016.52; 26973242 PMC5023453

[42] Du, J., Zhang, G., Qiu, H., Yu, H., Yuan, W. (2020). A novel positive feedback loop of linc02042 and c-Myc mediated by YBX1 promotes tumorigenesis and metastasis in esophageal squamous cell carcinoma. Cancer Cell International*,* 20 *(*1*),* 75. 10.1186/s12935-020-1154-x; 32161513 PMC7060651

[43] Wang, Q. S., Shi, L. L., Sun, F., Zhang, Y. F., Chen, R. W. et al. (2019). High expression of ANXA2 pseudogene ANXA2P2 promotes an aggressive phenotype in hepatocellular carcinoma. Disease Markers*,* 2019*,* 9267046. 10.1155/2019/9267046; 30881525 PMC6387700

[44] Liu, Y., Li, Y., Zhao, Y., Liu, Y., Fan, L. et al. (2019). ILF3 promotes gastric cancer proliferation and may be used as a prognostic marker. Molecular Medicine Reports*,* 20 *(*1*),* 125–134. 10.3892/mmr.2019.10229; 31115508 PMC6579973

[45] Jones, V. S., Huang, R. Y., Chen, L. P., Chen, Z. S., Fu, L. et al. (2016). Cytokines in cancer drug resistance: Cues to new therapeutic strategies. Biochimica et Biophysica Acta*,* 1865 *(*2*),* 255–265. 10.1016/j.bbcan.2016.03.005; 26993403

[46] Tan, C., Hu, W., He, Y., Zhang, Y., Zhang, G. et al. (2018). Cytokine-mediated therapeutic resistance in breast cancer. Cytokine*,* 108 *(*1*),* 151–159. 10.1016/j.cyto.2018.03.020; 29609137

[47] Sang, L., Yu, Z., Wang, A., Li, H., Dai, X. et al. (2020). Identification of methylated-differentially expressed genes and pathways in esophageal squamous cell carcinoma. Pathology Research and Practice*,* 216 *(*9*),* 153050. 10.1016/j.prp.2020.153050; 32825936 PMC7283077

[48] Chen, X. G., Ma, L., Xu, J. X. (2018). Abnormal DNA methylation may contribute to the progression of osteosarcoma. Molecular Medicine Reports*,* 17 *(*1*),* 193–199. 10.3892/mmr.2017.7869; 29115427 PMC5780126

[49] Su, X., Zhang, J., Yang, W., Liu, Y., Liu, Y. et al. (2020). Identification of the prognosis-related lncRNAs and genes in gastric cancer. Frontiers in Genetics*,* 11*,* 27. 10.3389/fgene.2020.00027; 32117443 PMC7027194

[50] Ji, M., Tang, L., Ding, R., Shi, L., Liu, A. et al. (2020). Long noncoding RNA-mRNA expression profiles and validation in pancreatic neuroendocrine neoplasms. Clinical Endocrinology*,* 92 *(*4*),* 312–322. 10.1111/cen.14156; 31943312

[51] Zhang, C., Cao, W., Wang, J., Liu, J., Liu, J. et al. (2020). A prognostic long non-coding RNA-associated competing endogenous RNA network in head and neck squamous cell carcinoma. PeerJ*,* 8 *(*6046*),* e9701. 10.7717/peerj.9701; 32983633 PMC7500352

[52] Schmitt, A. M., Chang, H. Y. (2017). Long noncoding RNAs: At the intersection of cancer and chromatin biology. Cold Spring Harbor Perspectives in Medicine*,* 7 *(*7*),* a026492. 10.1101/cshperspect.a026492; 28193769 PMC5495049

[53] Singh, A. P., Luo, H., Matur, M., Eshelman, M. A., Hamamoto, K. et al. (2022). A coordinated function of lncRNA HOTTIP and miRNA-196b underpinning leukemogenesis by targeting FAS signaling. Oncogene*,* 41 *(*5*),* 718–731. 10.1038/s41388-021-02127-3; 34845377 PMC8810734

[54] Vidovic, D., Huynh, T. T., Konda, P., Dean, C., Cruickshank, B. M. et al. (2020). ALDH1A3-regulated long non-coding RNA NRAD1 is a potential novel target for triple-negative breast tumors and cancer stem cells. Cell Death & Differentiation*,* 27 *(*1*),* 363–378. 10.1038/s41418-019-0362-1; 31197235 PMC7206030

[55] Iyer, S., Modali, S. D., Agarwal, S. K. (2017). Long noncoding RNA MEG3 is an epigenetic determinant of oncogenic signaling in functional pancreatic neuroendocrine tumor cells. Molecular and Cellular Biology*,* 37 *(*22*),* e00278. 10.1128/MCB.00278-17; 28847847 PMC5660463

[56] Choudhury, S. R., Dutta, S., Bhaduri, U., Rao, M. R. S. (2021). LncRNA Hmrhl regulates expression of cancer related genes in chronic myelogenous leukemia through chromatin association. NAR Cancer*,* 3 *(*4*),* zcab042. 10.1093/narcan/zcab042; 34734184 PMC8559160

