# Supplementary material for: Targeting LncRNA LLNLR-299G3.1 with antisense oligonucleotide inhibits malignancy of esophageal squamous cell carcinoma cells in vitro and in vivo
Source: Oncol Res. 2023 Jun 27;31(4):463–79. doi: 10.32604/or.2023.028791 (PMC10319589; doi:10.32604/or.2023.028791)
Supplement: SUPPLEMENTARY TABLE S1 [file OncolRes-31-28791-s001.doc]

Supplementary materials

| **Table S1.** Primers, oligos, and probes used in this study | |
| --- | --- |
|  | |
| **Oligo names** | **Sequences (5 ́-3 ́)** |
| Primers for LLNLR-299G3.1 |  |
|  | F: CTTCGGGTAGGACACACGCC |
|  | R: CAGCGATAGCCACCAGCAACT |
| Antisense oligonucleotides (ASOs) |  |
| ENSG00000272448-ASO-246 (ASO1) | GAUAGCCACCAGCAACUUGC |
| ENSG00000272448-ASO-304 (ASO2) | CUUUGGUUUGCACUGCUGGC |
| ENSG00000272448-ASO-480 (ASO3) | UUUCUGGAUGGGUCACAUUC |
| Primers for ENSG00000272448 |  |
| CTG0901 R1 | ATCTATTTCTGGATGGGTCACATT |
| CTG0901 F2 | TTCTCAAAGAGGCACTGCAGGGTC |
| CTG0901 R2 | CATTCATACAGTTCTGAGTTTGAG |
| Primers for 3′RACE |  |
| CTG0901 F3 | AGTTGCTGGTGGCTATCGCTGACG |
| CTG0901 F4 | GCGGGTAAAGTCAGTGCCAGCAGT |
| Primers for 5′RACE |  |
| CTG0901 R3 | ACTGCTGGCACTGACTTTACCCGC |
| CTG0901 R4 | CGTCAGCGATAGCCACCAGCAACT |
| CTG0901 P1 (for sequencing) | GCACACTGCAGCTGGGCTTT |
| Primers for RACE product |  |
| CTG0901 YZF1 | GATGAACAGACTGAAGCTTGGGTG |
| CTG0901 YZF2 | CGGAGGCCAGCAAGCAGAGGTACT |
| Probes for LLNLR-299G3.1 ChIRP |  |
| ChIRP-positive probe 1 | aacctcactacccccatc |
| ChIRP-positive probe 2 | caccagcaacttgcagga |
| ChIRP-positive probe 3 | cttctagaggggtcgagg |
| ChIRP-positive probe 4 | atctgggggtgcagtgaa |
| ChIRP-negative probe | AGAGTTGTTCTTTCCTTTCCT |
| Primers for RNA pull down |  |
| LLNLR-299G3.1-sense-F | TAATACGACTCACTATAGGGTGCTTG  ACCTCCTTGCTTGG |
| LLNLR-299G3.1-sense-R | TTTTTTTTTTTTAGTTGTTATTTTTAAT  TCATCTATTTCTGGATG |
| LLNLR-299G3.1-antisense-F | TGCTTGACCTCCTTGCTTGG |
| LLNLR-299G3.1-antisense-R | TAATACGACTCACTATAGGGCTGGA  TGGGTCACATTCATAC |
| Primers for LLNLR-299G3.1 target genes |  |
| | genOFFTM st-h-OSM_001 | | --- | | | GCCTGGATGTTCCTAAACT | | --- | |
| | genOFFTM st-h-OSM_002 | | --- | | | GCTACCATCGCTTCATGCA | | --- | |
| | genOFFTM st-h-OSM_003 | | --- | | | GGCTGAACATCGAGGACTT | | --- | |
| | genOFFTM st-h-TNFRSF4_001 | | --- |   （si-TNFRSF4） | | GACGCAATCTGTGAGGACA | | --- | |
| | genOFFTM st-h-TNFRSF4_002 | | --- | | | CAGCTACAAGCCTGGAGTT | | --- | |
| | genOFFTM st-h-TNFRSF4_003 | | --- | | | GCGACAACCAGGCCTGCAA | | --- | |
| | genOFFTM st-h-HRH3_001 | | --- | | (si-HRH3) | | | GCGTCACCTTCTTTAACCT | | --- | |
| | genOFFTM st-h-HRH3_002 | | --- | | | CATACACGCTGCTGATGAT | | --- | |
| | genOFFTMst-h-HRH3_003 | | --- | | | GCCGAGTTCTTCTACAACT | | --- | |
| | TNFRSF1-F |  | | --- | --- | | | GCCAACTCTGCACCGTTCT | | --- | |
| TNFRSF4-R | | AAGGTTTTTATTGTGGTCCCG | | --- | |
| OSM-F | | TCCTGGAATTTTCTCACGCA | | --- | |
| OSM-R | | ATGTCCCTCCAAGGAACTGG | | --- | |
| HRH3-F | | CTGCATCCCACTGTATGTACCCTAC | | --- | |
| HRH3-R | | GTCGTAGCTGATGAGCACGATGT | | --- | |
| SSTR3-F | | CAATGGCAAATGCACAGAGTAC | | --- | |
| SSTR3-R | | CTCCCAATGTCCCTGGTAGC |  | | --- | --- | |
| GAPDH-F | GAACGGGAAGCTCACTGG |
| GAPDH-R | GCCTGCTTCACCACCTTCT |

Table S2. LLNLR-299G3.1 binding proteins detected by RNA pull-down and MS

| **Accession** | **Description** | **Score** | **# Unique Peptides** | **# PSMs** | **# AAs** | **MW [kDa]** | **calc. pI** |
| --- | --- | --- | --- | --- | --- | --- | --- |
| Q9NZI8 | IGF2BP1 | 308.947855 | 10 | 12 | 577 | 63.4411789 | 9.20263672 |
| Q08211 | DHX9 | 263.989569 | 12 | 12 | 1270 | 140.869115 | 6.84423828 |
| P67809 | YBX1 | 159.020901 | 3 | 9 | 324 | 35.9026772 | 9.87646484 |
| Q9Y6M1 | IGF2BP | 150.418242 | 3 | 3 | 599 | 66.0806229 | 8.45556641 |
| A6NMY6 | ANXA2P2 | 138.385162 | 6 | 6 | 339 | 38.6348288 | 6.94677734 |
| P16989 | YBX3 | 134.014784 | 1 | 5 | 372 | 40.0660277 | 9.77392578 |
| Q12906 | ILF3 | 122.447428 | 6 | 8 | 894 | 95.2790768 | 8.76318359 |
| P62826 | RAN | 78.76 | 1 | 1 | 216 | 24.4076189 | 7.48876953 |
| P60866 | RPS20 | 72.2338492 | 2 | 2 | 119 | 13.3643091 | 9.93505859 |
| P26038 | MSN | 71.3645204 | 1 | 2 | 577 | 67.7777929 | 6.40478516 |
| P53999 | SUB1 | 70.32 | 1 | 1 | 127 | 14.3864109 | 9.59814453 |
| P39019 | RPS19 | 69.6817035 | 3 | 3 | 145 | 16.0505358 | 10.315918 |
| P31943 | HNRNPH1 | 69.6492083 | 1 | 2 | 449 | 49.1984112 | 6.30322266 |
| O75390 | CS | 65.4384074 | 2 | 2 | 466 | 51.6795158 | 8.32373047 |
| Q00839 | HNRNPU | 64.7077443 | 2 | 2 | 825 | 90.5280091 | 5.99853516 |
| P61247 | RPS3A | 62.34 | 1 | 1 | 264 | 29.9257408 | 9.72998047 |
| P62913 | RPL11 | 60.7 | 2 | 2 | 178 | 20.239641 | 9.59814453 |
| Q15717 | ELAVL1 | 60.35 | 1 | 1 | 326 | 36.0691517 | 9.17333984 |
| Q13610 | PWP1 | 59.37 | 1 | 1 | 501 | 55.7930779 | 4.76708984 |
| P14618 | PKM | 58.3845982 | 3 | 3 | 531 | 57.9000263 | 7.84033203 |
| P62263 | RS14 | 57.41 | 1 | 1 | 151 | 16.2625337 | 10.0522461 |
| P52597 | HNRPF | 53.435802 | 1 | 2 | 415 | 45.6428573 | 5.57958984 |
| Q09666 | AHNK | 52.4 | 1 | 2 | 5890 | 628.699422 | 6.15087891 |
| P22492 | H1T | 50.87 | 1 | 1 | 207 | 22.0056682 | 11.7075195 |
| P23443 | KS6B1 | 49.2853096 | 1 | 2 | 525 | 59.101818 | 6.65380859 |
| Q12905 | ILF2 | 49.12 | 2 | 2 | 390 | 43.0351891 | 5.26220703 |
| P55265 | DSRAD | 48.34 | 1 | 1 | 1226 | 135.9809 | 8.64599609 |
| O15347 | HMGB3 | 48.18 | 1 | 1 | 200 | 22.965443 | 8.36767578 |
| P05455 | LA | 46.5237803 | 2 | 4 | 408 | 46.8081631 | 7.12255859 |
| P46782 | RS5 | 46.4665832 | 2 | 2 | 204 | 22.8620535 | 9.71533203 |
| P52209 | 6PGD | 45.46 | 2 | 2 | 483 | 53.1059558 | 7.22509766 |
| P62701 | RS4X | 45.16 | 1 | 1 | 263 | 29.5790553 | 10.1547852 |
| P31942 | HNRH3 | 41.36 | 1 | 1 | 346 | 36.9030834 | 6.87353516 |
| Q9H361 | PABP3 | 40.46 | 1 | 1 | 631 | 69.9867452 | 9.67138672 |
| P40926 | MDHM | 37.88 | 1 | 1 | 338 | 35.4807344 | 8.67529297 |
| Q15366 | PCBP2 | 37.0354427 | 2 | 2 | 365 | 38.5555934 | 6.78564453 |
| P07910 | HNRPC | 36.62 | 1 | 1 | 306 | 33.6495406 | 5.08447266 |
| O43583 | DENR | 35.55 | 1 | 1 | 198 | 22.0780714 | 5.30029297 |
| P06748 | NPM | 35.19 | 1 | 1 | 294 | 32.5548433 | 4.77978516 |
| P48741 | HSP77 | 33.342141 | 1 | 3 | 367 | 40.2195892 | 7.86962891 |
| P49748 | ACADV | 32.86 | 1 | 1 | 655 | 70.3454318 | 8.74853516 |
| Q5VWN6 | F208B | 32.54 | 1 | 1 | 2430 | 268.675353 | 5.89697266 |
| O95793 | STAU1 | 32.3920498 | 2 | 2 | 577 | 63.1427625 | 9.43701172 |
| Q9H2U1 | DHX36 | 30.97 | 2 | 2 | 1008 | 114.687803 | 7.67919922 |
| P08708 | RS17 | 30.15 | 1 | 1 | 135 | 15.5403906 | 9.84716797 |
| P41091 | IF2G | 29.37 | 1 | 1 | 472 | 51.0772206 | 8.39697266 |
| P37108 | SRP14 | 29.07 | 1 | 2 | 136 | 14.5608177 | 10.0375977 |
| P62906 | RL10A | 28.53 | 1 | 1 | 217 | 24.8155362 | 9.93505859 |
| Q9HB71 | CYBP | 27.86 | 1 | 1 | 228 | 26.1936459 | 8.25048828 |
| P38919 | IF4A3 | 27.44 | 1 | 1 | 411 | 46.8411843 | 6.72705078 |
| P99999 | CYC | 26.7 | 1 | 1 | 105 | 11.741127 | 9.56884766 |
| Q9UHB6 | LIMA1 | 25.23 | 1 | 1 | 759 | 85.1733623 | 6.84423828 |

| Table S3. DE-genes regulated by ASO-LLNLR-299G3.1 | | | | | | |
| --- | --- | --- | --- | --- | --- | --- |
| **Gene_ID** | **Fold Change** | **log2 Fold Change** |  | **P-value** | **q-value** | **Regulation** |
| VAMP7-2 | 2.80E-07 | -21.77008072 |  | 2.57E-08 | 2.54E-06 | Down |
| KRT79 | 0.003566905 | -8.131111636 |  | 3.35E-11 | 6.50E-09 | Down |
| APOL4 | 0.005322596 | -7.55365421 |  | 4.52E-13 | 1.12E-10 | Down |
| CETP | 0.005817114 | -7.425480716 |  | 3.67E-09 | 4.55E-07 | Down |
| CPN2 | 0.006853512 | -7.188940714 |  | 5.08E-09 | 6.09E-07 | Down |
| ASTL | 0.009384567 | -6.735494135 |  | 2.28E-07 | 1.66E-05 | Down |
| SHISA7 | 0.00958588 | -6.704873417 |  | 8.48E-08 | 7.08E-06 | Down |
| CTCFL | 0.01033079 | -6.59690567 |  | 5.45E-07 | 3.55E-05 | Down |
| IL31 | 0.011036204 | -6.501612182 |  | 1.20E-06 | 7.32E-05 | Down |
| KRT76 | 0.011058491 | -6.498701597 |  | 1.02E-13 | 2.92E-11 | Down |
| CLEC1A | 0.011782391 | -6.407223815 |  | 1.61E-06 | 9.59E-05 | Down |
| AMPD1 | 0.012027109 | -6.377566267 |  | 4.09E-07 | 2.78E-05 | Down |
| ZFP3 | 0.012067458 | -6.372734387 |  | 3.16E-06 | 0.000175037 | Down |
| KRT77 | 0.012360478 | -6.338121696 |  | 2.35E-06 | 0.000134831 | Down |
| KRT1 | 0.012364667 | -6.337632784 |  | 2.28E-06 | 0.000130861 | Down |
| HSF5 | 0.012367728 | -6.337275697 |  | 2.54E-06 | 0.000145168 | Down |
| SPDYE21 | 0.012680147 | -6.301284735 |  | 3.00E-06 | 0.000167971 | Down |
| KRT73 | 0.012965044 | -6.269229066 |  | 4.46E-06 | 0.000234799 | Down |
| CFAP97D2 | 0.012986021 | -6.266896739 |  | 3.32E-06 | 0.000181649 | Down |
| MLNR | 0.013895561 | -6.169232076 |  | 1.50E-06 | 8.98E-05 | Down |
| LDHAL6A | 0.014078603 | -6.15035201 |  | 7.96E-06 | 0.000390475 | Down |
| LCT | 0.014212795 | -6.136665925 |  | 1.36E-08 | 1.46E-06 | Down |
| FCN2 | 0.015343563 | -6.026222685 |  | 1.17E-05 | 0.000527828 | Down |
| FAM83C | 0.016077621 | -5.958802274 |  | 5.02E-08 | 4.60E-06 | Down |
| SLC5A5 | 0.01671612 | -5.902616186 |  | 4.84E-06 | 0.000253058 | Down |
| ZAN | 0.016861807 | -5.89009702 |  | 2.59E-05 | 0.001037996 | Down |
| OR5AU1 | 0.016883238 | -5.888264561 |  | 3.46E-05 | 0.001323602 | Down |
| PPP1R27 | 0.017028798 | -5.875879546 |  | 5.58E-06 | 0.000283095 | Down |
| SNX31 | 0.017279887 | -5.854762394 |  | 7.10E-06 | 0.000354361 | Down |
| GIPC3 | 0.017349865 | -5.848931739 |  | 9.06E-06 | 0.000428084 | Down |
| HAPLN4 | 0.017462345 | -5.839608856 |  | 3.03E-05 | 0.001179171 | Down |
| CIB4 | 0.018594067 | -5.749013818 |  | 1.25E-05 | 0.000552504 | Down |
| MEIOB | 0.018730107 | -5.738497048 |  | 5.95E-05 | 0.002081188 | Down |
| LOC105376335 | 0.018776291 | -5.734944113 |  | 2.02E-22 | 1.60E-19 | Down |
| C1orf189 | 0.01897859 | -5.71948336 |  | 1.13E-05 | 0.000519109 | Down |
| ZFR2 | 0.019453597 | -5.683819245 |  | 7.67E-05 | 0.002570855 | Down |
| GLT6D1 | 0.019461093 | -5.683263456 |  | 7.17E-05 | 0.002429909 | Down |
| NRIP3 | 0.019508223 | -5.679773794 |  | 9.87E-05 | 0.003188419 | Down |
| CABP4 | 0.019761591 | -5.661157093 |  | 1.88E-05 | 0.000785785 | Down |
| RNF212 | 0.020235093 | -5.626996682 |  | 9.26E-05 | 0.003029368 | Down |
| DCDC2C | 0.020285081 | -5.623437121 |  | 9.76E-05 | 0.003160149 | Down |
| KRT36 | 0.021100412 | -5.566585011 |  | 0.000158888 | 0.004655177 | Down |
| SRARP | 0.021117847 | -5.565393423 |  | 0.000137816 | 0.004175342 | Down |
| KRT3 | 0.022354104 | -5.483316466 |  | 1.83E-09 | 2.45E-07 | Down |
| TMEM150B | 0.022935958 | -5.446245 |  | 4.23E-05 | 0.001558964 | Down |
| KISS1R | 0.023022829 | -5.440791079 |  | 0.000255135 | 0.006778792 | Down |
| KRT4 | 0.023602947 | -5.404889184 |  | 6.36E-40 | 3.21E-36 | Down |
| TEKT2 | 0.024088761 | -5.375495989 |  | 6.06E-05 | 0.002112513 | Down |
| SLC6A4 | 0.024113175 | -5.374034569 |  | 0.000290176 | 0.007563597 | Down |
| SSMEM1 | 0.024188732 | -5.369521031 |  | 0.000355926 | 0.008953234 | Down |
| SLC35G5 | 0.024201499 | -5.36875979 |  | 0.000405867 | 0.009960872 | Down |
| MOV10L1 | 0.025306496 | -5.304348407 |  | 7.51E-05 | 0.002522197 | Down |
| MC5R | 0.025371562 | -5.300643821 |  | 0.000690239 | 0.015048691 | Down |
| KRT38 | 0.026652326 | -5.229594743 |  | 0.000512971 | 0.012060799 | Down |
| RSPH9 | 0.026652326 | -5.229594743 |  | 0.000512971 | 0.012060799 | Down |
| SRD5A2 | 0.026666476 | -5.228829003 |  | 0.000523495 | 0.012251078 | Down |
| ELANE | 0.026729916 | -5.225400869 |  | 0.000637609 | 0.014154733 | Down |
| HORMAD2 | 0.026755035 | -5.224045771 |  | 0.00073443 | 0.015793903 | Down |
| TNFRSF4 | 0.027374534 | -5.191021788 |  | 0.000142854 | 0.004276554 | Down |
| OPN5 | 0.028011231 | -5.157850812 |  | 0.00151064 | 0.026922823 | Down |
| TMEM204 | 0.028065723 | -5.155046949 |  | 0.000922522 | 0.018472439 | Down |
| MUC21 | 0.0280904 | -5.153779005 |  | 0.001283629 | 0.024006424 | Down |
| HLA-DPB1 | 0.028104302 | -5.153065228 |  | 0.000746318 | 0.015956717 | Down |
| SLC39A12 | 0.028104302 | -5.153065228 |  | 0.000746318 | 0.015956717 | Down |
| KRT72 | 0.028115089 | -5.152511584 |  | 0.000788411 | 0.016583534 | Down |
| ACTN3 | 0.028124827 | -5.152011945 |  | 0.000833844 | 0.017317713 | Down |
| THSD8 | 0.028125653 | -5.151969618 |  | 0.000718735 | 0.015544822 | Down |
| TRIM67 | 0.02815567 | -5.150430708 |  | 0.000156453 | 0.004601675 | Down |
| FGF6 | 0.028189991 | -5.148673154 |  | 0.000823413 | 0.017193864 | Down |
| BAAT | 0.029041796 | -5.105725498 |  | 0.000182162 | 0.005215761 | Down |
| MOBP | 0.029045266 | -5.105553168 |  | 0.000193557 | 0.005417565 | Down |
| SLC6A3 | 0.029729948 | -5.07193923 |  | 2.07E-14 | 6.27E-12 | Down |
| KCNH4 | 0.029732682 | -5.071806564 |  | 0.001424886 | 0.025767263 | Down |
| CEACAM16 | 0.029782993 | -5.06936746 |  | 0.00099495 | 0.019636632 | Down |
| ASB14 | 0.029830708 | -5.067057971 |  | 0.001041759 | 0.020427133 | Down |
| ZNF560 | 0.029873286 | -5.065000239 |  | 0.000225249 | 0.006113658 | Down |
| ASB2 | 0.029924914 | -5.062509088 |  | 0.00021951 | 0.005979387 | Down |
| ACTL7B | 0.030837102 | -5.019189003 |  | 0.000250545 | 0.006668543 | Down |
| GJD4 | 0.031072556 | -5.008215259 |  | 1.20E-05 | 0.000532844 | Down |
| LDHC | 0.031500145 | -4.988497733 |  | 1.77E-05 | 0.000751236 | Down |
| LEFTY1 | 0.031565276 | -4.985517807 |  | 0.001775869 | 0.029869793 | Down |
| TMEM95 | 0.031600127 | -4.98392584 |  | 0.001893448 | 0.031182085 | Down |
| ASPN | 0.031618564 | -4.983084353 |  | 0.002214312 | 0.035163828 | Down |
| C17orf78 | 0.031633783 | -4.982390084 |  | 0.001574105 | 0.027608644 | Down |
| TEX35 | 0.031634718 | -4.982347464 |  | 0.001456907 | 0.026270944 | Down |
| MYBPC3 | 0.031674715 | -4.980524574 |  | 0.00141135 | 0.025583678 | Down |
| FFAR3 | 0.031701767 | -4.979292913 |  | 0.001470005 | 0.026403927 | Down |
| KIF25 | 0.031709842 | -4.9789255 |  | 1.05E-07 | 8.51E-06 | Down |
| ABCG5 | 0.031737079 | -4.977686856 |  | 0.001755858 | 0.029659289 | Down |
| CRX | 0.031845939 | -4.972746789 |  | 0.00041813 | 0.010179202 | Down |
| FAM177B | 0.032890541 | -4.926183464 |  | 0.00038533 | 0.009565555 | Down |
| FAM71A | 0.033328152 | -4.907114876 |  | 1.42E-24 | 1.43E-21 | Down |
| LOC107987243 | 0.033676421 | -4.892117368 |  | 0.00241063 | 0.037378359 | Down |
| HRC | 0.033692542 | -4.891426913 |  | 0.002358706 | 0.036799715 | Down |
| CDHR5 | 0.033746986 | -4.889097525 |  | 0.002033819 | 0.0328146 | Down |
| TARM1 | 0.033776845 | -4.887821609 |  | 0.002383138 | 0.037142564 | Down |
| OR6J1 | 0.033846807 | -4.884836461 |  | 0.002291133 | 0.036080573 | Down |
| RRH | 0.034016431 | -4.877624403 |  | 0.000516564 | 0.012107627 | Down |
| CATSPER4 | 0.034681111 | -4.849706054 |  | 4.20E-05 | 0.001555804 | Down |
| CTRB2 | 0.035953881 | -4.797708695 |  | 4.03E-05 | 0.001507246 | Down |
| RNASE13 | 0.036009336 | -4.795485193 |  | 0.0059192 | 0.069473874 | Down |
| GNAT1 | 0.03609004 | -4.792255455 |  | 0.003327498 | 0.046406935 | Down |
| RLN3 | 0.03612589 | -4.79082305 |  | 0.003734857 | 0.05027922 | Down |
| KRT33A | 0.036144097 | -4.790096129 |  | 0.003125097 | 0.044567541 | Down |
| AMER2 | 0.036152405 | -4.78976457 |  | 0.002949859 | 0.042716447 | Down |
| IHH | 0.036152405 | -4.78976457 |  | 0.002949859 | 0.042716447 | Down |
| SLFN14 | 0.036179787 | -4.788672289 |  | 4.60E-05 | 0.001671803 | Down |
| ORM2 | 0.036213795 | -4.787316832 |  | 0.003052071 | 0.043818808 | Down |
| MLIP | 0.036268236 | -4.785149616 |  | 0.003394624 | 0.047039343 | Down |
| MASP2 | 0.036311268 | -4.78343889 |  | 4.61E-07 | 3.06E-05 | Down |
| ENDOU | 0.036606298 | -4.771764317 |  | 0.000906578 | 0.018312588 | Down |
| GRK7 | 0.037834276 | -4.724162367 |  | 0.001503518 | 0.026867824 | Down |
| ADAM30 | 0.037974162 | -4.718838061 |  | 0.00089856 | 0.018234137 | Down |
| GPHB5 | 0.038854308 | -4.68578164 |  | 0.005505508 | 0.066320538 | Down |
| FGF21 | 0.038907105 | -4.683822558 |  | 0.004393787 | 0.056773742 | Down |
| LRRC43 | 0.038907105 | -4.683822558 |  | 0.004393787 | 0.056773742 | Down |
| CD1A | 0.038926555 | -4.683101505 |  | 0.004832643 | 0.060580341 | Down |
| LOC105374301 | 0.03893839 | -4.682662966 |  | 0.004651208 | 0.058990734 | Down |
| CELA2A | 0.039010202 | -4.680004716 |  | 0.004739497 | 0.05980945 | Down |
| FOXL3 | 0.039083606 | -4.677292616 |  | 0.005834925 | 0.068808419 | Down |
| SIGLEC16 | 0.039083606 | -4.677292616 |  | 0.005834925 | 0.068808419 | Down |
| SAG | 0.039357253 | -4.667226659 |  | 0.001304848 | 0.024323912 | Down |
| SLC28A1 | 0.039487747 | -4.662451131 |  | 0.001050599 | 0.020573785 | Down |
| ETV3L | 0.04099123 | -4.608540918 |  | 0.001585373 | 0.0277725 | Down |
| CTRB1 | 0.041050919 | -4.606441669 |  | 0.001365145 | 0.025046439 | Down |
| CYP27C1 | 0.041063293 | -4.606006876 |  | 0.001386011 | 0.025321288 | Down |
| BTLA | 0.041216836 | -4.600622419 |  | 0.001777128 | 0.029869793 | Down |
| MAS1 | 0.04210281 | -4.56993965 |  | 0.00711814 | 0.077307501 | Down |
| C5orf49 | 0.042115751 | -4.569496294 |  | 0.00679481 | 0.075200542 | Down |
| SERPINB9 | 0.042164337 | -4.567832906 |  | 0.006324534 | 0.071846578 | Down |
| CABP5 | 0.042176608 | -4.56741313 |  | 0.00633175 | 0.071864416 | Down |
| FAM25E | 0.042188775 | -4.566996993 |  | 0.006589072 | 0.073842546 | Down |
| FRMD7 | 0.042201003 | -4.566578915 |  | 0.007182021 | 0.077777788 | Down |
| SPATA32 | 0.042219393 | -4.565950345 |  | 5.96E-11 | 1.11E-08 | Down |
| HDC | 0.042235623 | -4.565395874 |  | 0.00676628 | 0.07493965 | Down |
| THRSP | 0.042236974 | -4.565349706 |  | 0.006870744 | 0.075766211 | Down |
| GDPD4 | 0.042284063 | -4.563742182 |  | 0.006900356 | 0.075766211 | Down |
| TBX21 | 0.042323014 | -4.562413812 |  | 0.007888765 | 0.083341961 | Down |
| CLEC10A | 0.042333213 | -4.562066185 |  | 0.007268576 | 0.078322406 | Down |
| SLC30A10 | 0.042436858 | -4.558538353 |  | 0.009387381 | 0.094162437 | Down |
| TDRD12 | 0.042688607 | -4.550005116 |  | 1.96E-09 | 2.58E-07 | Down |
| NLRP12 | 0.042869685 | -4.543898361 |  | 0.001610806 | 0.027958855 | Down |
| COL21A1 | 0.043866105 | -4.510749573 |  | 3.94E-06 | 0.000208857 | Down |
| PNLDC1 | 0.04445746 | -4.491430652 |  | 0.000190537 | 0.005384186 | Down |
| PSG11 | 0.044854945 | -4.478589152 |  | 0.001976502 | 0.032164427 | Down |
| TBC1D29P | 0.044951911 | -4.475473738 |  | 0.002296511 | 0.036098453 | Down |
| MT1H | 0.045911322 | -4.445006222 |  | 0.012577572 | 0.113625898 | Down |
| PODN | 0.045944137 | -4.443975417 |  | 0.010134551 | 0.098784102 | Down |
| CELA1 | 0.045973889 | -4.443041469 |  | 0.009817042 | 0.096876006 | Down |
| OR7D2 | 0.045988302 | -4.442589251 |  | 0.0101709 | 0.098993802 | Down |
| OR4D1 | 0.046001895 | -4.442162883 |  | 0.009502837 | 0.094765097 | Down |
| HSPB7 | 0.046003778 | -4.442103835 |  | 0.000257544 | 0.006818826 | Down |
| THBS2 | 0.046029169 | -4.441307807 |  | 0.011284274 | 0.10517269 | Down |
| PRDM14 | 0.046044215 | -4.440836289 |  | 0.00987339 | 0.097244256 | Down |
| PCDHGA8 | 0.046087342 | -4.439485633 |  | 0.011191382 | 0.104926937 | Down |
| DLK1 | 0.046145759 | -4.437658119 |  | 0.011389134 | 0.105632475 | Down |
| LOC107985021 | 0.046220315 | -4.435329106 |  | 6.16E-18 | 3.10E-15 | Down |
| KNCN | 0.046235223 | -4.434863857 |  | 0.01195342 | 0.109432044 | Down |
| MUC17 | 0.046665109 | -4.421511914 |  | 7.31E-06 | 0.000363645 | Down |
| TAS1R2 | 0.046944781 | -4.412891404 |  | 0.00261393 | 0.039438524 | Down |
| RENBP | 0.049245442 | -4.34386599 |  | 0.003323124 | 0.046406935 | Down |
| SLC4A1 | 0.049327649 | -4.341459652 |  | 0.003191818 | 0.045181564 | Down |
| LRRN4CL | 0.049360523 | -4.340498517 |  | 0.00320024 | 0.045226742 | Down |
| PRR35 | 0.049472085 | -4.337241473 |  | 0.004188415 | 0.054787192 | Down |
| MMP12 | 0.050465472 | -4.308559539 |  | 0.01763247 | 0.14126533 | Down |
| SEMG1 | 0.050484886 | -4.308004639 |  | 0.016546409 | 0.135655426 | Down |
| CBLL2 | 0.050537821 | -4.306492739 |  | 0.015615367 | 0.131006166 | Down |
| MCHR1 | 0.050538825 | -4.306464063 |  | 0.016608233 | 0.135941127 | Down |
| FAM228A | 0.050573094 | -4.305486158 |  | 0.020084875 | 0.154211857 | Down |
| IL17B | 0.050574382 | -4.305449407 |  | 0.014466084 | 0.125113422 | Down |
| LOC107987285 | 0.050574382 | -4.305449407 |  | 0.014466084 | 0.125113422 | Down |
| RHOH | 0.050589042 | -4.305031263 |  | 0.016665706 | 0.136209076 | Down |
| WNT9B | 0.050607822 | -4.3044958 |  | 0.01477242 | 0.126747699 | Down |
| NPHS1 | 0.050609346 | -4.304452357 |  | 0.01569364 | 0.131444017 | Down |
| NKAPL | 0.050625788 | -4.303983718 |  | 0.014065781 | 0.122775098 | Down |
| APOA4 | 0.05065933 | -4.3030282 |  | 0.01627433 | 0.134444519 | Down |
| DPRX | 0.05065933 | -4.3030282 |  | 0.01627433 | 0.134444519 | Down |
| C17orf98 | 0.050661168 | -4.302975858 |  | 0.01482837 | 0.127152485 | Down |
| GIMAP8 | 0.050679346 | -4.302458296 |  | 0.016769806 | 0.136671656 | Down |
| OR2C1 | 0.050712597 | -4.301512021 |  | 0.014608897 | 0.125629867 | Down |
| MPZ | 0.050731476 | -4.300975054 |  | 0.016356047 | 0.134605728 | Down |
| KLHL40 | 0.050764401 | -4.300039044 |  | 0.014936844 | 0.127445706 | Down |
| WDR49 | 0.050764401 | -4.300039044 |  | 0.014936844 | 0.127445706 | Down |
| NCKAP1L | 0.050783793 | -4.299488046 |  | 0.016415365 | 0.134873639 | Down |
| GNLY | 0.050800487 | -4.299013871 |  | 0.016909806 | 0.137368323 | Down |
| KRTAP4-12 | 0.050817414 | -4.298533218 |  | 0.015922444 | 0.132771929 | Down |
| GABRR1 | 0.050836853 | -4.297981457 |  | 0.01695209 | 0.137490179 | Down |
| OTOP1 | 0.050891362 | -4.296435394 |  | 0.018130502 | 0.144337506 | Down |
| VHLL | 0.051318021 | -4.284390652 |  | 0.000535424 | 0.012395938 | Down |
| SIGLEC11 | 0.052004413 | -4.26522215 |  | 0.004457226 | 0.057250931 | Down |
| ZBP1 | 0.054070273 | -4.209020537 |  | 8.78E-16 | 3.13E-13 | Down |
| LRTM2 | 0.05473893 | -4.191288952 |  | 0.005863283 | 0.069035139 | Down |
| KRT2 | 0.054741264 | -4.191227452 |  | 0.005265189 | 0.06445273 | Down |
| MT1A | 0.054811961 | -4.189365443 |  | 0.005114444 | 0.063118497 | Down |
| MEIOSIN | 0.054842033 | -4.188574142 |  | 0.005183186 | 0.063738115 | Down |
| FOXD4L5 | 0.054854756 | -4.188239484 |  | 0.00631532 | 0.071839739 | Down |
| CD79A | 0.055222965 | -4.178587845 |  | 3.72E-05 | 0.001403196 | Down |
| GPBAR1 | 0.055272231 | -4.177301345 |  | 3.78E-05 | 0.001423312 | Down |
| GH1 | 0.056087156 | -4.156185749 |  | 0.025238376 | 0.176400263 | Down |
| AMHR2 | 0.056121321 | -4.155307222 |  | 4.85E-05 | 0.001758392 | Down |
| HRH3 | 0.056132915 | -4.155009204 |  | 0.023690592 | 0.170073034 | Down |
| CAPN11 | 0.056176098 | -4.153899773 |  | 0.02536968 | 0.176823178 | Down |
| OVCH1 | 0.056261034 | -4.151720122 |  | 0.021935442 | 0.16280071 | Down |
| C3orf56 | 0.056346346 | -4.149534135 |  | 0.022049101 | 0.163244105 | Down |
| LAX1 | 0.056346346 | -4.149534135 |  | 0.022049101 | 0.163244105 | Down |
| C3orf22 | 0.05636829 | -4.148972378 |  | 0.022078444 | 0.163298396 | Down |
| FAM186A | 0.056391513 | -4.148378136 |  | 0.024056958 | 0.171069184 | Down |
| GLYATL3 | 0.056411664 | -4.147862705 |  | 0.024458525 | 0.173029471 | Down |
| ZNF404 | 0.056483895 | -4.146016619 |  | 0.033545452 | 0.209648676 | Down |
| BEST2 | 0.056592011 | -4.143257781 |  | 0.028901459 | 0.191636955 | Down |
| ABCA9 | 0.05753472 | -4.119423353 |  | 0.000822758 | 0.017193864 | Down |
| LDHAL6B | 0.057987589 | -4.108112025 |  | 0.006878512 | 0.075766211 | Down |
| GRM2 | 0.058688349 | -4.090782077 |  | 1.00E-09 | 1.45E-07 | Down |
| GRK1 | 0.0590473 | -4.081985099 |  | 1.50E-11 | 2.99E-09 | Down |
| GP5 | 0.059475128 | -4.071569718 |  | 0.000189876 | 0.005375895 | Down |
| CFAP157 | 0.05976272 | -4.064610384 |  | 0.0015277 | 0.027015905 | Down |
| FOXD4L4 | 0.061378351 | -4.026126313 |  | 0.001820457 | 0.030377121 | Down |
| DRC7 | 0.061655197 | -4.019633688 |  | 0.008567091 | 0.08798728 | Down |
| PLA2G2F | 0.061693125 | -4.018746463 |  | 0.008627992 | 0.088193368 | Down |
| GRP | 0.061731315 | -4.017853661 |  | 0.008808677 | 0.089676482 | Down |
| PLSCR2 | 0.061864694 | -4.014739888 |  | 0.010575093 | 0.101507462 | Down |
| PVALEF | 0.062605997 | -3.997555342 |  | 0.001632371 | 0.028203645 | Down |
| CA5A | 0.062664408 | -3.996209932 |  | 0.001670596 | 0.028667506 | Down |
| AIPL1 | 0.063027301 | -3.987879307 |  | 0.046973025 | 0.25380207 | Down |
| SGCA | 0.063027301 | -3.987879307 |  | 0.046973025 | 0.25380207 | Down |
| TRIM72 | 0.063117719 | -3.98581111 |  | 0.036852307 | 0.219295529 | Down |
| CIDEA | 0.063146583 | -3.985151525 |  | 0.035615346 | 0.215597222 | Down |
| TEX101 | 0.063146583 | -3.985151525 |  | 0.035615346 | 0.215597222 | Down |
| WFIKKN2 | 0.063164802 | -3.984735332 |  | 0.049151508 | 0.259025299 | Down |
| DNAJB8 | 0.063174411 | -3.984515889 |  | 0.035664465 | 0.21567015 | Down |
| ZNF799 | 0.063198679 | -3.983961783 |  | 0.03699812 | 0.21952024 | Down |
| CORIN | 0.063255804 | -3.982658331 |  | 0.033234459 | 0.20856727 | Down |
| SYT4 | 0.063255804 | -3.982658331 |  | 0.033234459 | 0.20856727 | Down |
| TSPO2 | 0.063255804 | -3.982658331 |  | 0.033234459 | 0.20856727 | Down |
| ZNF501 | 0.063255804 | -3.982658331 |  | 0.033234459 | 0.20856727 | Down |
| MUC8 | 0.063283272 | -3.982031991 |  | 0.03408977 | 0.210784926 | Down |
| UNCX | 0.063310903 | -3.981402214 |  | 0.037201174 | 0.220120295 | Down |
| F11 | 0.063335761 | -3.980835887 |  | 0.033368858 | 0.208905755 | Down |
| GSX2 | 0.063335761 | -3.980835887 |  | 0.033368858 | 0.208905755 | Down |
| ABCC12 | 0.063339866 | -3.980742372 |  | 0.049546536 | 0.260290384 | Down |
| TMPRSS11B | 0.063339866 | -3.980742372 |  | 0.049546536 | 0.260290384 | Down |
| IFNA6 | 0.063391652 | -3.979563322 |  | 0.047222023 | 0.2542388 | Down |
| OPALIN | 0.063391946 | -3.97955664 |  | 0.036049276 | 0.217128669 | Down |
| TMEM31 | 0.063391946 | -3.97955664 |  | 0.036049276 | 0.217128669 | Down |
| GYS2 | 0.063417013 | -3.978986266 |  | 0.036093567 | 0.217135909 | Down |
| MYOCOS | 0.063417013 | -3.978986266 |  | 0.036093567 | 0.217135909 | Down |
| IL13 | 0.063424063 | -3.978825896 |  | 0.047847982 | 0.255787053 | Down |
| CABP7 | 0.063444107 | -3.978370031 |  | 0.034364945 | 0.211541472 | Down |
| FAT3 | 0.063527493 | -3.976475093 |  | 0.037594113 | 0.221282436 | Down |
| TSSK2 | 0.063527493 | -3.976475093 |  | 0.037594113 | 0.221282436 | Down |
| GUCA2A | 0.063555791 | -3.975832599 |  | 0.037645733 | 0.221282436 | Down |
| MRC1 | 0.063976075 | -3.966323706 |  | 4.36E-22 | 3.30E-19 | Down |
| PRRT1B | 0.06547877 | -3.932828973 |  | 0.0182924 | 0.145320289 | Down |
| KRT31 | 0.065712249 | -3.92769387 |  | 0.014444627 | 0.125070944 | Down |
| HK3 | 0.065746016 | -3.926952723 |  | 0.011555218 | 0.106714589 | Down |
| TNNI1 | 0.065774367 | -3.926330736 |  | 0.011224182 | 0.104977567 | Down |
| ZNF709 | 0.065774369 | -3.926330697 |  | 0.011165986 | 0.104806786 | Down |
| RTP2 | 0.065802785 | -3.925707549 |  | 0.011754019 | 0.108042388 | Down |
| GPR25 | 0.065939416 | -3.922715075 |  | 0.002297044 | 0.036098453 | Down |
| TAT | 0.06718683 | -3.895677723 |  | 0.002506032 | 0.038268876 | Down |
| GPRC5D | 0.067725426 | -3.88415862 |  | 0.002200747 | 0.035058897 | Down |
| LCNL1 | 0.068746746 | -3.862564756 |  | 0.000218192 | 0.005954203 | Down |
| LRIT3 | 0.070220191 | -3.831970265 |  | 0.003250883 | 0.045803214 | Down |
| RNF212B | 0.070399118 | -3.828298841 |  | 0.021214726 | 0.159564289 | Down |
| ZNF750 | 0.070485343 | -3.826532894 |  | 0.016684691 | 0.136271828 | Down |
| KRT74 | 0.070584087 | -3.824513218 |  | 0.016327566 | 0.134444519 | Down |
| DPEP1 | 0.070594849 | -3.824293273 |  | 0.000248835 | 0.006646585 | Down |
| PCDHB7 | 0.070652893 | -3.823107554 |  | 0.016902758 | 0.137368323 | Down |
| LOC112268119 | 0.070912863 | -3.817808842 |  | 0.002572371 | 0.039084532 | Down |
| SPEM3 | 0.07226661 | -3.790526966 |  | 2.85E-08 | 2.76E-06 | Down |
| SPDYA | 0.074127877 | -3.753839996 |  | 1.95E-15 | 6.55E-13 | Down |
| SERPIND1 | 0.074129095 | -3.753816294 |  | 4.70E-09 | 5.69E-07 | Down |
| HAMP | 0.074374595 | -3.749046277 |  | 0.000379823 | 0.009444361 | Down |
| INHBC | 0.074811776 | -3.740590802 |  | 8.89E-07 | 5.53E-05 | Down |
| SMIM41 | 0.075522532 | -3.726949058 |  | 8.13E-07 | 5.14E-05 | Down |
| OR13A1 | 0.075663483 | -3.724259002 |  | 0.022991363 | 0.167115275 | Down |
| SYT5 | 0.07582011 | -3.721275641 |  | 1.36E-10 | 2.29E-08 | Down |
| HOXB1 | 0.075823463 | -3.721211846 |  | 0.020202142 | 0.154272109 | Down |
| RIIAD1 | 0.075878417 | -3.720166606 |  | 0.023360593 | 0.168736476 | Down |
| CHGA | 0.076135811 | -3.715280993 |  | 0.022476395 | 0.165148053 | Down |
| PRAM1 | 0.076156173 | -3.714895208 |  | 0.022503307 | 0.165148053 | Down |
| SERPINC1 | 0.076423857 | -3.709833127 |  | 0.000472182 | 0.011223985 | Down |
| LOC102724265 | 0.076744456 | -3.703793649 |  | 0.004590588 | 0.058508839 | Down |
| PKLR | 0.076880554 | -3.701237455 |  | 0.003941067 | 0.052540615 | Down |
| CYP2C18 | 0.076999808 | -3.699001333 |  | 0.000606551 | 0.013645588 | Down |
| STPG3 | 0.07703023 | -3.698431448 |  | 0.004368278 | 0.056686372 | Down |
| CLDN11 | 0.077143166 | -3.696317838 |  | 2.17E-07 | 1.58E-05 | Down |
| GPR4 | 0.079534011 | -3.65228426 |  | 1.57E-07 | 1.19E-05 | Down |
| TM4SF18 | 0.080126272 | -3.641580827 |  | 0.006052199 | 0.070220377 | Down |
| KRT6B | 0.080270136 | -3.638992842 |  | 0.0049548 | 0.061651577 | Down |
| FOXD4L6 | 0.082196858 | -3.604772946 |  | 0.028670423 | 0.191026645 | Down |
| NOSTRIN | 0.08237663 | -3.601621091 |  | 0.027455698 | 0.185654942 | Down |
| MASP1 | 0.082427524 | -3.600730037 |  | 0.030414325 | 0.198447892 | Down |
| TBC1D26 | 0.083141449 | -3.588288298 |  | 0.006557535 | 0.073707663 | Down |
| OIT3 | 0.083527825 | -3.58159932 |  | 3.14E-07 | 2.21E-05 | Down |
| COLEC11 | 0.083849317 | -3.576057163 |  | 0.000827264 | 0.017226682 | Down |
| SLC5A11 | 0.085613981 | -3.546009783 |  | 1.71E-07 | 1.28E-05 | Down |
| LRRC14B | 0.085710626 | -3.544382116 |  | 5.60E-07 | 3.63E-05 | Down |
| FSD2 | 0.087010692 | -3.522663495 |  | 2.04E-07 | 1.50E-05 | Down |
| SLFNL1 | 0.08796944 | -3.506853756 |  | 0.008389127 | 0.086630343 | Down |
| EID3 | 0.088082738 | -3.504996873 |  | 3.11E-08 | 2.97E-06 | Down |
| MPIG6B | 0.088240833 | -3.502409773 |  | 0.011065807 | 0.104199771 | Down |
| F2RL3 | 0.089463444 | -3.482557891 |  | 0.039059878 | 0.226943597 | Down |
| C19orf84 | 0.089522114 | -3.48161209 |  | 0.036383624 | 0.217840645 | Down |
| SLC8A3 | 0.089657495 | -3.479431993 |  | 0.035959674 | 0.216934695 | Down |
| TNFSF18 | 0.089657542 | -3.479431245 |  | 0.035755249 | 0.215904135 | Down |
| MRLN | 0.089817397 | -3.476861284 |  | 0.036142152 | 0.217168942 | Down |
| STAR | 0.089845597 | -3.476408381 |  | 0.036771268 | 0.219033895 | Down |
| H2BC6 | 0.090890035 | -3.459734053 |  | 5.84E-05 | 0.002052773 | Down |
| ADAM20 | 0.09107261 | -3.456838959 |  | 9.58E-12 | 1.93E-09 | Down |
| GPR52 | 0.091162053 | -3.455422774 |  | 0.011433328 | 0.105912412 | Down |
| RPS6KL1 | 0.091830124 | -3.444888696 |  | 0.001574193 | 0.027608644 | Down |
| SPATA22 | 0.092534736 | -3.433861166 |  | 0.010898718 | 0.103561799 | Down |
| ID2 | 0.093548811 | -3.418136867 |  | 8.32E-16 | 3.13E-13 | Down |
| HTRA4 | 0.094123157 | -3.409306486 |  | 1.19E-10 | 2.06E-08 | Down |
| ELAVL3 | 0.095352893 | -3.390579474 |  | 1.36E-05 | 0.000594247 | Down |
| FRZB | 0.096026867 | -3.380418086 |  | 0.000550318 | 0.012605614 | Down |
| PROZ | 0.096345076 | -3.375645249 |  | 0.000107458 | 0.003391556 | Down |
| GAL3ST1 | 0.097176865 | -3.363243303 |  | 3.08E-06 | 0.00017166 | Down |
| MMP25 | 0.098068916 | -3.350060263 |  | 7.86E-58 | 1.19E-53 | Down |
| NPC1L1 | 0.100280396 | -3.317888494 |  | 6.04E-14 | 1.76E-11 | Down |
| STAB1 | 0.100431388 | -3.315717871 |  | 5.96E-08 | 5.34E-06 | Down |
| CNGA4 | 0.100518657 | -3.314464789 |  | 0.016278179 | 0.134444519 | Down |
| GSG1L2 | 0.100573585 | -3.313676651 |  | 0.016306294 | 0.134444519 | Down |
| CLCA2 | 0.100611548 | -3.313132191 |  | 0.016325781 | 0.134444519 | Down |
| SLFN11 | 0.10127374 | -3.303667963 |  | 3.35E-05 | 0.001290068 | Down |
| ASB5 | 0.101528069 | -3.300049455 |  | 0.014938073 | 0.127445706 | Down |
| TMEM74 | 0.101601904 | -3.299000654 |  | 0.019670422 | 0.152033457 | Down |
| ABCA6 | 0.101752751 | -3.2968603 |  | 0.00346327 | 0.047433813 | Down |
| DDC | 0.102190794 | -3.290662854 |  | 5.72E-05 | 0.002025069 | Down |
| CLEC7A | 0.102811976 | -3.281919776 |  | 0.003746971 | 0.050352623 | Down |
| SSTR3 | 0.102835957 | -3.281583298 |  | 0.01719053 | 0.138501689 | Down |
| C11orf94 | 0.102854694 | -3.281320466 |  | 0.004648399 | 0.058990734 | Down |
| FOXE3 | 0.103905557 | -3.266655279 |  | 3.16E-06 | 0.000175037 | Down |
| SH2D4B | 0.106530891 | -3.230656258 |  | 1.51E-12 | 3.30E-10 | Down |
| CAMKV | 0.10684551 | -3.226401812 |  | 0.004939231 | 0.061508484 | Down |
| TSGA10IP | 0.107523666 | -3.217273866 |  | 0.000302109 | 0.007834114 | Down |
| CD226 | 0.107575714 | -3.216575679 |  | 0.023213375 | 0.168235764 | Down |
| GPR84 | 0.10759081 | -3.216373243 |  | 0.018834976 | 0.148074448 | Down |
| CCDC166 | 0.10798185 | -3.211139261 |  | 2.18E-05 | 0.000897238 | Down |
| PLVAP | 0.108436028 | -3.205083918 |  | 0.025220036 | 0.176353607 | Down |
| NUP210L | 0.108765319 | -3.200709481 |  | 1.19E-09 | 1.68E-07 | Down |
| SCUBE2 | 0.109376876 | -3.192620329 |  | 3.85E-13 | 9.69E-11 | Down |
| POU4F1 | 0.110158765 | -3.182343808 |  | 0.00567405 | 0.067508166 | Down |
| TXNDC2 | 0.11016013 | -3.182325926 |  | 0.012089127 | 0.110282467 | Down |
| PLD4 | 0.110207981 | -3.18169939 |  | 0.004461482 | 0.057256951 | Down |
| ICAM4 | 0.11044731 | -3.178569812 |  | 1.86E-10 | 3.06E-08 | Down |
| MYOM1 | 0.110450696 | -3.17852559 |  | 5.91E-08 | 5.34E-06 | Down |
| CCDC89 | 0.110877139 | -3.172966164 |  | 0.006096612 | 0.070627266 | Down |
| PPEF2 | 0.114061162 | -3.132120457 |  | 0.025023853 | 0.175468741 | Down |
| ZDHHC19 | 0.114944145 | -3.120995112 |  | 0.001708572 | 0.029165565 | Down |
| B4GALNT2 | 0.1149763 | -3.120591585 |  | 0.007990224 | 0.084061379 | Down |
| RAB44 | 0.115537711 | -3.113564275 |  | 0.001599244 | 0.027822069 | Down |
| GSC2 | 0.115860677 | -3.109537096 |  | 0.000193868 | 0.005417565 | Down |
| SUMO4 | 0.115888764 | -3.1091874 |  | 0.030334729 | 0.198270833 | Down |
| PDE6G | 0.116692081 | -3.099221437 |  | 1.22E-07 | 9.59E-06 | Down |
| GADD45G | 0.119011911 | -3.070822122 |  | 1.60E-46 | 1.21E-42 | Down |
| TRIM59 | 0.11941046 | -3.06599888 |  | 0.010242067 | 0.099292374 | Down |
| FBXW10 | 0.120099395 | -3.057699208 |  | 2.75E-16 | 1.07E-13 | Down |
| MSH4 | 0.120820988 | -3.04905701 |  | 1.34E-07 | 1.04E-05 | Down |
| CARMIL2 | 0.120841955 | -3.048806671 |  | 9.63E-24 | 9.10E-21 | Down |
| LRCOL1 | 0.121907042 | -3.036146628 |  | 0.031411177 | 0.202074115 | Down |
| CDKL4 | 0.122725315 | -3.026495222 |  | 8.90E-16 | 3.13E-13 | Down |
| USP50 | 0.123512844 | -3.01726702 |  | 0.036738452 | 0.218947117 | Down |
| SLC6A13 | 0.124315003 | -3.007927674 |  | 8.90E-08 | 7.35E-06 | Down |
| KLKB1 | 0.125948816 | -2.989090532 |  | 0.010032284 | 0.098294281 | Down |
| PDE4C | 0.126083696 | -2.987546358 |  | 1.02E-06 | 6.30E-05 | Down |
| HIPK4 | 0.127994496 | -2.965846319 |  | 0.000283565 | 0.007442595 | Down |
| DNAH17 | 0.130281097 | -2.940300322 |  | 2.19E-18 | 1.23E-15 | Down |
| SLC7A3 | 0.130611898 | -2.93664177 |  | 0.039290394 | 0.227679384 | Down |
| KLRC2 | 0.13096774 | -2.9327166 |  | 0.045463297 | 0.248756471 | Down |
| FOXD4L3 | 0.1315966 | -2.925805884 |  | 0.000394216 | 0.009738167 | Down |
| F2 | 0.131605704 | -2.925706077 |  | 6.00E-05 | 0.002095212 | Down |
| INSL4 | 0.132215875 | -2.919032686 |  | 0.004720793 | 0.059673032 | Down |
| SPIB | 0.132365896 | -2.91739664 |  | 4.92E-10 | 7.74E-08 | Down |
| TAGLN3 | 0.132642524 | -2.91438473 |  | 0.00625592 | 0.071548775 | Down |
| OR10H1 | 0.13269394 | -2.913825607 |  | 2.60E-09 | 3.36E-07 | Down |
| SLX1B | 0.132714603 | -2.913600971 |  | 0.001379995 | 0.025288205 | Down |
| SIX3 | 0.132839551 | -2.912243341 |  | 0.018519024 | 0.146889087 | Down |
| MUC13 | 0.133984045 | -2.89986688 |  | 0.000284232 | 0.007447168 | Down |
| FSCN3 | 0.135949724 | -2.878854871 |  | 0.000110403 | 0.003462818 | Down |
| POU5F2 | 0.137683578 | -2.860571597 |  | 0.018006104 | 0.143801519 | Down |
| DLL4 | 0.137870816 | -2.858610991 |  | 3.65E-26 | 4.59E-23 | Down |
| DPEP2NB | 0.138497096 | -2.852072366 |  | 0.022904098 | 0.166926651 | Down |
| MYOZ3 | 0.138713082 | -2.849824237 |  | 0.016528439 | 0.135581626 | Down |
| LTA | 0.139351152 | -2.843203163 |  | 9.53E-11 | 1.72E-08 | Down |
| MATN4 | 0.13954122 | -2.841236747 |  | 9.95E-10 | 1.45E-07 | Down |
| LIN28A | 0.142793362 | -2.807999185 |  | 3.26E-09 | 4.11E-07 | Down |
| SLC7A9 | 0.145223023 | -2.783657908 |  | 7.49E-08 | 6.47E-06 | Down |
| KCNE4 | 0.145860408 | -2.777339762 |  | 0.021127339 | 0.159126934 | Down |
| ALLC | 0.146053793 | -2.775428266 |  | 0.000352595 | 0.008899056 | Down |
| CYP2C19 | 0.146362236 | -2.772384733 |  | 0.024118913 | 0.171187663 | Down |
| TUBB1 | 0.146599342 | -2.770049464 |  | 1.17E-07 | 9.28E-06 | Down |
| ERICH4 | 0.147069824 | -2.765426831 |  | 0.011353131 | 0.105492711 | Down |
| OSM | 0.148024905 | -2.756088163 |  | 0.008115369 | 0.085140975 | Down |
| TNFSF14 | 0.148329473 | -2.753122802 |  | 3.20E-06 | 0.000176121 | Down |
| ARHGAP9 | 0.148601444 | -2.750479958 |  | 1.52E-08 | 1.61E-06 | Down |
| SEPTIN12 | 0.14921344 | -2.74455061 |  | 0.00010512 | 0.003331679 | Down |
| LST1 | 0.15065047 | -2.730722917 |  | 0.000127986 | 0.003916799 | Down |
| STPG4 | 0.152720326 | -2.711036003 |  | 8.06E-20 | 4.69E-17 | Down |
| TMEM88 | 0.153056771 | -2.707861224 |  | 0.00531111 | 0.064742047 | Down |
| TAC3 | 0.1533215 | -2.705368072 |  | 0.030914958 | 0.200588986 | Down |
| WDR86 | 0.153433137 | -2.704317997 |  | 0.009898207 | 0.0973592 | Down |
| DLX2 | 0.154121249 | -2.69786231 |  | 2.33E-08 | 2.35E-06 | Down |
| CXCR4 | 0.154903405 | -2.690559237 |  | 0.031095459 | 0.201414377 | Down |
| FBLL1 | 0.156091911 | -2.679532315 |  | 6.79E-05 | 0.002319871 | Down |
| SLC44A5 | 0.156448777 | -2.676237717 |  | 0.006258935 | 0.071548775 | Down |
| EVI2A | 0.157332017 | -2.668115803 |  | 6.83E-05 | 0.002327175 | Down |
| SLC44A4 | 0.160145967 | -2.642540627 |  | 0.034063566 | 0.210773175 | Down |
| LOC101928268 | 0.16135799 | -2.631663081 |  | 0.018819117 | 0.14802675 | Down |
| LGALS4 | 0.161462193 | -2.630731707 |  | 3.10E-13 | 7.93E-11 | Down |
| MYBPHL | 0.162062561 | -2.625377249 |  | 0.030499478 | 0.198746166 | Down |
| MPP4 | 0.163290737 | -2.614485142 |  | 0.000657149 | 0.014503317 | Down |
| IQCA1L | 0.163299457 | -2.614408102 |  | 0.040814695 | 0.233460671 | Down |
| SLC35D3 | 0.163516888 | -2.61248845 |  | 0.036329085 | 0.217600283 | Down |
| TMEM236 | 0.16481958 | -2.601040458 |  | 1.58E-14 | 4.96E-12 | Down |
| SAA2 | 0.164848597 | -2.600786489 |  | 0.001143731 | 0.021970674 | Down |
| TMEM225B | 0.165440929 | -2.595611901 |  | 0.007930741 | 0.083668492 | Down |
| KLF17 | 0.165835029 | -2.592179318 |  | 0.009603447 | 0.095324329 | Down |
| SLC39A2 | 0.165933755 | -2.591320702 |  | 0.015111614 | 0.128346844 | Down |
| HCLS1 | 0.166482671 | -2.586556081 |  | 6.23E-07 | 4.03E-05 | Down |
| C6orf201 | 0.166587226 | -2.585650313 |  | 0.023379814 | 0.168794662 | Down |
| KRT78 | 0.168079341 | -2.572785686 |  | 2.84E-18 | 1.54E-15 | Down |
| TTLL10 | 0.16823245 | -2.571472088 |  | 1.21E-10 | 2.08E-08 | Down |
| LOC107987373 | 0.168284321 | -2.571027328 |  | 0.004120513 | 0.054262998 | Down |
| SLC27A5 | 0.169606853 | -2.559733633 |  | 0.005231641 | 0.064102255 | Down |
| NR5A1 | 0.169723836 | -2.558738906 |  | 0.002107392 | 0.033785318 | Down |
| ADAM32 | 0.170524116 | -2.551952315 |  | 7.43E-13 | 1.73E-10 | Down |
| GCNA | 0.170982934 | -2.548075762 |  | 0.000793366 | 0.016658474 | Down |
| CTRL | 0.172446189 | -2.535781851 |  | 5.04E-05 | 0.001808977 | Down |
| SLC26A7 | 0.17300343 | -2.531127456 |  | 0.022430743 | 0.164935784 | Down |
| LDLRAD4 | 0.173268083 | -2.528922169 |  | 0.001566388 | 0.027567697 | Down |
| G6PC1 | 0.176573049 | -2.501662938 |  | 0.029638926 | 0.194899342 | Down |
| ASGR2 | 0.1776412 | -2.492961875 |  | 0.013615822 | 0.120250524 | Down |
| RSPH6A | 0.177966681 | -2.490320932 |  | 0.013981475 | 0.122321722 | Down |
| H2BC10 | 0.178930326 | -2.482530175 |  | 0.001237659 | 0.02341793 | Down |
| MYLK2 | 0.179776616 | -2.475722715 |  | 8.75E-17 | 3.78E-14 | Down |
| SLC22A14 | 0.179779612 | -2.475698676 |  | 1.66E-09 | 2.24E-07 | Down |
| GPR83 | 0.179857437 | -2.47507428 |  | 0.000409701 | 0.010038663 | Down |
| CSPG5 | 0.180318856 | -2.471377828 |  | 2.22E-06 | 0.00012834 | Down |
| PYGM | 0.180985907 | -2.46605073 |  | 8.20E-21 | 5.63E-18 | Down |
| FERMT3 | 0.181630076 | -2.460924977 |  | 2.31E-16 | 9.42E-14 | Down |
| NIM1K | 0.182571038 | -2.453470171 |  | 0.000688173 | 0.015034387 | Down |
| PLA2G4E | 0.182820405 | -2.451500992 |  | 0.02063238 | 0.156665152 | Down |
| CDHR2 | 0.18332074 | -2.447558082 |  | 0.013490551 | 0.119478705 | Down |
| FAM209A | 0.183582761 | -2.445497502 |  | 0.004493874 | 0.057574903 | Down |
| RPL3L | 0.184578986 | -2.43768978 |  | 0.000574635 | 0.013044045 | Down |
| KIF5A | 0.185081488 | -2.433767493 |  | 0.000191273 | 0.005384953 | Down |
| GFRA3 | 0.185953836 | -2.426983586 |  | 0.01855216 | 0.147074752 | Down |
| CACNA1B | 0.188072397 | -2.410639968 |  | 8.87E-16 | 3.13E-13 | Down |
| WFIKKN1 | 0.189086144 | -2.402884445 |  | 0.00262414 | 0.039474379 | Down |
| FAM71D | 0.190275768 | -2.39383625 |  | 0.024641118 | 0.173752062 | Down |
| PLEKHD1 | 0.190662359 | -2.390908046 |  | 7.40E-06 | 0.000365406 | Down |
| CD7 | 0.190785686 | -2.38997516 |  | 0.008544521 | 0.087815134 | Down |
| NACAD | 0.191371443 | -2.385552535 |  | 1.61E-16 | 6.76E-14 | Down |
| CFAP65 | 0.192856682 | -2.37439896 |  | 0.02784224 | 0.187324869 | Down |
| TEK | 0.192991497 | -2.37339081 |  | 0.041606255 | 0.237114943 | Down |
| CTRC | 0.193177221 | -2.372003111 |  | 0.006325426 | 0.071846578 | Down |
| IMPG2 | 0.193241902 | -2.371520136 |  | 6.00E-11 | 1.11E-08 | Down |
| GSDMA | 0.193315269 | -2.370972502 |  | 0.032111304 | 0.204834889 | Down |
| LRRC15 | 0.193333082 | -2.370839573 |  | 1.48E-17 | 6.98E-15 | Down |
| CDHR4 | 0.193972418 | -2.366076571 |  | 0.033892991 | 0.210169911 | Down |
| TERB1 | 0.195017017 | -2.358328081 |  | 0.036995747 | 0.21952024 | Down |
| RSPH4A | 0.195324352 | -2.356056266 |  | 0.005084548 | 0.062852169 | Down |
| CYP2E1 | 0.196524792 | -2.347216771 |  | 0.011406543 | 0.10572907 | Down |
| H3C3 | 0.196882634 | -2.344592227 |  | 1.01E-06 | 6.27E-05 | Down |
| C20orf204 | 0.197337883 | -2.341260161 |  | 3.13E-09 | 3.99E-07 | Down |
| ADCY10 | 0.197391788 | -2.340866124 |  | 0.000599737 | 0.013512405 | Down |
| KRT14 | 0.197678396 | -2.338772887 |  | 0.009960696 | 0.097719532 | Down |
| SPI1 | 0.197896437 | -2.33718246 |  | 0.000189623 | 0.005375895 | Down |
| IL16 | 0.198126015 | -2.335509772 |  | 0.003309922 | 0.046332782 | Down |
| SULT1A4 | 0.198276661 | -2.334413225 |  | 0.043400004 | 0.243278183 | Down |
| C5AR2 | 0.198654162 | -2.331669074 |  | 0.028859214 | 0.191524842 | Down |
| VWCE | 0.199678074 | -2.324252172 |  | 0.000178719 | 0.005136652 | Down |
| LGSN | 0.19970863 | -2.324031417 |  | 0.000338911 | 0.008640225 | Down |
| RNF112 | 0.200066713 | -2.321446946 |  | 0.00240514 | 0.037369889 | Down |
| AGBL2 | 0.200727478 | -2.316689972 |  | 6.30E-09 | 7.26E-07 | Down |
| FAM71F1 | 0.203351343 | -2.297953573 |  | 6.60E-13 | 1.58E-10 | Down |
| AZU1 | 0.204001907 | -2.293345455 |  | 0.011578079 | 0.106860442 | Down |
| TMEM151B | 0.20403859 | -2.293086057 |  | 0.015555214 | 0.130573975 | Down |
| SLC8A2 | 0.204088845 | -2.292730765 |  | 8.30E-08 | 7.01E-06 | Down |
| SRL | 0.204255428 | -2.291553674 |  | 4.09E-05 | 0.001521362 | Down |
| ORM1 | 0.20449658 | -2.289851381 |  | 0.049595924 | 0.260434589 | Down |
| SPEGNB | 0.204524695 | -2.289653043 |  | 0.044368039 | 0.245953619 | Down |
| COL2A1 | 0.205727972 | -2.281190128 |  | 0.03352594 | 0.209648676 | Down |
| EPHX3 | 0.206580452 | -2.275224352 |  | 1.14E-05 | 0.000520209 | Down |
| PXT1 | 0.207040859 | -2.272012583 |  | 0.009734472 | 0.096249672 | Down |
| NANOS3 | 0.209038435 | -2.258159869 |  | 0.001557811 | 0.027512831 | Down |
| SCN4A | 0.209339215 | -2.256085502 |  | 0.023676735 | 0.170073034 | Down |
| CLDN6 | 0.211123707 | -2.243839506 |  | 1.03E-06 | 6.30E-05 | Down |
| CNTD1 | 0.211539644 | -2.241000035 |  | 0.003502763 | 0.047857661 | Down |
| GCKR | 0.211782696 | -2.239343379 |  | 0.00365668 | 0.04945468 | Down |
| PRPH | 0.211840595 | -2.238949017 |  | 0.013229764 | 0.1178595 | Down |
| CASS4 | 0.213371405 | -2.228561251 |  | 7.83E-06 | 0.000385345 | Down |
| EFHB | 0.213559552 | -2.227289664 |  | 0.011168391 | 0.104806786 | Down |
| MUC2 | 0.214731571 | -2.219393776 |  | 0.010111311 | 0.098725382 | Down |
| RAB33A | 0.215534614 | -2.214008513 |  | 0.006249788 | 0.071548775 | Down |
| MYO1G | 0.215777849 | -2.212381322 |  | 2.19E-05 | 0.00089726 | Down |
| FAM71E2 | 0.215846968 | -2.211919269 |  | 4.08E-05 | 0.001521362 | Down |
| ART4 | 0.21608018 | -2.210361346 |  | 0.004074901 | 0.05383253 | Down |
| PSG7 | 0.216687007 | -2.206315444 |  | 0.034839166 | 0.212979584 | Down |
| TNF | 0.217004523 | -2.204202984 |  | 0.031133532 | 0.201488327 | Down |
| CRYBB2 | 0.217055557 | -2.203863739 |  | 6.93E-08 | 6.02E-06 | Down |
| U2AF1 | 0.21755145 | -2.200571465 |  | 0.017100031 | 0.138267522 | Down |
| NPIPB6 | 0.219513744 | -2.18761682 |  | 0.006659012 | 0.074309161 | Down |
| TTBK1 | 0.219690741 | -2.186454029 |  | 0.033846771 | 0.210169911 | Down |
| ATP2A1 | 0.219784636 | -2.185837558 |  | 8.36E-09 | 9.44E-07 | Down |
| FNDC7 | 0.219955952 | -2.184713455 |  | 8.74E-05 | 0.002866337 | Down |
| KRTDAP | 0.220442724 | -2.181524238 |  | 0.018893386 | 0.148379332 | Down |
| SCG2 | 0.220478079 | -2.181292874 |  | 0.019467094 | 0.151002324 | Down |
| ARC | 0.22237939 | -2.168905009 |  | 1.47E-36 | 5.56E-33 | Down |
| GPR132 | 0.222904233 | -2.165504079 |  | 1.51E-07 | 1.15E-05 | Down |
| CUZD1 | 0.224090321 | -2.157847759 |  | 0.000949747 | 0.018903072 | Down |
| PSG4 | 0.224260319 | -2.156753728 |  | 0.002863523 | 0.042111621 | Down |
| BNIPL | 0.224418648 | -2.155735532 |  | 3.18E-06 | 0.000175227 | Down |
| MYH11 | 0.225785932 | -2.146972494 |  | 0.000441097 | 0.010652568 | Down |
| PLCD4 | 0.226415595 | -2.142954766 |  | 0.003594097 | 0.048731438 | Down |
| ADGRV1 | 0.226702049 | -2.141130666 |  | 0.004764909 | 0.060029912 | Down |
| C19orf38 | 0.228003622 | -2.132871352 |  | 0.000316142 | 0.008142133 | Down |
| GOLGA6B | 0.228448366 | -2.130059974 |  | 0.000811188 | 0.016985506 | Down |
| PTPN22 | 0.22872211 | -2.128332258 |  | 0.002222604 | 0.035221523 | Down |
| ACTA1 | 0.228907665 | -2.127162324 |  | 8.60E-06 | 0.000412737 | Down |
| CHRM5 | 0.229032011 | -2.12637884 |  | 0.039300439 | 0.227679384 | Down |
| FITM1 | 0.229375217 | -2.124218575 |  | 0.018181514 | 0.144591333 | Down |
| GCM1 | 0.230120736 | -2.119537103 |  | 0.001364283 | 0.025046439 | Down |
| ASIC4 | 0.23050538 | -2.117127672 |  | 0.004454487 | 0.057250931 | Down |
| CFAP97D1 | 0.23091476 | -2.114567705 |  | 0.00657853 | 0.073842546 | Down |
| AXDND1 | 0.231325209 | -2.112005603 |  | 0.003291948 | 0.046209532 | Down |
| GREM1 | 0.236358207 | -2.080953135 |  | 2.51E-05 | 0.001012111 | Down |
| ATP6V1FNB | 0.238361454 | -2.06877714 |  | 8.67E-08 | 7.20E-06 | Down |
| MYO1A | 0.242754933 | -2.042427479 |  | 0.000660344 | 0.014533151 | Down |
| ADGRF2 | 0.242989329 | -2.041035139 |  | 0.005435787 | 0.065840062 | Down |
| ITGA10 | 0.243035695 | -2.040759873 |  | 3.17E-08 | 3.02E-06 | Down |
| CFAP70 | 0.244108993 | -2.034402649 |  | 1.05E-17 | 5.13E-15 | Down |
| STAC3 | 0.245488717 | -2.02627138 |  | 1.25E-06 | 7.59E-05 | Down |
| PECAM1 | 0.248890778 | -2.00641532 |  | 0.038119996 | 0.222734198 | Down |
| MEFV | 0.248920278 | -2.006244331 |  | 0.00724538 | 0.078183904 | Down |
| ANKRD61 | 0.249304247 | -2.004020637 |  | 8.36E-06 | 0.000403954 | Down |
| MYO1H | 0.250472142 | -1.99727794 |  | 0.026013197 | 0.179002052 | Down |
| FAM209B | 0.250634996 | -1.996340221 |  | 0.003635822 | 0.049253003 | Down |
| WDR87 | 0.251100428 | -1.993663605 |  | 0.013886646 | 0.121915394 | Down |
| TCIM | 0.251605482 | -1.99076474 |  | 0.040725884 | 0.233305767 | Down |
| C11orf86 | 0.251769405 | -1.989825116 |  | 0.04075458 | 0.233381721 | Down |
| HSFX2 | 0.251817005 | -1.989552387 |  | 0.037938847 | 0.222145985 | Down |
| PIWIL3 | 0.251843702 | -1.989399441 |  | 0.045091777 | 0.248215612 | Down |
| ABO | 0.25736484 | -1.958113122 |  | 0.041645264 | 0.237114943 | Down |
| SLC22A1 | 0.257579625 | -1.956909618 |  | 4.87E-06 | 0.000254133 | Down |
| CCDC116 | 0.257819872 | -1.955564626 |  | 0.002918005 | 0.042499422 | Down |
| CYP2C8 | 0.258507188 | -1.951723699 |  | 0.010215368 | 0.099124478 | Down |
| LOC400499 | 0.258543561 | -1.951520721 |  | 1.65E-07 | 1.24E-05 | Down |
| BTBD16 | 0.258589143 | -1.951266393 |  | 0.000627565 | 0.013993401 | Down |
| CCDC74B | 0.25900349 | -1.948956559 |  | 0.000349682 | 0.008840287 | Down |
| AOC3 | 0.259211796 | -1.94779672 |  | 4.73E-20 | 2.98E-17 | Down |
| PADI3 | 0.261727164 | -1.933864428 |  | 6.10E-09 | 7.15E-07 | Down |
| C11orf91 | 0.26247465 | -1.929750003 |  | 0.003152197 | 0.044746396 | Down |
| TMOD4 | 0.262756579 | -1.928201207 |  | 8.29E-06 | 0.000403954 | Down |
| CEL | 0.262876848 | -1.927541007 |  | 2.16E-09 | 2.81E-07 | Down |
| CATSPER3 | 0.26393054 | -1.9217698 |  | 0.008275633 | 0.086046093 | Down |
| ACRBP | 0.266321658 | -1.908758336 |  | 0.002726661 | 0.040612476 | Down |
| RASIP1 | 0.266868226 | -1.905800548 |  | 0.001057449 | 0.020681124 | Down |
| AVIL | 0.267166831 | -1.904187186 |  | 8.62E-10 | 1.28E-07 | Down |
| BCL2L15 | 0.267830095 | -1.900610017 |  | 0.006620619 | 0.07408625 | Down |
| DNAH2 | 0.26915275 | -1.893502928 |  | 0.004873245 | 0.060937738 | Down |
| GDF9 | 0.270695733 | -1.885255947 |  | 0.00010464 | 0.003323427 | Down |
| GDPD2 | 0.271667441 | -1.880086423 |  | 0.029125931 | 0.192702767 | Down |
| C11orf65 | 0.271784438 | -1.879465242 |  | 0.020550185 | 0.156276505 | Down |
| WDR97 | 0.272331291 | -1.876565338 |  | 0.001870698 | 0.0310101 | Down |
| GP6 | 0.276828696 | -1.852934595 |  | 0.005576759 | 0.066828671 | Down |
| TEX29 | 0.277875386 | -1.847490046 |  | 1.10E-05 | 0.000506878 | Down |
| SELPLG | 0.283465282 | -1.818756047 |  | 3.97E-17 | 1.76E-14 | Down |
| TMPRSS9 | 0.284034852 | -1.815860129 |  | 0.018715858 | 0.147673042 | Down |
| CHRM4 | 0.284356872 | -1.814225425 |  | 0.000110394 | 0.003462818 | Down |
| PIFO | 0.284781945 | -1.812070413 |  | 0.042291029 | 0.238922189 | Down |
| C8G | 0.285031377 | -1.810807353 |  | 4.76E-06 | 0.000249702 | Down |
| SEMA3G | 0.285092047 | -1.8105003 |  | 0.001347222 | 0.024959923 | Down |
| KLF1 | 0.285325299 | -1.809320424 |  | 0.044169674 | 0.245589236 | Down |
| CEP295NL | 0.285710052 | -1.807376302 |  | 9.69E-06 | 0.000453489 | Down |
| CLEC18B | 0.285860149 | -1.806618585 |  | 0.021550959 | 0.161131252 | Down |
| CHD5 | 0.285909069 | -1.80637171 |  | 1.44E-12 | 3.20E-10 | Down |
| RPGRIP1 | 0.286012077 | -1.805852028 |  | 0.005670566 | 0.067508166 | Down |
| CPNE9 | 0.286623141 | -1.802773001 |  | 0.005962586 | 0.069769639 | Down |
| MAP7D2 | 0.288392221 | -1.793895842 |  | 0.000902169 | 0.018258361 | Down |
| NWD2 | 0.288400544 | -1.793854208 |  | 0.048003167 | 0.25644454 | Down |
| BAIAP2L2 | 0.290281198 | -1.784476967 |  | 2.07E-05 | 0.000856236 | Down |
| TMEM119 | 0.290835198 | -1.781726216 |  | 0.039366864 | 0.227679384 | Down |
| CXCL8 | 0.291052553 | -1.780648423 |  | 5.24E-10 | 8.09E-08 | Down |
| DNAH12 | 0.291740052 | -1.777244631 |  | 3.35E-05 | 0.001290068 | Down |
| GOLGA6A | 0.291849487 | -1.77670356 |  | 0.012228414 | 0.111166065 | Down |
| DNAH11 | 0.29195374 | -1.776188301 |  | 0.000145584 | 0.004349668 | Down |
| BHLHE41 | 0.294297653 | -1.764652058 |  | 4.39E-09 | 5.35E-07 | Down |
| TGM4 | 0.294331279 | -1.764487226 |  | 0.014375043 | 0.124539771 | Down |
| N4BP2L1 | 0.294400266 | -1.764149122 |  | 9.06E-06 | 0.000428084 | Down |
| CPLX3 | 0.294449387 | -1.763908423 |  | 0.040509186 | 0.232416653 | Down |
| HUS1B | 0.295491903 | -1.758809497 |  | 0.012888213 | 0.115634428 | Down |
| MEIG1 | 0.296633694 | -1.753245614 |  | 0.017289909 | 0.139110617 | Down |
| MEIOC | 0.297413053 | -1.749460126 |  | 0.037968894 | 0.22222754 | Down |
| RND1 | 0.299576569 | -1.739003305 |  | 5.55E-14 | 1.65E-11 | Down |
| USP6 | 0.301166434 | -1.731367109 |  | 0.032780318 | 0.2070062 | Down |
| UCP3 | 0.301561312 | -1.729476742 |  | 1.23E-09 | 1.72E-07 | Down |
| F10 | 0.302676814 | -1.724149932 |  | 0.032235042 | 0.205277743 | Down |
| ZNF546 | 0.303037323 | -1.722432604 |  | 3.92E-12 | 8.12E-10 | Down |
| KCNN3 | 0.303488744 | -1.720285083 |  | 0.000504253 | 0.011892818 | Down |
| CCDC17 | 0.303509321 | -1.72018727 |  | 4.30E-08 | 3.96E-06 | Down |
| SRRM5 | 0.303772662 | -1.718936055 |  | 0.001256168 | 0.023679244 | Down |
| LGALS9 | 0.303898295 | -1.718339516 |  | 1.83E-13 | 5.04E-11 | Down |
| FBXL22 | 0.305046358 | -1.712899589 |  | 0.033762046 | 0.210164777 | Down |
| RAB40A | 0.305636116 | -1.710113061 |  | 0.048857443 | 0.258622838 | Down |
| ACY1 | 0.306090429 | -1.70797016 |  | 0.029143743 | 0.192721916 | Down |
| PERM1 | 0.30619079 | -1.707497208 |  | 0.011065207 | 0.104199771 | Down |
| UTS2B | 0.306261733 | -1.707162977 |  | 0.017189299 | 0.138501689 | Down |
| ASGR1 | 0.308196883 | -1.698075824 |  | 1.60E-05 | 0.000682044 | Down |
| NR4A2 | 0.309155327 | -1.69359623 |  | 7.16E-20 | 4.33E-17 | Down |
| EMC10 | 0.309370061 | -1.692594509 |  | 0.030153555 | 0.197513625 | Down |
| TG | 0.309926717 | -1.69000097 |  | 0.000287348 | 0.007502818 | Down |
| NTRK1 | 0.310425022 | -1.687683242 |  | 0.014374618 | 0.124539771 | Down |
| FOXD4L1 | 0.310466695 | -1.687489582 |  | 0.002703234 | 0.040343039 | Down |
| ARHGAP30 | 0.311112167 | -1.684493277 |  | 8.16E-07 | 5.14E-05 | Down |
| NRBP2 | 0.311465155 | -1.682857322 |  | 3.40E-28 | 5.72E-25 | Down |
| C7orf61 | 0.311715893 | -1.681696382 |  | 0.012868276 | 0.115553466 | Down |
| CKM | 0.313506342 | -1.673433468 |  | 0.032549359 | 0.206150487 | Down |
| ALDH8A1 | 0.31352864 | -1.673330861 |  | 0.005502062 | 0.066320538 | Down |
| SCARF1 | 0.313880854 | -1.671711062 |  | 0.000118573 | 0.003673346 | Down |
| SLC25A34 | 0.314153917 | -1.670456525 |  | 1.55E-07 | 1.18E-05 | Down |
| C2orf16 | 0.316730141 | -1.658673928 |  | 0.034382315 | 0.211541472 | Down |
| TMEM240 | 0.3177419 | -1.654072746 |  | 0.00775746 | 0.082184495 | Down |
| ANKRD34A | 0.318081625 | -1.652531061 |  | 9.28E-16 | 3.19E-13 | Down |
| FOXD4 | 0.319477308 | -1.646214634 |  | 8.02E-06 | 0.00039226 | Down |
| DDR2 | 0.319846816 | -1.644546973 |  | 1.46E-14 | 4.68E-12 | Down |
| NEK10 | 0.320176479 | -1.643060765 |  | 1.06E-35 | 3.20E-32 | Down |
| GPT | 0.32127559 | -1.638116725 |  | 0.031754881 | 0.203287844 | Down |
| APOA1 | 0.321401065 | -1.637553383 |  | 0.012062236 | 0.110252041 | Down |
| POU3F1 | 0.322099005 | -1.634423891 |  | 1.79E-05 | 0.000754422 | Down |
| RAPSN | 0.322376712 | -1.633180565 |  | 0.046907604 | 0.25372063 | Down |
| FOSB | 0.326365179 | -1.615440955 |  | 3.87E-30 | 7.31E-27 | Down |
| SCNN1G | 0.327207077 | -1.611724145 |  | 0.036871787 | 0.219295529 | Down |
| ADAMTS4 | 0.327744309 | -1.609357367 |  | 0.000272519 | 0.007165124 | Down |
| TEX14 | 0.328352075 | -1.606684523 |  | 8.53E-09 | 9.56E-07 | Down |
| MIP | 0.329044664 | -1.603644667 |  | 0.015093304 | 0.128263387 | Down |
| SERINC4 | 0.330511132 | -1.597229229 |  | 0.006416842 | 0.072449453 | Down |
| CLEC18A | 0.331343188 | -1.593601835 |  | 0.049136615 | 0.259025299 | Down |
| SPEM2 | 0.33145141 | -1.593130705 |  | 0.004420962 | 0.057076094 | Down |
| RAD51AP2 | 0.332473721 | -1.58868778 |  | 0.034976771 | 0.213303276 | Down |
| FAM83E | 0.332540277 | -1.588399006 |  | 3.11E-07 | 2.21E-05 | Down |
| RSPH10B | 0.335084812 | -1.577401798 |  | 0.003283978 | 0.046183426 | Down |
| ESPNL | 0.337228029 | -1.568203642 |  | 0.000584607 | 0.013230663 | Down |
| SLC22A13 | 0.338969187 | -1.560773961 |  | 0.00551743 | 0.066411233 | Down |
| GNB3 | 0.33900686 | -1.560613628 |  | 0.000346967 | 0.00878635 | Down |
| ZDHHC11B | 0.33975544 | -1.557431444 |  | 1.17E-09 | 1.67E-07 | Down |
| CNBD2 | 0.340121667 | -1.555877183 |  | 0.001286229 | 0.024006424 | Down |
| ADGRG3 | 0.340329236 | -1.554997004 |  | 3.46E-05 | 0.001323602 | Down |
| CYP2D6 | 0.341974251 | -1.548040395 |  | 2.75E-08 | 2.69E-06 | Down |
| SAP25 | 0.342443204 | -1.546063366 |  | 1.08E-07 | 8.64E-06 | Down |
| CUBN | 0.343257688 | -1.542636062 |  | 0.002546469 | 0.038807978 | Down |
| LOC107986982 | 0.343625455 | -1.541091181 |  | 0.034408022 | 0.211541472 | Down |
| TNNI2 | 0.343763169 | -1.540513114 |  | 0.002032086 | 0.0328146 | Down |
| ADRA2B | 0.343930768 | -1.539809909 |  | 0.002475078 | 0.038181865 | Down |
| NHLH1 | 0.344889683 | -1.53579312 |  | 0.010915402 | 0.103655184 | Down |
| NPIPA7 | 0.345723273 | -1.532310371 |  | 5.82E-06 | 0.000294296 | Down |
| DCAF4L1 | 0.346714203 | -1.528181158 |  | 0.000195789 | 0.00545109 | Down |
| SIK1 | 0.346871946 | -1.527524932 |  | 0.03133623 | 0.201849651 | Down |
| FAXDC2 | 0.346881546 | -1.527485001 |  | 0.027971803 | 0.18767236 | Down |
| SCRT1 | 0.347917771 | -1.523181725 |  | 0.042664424 | 0.240134311 | Down |
| HMCN2 | 0.348082128 | -1.522500354 |  | 0.030317373 | 0.198243099 | Down |
| MUCL3 | 0.349527796 | -1.516520907 |  | 9.59E-09 | 1.06E-06 | Down |
| RCN3 | 0.349727106 | -1.515698478 |  | 0.000459661 | 0.011012922 | Down |
| STEAP4 | 0.351095606 | -1.510064156 |  | 0.003505697 | 0.047857661 | Down |
| ANGPTL6 | 0.3520871 | -1.505995723 |  | 0.012561055 | 0.113575377 | Down |
| RAB40AL | 0.352699226 | -1.503489684 |  | 0.02941371 | 0.194012417 | Down |
| LAG3 | 0.353694794 | -1.49942311 |  | 0.001097388 | 0.021179044 | Down |
| KLLN | 0.354494859 | -1.496163389 |  | 0.001517378 | 0.026987909 | Down |
| IQCN | 0.354502539 | -1.496132135 |  | 3.14E-09 | 3.99E-07 | Down |
| IL20RB | 0.354774078 | -1.495027492 |  | 0.000152889 | 0.004523233 | Down |
| SPDEF | 0.35504501 | -1.493926165 |  | 0.019356595 | 0.150633805 | Down |
| STX19 | 0.355076665 | -1.493797544 |  | 3.18E-05 | 0.001232089 | Down |
| LOC102724474 | 0.356508574 | -1.487991319 |  | 0.000549802 | 0.012605614 | Down |
| TAMALIN | 0.356538042 | -1.487872077 |  | 0.022493983 | 0.165148053 | Down |
| CCDC168 | 0.357119629 | -1.485520662 |  | 0.000527972 | 0.012298743 | Down |
| PATL2 | 0.357564316 | -1.483725328 |  | 0.04915621 | 0.259025299 | Down |
| MUC20 | 0.359541119 | -1.475771322 |  | 4.99E-06 | 0.000259123 | Down |
| ARL13A | 0.360266488 | -1.472863637 |  | 0.006178971 | 0.071281064 | Down |
| PLB1 | 0.361986879 | -1.465990692 |  | 5.21E-10 | 8.09E-08 | Down |
| COL20A1 | 0.36241628 | -1.464280332 |  | 2.70E-07 | 1.94E-05 | Down |
| CHRNA4 | 0.363408379 | -1.46033641 |  | 0.029304233 | 0.193374683 | Down |
| DNAH10 | 0.363541268 | -1.459808951 |  | 0.005856065 | 0.069003893 | Down |
| S1PR4 | 0.363563612 | -1.459720283 |  | 0.041695601 | 0.237153537 | Down |
| SH2D6 | 0.363775989 | -1.458877774 |  | 0.002043713 | 0.032939072 | Down |
| LCN12 | 0.364496853 | -1.456021738 |  | 2.68E-06 | 0.000151734 | Down |
| CDRT1 | 0.365789583 | -1.450914106 |  | 8.95E-26 | 1.04E-22 | Down |
| TPPP | 0.36620103 | -1.449292246 |  | 3.96E-10 | 6.30E-08 | Down |
| ESR2 | 0.367429414 | -1.444460972 |  | 0.001678677 | 0.028740934 | Down |
| ITIH4 | 0.367571853 | -1.443901799 |  | 0.000587156 | 0.013268503 | Down |
| FAM106B | 0.367687522 | -1.443447877 |  | 0.005615298 | 0.067161452 | Down |
| MYB | 0.368982062 | -1.438377414 |  | 0.036925185 | 0.219506656 | Down |
| SBSN | 0.37047788 | -1.432540688 |  | 0.001084483 | 0.021046491 | Down |
| CLCN1 | 0.372533046 | -1.424559687 |  | 0.002394177 | 0.037276172 | Down |
| ANKRD31 | 0.372815017 | -1.423468121 |  | 4.04E-07 | 2.77E-05 | Down |
| RASD1 | 0.37520054 | -1.41426619 |  | 5.21E-13 | 1.27E-10 | Down |
| PSG2 | 0.375803886 | -1.411948109 |  | 0.007671975 | 0.081622039 | Down |
| LSMEM2 | 0.375975952 | -1.411287708 |  | 0.001919316 | 0.031539369 | Down |
| H4-16 | 0.376791918 | -1.408160075 |  | 0.021043075 | 0.158667937 | Down |
| IL6 | 0.382566746 | -1.386216619 |  | 0.000138197 | 0.004178514 | Down |
| CPA5 | 0.383214513 | -1.383775897 |  | 0.00078309 | 0.016511509 | Down |
| OR1F1 | 0.384320409 | -1.379618503 |  | 0.015665881 | 0.131357066 | Down |
| LHX3 | 0.38456642 | -1.378695302 |  | 1.16E-05 | 0.000526688 | Down |
| PROX1 | 0.38485934 | -1.377596835 |  | 0.002408072 | 0.03737704 | Down |
| HOXA2 | 0.389644126 | -1.359771028 |  | 4.03E-09 | 4.96E-07 | Down |
| MYH7B | 0.390152162 | -1.3578912 |  | 0.016296713 | 0.134444519 | Down |
| TCAP | 0.390461977 | -1.356746028 |  | 0.019311337 | 0.150633805 | Down |
| UPK1B | 0.391325373 | -1.353559439 |  | 0.007704789 | 0.081741055 | Down |
| ABCC2 | 0.391624059 | -1.352458697 |  | 2.16E-10 | 3.51E-08 | Down |
| AGER | 0.392331466 | -1.349855047 |  | 8.98E-08 | 7.38E-06 | Down |
| RASL10A | 0.392356057 | -1.349764622 |  | 0.000114917 | 0.003582091 | Down |
| DCST1 | 0.393142201 | -1.346876861 |  | 0.002617064 | 0.039446436 | Down |
| ALPK3 | 0.393646053 | -1.345029082 |  | 3.08E-12 | 6.48E-10 | Down |
| LOC105377310 | 0.394763647 | -1.340938953 |  | 0.024581103 | 0.173571753 | Down |
| NR4A3 | 0.395145127 | -1.339545477 |  | 2.59E-16 | 1.03E-13 | Down |
| SPOCK2 | 0.395532666 | -1.338131248 |  | 1.44E-21 | 1.04E-18 | Down |
| ECM2 | 0.398053469 | -1.328965858 |  | 0.013161558 | 0.117390225 | Down |
| NPIPB11 | 0.399232185 | -1.324700064 |  | 1.80E-07 | 1.34E-05 | Down |
| LDB3 | 0.400189884 | -1.321243395 |  | 0.027790099 | 0.187140634 | Down |
| RELN | 0.401427523 | -1.316788559 |  | 4.21E-05 | 0.00155782 | Down |
| MYH3 | 0.401499608 | -1.316529517 |  | 2.45E-08 | 2.45E-06 | Down |
| RP1 | 0.401655956 | -1.315967825 |  | 0.038480569 | 0.224165444 | Down |
| EFCAB8 | 0.402381874 | -1.313362777 |  | 1.72E-10 | 2.86E-08 | Down |
| DRICH1 | 0.403228953 | -1.310328862 |  | 0.003303385 | 0.046284126 | Down |
| CCDC13 | 0.403230427 | -1.310323589 |  | 0.040812651 | 0.233460671 | Down |
| IFFO1 | 0.403646608 | -1.308835328 |  | 3.78E-07 | 2.62E-05 | Down |
| SNAI1 | 0.404608155 | -1.305402695 |  | 4.41E-27 | 6.67E-24 | Down |
| SYNPO2 | 0.406653291 | -1.298128805 |  | 6.96E-06 | 0.00034858 | Down |
| LPXN | 0.407187831 | -1.296233647 |  | 8.35E-08 | 7.02E-06 | Down |
| FOS | 0.407435246 | -1.295357307 |  | 1.28E-06 | 7.75E-05 | Down |
| CHRNA10 | 0.408207307 | -1.292626088 |  | 6.47E-05 | 0.002232614 | Down |
| H3C1 | 0.408559876 | -1.291380569 |  | 0.03951674 | 0.22828203 | Down |
| LEKR1 | 0.408599118 | -1.291242004 |  | 0.000149184 | 0.0044397 | Down |
| EFNA2 | 0.408695045 | -1.290903342 |  | 1.93E-09 | 2.55E-07 | Down |
| ITGB7 | 0.408705764 | -1.290865505 |  | 0.001881287 | 0.031117388 | Down |
| TF | 0.411352832 | -1.281551719 |  | 0.022757486 | 0.166367349 | Down |
| SEMA4A | 0.414491393 | -1.270585951 |  | 1.01E-10 | 1.78E-08 | Down |
| SHLD3 | 0.414939383 | -1.269027502 |  | 0.00089564 | 0.01822381 | Down |
| PTCH2 | 0.415178501 | -1.268196356 |  | 0.001386601 | 0.025321288 | Down |
| PLA2G4C | 0.415477264 | -1.267158565 |  | 0.000372244 | 0.009286448 | Down |
| PRDM12 | 0.415593125 | -1.266756307 |  | 0.048288626 | 0.25697403 | Down |
| CHRNB2 | 0.416125325 | -1.264910003 |  | 1.25E-05 | 0.000552504 | Down |
| IL2RB | 0.419644068 | -1.252761906 |  | 0.031174007 | 0.201577687 | Down |
| NPIPB2 | 0.421386545 | -1.246783845 |  | 5.02E-05 | 0.00180731 | Down |
| LRGUK | 0.423343733 | -1.240098564 |  | 0.02970108 | 0.195141643 | Down |
| SSUH2 | 0.426315022 | -1.230008204 |  | 0.005319976 | 0.064756353 | Down |
| REELD1 | 0.429376603 | -1.219684516 |  | 0.039196208 | 0.227560782 | Down |
| H2BC7 | 0.430912138 | -1.214534359 |  | 0.010440266 | 0.100853635 | Down |
| C5AR1 | 0.43213234 | -1.210454892 |  | 0.000128513 | 0.003917059 | Down |
| HGFAC | 0.433440922 | -1.206092729 |  | 0.014280264 | 0.124145506 | Down |
| C3orf20 | 0.433765898 | -1.205011459 |  | 5.92E-05 | 0.002075965 | Down |
| UCN | 0.434162859 | -1.203691781 |  | 0.024808613 | 0.174363833 | Down |
| ADHFE1 | 0.436163113 | -1.19706033 |  | 0.020146658 | 0.154272109 | Down |
| C4B | 0.436957963 | -1.1944336 |  | 0.006703606 | 0.074628216 | Down |
| PHKG1 | 0.437118414 | -1.193903942 |  | 0.000363637 | 0.00911685 | Down |
| REC8 | 0.438028104 | -1.190904657 |  | 0.001008848 | 0.019884957 | Down |
| KLK14 | 0.439581468 | -1.185797528 |  | 0.001466816 | 0.026399191 | Down |
| HIC1 | 0.443652275 | -1.172498728 |  | 2.75E-06 | 0.000154509 | Down |
| HEY1 | 0.444083619 | -1.17109674 |  | 0.037722058 | 0.221434203 | Down |
| GADD45B | 0.444251097 | -1.170552755 |  | 2.49E-33 | 6.26E-30 | Down |
| C1orf162 | 0.44484709 | -1.168618581 |  | 0.000109305 | 0.003442658 | Down |
| C1orf54 | 0.445475593 | -1.166581703 |  | 0.022404745 | 0.164871388 | Down |
| FAM186B | 0.445673371 | -1.165941333 |  | 0.027564012 | 0.186198722 | Down |
| PRDM1 | 0.449759726 | -1.152773616 |  | 2.65E-05 | 0.001053563 | Down |
| MAFB | 0.450028482 | -1.151911784 |  | 6.06E-18 | 3.10E-15 | Down |
| POU2F2 | 0.451421571 | -1.147452736 |  | 0.00184122 | 0.030593415 | Down |
| INTS6L | 0.452642899 | -1.143554773 |  | 3.52E-05 | 0.001336827 | Down |
| KIF17 | 0.454100947 | -1.13891505 |  | 4.51E-07 | 3.01E-05 | Down |
| MAK | 0.455122954 | -1.135671745 |  | 0.001470572 | 0.026403927 | Down |
| STARD5 | 0.458545198 | -1.124864151 |  | 3.92E-11 | 7.51E-09 | Down |
| KRT9 | 0.461428366 | -1.115821399 |  | 0.032933061 | 0.207364441 | Down |
| LNP1 | 0.461691155 | -1.115000001 |  | 0.001263719 | 0.023791901 | Down |
| NPIPB13 | 0.462412433 | -1.112747906 |  | 0.000469171 | 0.011205247 | Down |
| ROPN1L | 0.462906527 | -1.111207191 |  | 0.010978968 | 0.103932402 | Down |
| GHRL | 0.463526271 | -1.109276988 |  | 0.031297982 | 0.201849651 | Down |
| CCDC62 | 0.465261009 | -1.103887806 |  | 0.030110148 | 0.197314788 | Down |
| CD101 | 0.467348869 | -1.097428191 |  | 0.001130578 | 0.021745643 | Down |
| MICALCL | 0.467913492 | -1.095686267 |  | 0.000176665 | 0.005087288 | Down |
| EGR4 | 0.469088172 | -1.092068971 |  | 1.59E-05 | 0.000682044 | Down |
| TMEM81 | 0.471426804 | -1.084894306 |  | 0.000198779 | 0.005503929 | Down |
| MEX3B | 0.471514886 | -1.084624777 |  | 0.011365639 | 0.105541312 | Down |
| TENT5C | 0.471928057 | -1.083361151 |  | 2.00E-08 | 2.04E-06 | Down |
| DACT3 | 0.472728932 | -1.080914933 |  | 0.047406388 | 0.254596725 | Down |
| PRR4 | 0.47370377 | -1.077942942 |  | 0.037116014 | 0.219788447 | Down |
| PZP | 0.474018515 | -1.076984684 |  | 0.037368921 | 0.220680994 | Down |
| ESAM | 0.475191455 | -1.073419201 |  | 0.00328973 | 0.046209532 | Down |
| ALB | 0.475753531 | -1.07171373 |  | 0.001392826 | 0.025339034 | Down |
| IL4I1 | 0.476690708 | -1.068874591 |  | 0.024104794 | 0.171167816 | Down |
| HAL | 0.476776226 | -1.068615797 |  | 0.035584248 | 0.215597222 | Down |
| NR1D1 | 0.477667614 | -1.065921029 |  | 2.32E-13 | 6.25E-11 | Down |
| CXCL3 | 0.481204153 | -1.055279001 |  | 0.034317296 | 0.211541472 | Down |
| FBXO24 | 0.481914116 | -1.053152033 |  | 0.033649624 | 0.210003075 | Down |
| LOC105379752 | 0.482010894 | -1.052862341 |  | 6.70E-05 | 0.002307161 | Down |
| OVGP1 | 0.482198588 | -1.052300669 |  | 0.001935841 | 0.031707522 | Down |
| C2 | 0.482724194 | -1.05072896 |  | 0.002820326 | 0.041557202 | Down |
| ZNF547 | 0.483842939 | -1.047389288 |  | 0.000928313 | 0.018563811 | Down |
| ARID3C | 0.483898163 | -1.047224634 |  | 0.04398959 | 0.245219255 | Down |
| H3C8 | 0.48610011 | -1.040674634 |  | 0.00568001 | 0.067508166 | Down |
| CBLN3 | 0.4864369 | -1.039675423 |  | 0.01056219 | 0.101448023 | Down |
| AKAP3 | 0.487576648 | -1.036299064 |  | 0.004893144 | 0.061085512 | Down |
| UBAP1L | 0.489850091 | -1.029587786 |  | 0.000175175 | 0.005073364 | Down |
| TNFSF4 | 0.490769424 | -1.026882727 |  | 0.003111463 | 0.044460395 | Down |
| DNAJC28 | 0.491724243 | -1.024078611 |  | 0.012926133 | 0.115896164 | Down |
| B3GALT4 | 0.493747256 | -1.018155365 |  | 0.000654517 | 0.014466349 | Down |
| SARM1 | 0.493894265 | -1.017725879 |  | 0.002309339 | 0.036201575 | Down |
| ATP1A2 | 0.4942084 | -1.016808561 |  | 0.002556125 | 0.038876752 | Down |
| CXCL2 | 0.494707106 | -1.015353471 |  | 0.037637719 | 0.221282436 | Down |
| NLGN3 | 0.494889634 | -1.014821273 |  | 0.0255443 | 0.177454886 | Down |
| EGR2 | 0.495067465 | -1.014302953 |  | 0.008601764 | 0.088033823 | Down |
| KLHL31 | 0.495424183 | -1.013263804 |  | 0.008294246 | 0.086062055 | Down |
| C6orf226 | 0.495735668 | -1.01235703 |  | 0.000200674 | 0.00554623 | Down |
| PTX3 | 0.496231666 | -1.010914294 |  | 0.002229204 | 0.035289124 | Down |
| RAD9B | 0.496638195 | -1.009732876 |  | 0.002818632 | 0.041557202 | Down |
| NGFR | 0.49725905 | -1.007930468 |  | 0.002087373 | 0.033528182 | Down |
| H2AC17 | 0.497367073 | -1.007617095 |  | 0.010497363 | 0.101082255 | Down |
| PLIN5 | 0.498849483 | -1.003323515 |  | 0.031674826 | 0.203165048 | Down |
| PCDHB10 | 2.088397416 | 1.062396279 |  | 0.032734842 | 0.206875142 | Up |
| TRANK1 | 2.412794916 | 1.270705294 |  | 0.004273074 | 0.055641974 | Up |
| ISM1 | 2.6195948 | 1.389343672 |  | 0.041627671 | 0.237114943 | Up |
| MFAP4 | 2.813478565 | 1.492354972 |  | 0.023857816 | 0.170213529 | Up |
| KIRREL2 | 3.265998897 | 1.707524304 |  | 0.012273787 | 0.111444515 | Up |
| CSRNP3 | 3.484416492 | 1.80091708 |  | 0.024250316 | 0.171958854 | Up |
| PRODH | 3.734246857 | 1.900817302 |  | 0.032813744 | 0.207050619 | Up |
| C15orf65 | 4.113708932 | 2.040439719 |  | 0.034884502 | 0.213170537 | Up |
| GOLGA8O | 5.339009767 | 2.416572188 |  | 0.029079704 | 0.192481161 | Up |
| PLA2G4B | 5.634511215 | 2.494290464 |  | 0.031299238 | 0.201849651 | Up |
| PPP2R3B-2 | 7.495968709 | 2.906114931 |  | 0.009149157 | 0.092211299 | Up |
| MATK | 8.655325924 | 3.113588147 |  | 0.028298669 | 0.189300567 | Up |
| DCN | 12.53449066 | 3.647831468 |  | 0.032288996 | 0.205534752 | Up |
| U2AF1L5 | 17.47977227 | 4.127614484 |  | 0.011281672 | 0.10517269 | Up |
|  |  |  |  |  |  |  |

Table S4. Results of KEGG analysis on DE-genes

| **ID** | **Term** | **P-value** | **q-value** | **Enrichment_score** |
| --- | --- | --- | --- | --- |
| hsa00591 | Linoleic acid metabolism | 8.85E-06 | 0.002318 | 7.214854111 |
| hsa04610 | Complement and coagulation cascades | 1.78E-05 | 0.002335 | 4 |
| hsa05150 | Staphylococcus aureus infection | 5.97E-05 | 0.005216 | 3.578947368 |
| hsa04080 | Neuroactive ligand-receptor interaction | 0.000247 | 0.016181 | 2.070832393 |
| hsa04060 | Cytokine-cytokine receptor interaction | 0.000369 | 0.019326 | 2.127770535 |
| hsa05322 | Systemic lupus erythematosus | 0.000668 | 0.029168 | 2.692307692 |
| hsa04974 | Protein digestion and absorption | 0.001844 | 0.063021 | 2.793129201 |
| hsa00590 | Arachidonic acid metabolism | 0.002097 | 0.063021 | 3.43001261 |
| hsa00592 | alpha-Linolenic acid metabolism | 0.002235 | 0.063021 | 5.230769231 |
| hsa00565 | Ether lipid metabolism | 0.002405 | 0.063021 | 3.736263736 |
| hsa00360 | Phenylalanine metabolism | 0.003359 | 0.080006 | 6.153846154 |
| hsa04744 | Phototransduction | 0.003764 | 0.08218 | 4.67032967 |
| hsa04726 | Serotonergic synapse | 0.004106 | 0.082761 | 2.523616734 |
| hsa04061 | Viral protein interaction with cytokine and cytokine receptor | 0.004738 | 0.085259 | 2.615384615 |
| hsa04975 | Fat digestion and absorption | 0.004881 | 0.085259 | 3.736263736 |
| hsa05202 | Transcriptional misregulation in cancer | 0.006664 | 0.109122 | 2.043269231 |
| hsa05410 | Hypertrophic cardiomyopathy | 0.007228 | 0.111402 | 2.615384615 |
| hsa04657 | IL-17 signaling pathway | 0.009539 | 0.138839 | 2.504091653 |
| hsa04977 | Vitamin digestion and absorption | 0.012154 | 0.167594 | 4.358974359 |
| hsa04972 | Pancreatic secretion | 0.014869 | 0.194779 | 2.330540746 |
| hsa05146 | Amoebiasis | 0.01578 | 0.196872 | 2.307692308 |
| hsa05130 | Pathogenic Escherichia coli infection | 0.018627 | 0.221829 | 1.858648965 |
| hsa00400 | Phenylalanine, tyrosine and tryptophan biosynthesis | 0.019737 | 0.224826 | 8.717948718 |
| hsa04978 | Mineral absorption | 0.024476 | 0.258801 | 2.659713168 |
| hsa05323 | Rheumatoid arthritis | 0.025476 | 0.258801 | 2.249793218 |
| hsa04350 | TGF-beta signaling pathway | 0.026958 | 0.258801 | 2.225859247 |
| hsa05412 | Arrhythmogenic right ventricular cardiomyopathy | 0.027293 | 0.258801 | 2.377622378 |
| hsa02010 | ABC transporters | 0.027658 | 0.258801 | 2.905982906 |
| hsa05414 | Dilated cardiomyopathy | 0.030101 | 0.271948 | 2.179487179 |
| hsa04973 | Carbohydrate digestion and absorption | 0.032664 | 0.285261 | 2.782324059 |
| hsa00270 | Cysteine and methionine metabolism | 0.04117 | 0.326864 | 2.615384615 |
| hsa04979 | Cholesterol metabolism | 0.04117 | 0.326864 | 2.615384615 |
| hsa05144 | Malaria | 0.04117 | 0.326864 | 2.615384615 |
| hsa00500 | Starch and sucrose metabolism | 0.043225 | 0.328309 | 2.989010989 |
| hsa00010 | Glycolysis / Gluconeogenesis | 0.044862 | 0.328309 | 2.307692308 |
| hsa04064 | NF-kappa B signaling pathway | 0.045175 | 0.328309 | 2.01183432 |
| hsa00350 | Tyrosine metabolism | 0.04722 | 0.328309 | 2.905982906 |
| hsa04611 | Platelet activation | 0.047617 | 0.328309 | 1.898263027 |

Table S5. LLNLR-299G3.1 directly bound targets obtained by ChIRP-seq

| **PeakID (cmd=annotatePeaks.pl CHIRP_peaks.bed hg38)** | **Peak Score** | **Annotation** | **Gene Name** | **Gene Type** | |
| --- | --- | --- | --- | --- | --- |
| MACS_peak_8252 | 3181.34 | intron (NM_001846, intron 40 of 47) | COL4A2-AS1 | ncRNA |  |
| MACS_peak_22540 | 3164.62 | non-coding (NR_003286, exon 1 of 1) | RNA5-8SN3 | rRNA |  |
| MACS_peak_11490 | 3106.22 | intron (NM_001190158, intron 2 of 13) | CES5A | protein-coding | |
| MACS_peak_5792 | 3100 | Intergenic | LINC01488 | ncRNA |  |
| MACS_peak_21265 | 3100 | Intergenic | CCDC188 | protein-coding | |
| MACS_peak_23906 | 3100 | intron (NM_001291979, intron 3 of 16) | NOP14 | protein-coding | |
| MACS_peak_22539 | 3100 | non-coding (NR_046235, exon 1 of 1).2 | RNA45SN5 | rRNA |  |
| MACS_peak_24486 | 3100 | intron (NM_018140, intron 11 of 11) | CEP72 | protein-coding | |
| MACS_peak_14343 | 3100 | Intergenic | RPL23AP87 | pseudo |  |
| MACS_peak_22863 | 3100 | Intergenic | LOC101927829 | ncRNA |  |
| MACS_peak_24414 | 3100 | intron (NM_145265, intron 2 of 2) | CCDC127 | protein-coding | |
| MACS_peak_14740 | 3100 | intron (NM_014963, intron 1 of 31) | SBNO2 | protein-coding | |
| MACS_peak_20366 | 3100 | Intergenic | NPBWR2 | protein-coding | |
| MACS_peak_21038 | 3100 | intron (NM_001136004, intron 17 of 21) | MICAL3 | protein-coding | |
| MACS_peak_20554 | 3100 | Intergenic | SCAF4 | protein-coding | |
| MACS_peak_31984 | 3100 | Intergenic | LOC100505874 | ncRNA |  |
| MACS_peak_8408 | 3100 | Intergenic | TFDP1 | protein-coding | |
| MACS_peak_24912 | 3100 | intron (NM_007118, intron 31 of 56) | SNORD170 | snoRNA |  |
| MACS_peak_24490 | 3100 | intron (NM_007030, intron 2 of 3) | TPPP | protein-coding | |
| MACS_peak_31974 | 3100 | non-coding (NR_046235, exon 1 of 1).3 | RNA28SN5 | rRNA |  |
| MACS_peak_336 | 3100 | intron (NM_001242672, intron 5 of 7) | MMEL1 | protein-coding | |
| MACS_peak_31980 | 3100 | Intergenic | RNA5-8SN4 | rRNA |  |
| MACS_peak_24589 | 3100 | intron (NM_198253, intron 2 of 15) | TERT | protein-coding | |
| MACS_peak_14260 | 3100 | intron (NM_001367828, intron 13 of 16) | FASN | protein-coding | |
| MACS_peak_447 | 3100 | intron (NM_001409, intron 3 of 36) | MIR551A | ncRNA |  |
| MACS_peak_338 | 3100 | intron (NM_001242672, intron 5 of 7) | TTC34 | protein-coding | |
| MACS_peak_13635 | 3100 | intron (NM_002737, intron 16 of 16) | MIR634 | ncRNA |  |
| MACS_peak_26958 | 3100 | Intergenic | UNCX | protein-coding | |
| MACS_peak_31983 | 3100 | Intergenic | LOC100505874 | ncRNA |  |
| MACS_peak_484 | 3100 | Intergenic | LINC01345 | ncRNA |  |
| MACS_peak_20474 | 3100 | promoter-TSS (NR_145821).3 | RNA5-8SN1 | rRNA |  |
| MACS_peak_29279 | 3100 | intron (NM_130843, intron 16 of 21) | MIR153-2 | ncRNA |  |
| MACS_peak_18383 | 3100 | intron (NM_030923, intron 2 of 7) | TMEM163 | protein-coding | |
| MACS_peak_10570 | 3100 | intron (NR_147885, intron 10 of 11) | PRR25 | protein-coding | |
| MACS_peak_32015 | 3100 | NA |  |  |  |
| MACS_peak_26947 | 3100 | Intergenic | UNCX | protein-coding | |
| MACS_peak_22548 | 3100 | Intergenic | RNA5-8SN4 | rRNA |  |
| MACS_peak_20462 | 3100 | TTS (NR_003286).3 | RNA5-8SN2 | rRNA |  |
| MACS_peak_28670 | 3100 | exon (NM_005960, exon 2 of 12) | MUC3A | protein-coding | |
| MACS_peak_31972 | 3100 | TTS (NR_106782).6 | RNA45SN1 | rRNA |  |
| MACS_peak_15763 | 3100 | Intergenic | C19orf12 | protein-coding | |
| MACS_peak_7785 | 3100 | Intergenic | FBRSL1 | protein-coding | |
| MACS_peak_339 | 3100 | intron (NM_001242672, intron 5 of 7) | TTC34 | protein-coding | |
| MACS_peak_23733 | 3100 | Intergenic | LINC02012 | ncRNA |  |
| MACS_peak_31982 | 3100 | Intergenic | LOC100505874 | ncRNA |  |
| MACS_peak_20481 | 3100 | non-coding (NR_046235, exon 1 of 1) | RNA28SN5 | rRNA |  |
| MACS_peak_22541 | 3100 | non-coding (NR_046235, exon 1 of 1).2 | RNA28SN1 | rRNA |  |
| MACS_peak_15417 | 3100 | intron (NR_033864, intron 1 of 9) | CYP4F24P | pseudo |  |
| MACS_peak_31701 | 3100 | Intergenic | LHX3 | protein-coding | |
| MACS_peak_20759 | 3100 | Intergenic | LINC00322 | ncRNA |  |
| MACS_peak_23984 | 3100 | intron (NM_001206994, intron 2 of 9) | PPP2R2C | protein-coding | |
| MACS_peak_15983 | 3053.24 | Intergenic | LOC100134317 | ncRNA |  |
| MACS_peak_20463 | 3015.94 | non-coding (NR_146144, exon 1 of 1) | RNA28SN2 | rRNA |  |
| MACS_peak_31950 | 2964.75 | NA |  |  |  |
| MACS_peak_27049 | 2935.32 | intron (NM_001013837, intron 16 of 18) | MAD1L1 | protein-coding | |
| MACS_peak_29305 | 2922.36 | intron (NM_130843, intron 6 of 21) | LOC100506585 | ncRNA |  |
| MACS_peak_31979 | 2918.31 | non-coding (NR_003286, exon 1 of 1).4 | RNA5-8SN1 | rRNA |  |
| MACS_peak_20480 | 2804.94 | TTS (NR_145820).3 | RNA5-8SN5 | rRNA |  |
| MACS_peak_20475 | 2789.89 | non-coding (NR_146151, exon 1 of 1) | RNA28SN3 | rRNA |  |
| MACS_peak_17865 | 2763.98 | intron (NM_001130985, intron 29 of 54) | DYSF | protein-coding | |
| MACS_peak_18829 | 2699.19 | intron (NM_005301, intron 1 of 1) | GPR35 | protein-coding | |
| MACS_peak_20461 | 2694.22 | non-coding (NR_146144, exon 1 of 1) | RNA45SN2 | rRNA |  |
| MACS_peak_32013 | 2693.62 | NA |  |  |  |
| MACS_peak_26898 | 2673.41 | intron (NM_006869, intron 2 of 10) | ADAP1 | protein-coding | |
| MACS_peak_31973 | 2671.14 | non-coding (NR_046235, exon 1 of 1).3 | RNA5-8SN1 | rRNA |  |
| MACS_peak_6602 | 2570.08 | intron (NM_006675, intron 2 of 8) | TSPAN9 | protein-coding | |
| MACS_peak_20473 | 2558.56 | promoter-TSS (NR_146151) | RNA45SN3 | rRNA |  |
| MACS_peak_5021 | 2550.97 | intron (NM_001200049, intron 17 of 57) | CFAP46 | protein-coding | |
| MACS_peak_4771 | 2530.04 | intron (NM_001001936, intron 10 of 18) | VWA2 | protein-coding | |
| MACS_peak_4950 | 2530.01 | Intergenic | LINC01164 | ncRNA |  |
| MACS_peak_31978 | 2483.8 | Intergenic | MIR6724-3 | ncRNA |  |
| MACS_peak_10744 | 2459.78 | TTS (NM_001694) | AMDHD2 | protein-coding | |
| MACS_peak_5065 | 2450 | Intergenic | SCART1 | pseudo |  |
| MACS_peak_9266 | 2423.43 | Intergenic | ZBTB42 | protein-coding | |
| MACS_peak_31951 | 2420.06 | NA |  |  |  |
| MACS_peak_19441 | 2417.64 | Intergenic | SLC2A10 | protein-coding | |
| MACS_peak_15982 | 2408.95 | Intergenic | LOC100134317 | ncRNA |  |
| MACS_peak_15159 | 2364.86 | intron (NM_012335, intron 14 of 27) | ZNF414 | protein-coding | |
| MACS_peak_20514 | 2359.62 | intron (NM_001187, intron 3 of 4) | BAGE2 | protein-coding | |
| MACS_peak_20961 | 2341.47 | Intergenic | LOC102723769 | ncRNA |  |
| MACS_peak_20479 | 2324.14 | non-coding (NR_046235, exon 1 of 1) | RNA45SN1 | rRNA |  |
| MACS_peak_20194 | 2272.34 | intron (NR_033370, intron 1 of 4) | LINC01056 | ncRNA |  |
| MACS_peak_3965 | 2268.86 | Intergenic | GDF2 | protein-coding | |
| MACS_peak_24516 | 2231.42 | Intergenic | ZDHHC11 | protein-coding | |
| MACS_peak_7662 | 2229.72 | intron (NM_004764, intron 1 of 20) | PIWIL1 | protein-coding | |
| MACS_peak_3563 | 2227.9 | intron (NM_014974, intron 31 of 36) | ZMYND11 | protein-coding | |
| MACS_peak_11969 | 2222.37 | intron (NM_001351938, intron 4 of 10) | KLHDC4 | protein-coding | |
| MACS_peak_20469 | 2205.44 | non-coding (NR_003287, exon 2 of 3) | RNA5-8SN4 | rRNA |  |
| MACS_peak_5858 | 2204 | intron (NM_005231, intron 1 of 17) | CTTN | protein-coding | |
| MACS_peak_24644 | 2198.49 | Intergenic | MRPL36 | protein-coding | |
| MACS_peak_19826 | 2180.29 | Intergenic | ANKRD60 | protein-coding | |
| MACS_peak_29375 | 2162.16 | intron (NM_001346810, intron 2 of 14) | LOC105377777 | ncRNA |  |
| MACS_peak_2889 | 2157.65 | exon (NM_001127709, exon 5 of 11) | PRG4 | protein-coding | |
| MACS_peak_20322 | 2098.38 | intron (NR_110081, intron 1 of 2) | ZBTB46-AS1 | ncRNA |  |
| MACS_peak_26764 | 2079.01 | Intergenic | LOC102724511 | ncRNA |  |
| MACS_peak_24659 | 2078.35 | Intergenic | CTD-2194D22.4 | ncRNA |  |
| MACS_peak_19664 | 2075.81 | Intergenic | LINC01524 | ncRNA |  |
| MACS_peak_373 | 2023.62 | Intergenic | ACTRT2 | protein-coding | |
| MACS_peak_3360 | 2009.44 | TTS (NR_023363).3 | RNA5S11 | rRNA |  |
| MACS_peak_24408 | 1967.21 | intron (NM_052909, intron 18 of 19) | LRRC14B | protein-coding | |
| MACS_peak_30238 | 1958.19 | intron (NM_015117, intron 4 of 11) | SNORD149 | snoRNA |  |
| MACS_peak_3364 | 1949.08 | Intergenic | RHOU | protein-coding | |
| MACS_peak_4417 | 1910.14 | intron (NM_001271519, intron 1 of 28) | KCNMA1 | protein-coding | |
| MACS_peak_20764 | 1902.14 | Intergenic | LINC00322 | ncRNA |  |
| MACS_peak_444 | 1876.78 | intron (NM_001409, intron 4 of 36) | MIR551A | ncRNA |  |
| MACS_peak_26785 | 1874.75 | Intergenic | LOC102723672 | ncRNA |  |
| MACS_peak_17912 | 1866.8 | intron (NM_133478, intron 4 of 30) | SLC4A5 | protein-coding | |
| MACS_peak_20071 | 1863.33 | intron (NM_005560, intron 18 of 79) | MIR4758 | ncRNA |  |
| MACS_peak_20970 | 1856.64 | Intergenic | LOC102723769 | ncRNA |  |
| MACS_peak_1040 | 1842.2 | intron (NR_137287, intron 27 of 29) | ARHGEF10L | protein-coding | |
| MACS_peak_26906 | 1834.96 | intron (NM_001134395, intron 3 of 4) | CYP2W1 | protein-coding | |
| MACS_peak_31584 | 1801.2 | intron (NM_001278074, intron 4 of 65) | COL5A1-AS1 | ncRNA |  |
| MACS_peak_24633 | 1798.33 | Intergenic | LOC728613 | pseudo |  |
| MACS_peak_1337 | 1773.43 | Intergenic | LINC01648 | ncRNA |  |
| MACS_peak_26758 | 1758.92 | Intergenic | LOC100131532 | ncRNA |  |
| MACS_peak_28034 | 1734.35 | Intergenic | LOC650226 | pseudo |  |
| MACS_peak_20466 | 1725.91 | TTS (NR_106782).2 | MIR6724-2 | ncRNA |  |
| MACS_peak_24425 | 1715.96 | intron (NM_001267558, intron 5 of 6) | AHRR | protein-coding | |
| MACS_peak_29768 | 1695.28 | promoter-TSS (NR_003594).9 | REXO1L2P | pseudo |  |
| MACS_peak_7522 | 1672.26 | Intergenic | CDK2AP1 | protein-coding | |
| MACS_peak_3550 | 1656.1 | intron (NM_001085474, intron 5 of 6) | LYPD8 | protein-coding | |
| MACS_peak_22321 | 1654.59 | intron (NM_001104595, intron 5 of 9) | FAM118A | protein-coding | |
| MACS_peak_20375 | 1650.1 | intron (NM_001104925, intron 5 of 5) | PCMTD2 | protein-coding | |
| MACS_peak_4860 | 1632.1 | intron (NM_022126, intron 6 of 6) | FAM53B-AS1 | ncRNA |  |
| MACS_peak_31877 | 1628.04 | intron (NR_104598, intron 4 of 10) | EXD3 | protein-coding | |
| MACS_peak_20465 | 1600.98 | intron (NR_003287, intron 1 of 2) | MIR6724-3 | ncRNA |  |
| MACS_peak_25756 | 1593.11 | Intergenic | LINC01622 | ncRNA |  |
| MACS_peak_16979 | 1592.69 | intron (NM_032701, intron 1 of 8) | KMT5C | protein-coding | |
| MACS_peak_8294 | 1592.59 | Intergenic | LINC02337 | ncRNA |  |
| MACS_peak_12913 | 1592.26 | exon (NM_001291462, exon 14 of 14).7 | TBC1D3E | protein-coding | |
| MACS_peak_7739 | 1587.5 | Intergenic | MMP17 | protein-coding | |
| MACS_peak_20243 | 1564.89 | Intergenic | CHRNA4 | protein-coding | |
| MACS_peak_12148 | 1554.03 | intron (NM_153636, intron 9 of 14) | CPNE7 | protein-coding | |
| MACS_peak_1579 | 1552.36 | intron (NM_024503, intron 5 of 8) | EDN2 | protein-coding | |
| MACS_peak_29754 | 1549.87 | non-coding (NR_003594, exon 1 of 1).4 | REXO1L2P | pseudo |  |
| MACS_peak_927 | 1543.75 | promoter-TSS (NM_015164) | PLEKHM2 | protein-coding | |
| MACS_peak_8446 | 1524.11 | intron (NM_001320821, intron 6 of 25).2 | RASA3 | protein-coding | |
| MACS_peak_14065 | 1511.57 | intron (NM_020954, intron 15 of 16) | RNF213 | protein-coding | |
| MACS_peak_20468 | 1501.12 | promoter-TSS (NR_145821).2 | RNA5-8SN1 | rRNA |  |
| MACS_peak_5076 | 1500.14 | Intergenic | FRG2B | protein-coding | |
| MACS_peak_18877 | 1489.7 | intron (NM_015963, intron 2 of 5) | THAP4 | protein-coding | |
| MACS_peak_16355 | 1471.66 | TTS (NM_001130852) | BCAM | protein-coding | |
| MACS_peak_29257 | 1470.77 | intron (NR_026865, intron 2 of 3) | LINC01006 | ncRNA |  |
| MACS_peak_29912 | 1465.54 | Intergenic | LINC00824 | ncRNA |  |
| MACS_peak_8267 | 1461.84 | promoter-TSS (NM_198219) | ING1 | protein-coding | |
| MACS_peak_29386 | 1458.01 | intron (NM_001346810, intron 3 of 14) | DLGAP2-AS1 | ncRNA |  |
| MACS_peak_24380 | 1451.86 | Intergenic | DUX4 | protein-coding | |
| MACS_peak_22621 | 1445.37 | promoter-TSS (NM_001292043) | THUMPD3-AS1 | ncRNA |  |
| MACS_peak_8406 | 1436.85 | Intergenic | TFDP1 | protein-coding | |
| MACS_peak_1864 | 1424.31 | Intergenic | MGC34796 | pseudo |  |
| MACS_peak_5038 | 1421.57 | intron (NM_001083909, intron 5 of 6) | ADGRA1 | protein-coding | |
| MACS_peak_27835 | 1414.98 | TTS (NM_033054) | MYO1G | protein-coding | |
| MACS_peak_7820 | 1414.39 | intron (NM_001172557, intron 13 of 15) | ANKLE2 | protein-coding | |
| MACS_peak_17797 | 1400.16 | Intergenic | MIR4433B | ncRNA |  |
| MACS_peak_25991 | 1394.49 | promoter-TSS (NR_149095) | FLOT1 | protein-coding | |
| MACS_peak_20055 | 1392.45 | intron (NM_001278649, intron 11 of 12) | ADRM1 | protein-coding | |
| MACS_peak_4888 | 1386.77 | intron (NM_001290223, intron 1 of 51) | DOCK1 | protein-coding | |
| MACS_peak_8361 | 1383.22 | intron (NM_001366646, intron 2 of 27) | MCF2L | protein-coding | |
| MACS_peak_363 | 1377.8 | Intergenic | TTC34 | protein-coding | |
| MACS_peak_29374 | 1376.37 | intron (NM_001346810, intron 2 of 14) | LOC105377777 | ncRNA |  |
| MACS_peak_7693 | 1373.72 | intron (NM_198827, intron 13 of 24) | ADGRD1-AS1 | ncRNA |  |
| MACS_peak_29773 | 1373.48 | non-coding (NR_003594, exon 1 of 1).10 | REXO1L2P | pseudo |  |
| MACS_peak_1580 | 1371.03 | intron (NM_024503, intron 5 of 8) | EDN2 | protein-coding | |
| MACS_peak_20227 | 1364.91 | promoter-TSS (NM_020882) | COL20A1 | protein-coding | |
| MACS_peak_29752 | 1356.5 | Intergenic | REXO1L2P | pseudo |  |
| MACS_peak_10793 | 1347.11 | promoter-TSS (NM_024339) | THOC6 | protein-coding | |
| MACS_peak_773 | 1347.02 | intron (NM_001079843, intron 2 of 20) | CASZ1 | protein-coding | |
| MACS_peak_19243 | 1345.35 | intron (NR_109939, intron 2 of 4) | DLGAP4-AS1 | ncRNA |  |
| MACS_peak_21492 | 1339.09 | intron (NM_014634, intron 1 of 7) | PPM1F | protein-coding | |
| MACS_peak_7800 | 1326.39 | intron (NM_001142641, intron 2 of 16) | FBRSL1 | protein-coding | |
| MACS_peak_20476 | 1324.96 | non-coding (NR_038958, exon 4 of 7).2 | RNA28SN3 | rRNA |  |
| MACS_peak_21434 | 1322.74 | intron (NM_015094, intron 1 of 2) | HIC2 | protein-coding | |
| MACS_peak_21070 | 1320.06 | Intergenic | PI4KAP1 | pseudo |  |
| MACS_peak_26850 | 1314.29 | intron (NM_001164760, intron 10 of 10) | LOC101927000 | ncRNA |  |
| MACS_peak_24548 | 1313.87 | intron (NM_006598, intron 17 of 23).2 | MIR4635 | ncRNA |  |
| MACS_peak_29755 | 1306.54 | non-coding (NR_003594, exon 1 of 1).5 | REXO1L2P | pseudo |  |
| MACS_peak_669 | 1303.05 | promoter-TSS (NM_007262) | PARK7 | protein-coding | |
| MACS_peak_20477 | 1302.97 | Intergenic | MIR6724-1 | ncRNA |  |
| MACS_peak_24989 | 1299.79 | intron (NM_004061, intron 4 of 14) | PMCHL1 | pseudo |  |
| MACS_peak_5513 | 1295.06 | TTS (NM_013265) | TM7SF2 | protein-coding | |
| MACS_peak_9200 | 1295 | Intergenic | LINC02691 | ncRNA |  |
| MACS_peak_29766 | 1290.33 | non-coding (NR_003594, exon 1 of 1).8 | REXO1L2P | pseudo |  |
| MACS_peak_17534 | 1284.5 | intron (NR_027099, intron 10 of 10) | LINC00486 | ncRNA |  |
| MACS_peak_16701 | 1282.24 | intron (NM_006270, intron 1 of 5) | RRAS | protein-coding | |
| MACS_peak_5024 | 1276.33 | intron (NR_120627, intron 1 of 1) | LINC01166 | ncRNA |  |
| MACS_peak_15980 | 1275.02 | Intergenic | ZNF565 | protein-coding | |
| MACS_peak_2376 | 1268.6 | Intergenic | LOR | protein-coding | |
| MACS_peak_31457 | 1268.46 | Intergenic | ABO | protein-coding | |
| MACS_peak_24597 | 1262.26 | Intergenic | LINC01511 | ncRNA |  |
| MACS_peak_20293 | 1250.93 | promoter-TSS (NM_012384) | MHENCR | ncRNA |  |
| MACS_peak_8457 | 1248.67 | intron (NM_001320821, intron 1 of 25).2 | RASA3 | protein-coding | |
| MACS_peak_14481 | 1246.29 | Intergenic | ANKRD62 | protein-coding | |
| MACS_peak_20082 | 1242.99 | intron (NM_001024, intron 1 of 5) | RPS21 | protein-coding | |
| MACS_peak_30271 | 1241.72 | intron (NM_139021, intron 13 of 13) | MAPK15 | protein-coding | |
| MACS_peak_28744 | 1235.02 | 3' UTR (NM_001367767, exon 21 of 21) | POLR2J | protein-coding | |
| MACS_peak_30098 | 1233.82 | Intergenic | MIR4472-1 | ncRNA |  |
| MACS_peak_7810 | 1232.41 | intron (NM_001142641, intron 5 of 16) | MIR6763 | ncRNA |  |
| MACS_peak_12908 | 1231.44 | exon (NM_001291465, exon 14 of 14).13 | TBC1D3D | protein-coding | |
| MACS_peak_24482 | 1225.96 | intron (NR_103444, intron 2 of 2) | LOC100996325 | ncRNA |  |
| MACS_peak_17004 | 1218.75 | exon (NM_001144950, exon 14 of 14) | SBK2 | protein-coding | |
| MACS_peak_8928 | 1212.51 | intron (NR_073547, intron 14 of 20) | NRXN3 | protein-coding | |
| MACS_peak_29749 | 1210.6 | non-coding (NR_003594, exon 1 of 1).3 | REXO1L2P | pseudo |  |
| MACS_peak_12311 | 1209.35 | intron (NM_174955, intron 18 of 21) | P2RX1 | protein-coding | |
| MACS_peak_20487 | 1208.75 | Intergenic | MIR3648-1 | ncRNA |  |
| MACS_peak_18855 | 1206.7 | intron (NM_001080437, intron 1 of 31) | SNED1 | protein-coding | |
| MACS_peak_19982 | 1205.02 | promoter-TSS (NR_147702) | LOC100128310 | ncRNA |  |
| MACS_peak_28470 | 1204.41 | intron (NR_023383, intron 2 of 10) | DTX2P1-UPK3BP1-PMS2P11 | pseudo |  |
| MACS_peak_25205 | 1204.11 | intron (NR_029426, intron 4 of 9) | LOC101929599 | pseudo |  |
| MACS_peak_29758 | 1202.37 | Intergenic | REXO1L2P | pseudo |  |
| MACS_peak_31577 | 1200.95 | intron (NM_001278074, intron 1 of 65) | COL5A1-AS1 | ncRNA |  |
| MACS_peak_14483 | 1198.36 | Intergenic | ANKRD62 | protein-coding | |
| MACS_peak_12074 | 1195.45 | intron (NM_001323544, intron 11 of 14) | APRT | protein-coding | |
| MACS_peak_5079 | 1193.01 | Intergenic | FRG2B | protein-coding | |
| MACS_peak_21257 | 1189.95 | intron (NM_001278641, intron 3 of 5) | SNORA77B | snoRNA |  |
| MACS_peak_22456 | 1189.26 | intron (NM_001350317, intron 13 of 13) | |  |  |
| MACS_peak_3535 | 1187.27 | intron (NM_015431, intron 5 of 5) | TRIM58 | protein-coding | |
| MACS_peak_20178 | 1185.43 | intron (NM_001302643, intron 11 of 13) | SLC17A9 | protein-coding | |
| MACS_peak_7719 | 1183.56 | Intergenic | LINC02414 | ncRNA |  |
| MACS_peak_28765 | 1182.97 | TTS (NM_001367767).2 | POLR2J3 | protein-coding | |
| MACS_peak_24555 | 1175.77 | intron (NM_006598, intron 1 of 23).2 | SLC12A7 | protein-coding | |
| MACS_peak_3361 | 1168.04 | TTS (NR_023363).7 | RNA5S3 | rRNA |  |
| MACS_peak_12898 | 1164.88 | exon (NM_001291465, exon 14 of 14).16 | TBC1D3I | protein-coding | |
| MACS_peak_1224 | 1164.01 | TTS (NR_027087) | LINC02800 | ncRNA |  |
| MACS_peak_29750 | 1157.51 | Intergenic | REXO1L2P | pseudo |  |
| MACS_peak_16161 | 1154.08 | intron (NM_001031696, intron 11 of 12) | MIR6796 | ncRNA |  |
| MACS_peak_20259 | 1151.15 | intron (NM_172109, intron 4 of 7) | KCNQ2 | protein-coding | |
| MACS_peak_24388 | 1149.18 | Intergenic | DUX4 | protein-coding | |
| MACS_peak_20183 | 1146.16 | Intergenic | BHLHE23 | protein-coding | |
| MACS_peak_7705 | 1144.13 | Intergenic | LINC02415 | ncRNA |  |
| MACS_peak_27782 | 1143.53 | promoter-TSS (NM_001127218) | POLD2 | protein-coding | |
| MACS_peak_30388 | 1142.68 | promoter-TSS (NM_000973) | RPL8 | protein-coding | |
| MACS_peak_29397 | 1140.58 | Intergenic | MIR596 | ncRNA |  |
| MACS_peak_18320 | 1136.13 | Intergenic | HS6ST1 | protein-coding | |
| MACS_peak_32009 | 1135.07 | NA |  |  |  |
| MACS_peak_14116 | 1131.91 | intron (NM_001144888, intron 3 of 13) | BAIAP2 | protein-coding | |
| MACS_peak_29765 | 1129.35 | Intergenic | REXO1L2P | pseudo |  |
| MACS_peak_30924 | 1119.63 | Intergenic | ZNF618 | protein-coding | |
| MACS_peak_5777 | 1118.78 | Intergenic | MYEOV | protein-coding | |
| MACS_peak_21430 | 1112.36 | intron (NM_015094, intron 1 of 2) | HIC2 | protein-coding | |
| MACS_peak_3586 | 1110.89 | promoter-TSS (NR_134300) | IDI1 | protein-coding | |
| MACS_peak_7125 | 1108.97 | promoter-TSS (NM_001029) | RPS26 | protein-coding | |
| MACS_peak_29744 | 1106.22 | non-coding (NR_003594, exon 1 of 1) | REXO1L2P | pseudo |  |
| MACS_peak_13614 | 1103.79 | Intergenic | LRRC37A3 | protein-coding | |
| MACS_peak_5132 | 1102.66 | Intergenic | FAM99A | ncRNA |  |
| MACS_peak_5072 | 1098.7 | Intergenic | FRG2B | protein-coding | |
| MACS_peak_9106 | 1097.3 | Intergenic | MIR376C | ncRNA |  |
| MACS_peak_30066 | 1095.59 | Intergenic | MROH5 | protein-coding | |
| MACS_peak_17140 | 1095.45 | intron (NM_012293, intron 1 of 22) | PXDN | protein-coding | |
| MACS_peak_5524 | 1092.03 | promoter-TSS (NR_028272) | NEAT1 | ncRNA |  |
| MACS_peak_8434 | 1084.37 | intron (NM_001365455, intron 6 of 6).2 | C13orf46 | protein-coding | |
| MACS_peak_30113 | 1079.61 | intron (NM_001366901, intron 12 of 13) | LINC00051 | ncRNA |  |
| MACS_peak_30825 | 1078.11 | Intergenic | NAMA | ncRNA |  |
| MACS_peak_10716 | 1076.55 | promoter-TSS (NR_132114) | TRAF7 | protein-coding | |
| MACS_peak_29757 | 1071.55 | Intergenic | REXO1L2P | pseudo |  |
| MACS_peak_29743 | 1070.89 | Intergenic | REXO1L2P | pseudo |  |
| MACS_peak_20078 | 1070.2 | intron (NM_005560, intron 2 of 79) | LAMA5-AS1 | ncRNA |  |
| MACS_peak_15585 | 1069.42 | Intergenic | LRRC25 | protein-coding | |
| MACS_peak_29770 | 1066.73 | Intergenic | REXO1L2P | pseudo |  |
| MACS_peak_29756 | 1065.86 | Intergenic | REXO1L2P | pseudo |  |
| MACS_peak_21367 | 1058.24 | exon (NM_001018060, exon 5 of 20) | AIFM3 | protein-coding | |
| MACS_peak_27379 | 1057.31 | intron (NM_001350838, intron 6 of 11) | ICA1 | protein-coding | |
| MACS_peak_20960 | 1056.72 | Intergenic | LOC102723769 | ncRNA |  |
| MACS_peak_25233 | 1049.01 | intron (NR_033968, intron 2 of 3).2 | GUSBP1 | pseudo |  |
| MACS_peak_24809 | 1048.77 | Intergenic | ADCY2 | protein-coding | |
| MACS_peak_24230 | 1047.22 | intron (NM_152542, intron 5 of 6) | PPM1K | protein-coding | |
| MACS_peak_26907 | 1047.18 | intron (NR_146964, intron 1 of 4) | MIR339 | ncRNA |  |
| MACS_peak_29769 | 1043.64 | Intergenic | REXO1L2P | pseudo |  |
| MACS_peak_15011 | 1043.32 | intron (NM_001370094, intron 2 of 10) | KDM4B | protein-coding | |
| MACS_peak_24651 | 1040.92 | Intergenic | LINC02116 | ncRNA |  |
| MACS_peak_5077 | 1039.21 | Intergenic | FRG2B | protein-coding | |
| MACS_peak_21451 | 1038.77 | promoter-TSS (NR_003700) | PI4KAP2 | pseudo |  |
| MACS_peak_22462 | 1036.76 | Intergenic | PANX2 | protein-coding | |
| MACS_peak_15981 | 1033.4 | Intergenic | ZNF565 | protein-coding | |
| MACS_peak_103 | 1033.15 | intron (NR_038869, intron 1 of 2) | LINC01342 | ncRNA |  |
| MACS_peak_24411 | 1031.82 | intron (NM_001080478, intron 1 of 1) | LRRC14B | protein-coding | |
| MACS_peak_5070 | 1024.95 | Intergenic | FRG2B | protein-coding | |
| MACS_peak_12264 | 1023.68 | intron (NM_001256827, intron 6 of 11) | SMG6 | protein-coding | |
| MACS_peak_11162 | 1019.4 | non-coding (NR_146331, exon 18 of 38) | MIR6770-1 | ncRNA |  |
| MACS_peak_20695 | 1019.04 | intron (NM_001040424, intron 21 of 23) | RIPK4 | protein-coding | |
| MACS_peak_2703 | 1013.58 | Intergenic | FCGR2A | protein-coding | |
| MACS_peak_8268 | 1008.95 | exon (NM_005537, exon 1 of 2) | ING1 | protein-coding | |
| MACS_peak_31192 | 1008.27 | TTS (NR_029619) | MIR199B | ncRNA |  |
| MACS_peak_7674 | 1004.56 | intron (NM_001351226, intron 1 of 21) | RIMBP2 | protein-coding | |
| MACS_peak_11156 | 1003.53 | promoter-TSS (NR_106766).4 | MIR6511A3 | ncRNA |  |
| MACS_peak_23330 | 1000.56 | Intergenic | MIR6825 | ncRNA |  |
| MACS_peak_30146 | 1000.4 | intron (NM_001702, intron 19 of 30) | ADGRB1 | protein-coding | |
| MACS_peak_12906 | 1000.29 | intron (NM_001369503, intron 13 of 13).3 | TBC1D3D | protein-coding | |
| MACS_peak_29745 | 1000.08 | Intergenic | REXO1L2P | pseudo |  |
| MACS_peak_29763 | 996.82 | non-coding (NR_003594, exon 1 of 1).7 | REXO1L2P | pseudo |  |
| MACS_peak_12150 | 996.25 | Intergenic | DPEP1 | protein-coding | |
| MACS_peak_29751 | 988.32 | Intergenic | REXO1L2P | pseudo |  |
| MACS_peak_846 | 985.19 | promoter-TSS (NR_046428) | CLCN6 | protein-coding | |
| MACS_peak_20711 | 976.11 | intron (NM_001004416, intron 12 of 22) | UMODL1-AS1 | ncRNA |  |
| MACS_peak_20307 | 974.76 | non-coding (NR_051954, exon 8 of 8) | TNFRSF6B | protein-coding | |
| MACS_peak_10746 | 972.72 | promoter-TSS (NR_036139) | MIR3178 | ncRNA |  |
| MACS_peak_24327 | 967.4 | Intergenic | TRIM2 | protein-coding | |
| MACS_peak_24401 | 966.91 | intron (NM_052909, intron 1 of 19) | PLEKHG4B | protein-coding | |
| MACS_peak_9377 | 966.74 | Intergenic | MAFIP | pseudo |  |
| MACS_peak_22547 | 964.44 | promoter-TSS (NR_003286).2 | MIR10396B | ncRNA |  |
| MACS_peak_27992 | 961.35 | intron (NM_001346941, intron 9 of 21) | EGFR-AS1 | ncRNA |  |
| MACS_peak_20483 | 957.91 | Intergenic | RNA28SN5 | rRNA |  |
| MACS_peak_22336 | 957.25 | Intergenic | WNT7B | protein-coding | |
| MACS_peak_5747 | 955.59 | Intergenic | TPCN2 | protein-coding | |
| MACS_peak_20088 | 955.55 | intron (NM_080833, intron 8 of 13) | CABLES2 | protein-coding | |
| MACS_peak_11260 | 953.87 | Intergenic | KDM8 | protein-coding | |
| MACS_peak_14173 | 950.68 | intron (NM_001291324, intron 1 of 27) | MIR4740 | ncRNA |  |
| MACS_peak_21426 | 947.56 | promoter-TSS (NM_015094) | HIC2 | protein-coding | |
| MACS_peak_26999 | 947.44 | Intergenic | TFAMP1 | pseudo |  |
| MACS_peak_22546 | 942.88 | Intergenic | MIR10396B | ncRNA |  |
| MACS_peak_21332 | 939.6 | Intergenic | POM121L4P | pseudo |  |
| MACS_peak_30311 | 939.1 | intron (NM_198572, intron 1 of 4) | SPATC1 | protein-coding | |
| MACS_peak_5069 | 938.92 | Intergenic | FRG2B | protein-coding | |
| MACS_peak_31804 | 937.66 | promoter-TSS (NR_039697) | MIR4479 | ncRNA |  |
| MACS_peak_26889 | 935.32 | intron (NM_015949, intron 6 of 8) | GET4 | protein-coding | |
| MACS_peak_29764 | 929.44 | Intergenic | REXO1L2P | pseudo |  |
| MACS_peak_24586 | 927.36 | intron (NM_198253, intron 6 of 15) | TERT | protein-coding | |
| MACS_peak_24554 | 927.16 | intron (NM_006598, intron 1 of 23).2 | SLC12A7 | protein-coding | |
| MACS_peak_29324 | 925.46 | intron (NM_130842, intron 1 of 21) | MIR595 | ncRNA |  |
| MACS_peak_25054 | 925.34 | intron (NR_146599, intron 3 of 11) | LOC646652 | pseudo |  |
| MACS_peak_26763 | 914.9 | Intergenic | LOC102724511 | ncRNA |  |
| MACS_peak_12202 | 911.59 | intron (NM_006987, intron 5 of 9).2 | LOC100506388 | protein-coding | |
| MACS_peak_7748 | 911.29 | 5' UTR (NM_025215, exon 1 of 6) | PUS1 | protein-coding | |
| MACS_peak_11115 | 910.6 | promoter-TSS (NR_106766).2 | MIR6511A3 | ncRNA |  |
| MACS_peak_7343 | 910.1 | intron (NM_001109662, intron 7 of 75) | HECTD4 | protein-coding | |
| MACS_peak_16055 | 907.17 | intron (NM_001042723, intron 70 of 104) | LOC105372397 | ncRNA |  |
| MACS_peak_30231 | 904.62 | Intergenic | MAFA-AS1 | ncRNA |  |
| MACS_peak_21443 | 904.35 | intron (NR_003700, intron 1 of 16) | PI4KAP2 | pseudo |  |
| MACS_peak_20069 | 904.2 | intron (NM_005560, intron 28 of 79) | MIR4758 | ncRNA |  |
| MACS_peak_17638 | 903.29 | Intergenic | LINC02580 | ncRNA |  |
| MACS_peak_26892 | 902.48 | intron (NM_006869, intron 4 of 10) | ADAP1 | protein-coding | |
| MACS_peak_15106 | 901.52 | intron (NM_015318, intron 1 of 20) | ARHGEF18 | protein-coding | |
| MACS_peak_24466 | 898.99 | intron (NM_001284351, intron 1 of 16) | SLC9A3 | protein-coding | |
| MACS_peak_16949 | 898.86 | promoter-TSS (NM_017729).8 | EPS8L1 | protein-coding | |
| MACS_peak_28675 | 898.82 | exon (NM_001164462, exon 2 of 12) | LOC102724094 | ncRNA |  |
| MACS_peak_18894 | 895.22 | intron (NR_135766, intron 2 of 2) | LOC285095 | ncRNA |  |
| MACS_peak_148 | 894.46 | promoter-TSS (NR_106866) | MIR6808 | ncRNA |  |
| MACS_peak_21433 | 891.47 | intron (NM_015094, intron 1 of 2) | HIC2 | protein-coding | |
| MACS_peak_9079 | 889.2 | intron (NM_004434, intron 17 of 21) | EVL | protein-coding | |
| MACS_peak_8420 | 882.34 | intron (NM_182614, intron 1 of 8) | TMEM255B | protein-coding | |
| MACS_peak_86 | 881.49 | Intergenic | RNF223 | protein-coding | |
| MACS_peak_980 | 877.7 | intron (NR_026752, intron 1 of 6) | CROCCP2 | pseudo |  |
| MACS_peak_29648 | 877.65 | promoter-TSS (NM_052937) | PCMTD1 | protein-coding | |
| MACS_peak_11735 | 877.54 | intron (NM_030629, intron 1 of 20) | MIR7854 | ncRNA |  |
| MACS_peak_30369 | 875.48 | exon (NM_004260, exon 13 of 21) | RECQL4 | protein-coding | |
| MACS_peak_29211 | 874.59 | Intergenic | BLACE | ncRNA |  |
| MACS_peak_2707 | 873.16 | Intergenic | FCGR2A | protein-coding | |
| MACS_peak_340 | 872.74 | intron (NM_001242672, intron 5 of 7) | TTC34 | protein-coding | |
| MACS_peak_14659 | 871.39 | TTS (NM_005317) | GZMM | protein-coding | |
| MACS_peak_2424 | 870.59 | promoter-TSS (NM_001367466) | DENND4B | protein-coding | |
| MACS_peak_27760 | 868.1 | non-coding (NR_024116, exon 9 of 9) | LINC00957 | ncRNA |  |
| MACS_peak_8266 | 866.45 | promoter-TSS (NM_001352252) | CARS2 | protein-coding | |
| MACS_peak_16804 | 865.41 | promoter-TSS (NR_038359) | LINC01869 | ncRNA |  |
| MACS_peak_17040 | 865.3 | intron (NM_018337, intron 3 of 4) | ZNF444 | protein-coding | |
| MACS_peak_26918 | 863.16 | intron (NM_001134395, intron 2 of 4) | GPR146 | protein-coding | |
| MACS_peak_28569 | 862.56 | Intergenic | TMEM130 | protein-coding | |
| MACS_peak_671 | 862.04 | Intergenic | PARK7 | protein-coding | |
| MACS_peak_7775 | 861.42 | intron (NM_001122636, intron 1 of 10) | LOC100130238 | ncRNA |  |
| MACS_peak_14380 | 861.05 | 5' UTR (NM_001303047, exon 1 of 5) | MYL12A | protein-coding | |
| MACS_peak_29978 | 859.02 | intron (NM_152888, intron 22 of 64) | COL22A1 | protein-coding | |
| MACS_peak_27229 | 858.27 | exon (NM_018059, exon 7 of 15) | SNORD165 | snoRNA |  |
| MACS_peak_8962 | 855.75 | intron (NM_001085471, intron 2 of 6) | FOXN3 | protein-coding | |
| MACS_peak_5106 | 854.54 | intron (NM_001025235, intron 3 of 8) | TSPAN4 | protein-coding | |
| MACS_peak_4720 | 854 | Intergenic | RPEL1 | protein-coding | |
| MACS_peak_27294 | 853.65 | intron (NM_024963, intron 3 of 4) | MIR589 | ncRNA |  |
| MACS_peak_20323 | 853.08 | intron (NM_001369741, intron 1 of 4) | ZBTB46 | protein-coding | |
| MACS_peak_8299 | 852.75 | Intergenic | LINC02337 | ncRNA |  |
| MACS_peak_7398 | 848.86 | intron (NM_173598, intron 3 of 19) | KSR2 | protein-coding | |
| MACS_peak_21194 | 847.22 | Intergenic | GP1BB | protein-coding | |
| MACS_peak_31895 | 846.84 | promoter-TSS (NM_152286) | PNPLA7 | protein-coding | |
| MACS_peak_27382 | 846.62 | Intergenic | PER4 | pseudo |  |
| MACS_peak_31954 | 843.01 | NA |  |  |  |
| MACS_peak_445 | 841.8 | intron (NM_001409, intron 4 of 36) | MIR551A | ncRNA |  |
| MACS_peak_5078 | 840.52 | Intergenic | FRG2B | protein-coding | |
| MACS_peak_2500 | 837.03 | promoter-TSS (NM_001256601) | TRIM46 | protein-coding | |
| MACS_peak_12865 | 835.4 | intron (NM_032258, intron 13 of 14).2 | TBC1D3K | protein-coding | |
| MACS_peak_26449 | 835.37 | intron (NR_132998, intron 3 of 5) | GUSBP4 | pseudo |  |
| MACS_peak_21217 | 832.31 | TTS (NM_006440) | GNB1L | protein-coding | |
| MACS_peak_76 | 830.19 | intron (NM_198576, intron 2 of 35) | AGRN | protein-coding | |
| MACS_peak_12916 | 829.28 | intron (NM_001001418, intron 13 of 13).3 | TBC1D3E | protein-coding | |
| MACS_peak_27147 | 829.09 | exon (NM_001284355, exon 5 of 6) | AMZ1 | protein-coding | |
| MACS_peak_21261 | 827.7 | 3' UTR (NM_013373, exon 11 of 11) | CCDC188 | protein-coding | |
| MACS_peak_1367 | 827.46 | Intergenic | FABP3 | protein-coding | |
| MACS_peak_24551 | 823.16 | intron (NM_006598, intron 9 of 23).2 | MIR4635 | ncRNA |  |
| MACS_peak_14498 | 822.62 | intron (NM_001142405, intron 2 of 6) | PRELID3A | protein-coding | |
| MACS_peak_13559 | 821.01 | intron (NM_001288780, intron 3 of 10) | 10-Mar | protein-coding | |
| MACS_peak_27834 | 816.62 | Intergenic | MYO1G | protein-coding | |
| MACS_peak_21378 | 816.27 | intron (NM_005446, intron 10 of 11) | SLC7A4 | protein-coding | |
| MACS_peak_10998 | 816 | promoter-TSS (NM_003745) | SOCS1 | protein-coding | |
| MACS_peak_25201 | 815.57 | intron (NR_027386, intron 4 of 5) | LOC101929599 | pseudo |  |
| MACS_peak_3305 | 812.13 | Intergenic | PRSS38 | protein-coding | |
| MACS_peak_26757 | 810.86 | Intergenic | LINC00242 | ncRNA |  |
| MACS_peak_19783 | 809.62 | intron (NR_136660, intron 5 of 7) | ZBP1 | protein-coding | |
| MACS_peak_5071 | 809.5 | Intergenic | FRG2B | protein-coding | |
| MACS_peak_404 | 809.01 | intron (NM_022114, intron 3 of 16) | ARHGEF16 | protein-coding | |
| MACS_peak_21260 | 807.97 | intron (NM_001185024, intron 5 of 10) | ZDHHC8 | protein-coding | |
| MACS_peak_21448 | 805.96 | intron (NR_003700, intron 1 of 16) | PI4KAP2 | pseudo |  |
| MACS_peak_21269 | 805.84 | Intergenic | LOC284865 | ncRNA |  |
| MACS_peak_6069 | 803.35 | promoter-TSS (NM_001235) | SERPINH1 | protein-coding | |
| MACS_peak_24389 | 800.56 | Intergenic | DUX4 | protein-coding | |
| MACS_peak_23836 | 799.28 | intron (NM_001328, intron 1 of 8) | CTBP1 | protein-coding | |
| MACS_peak_5080 | 798.55 | Intergenic | FRG2B | protein-coding | |
| MACS_peak_27110 | 798.05 | Intergenic | LFNG | protein-coding | |
| MACS_peak_22440 | 793.27 | Intergenic | C22orf34 | protein-coding | |
| MACS_peak_543 | 793.21 | intron (NR_111987, intron 18 of 32) | MIR4689 | ncRNA |  |
| MACS_peak_16026 | 792.96 | Intergenic | DPF1 | protein-coding | |
| MACS_peak_10693 | 791.91 | intron (NM_004548, intron 1 of 3) | NDUFB10 | protein-coding | |
| MACS_peak_29759 | 791.02 | TTS (NR_003594).6 | REXO1L2P | pseudo |  |
| MACS_peak_5404 | 786.81 | Intergenic | DAGLA | protein-coding | |
| MACS_peak_14080 | 784.79 | Intergenic | NPTX1 | protein-coding | |
| MACS_peak_26896 | 784.03 | intron (NM_006869, intron 3 of 10) | ADAP1 | protein-coding | |
| MACS_peak_14625 | 782.84 | intron (NR_148360, intron 6 of 31) | ATP9B | protein-coding | |
| MACS_peak_12563 | 781.56 | TTS (NR_003271) | SNORD3B-1 | snoRNA |  |
| MACS_peak_29378 | 781.4 | intron (NM_001346810, intron 2 of 14) | LOC105377777 | ncRNA |  |
| MACS_peak_21196 | 781.17 | Intergenic | GP1BB | protein-coding | |
| MACS_peak_2646 | 780.05 | promoter-TSS (NM_001277223) | TAGLN2 | protein-coding | |
| MACS_peak_411 | 779.15 | intron (NM_022114, intron 3 of 16) | ARHGEF16 | protein-coding | |
| MACS_peak_15116 | 775.46 | intron (NM_020902, intron 13 of 16) | MIR6792 | ncRNA |  |
| MACS_peak_14626 | 775.31 | intron (NR_148360, intron 26 of 31) | NFATC1 | protein-coding | |
| MACS_peak_20079 | 774.35 | intron (NM_005560, intron 1 of 79) | LAMA5 | protein-coding | |
| MACS_peak_24047 | 773.82 | intron (NM_003501, intron 5 of 17) | ACOX3 | protein-coding | |
| MACS_peak_31365 | 773.76 | Intergenic | FIBCD1 | protein-coding | |
| MACS_peak_12159 | 773.23 | promoter-TSS (NM_153025) | CHMP1A | protein-coding | |
| MACS_peak_24960 | 771.77 | intron (NR_027253, intron 2 of 2) | MIR10522 | ncRNA |  |
| MACS_peak_21386 | 770.12 | intron (NR_037566, intron 3 of 5) | BCRP2 | pseudo |  |
| MACS_peak_20091 | 769.84 | intron (NM_080833, intron 1 of 13) | RBBP8NL | protein-coding | |
| MACS_peak_26872 | 769.72 | intron (NR_075098, intron 4 of 12) | PRKAR1B | protein-coding | |
| MACS_peak_21675 | 768.69 | intron (NM_001278500, intron 1 of 2) | ADORA2A | protein-coding | |
| MACS_peak_22262 | 768.61 | intron (NM_001362786, intron 5 of 6) | EFCAB6-AS1 | ncRNA |  |
| MACS_peak_21895 | 766.15 | intron (NR_040114, intron 1 of 3) | AP1B1P1 | pseudo |  |
| MACS_peak_14860 | 764.8 | intron (NM_032737, intron 1 of 11) | LMNB2 | protein-coding | |
| MACS_peak_24180 | 763.24 | Intergenic | LINC02429 | ncRNA |  |
| MACS_peak_12895 | 763.15 | intron (NM_001123391, intron 13 of 13) | TBC1D3K | protein-coding | |
| MACS_peak_18129 | 761.07 | intron (NM_001351428, intron 1 of 24) | INPP4A | protein-coding | |
| MACS_peak_21664 | 760.92 | TTS (NM_019601) | SUSD2 | protein-coding | |
| MACS_peak_32026 | 760.9 | NA |  |  |  |
| MACS_peak_13245 | 757.12 | intron (NR_027782, intron 8 of 10) | ARHGAP27 | protein-coding | |
| MACS_peak_15325 | 756.51 | intron (NM_001370095, intron 2 of 9) | PODNL1 | protein-coding | |
| MACS_peak_21149 | 756.09 | promoter-TSS (NR_103767) | LINC01311 | ncRNA |  |
| MACS_peak_7814 | 756.04 | intron (NM_018663, intron 1 of 4) | PXMP2 | protein-coding | |
| MACS_peak_29170 | 755.63 | intron (NM_016203, intron 1 of 15) | PRKAG2 | protein-coding | |
| MACS_peak_17766 | 755.37 | intron (NM_001363864, intron 2 of 3) | MIR4432HG | ncRNA |  |
| MACS_peak_7783 | 754.7 | Intergenic | LOC101928416 | ncRNA |  |
| MACS_peak_5107 | 754.17 | intron (NM_023947, intron 7 of 12) | CHID1 | protein-coding | |
| MACS_peak_25214 | 752.48 | intron (NR_027386, intron 4 of 5).2 | SMA4 | pseudo |  |
| MACS_peak_29244 | 751.49 | Intergenic | SHH | protein-coding | |
| MACS_peak_16624 | 751.38 | TTS (NM_016246) | BCAT2 | protein-coding | |
| MACS_peak_10715 | 748.62 | intron (NM_014353, intron 2 of 8) | RAB26 | protein-coding | |
| MACS_peak_5027 | 747.35 | Intergenic | LINC01168 | ncRNA |  |
| MACS_peak_2528 | 746.02 | promoter-TSS (NM_014949) | KHDC4 | protein-coding | |
| MACS_peak_25267 | 744.41 | intron (NM_006909, intron 17 of 26) | CKMT2 | protein-coding | |
| MACS_peak_12201 | 742.66 | intron (NM_006987, intron 5 of 9).2 | LOC100506388 | protein-coding | |
| MACS_peak_19745 | 738.56 | TTS (NR_110631) | BMP7-AS1 | ncRNA |  |
| MACS_peak_2702 | 737.17 | Intergenic | FCGR2A | protein-coding | |
| MACS_peak_704 | 734.69 | Intergenic | MIR34AHG | ncRNA |  |
| MACS_peak_17148 | 733.8 | Intergenic | MYT1L | protein-coding | |
| MACS_peak_24530 | 733.67 | Intergenic | LOC100506688 | ncRNA |  |
| MACS_peak_29292 | 731.4 | intron (NM_130843, intron 11 of 21) | LOC100506585 | ncRNA |  |
| MACS_peak_4317 | 730.54 | Intergenic | DNAJB12 | protein-coding | |
| MACS_peak_20106 | 729.46 | Intergenic | GATA5 | protein-coding | |
| MACS_peak_20909 | 729.39 | intron (NR_145493, intron 10 of 12) | LOC101928796 | ncRNA |  |
| MACS_peak_18221 | 728.65 | Intergenic | ZC3H6 | protein-coding | |
| MACS_peak_28788 | 726.24 | non-coding (NR_146066, exon 12 of 12) | POLR2J2 | protein-coding | |
| MACS_peak_21072 | 720.34 | Intergenic | PI4KAP1 | pseudo |  |
| MACS_peak_10665 | 720.23 | intron (NM_001351846, intron 1 of 20) | TELO2 | protein-coding | |
| MACS_peak_18875 | 720.05 | intron (NM_032515, intron 4 of 4) | BOK-AS1 | ncRNA |  |
| MACS_peak_7309 | 719.75 | Intergenic | MVK | protein-coding | |
| MACS_peak_18767 | 717.72 | Intergenic | ASB1 | protein-coding | |
| MACS_peak_8369 | 717.55 | intron (NR_051961, intron 2 of 6) | F7 | protein-coding | |
| MACS_peak_7005 | 716.45 | Intergenic | KRT80 | protein-coding | |
| MACS_peak_16966 | 714.97 | intron (NM_002842, intron 1 of 19) | PTPRH | protein-coding | |
| MACS_peak_18063 | 714.6 | Intergenic | ACTR3BP2 | pseudo |  |
| MACS_peak_24615 | 711.99 | promoter-TSS (NM_024830) | LPCAT1 | protein-coding | |
| MACS_peak_10189 | 711.44 | intron (NR_033738, intron 1 of 7) | LOC440300 | pseudo |  |
| MACS_peak_31179 | 710.92 | non-coding (NR_033374, exon 1 of 5) | SLC25A25-AS1 | ncRNA |  |
| MACS_peak_14820 | 710.77 | intron (NM_017797, intron 1 of 8) | BTBD2 | protein-coding | |
| MACS_peak_16974 | 710.07 | intron (NM_032430, intron 1 of 18) | BRSK1 | protein-coding | |
| MACS_peak_21136 | 710.07 | Intergenic | DGCR10 | ncRNA |  |
| MACS_peak_27091 | 709.89 | Intergenic | CHST12 | protein-coding | |
| MACS_peak_3362 | 708.4 | promoter-TSS (NR_023363).8 | RNA5S14 | rRNA |  |
| MACS_peak_31949 | 706.95 | NA |  |  |  |
| MACS_peak_28597 | 706.09 | promoter-TSS (NM_004722) | MCM7 | protein-coding | |
| MACS_peak_27261 | 705.77 | intron (NM_001080495, intron 21 of 29) | SLC29A4 | protein-coding | |
| MACS_peak_7405 | 704.72 | Intergenic | LINC02423 | ncRNA |  |
| MACS_peak_10239 | 704.3 | Intergenic | LOC101929479 | pseudo |  |
| MACS_peak_29415 | 703.67 | intron (NM_033225, intron 2 of 69) | CSMD1 | protein-coding | |
| MACS_peak_23989 | 702.33 | intron (NM_001292038, intron 3 of 18) | MAN2B2 | protein-coding | |
| MACS_peak_18343 | 701.9 | Intergenic | CCDC115 | protein-coding | |
| MACS_peak_21504 | 698.65 | Intergenic | PRAMENP | pseudo |  |
| MACS_peak_6083 | 698.12 | Intergenic | WNT11 | protein-coding | |
| MACS_peak_5115 | 695.83 | exon (NM_002457, exon 32 of 61) | MUC2 | protein-coding | |
| MACS_peak_26857 | 693.68 | intron (NM_001164760, intron 7 of 10) | LOC101926963 | ncRNA |  |
| MACS_peak_27059 | 692.89 | intron (NM_001013837, intron 12 of 18) | SNORA114 | snoRNA |  |
| MACS_peak_31590 | 691.88 | intron (NM_001278074, intron 18 of 65) | MIR3689C | ncRNA |  |
| MACS_peak_5074 | 691.47 | Intergenic | FRG2B | protein-coding | |
| MACS_peak_31269 | 691.36 | Intergenic | LINC01503 | ncRNA |  |
| MACS_peak_20351 | 691.36 | intron (NM_003195, intron 1 of 9) | TCEA2 | protein-coding | |
| MACS_peak_2705 | 690.88 | Intergenic | FCGR2A | protein-coding | |
| MACS_peak_21435 | 690.69 | exon (NM_015094, exon 3 of 3) | TMEM191C | protein-coding | |
| MACS_peak_8394 | 690.66 | Intergenic | GRTP1 | protein-coding | |
| MACS_peak_21442 | 690.54 | intron (NR_003700, intron 1 of 16) | PI4KAP2 | pseudo |  |
| MACS_peak_4474 | 690.42 | intron (NR_015429, intron 3 of 7) | ZMIZ1-AS1 | ncRNA |  |
| MACS_peak_27863 | 689.8 | intron (NM_001281768, intron 3 of 9) | ADCY1 | protein-coding | |
| MACS_peak_13077 | 688.87 | intron (NM_001352776, intron 1 of 15) | JUP | protein-coding | |
| MACS_peak_22701 | 686.66 | intron (NM_001330619, intron 2 of 12) | IQSEC1 | protein-coding | |
| MACS_peak_16417 | 685.3 | exon (NM_001017989, exon 2 of 2) | VASP | protein-coding | |
| MACS_peak_20167 | 685.11 | intron (NM_001853, intron 15 of 31) | COL9A3 | protein-coding | |
| MACS_peak_27799 | 684.89 | intron (NM_172081, intron 4 of 18) | YKT6 | protein-coding | |
| MACS_peak_20008 | 682.49 | intron (NM_001252339, intron 6 of 14) | MIR1257 | ncRNA |  |
| MACS_peak_8684 | 677.44 | Intergenic | SAMD4A | protein-coding | |
| MACS_peak_21405 | 674.33 | intron (NR_024583, intron 5 of 6) | POM121L8P | pseudo |  |
| MACS_peak_28203 | 674.26 | Intergenic | LOC100101148 | pseudo |  |
| MACS_peak_19939 | 674.05 | intron (NM_001794, intron 2 of 15) | CDH4 | protein-coding | |
| MACS_peak_25212 | 673.62 | intron (NR_029426, intron 6 of 9).2 | SMA4 | pseudo |  |
| MACS_peak_13995 | 673.16 | intron (NM_001350451, intron 1 of 14) | RBFOX3 | protein-coding | |
| MACS_peak_12858 | 672.6 | 3' UTR (NM_001291462, exon 14 of 14).6 | TBC1D3D | protein-coding | |
| MACS_peak_14832 | 672.06 | intron (NM_003938, intron 8 of 29) | AP3D1 | protein-coding | |
| MACS_peak_462 | 671.32 | intron (NM_001204189, intron 1 of 8) | TP73 | protein-coding | |
| MACS_peak_17798 | 669.51 | promoter-TSS (NM_014181) | LGALSL | protein-coding | |
| MACS_peak_20845 | 667.69 | Intergenic | UBE2G2 | protein-coding | |
| MACS_peak_30215 | 666.65 | intron (NM_052963, intron 7 of 13) | TOP1MT | protein-coding | |
| MACS_peak_19746 | 666.53 | intron (NM_001719, intron 2 of 6) | BMP7-AS1 | ncRNA |  |
| MACS_peak_14972 | 666.36 | intron (NM_001330429, intron 10 of 12) | STAP2 | protein-coding | |
| MACS_peak_1368 | 666.1 | intron (NM_178865, intron 8 of 9) | SERINC2 | protein-coding | |
| MACS_peak_24641 | 665.34 | Intergenic | MIR4277 | ncRNA |  |
| MACS_peak_25230 | 664.01 | intron (NR_027028, intron 1 of 2) | GUSBP1 | pseudo |  |
| MACS_peak_20484 | 662.11 | Intergenic | RNA28SN5 | rRNA |  |
| MACS_peak_24484 | 661.22 | intron (NM_018140, intron 9 of 11) | CEP72 | protein-coding | |
| MACS_peak_30404 | 660.75 | intron (NM_001193536, intron 25 of 46) | KANK1 | protein-coding | |
| MACS_peak_32040 | 660.55 | Intergenic | PLCXD1 | protein-coding | |
| MACS_peak_337 | 660.43 | intron (NM_001242672, intron 5 of 7) | MMEL1 | protein-coding | |
| MACS_peak_177 | 660.41 | TTS (NM_001317238) | ATAD3A | protein-coding | |
| MACS_peak_20252 | 659.91 | 3' UTR (NM_172108, exon 16 of 16) | CHRNA4 | protein-coding | |
| MACS_peak_5081 | 659.77 | Intergenic | FRG2B | protein-coding | |
| MACS_peak_16953 | 659.77 | exon (NM_017607, exon 18 of 22) | EPS8L1 | protein-coding | |
| MACS_peak_27486 | 659.19 | Intergenic | NPY | protein-coding | |
| MACS_peak_17441 | 658.91 | intron (NR_104246, intron 1 of 15) | AGBL5 | protein-coding | |
| MACS_peak_21364 | 658.58 | intron (NR_110537, intron 1 of 1) | AIFM3 | protein-coding | |
| MACS_peak_4149 | 658.19 | TTS (NM_145306) | FAM241B | protein-coding | |
| MACS_peak_22957 | 656.82 | promoter-TSS (NM_001304288) | RPSA | protein-coding | |
| MACS_peak_22526 | 656.29 | TTS (NR_003267).2 | GGT3P | pseudo |  |
| MACS_peak_19767 | 655.54 | Intergenic | LOC100291105 | ncRNA |  |
| MACS_peak_29319 | 655.11 | intron (NM_130842, intron 1 of 21) | MIR595 | ncRNA |  |
| MACS_peak_7154 | 654.89 | exon (NM_001365896, exon 3 of 9) | NACA | protein-coding | |
| MACS_peak_17128 | 654.68 | intron (NM_018968, intron 9 of 16) | TPO | protein-coding | |
| MACS_peak_29300 | 654.16 | intron (NM_130843, intron 10 of 21) | LOC100506585 | ncRNA |  |
| MACS_peak_24150 | 652.96 | Intergenic | CWH43 | protein-coding | |
| MACS_peak_14269 | 652.65 | promoter-TSS (NR_135639) | LINC01970 | ncRNA |  |
| MACS_peak_15359 | 652.49 | Intergenic | PTGER1 | protein-coding | |
| MACS_peak_29136 | 650.27 | intron (NM_001282292, intron 14 of 15) | ASIC3 | protein-coding | |
| MACS_peak_22457 | 649.93 | intron (NM_001350317, intron 1 of 13) | TTLL8 | protein-coding | |
| MACS_peak_16602 | 649.5 | Intergenic | SULT2B1 | protein-coding | |
| MACS_peak_7016 | 647.78 | TTS (NR_146274) | KRT7-AS | ncRNA |  |
| MACS_peak_16692 | 647.54 | intron (NM_020719, intron 1 of 13) | PRR12 | protein-coding | |
| MACS_peak_21258 | 646.19 | intron (NM_001278641, intron 3 of 5) | SNORA77B | snoRNA |  |
| MACS_peak_9387 | 645.84 | NA |  |  |  |
| MACS_peak_20664 | 645.04 | non-coding (NR_148920, exon 1 of 1) | PLAC4 | ncRNA |  |
| MACS_peak_30749 | 644.51 | Intergenic | C9orf129 | protein-coding | |
| MACS_peak_5922 | 644.32 | intron (NM_018161, intron 13 of 20) | MIR6754 | ncRNA |  |
| MACS_peak_393 | 643.71 | intron (NM_022114, intron 1 of 16) | MIR4251 | ncRNA |  |
| MACS_peak_25542 | 642.65 | Intergenic | GABRP | protein-coding | |
| MACS_peak_7746 | 641.66 | intron (NM_003565, intron 23 of 27) | PUS1 | protein-coding | |
| MACS_peak_12130 | 640.05 | intron (NR_045839, intron 5 of 9) | LOC105371414 | ncRNA |  |
| MACS_peak_20362 | 639.58 | Intergenic | NPBWR2 | protein-coding | |
| MACS_peak_27886 | 639.47 | promoter-TSS (NM_000598) | IGFBP3 | protein-coding | |
| MACS_peak_3355 | 639.02 | Intergenic | MIR4666A | ncRNA |  |
| MACS_peak_22775 | 638.81 | non-coding (NR_002223, exon 3 of 3) | CHCHD4 | protein-coding | |
| MACS_peak_27216 | 637.98 | exon (NM_001037165, exon 2 of 9) | AP5Z1 | protein-coding | |
| MACS_peak_25743 | 637.94 | intron (NR_104473, intron 3 of 5) | DUSP22 | protein-coding | |
| MACS_peak_15084 | 637.84 | intron (NM_003811, intron 1 of 2) | TNFSF9 | protein-coding | |
| MACS_peak_30069 | 636.84 | Intergenic | MROH5 | protein-coding | |
| MACS_peak_27592 | 636.26 | intron (NR_037598, intron 17 of 19) | AQP1 | protein-coding | |
| MACS_peak_3363 | 636.26 | TTS (NR_023371) | RNA5S9 | rRNA |  |
| MACS_peak_9337 | 635.95 | Intergenic | ELK2AP | pseudo |  |
| MACS_peak_27184 | 635.41 | intron (NM_152744, intron 13 of 44) | SDK1 | protein-coding | |
| MACS_peak_5068 | 634.67 | Intergenic | FRG2B | protein-coding | |
| MACS_peak_4430 | 631.43 | exon (NM_004747, exon 3 of 32).2 | DLG5 | protein-coding | |
| MACS_peak_16670 | 631.24 | promoter-TSS (NR_110729) | CCDC155 | protein-coding | |
| MACS_peak_25331 | 631.12 | Intergenic | LMNB1 | protein-coding | |
| MACS_peak_20114 | 631.09 | Intergenic | MIR1-1HG | protein-coding | |
| MACS_peak_3565 | 630.88 | intron (NM_014974, intron 26 of 36) | ZMYND11 | protein-coding | |
| MACS_peak_24395 | 630.84 | Intergenic | PLEKHG4B | protein-coding | |
| MACS_peak_7598 | 630.74 | promoter-TSS (NM_001367982) | SCARB1 | protein-coding | |
| MACS_peak_17153 | 630.4 | Intergenic | MYT1L | protein-coding | |
| MACS_peak_4978 | 626.85 | intron (NM_001143757, intron 8 of 10) | LRRC27 | protein-coding | |
| MACS_peak_10792 | 626.79 | intron (NM_016639, intron 1 of 3) | TNFRSF12A | protein-coding | |
| MACS_peak_12075 | 626.59 | intron (NM_001323544, intron 10 of 14) | APRT | protein-coding | |
| MACS_peak_18315 | 625.91 | intron (NM_001330299, intron 14 of 20) | SAP130 | protein-coding | |
| MACS_peak_22877 | 625.35 | intron (NM_001354708, intron 2 of 9) | LOC101927854 | ncRNA |  |
| MACS_peak_17995 | 624.39 | Intergenic | MIR4771-1 | ncRNA |  |
| MACS_peak_405 | 624.37 | intron (NM_022114, intron 3 of 16) | ARHGEF16 | protein-coding | |
| MACS_peak_9244 | 623.11 | Intergenic | MIR4710 | ncRNA |  |
| MACS_peak_23858 | 622.99 | intron (NM_001174070, intron 2 of 4) | FAM53A | protein-coding | |
| MACS_peak_12978 | 622.95 | TTS (NM_181505) | STARD3 | protein-coding | |
| MACS_peak_28224 | 622.72 | Intergenic | SPDYE8P | pseudo |  |
| MACS_peak_21077 | 622.32 | Intergenic | PI4KAP1 | pseudo |  |
| MACS_peak_30222 | 622.12 | Intergenic | RHPN1 | protein-coding | |
| MACS_peak_14889 | 621.59 | intron (NM_021217, intron 1 of 3) | ZNF77 | protein-coding | |
| MACS_peak_30017 | 621.44 | intron (NM_001164623, intron 1 of 17) | AGO2 | protein-coding | |
| MACS_peak_23896 | 621.2 | intron (NM_001292016, intron 1 of 4) | TNIP2 | protein-coding | |
| MACS_peak_30164 | 621.04 | intron (NM_005672, intron 2 of 2) | PSCA | protein-coding | |
| MACS_peak_8158 | 620.54 | intron (NM_002271, intron 3 of 28) | IPO5 | protein-coding | |
| MACS_peak_16615 | 620.49 | intron (NR_004401, intron 3 of 4) | NTN5 | protein-coding | |
| MACS_peak_9935 | 620.18 | intron (NM_001038640, intron 13 of 17) | GOLGA6A | protein-coding | |
| MACS_peak_5746 | 617.06 | intron (NR_120541, intron 1 of 5) | MRGPRF-AS1 | ncRNA |  |
| MACS_peak_4485 | 616.23 | intron (NM_020338, intron 1 of 24) | ZMIZ1 | protein-coding | |
| MACS_peak_24538 | 615.56 | intron (NM_001271082, intron 3 of 10) | NKD2 | protein-coding | |
| MACS_peak_14335 | 615.49 | Intergenic | METRNL | protein-coding | |
| MACS_peak_25056 | 615.47 | non-coding (NR_157803, exon 1 of 1) | LOC646652 | pseudo |  |
| MACS_peak_21373 | 615.33 | exon (NM_030573, exon 3 of 4) | THAP7-AS1 | ncRNA |  |
| MACS_peak_68 | 614.57 | Intergenic | HES4 | protein-coding | |
| MACS_peak_10605 | 613.23 | Intergenic | C1QTNF8 | protein-coding | |
| MACS_peak_7766 | 612.18 | intron (NM_001122636, intron 6 of 10) | GALNT9 | protein-coding | |
| MACS_peak_20257 | 611.23 | intron (NM_004518, intron 8 of 14) | KCNQ2 | protein-coding | |
| MACS_peak_30530 | 610.29 | Intergenic | MIR4477A | ncRNA |  |
| MACS_peak_30509 | 610.06 | exon (NM_001085452, exon 4 of 4) | SPATA31A1 | protein-coding | |
| MACS_peak_20852 | 609.96 | non-coding (NR_104597, exon 1 of 5) | PTTG1IP | protein-coding | |
| MACS_peak_14177 | 609.82 | intron (NM_001291324, intron 2 of 27) | MIR3186 | ncRNA |  |
| MACS_peak_20510 | 609.35 | Intergenic | BAGE | protein-coding | |
| MACS_peak_11366 | 609.07 | intron (NM_001172432, intron 2 of 10) | PHKG2 | protein-coding | |
| MACS_peak_13298 | 608.95 | promoter-TSS (NM_014726) | TBKBP1 | protein-coding | |
| MACS_peak_12641 | 607.78 | Intergenic | FLJ36000 | ncRNA |  |
| MACS_peak_28414 | 606.98 | intron (NM_001099415, intron 13 of 14) | NSUN5P1 | pseudo |  |
| MACS_peak_20001 | 606.27 | intron (NM_001252339, intron 5 of 14) | MIR1257 | ncRNA |  |
| MACS_peak_19478 | 605.78 | Intergenic | LINC01523 | ncRNA |  |
| MACS_peak_265 | 604.46 | intron (NM_003036, intron 1 of 6) | SKI | protein-coding | |
| MACS_peak_5011 | 602.16 | intron (NM_001200049, intron 50 of 57) | NKX6-2 | protein-coding | |
| MACS_peak_21608 | 601.21 | TTS (NM_182520).2 | CHCHD10 | protein-coding | |
| MACS_peak_25579 | 601.08 | intron (NM_015980, intron 3 of 4) | NSG2 | protein-coding | |
| MACS_peak_21636 | 600.6 | promoter-TSS (NM_001355) | GSTT2B | protein-coding | |
| MACS_peak_22543 | 600.51 | Intergenic | RNA28SN1 | rRNA |  |
| MACS_peak_26989 | 600.41 | intron (NM_001080453, intron 2 of 47) | INTS1 | protein-coding | |
| MACS_peak_10527 | 600.22 | TTS (NM_145270) | NHLRC4 | protein-coding | |
| MACS_peak_28780 | 599.61 | intron (NM_032959, intron 8 of 8) | UPK3BL1 | protein-coding | |
| MACS_peak_14631 | 599.48 | intron (NM_012283, intron 1 of 1) | KCNG2 | protein-coding | |
| MACS_peak_21076 | 597.42 | Intergenic | PI4KAP1 | pseudo |  |
| MACS_peak_24643 | 596.84 | Intergenic | MRPL36 | protein-coding | |
| MACS_peak_25220 | 596.12 | intron (NR_033968, intron 2 of 3) | GUSBP9 | pseudo |  |
| MACS_peak_20336 | 596 | TTS (NM_020713) | UCKL1 | protein-coding | |
| MACS_peak_9201 | 595.31 | Intergenic | LINC02691 | ncRNA |  |
| MACS_peak_21151 | 594.07 | Intergenic | LINC01311 | ncRNA |  |
| MACS_peak_21043 | 593.79 | intron (NM_015241, intron 1 of 31) | MIR648 | ncRNA |  |
| MACS_peak_7812 | 593.4 | TTS (NR_106821) | MIR6763 | ncRNA |  |
| MACS_peak_22558 | 592.34 | Intergenic | LOC102724728 | pseudo |  |
| MACS_peak_205 | 591.89 | intron (NM_001290264, intron 1 of 9) | SLC35E2B | protein-coding | |
| MACS_peak_14877 | 591.71 | intron (NM_052847, intron 1 of 4) | GNG7 | protein-coding | |
| MACS_peak_19436 | 590.51 | intron (NM_001193340, intron 1 of 12) | SLC13A3 | protein-coding | |
| MACS_peak_14873 | 590.34 | intron (NM_052847, intron 2 of 4) | MIR7850 | ncRNA |  |
| MACS_peak_14926 | 590.28 | intron (NM_006339, intron 2 of 9) | HMG20B | protein-coding | |
| MACS_peak_12915 | 590.14 | TTS (NM_001291466) | TBC1D3E | protein-coding | |
| MACS_peak_82 | 589.94 | TTS (NM_001305275) | LOC100288175 | ncRNA |  |
| MACS_peak_11164 | 589.82 | intron (NR_146331, intron 14 of 37) | MIR6770-1 | ncRNA |  |
| MACS_peak_13233 | 589.74 | Intergenic | LOC105371795 | ncRNA |  |
| MACS_peak_13046 | 589.68 | exon (NM_002275, exon 1 of 8) | KRT15 | protein-coding | |
| MACS_peak_5127 | 588.61 | intron (NM_019009, intron 1 of 5) | TOLLIP | protein-coding | |
| MACS_peak_10919 | 588.35 | promoter-TSS (NM_001142334) | RBFOX1 | protein-coding | |
| MACS_peak_418 | 587.8 | intron (NM_022114, intron 9 of 16) | ARHGEF16 | protein-coding | |
| MACS_peak_569 | 587.64 | intron (NM_015557, intron 4 of 41) | CHD5 | protein-coding | |
| MACS_peak_5061 | 587.39 | intron (NM_004092, intron 1 of 7) | ECHS1 | protein-coding | |
| MACS_peak_153 | 586.77 | exon (NM_001282584, exon 4 of 9) | MXRA8 | protein-coding | |
| MACS_peak_15513 | 586.02 | exon (NM_138401, exon 1 of 9) | MVB12A | protein-coding | |
| MACS_peak_30181 | 585.66 | Intergenic | CDC42P3 | pseudo |  |
| MACS_peak_26833 | 585.54 | Intergenic | PDGFA | protein-coding | |
| MACS_peak_26883 | 585.35 | promoter-TSS (NM_001367665) | SUN1 | protein-coding | |
| MACS_peak_24399 | 585.28 | Intergenic | PLEKHG4B | protein-coding | |
| MACS_peak_29317 | 585.28 | intron (NM_130842, intron 1 of 21) | MIR595 | ncRNA |  |
| MACS_peak_30293 | 584.53 | intron (NM_031308, intron 1 of 1) | EPPK1 | protein-coding | |
| MACS_peak_8464 | 584.08 | Intergenic | RASA3 | protein-coding | |
| MACS_peak_6277 | 583.3 | 5' UTR (NM_001282098, exon 1 of 7) | YAP1 | protein-coding | |
| MACS_peak_27221 | 581.48 | TTS (NM_001037165) | AP5Z1 | protein-coding | |
| MACS_peak_16679 | 580.49 | intron (NM_153329, intron 7 of 16) | ALDH16A1 | protein-coding | |
| MACS_peak_12652 | 580.35 | Intergenic | FLJ36000 | ncRNA |  |
| MACS_peak_10238 | 580.18 | intron (NM_001243137, intron 20 of 21) | LOC101929479 | pseudo |  |
| MACS_peak_298 | 579.47 | Intergenic | PLCH2 | protein-coding | |
| MACS_peak_16191 | 578.91 | Intergenic | NUMBL | protein-coding | |
| MACS_peak_29946 | 578.91 | Intergenic | ST3GAL1 | protein-coding | |
| MACS_peak_26637 | 578.02 | intron (NM_024630, intron 7 of 8) | MIR3692 | ncRNA |  |
| MACS_peak_5505 | 577.72 | promoter-TSS (NM_017525) | CDC42BPG | protein-coding | |
| MACS_peak_13950 | 577.36 | intron (NM_001321291, intron 17 of 20) | CYTH1 | protein-coding | |
| MACS_peak_11409 | 576.47 | Intergenic | SLC6A10P | pseudo |  |
| MACS_peak_16062 | 576.19 | intron (NM_007181, intron 16 of 31) | MAP4K1 | protein-coding | |
| MACS_peak_1396 | 575.85 | Intergenic | ZBTB8OS | protein-coding | |
| MACS_peak_9260 | 575.65 | exon (NM_152328, exon 1 of 13) | ADSSL1 | protein-coding | |
| MACS_peak_26905 | 575.21 | TTS (NM_017781) | CYP2W1 | protein-coding | |
| MACS_peak_29385 | 574.96 | intron (NM_001346810, intron 3 of 14) | LOC286083 | ncRNA |  |
| MACS_peak_633 | 573.8 | intron (NM_001349608, intron 3 of 21) | LOC102725193 | ncRNA |  |
| MACS_peak_10848 | 573.61 | intron (NM_003223, intron 1 of 6) | TFAP4 | protein-coding | |
| MACS_peak_16098 | 573.49 | intron (NM_001014834, intron 1 of 7) | PAK4 | protein-coding | |
| MACS_peak_21114 | 573.18 | non-coding (NR_135922, exon 4 of 6) | LOC102725072 | pseudo |  |
| MACS_peak_2152 | 572.65 | Intergenic | NBPF8 | protein-coding | |
| MACS_peak_27598 | 570.24 | intron (NM_198098, intron 1 of 3) | AQP1 | protein-coding | |
| MACS_peak_24385 | 569.68 | Intergenic | DUX4 | protein-coding | |
| MACS_peak_14201 | 569.62 | intron (NR_026872, intron 2 of 2) | PDE6G | protein-coding | |
| MACS_peak_11377 | 568.98 | promoter-TSS (NM_001142777) | HSD3B7 | protein-coding | |
| MACS_peak_16053 | 568.45 | intron (NM_001042723, intron 45 of 104) | RYR1 | protein-coding | |
| MACS_peak_28623 | 568.12 | promoter-TSS (NM_001194991) | ZCWPW1 | protein-coding | |
| MACS_peak_24378 | 568.07 | Intergenic | DUX4 | protein-coding | |
| MACS_peak_29039 | 566.33 | intron (NM_001363538, intron 11 of 11) | MIR11400 | ncRNA |  |
| MACS_peak_10070 | 566.23 | promoter-TSS (NM_001301189) | LINGO1 | protein-coding | |
| MACS_peak_12850 | 565.78 | intron (NM_001291463, intron 13 of 13).14 | TBC1D3I | protein-coding | |
| MACS_peak_27896 | 565.48 | Intergenic | LOC730338 | ncRNA |  |
| MACS_peak_23681 | 565.42 | intron (NM_018406, intron 7 of 24) | MUC4 | protein-coding | |
| MACS_peak_14522 | 565.33 | intron (NM_181482, intron 2 of 5) | LDLRAD4-AS1 | ncRNA |  |
| MACS_peak_20218 | 564.45 | intron (NM_001363718, intron 5 of 5) | MIR3196 | ncRNA |  |
| MACS_peak_20724 | 564.07 | intron (NM_207627, intron 3 of 15) | ABCG1 | protein-coding | |
| MACS_peak_24371 | 562.42 | Intergenic | LINC00290 | ncRNA |  |
| MACS_peak_15306 | 561.26 | intron (NM_023035, intron 31 of 46) | IER2 | protein-coding | |
| MACS_peak_25048 | 561.06 | intron (NM_030955, intron 13 of 23) | TARS | protein-coding | |
| MACS_peak_20213 | 560.64 | Intergenic | MIR124-3 | ncRNA |  |
| MACS_peak_9427 | 560.44 | intron (NM_001145004, intron 1 of 8) | GOLGA6L6 | protein-coding | |
| MACS_peak_91 | 560.19 | intron (NM_017891, intron 4 of 9) | RNF223 | protein-coding | |
| MACS_peak_3471 | 559.82 | Intergenic | CHRM3 | protein-coding | |
| MACS_peak_2217 | 559.23 | promoter-TSS (NR_027354).3 | LSP1P5 | pseudo |  |
| MACS_peak_6119 | 558.8 | exon (NM_004055, exon 5 of 13) | OMP | protein-coding | |
| MACS_peak_174 | 558.48 | intron (NM_031921, intron 11 of 15) | ATAD3B | protein-coding | |
| MACS_peak_19396 | 556.53 | Intergenic | WFDC3 | protein-coding | |
| MACS_peak_4971 | 556.42 | intron (NM_001318878, intron 1 of 11) | STK32C | protein-coding | |
| MACS_peak_288 | 556.01 | Intergenic | PEX10 | protein-coding | |
| MACS_peak_32014 | 555.94 | NA |  |  |  |
| MACS_peak_19825 | 555.72 | Intergenic | C20orf85 | protein-coding | |
| MACS_peak_21996 | 555.42 | intron (NM_001346222, intron 1 of 9) | IL2RB | protein-coding | |
| MACS_peak_20655 | 555.37 | TTS (NM_001356339) | B3GALT5 | protein-coding | |
| MACS_peak_8247 | 555.26 | intron (NM_001846, intron 4 of 47) | MIR8073 | ncRNA |  |
| MACS_peak_12642 | 555.15 | Intergenic | FLJ36000 | ncRNA |  |
| MACS_peak_990 | 554.62 | intron (NR_026567, intron 1 of 10) | ESPNP | pseudo |  |
| MACS_peak_26620 | 554.32 | Intergenic | MIR1273C | ncRNA |  |
| MACS_peak_29275 | 554.2 | intron (NR_110157, intron 2 of 3) | LOC101927914 | ncRNA |  |
| MACS_peak_8320 | 553.02 | Intergenic | LINC01070 | ncRNA |  |
| MACS_peak_8257 | 552.93 | intron (NM_001242882, intron 4 of 9) | NAXD | protein-coding | |
| MACS_peak_21466 | 552.69 | intron (NM_022044, intron 1 of 2) | SDF2L1 | protein-coding | |
| MACS_peak_8871 | 552.26 | intron (NM_130469, intron 1 of 3) | JDP2 | protein-coding | |
| MACS_peak_11214 | 551.17 | Intergenic | SLC7A5P2 | pseudo |  |
| MACS_peak_31011 | 551.1 | Intergenic | MIR4478 | ncRNA |  |
| MACS_peak_3987 | 551.04 | Intergenic | LOC102724488 | protein-coding | |
| MACS_peak_21897 | 550.72 | intron (NR_149072, intron 1 of 3) | SLC5A4-AS1 | ncRNA |  |
| MACS_peak_3775 | 550.09 | Intergenic | ARHGAP12 | protein-coding | |
| MACS_peak_19873 | 549.85 | intron (NR_132272, intron 1 of 3) | GNAS | protein-coding | |
| MACS_peak_16256 | 549.83 | intron (NM_001365103, intron 1 of 5) | LOC390937 | protein-coding | |
| MACS_peak_7716 | 549.4 | Intergenic | LINC02414 | ncRNA |  |
| MACS_peak_677 | 549.24 | Intergenic | SLC45A1 | protein-coding | |
| MACS_peak_21215 | 549.23 | promoter-TSS (NM_053004) | RTL10 | protein-coding | |
| MACS_peak_28251 | 548.8 | TTS (NM_003602) | FKBP6 | protein-coding | |
| MACS_peak_535 | 548.74 | Intergenic | MIR4689 | ncRNA |  |
| MACS_peak_11125 | 548.47 | TTS (NR_106766).3 | MIR6511A3 | ncRNA |  |
| MACS_peak_29098 | 548.39 | Intergenic | ZNF467 | protein-coding | |
| MACS_peak_17613 | 547.83 | Intergenic | HAAO | protein-coding | |
| MACS_peak_20348 | 546.47 | Intergenic | TCEA2 | protein-coding | |
| MACS_peak_218 | 546.38 | 5' UTR (NM_002074, exon 1 of 12) | GNB1 | protein-coding | |
| MACS_peak_12857 | 545.25 | TTS (NM_001291462).9 | TBC1D3G | protein-coding | |
| MACS_peak_28105 | 545.11 | Intergenic | MIR4283-1 | ncRNA |  |
| MACS_peak_19505 | 544.77 | Intergenic | LINC00494 | ncRNA |  |
| MACS_peak_10513 | 544.56 | intron (NM_014700, intron 2 of 13).2 | RAB11FIP3 | protein-coding | |
| MACS_peak_5010 | 543.16 | intron (NM_001200049, intron 54 of 57) | NKX6-2 | protein-coding | |
| MACS_peak_14369 | 542.85 | Intergenic | LINC00470 | ncRNA |  |
| MACS_peak_30346 | 542.84 | intron (NM_174922, intron 1 of 14) | ADCK5 | protein-coding | |
| MACS_peak_8778 | 542.74 | promoter-TSS (NM_001193362) | EXD2 | protein-coding | |
| MACS_peak_21665 | 542.5 | intron (NM_001099781, intron 8 of 11) | GGT5 | protein-coding | |
| MACS_peak_28217 | 542.45 | Intergenic | SPDYE8P | pseudo |  |
| MACS_peak_26737 | 541.72 | Intergenic | DACT2 | protein-coding | |
| MACS_peak_19375 | 540.01 | promoter-TSS (NM_001205317) | RIMS4 | protein-coding | |
| MACS_peak_6708 | 539.65 | exon (NM_001007026, exon 5 of 10) | C12orf57 | protein-coding | |
| MACS_peak_12651 | 538.8 | Intergenic | FLJ36000 | ncRNA |  |
| MACS_peak_23718 | 538.18 | Intergenic | CEP19 | protein-coding | |
| MACS_peak_22680 | 538.14 | promoter-TSS (NM_138711) | PPARG | protein-coding | |
| MACS_peak_28197 | 537.79 | intron (NM_001257190, intron 14 of 15) | NSUN5P2 | pseudo |  |
| MACS_peak_27116 | 537.3 | intron (NM_001040168, intron 1 of 7) | MIR4648 | ncRNA |  |
| MACS_peak_6915 | 537.19 | promoter-TSS (NM_001206917) | CACNB3 | protein-coding | |
| MACS_peak_13653 | 536.81 | Intergenic | KPNA2 | protein-coding | |
| MACS_peak_17412 | 536.71 | intron (NM_194248, intron 13 of 46) | OTOF | protein-coding | |
| MACS_peak_4096 | 535.72 | intron (NM_001282405, intron 1 of 2) | NRBF2 | protein-coding | |
| MACS_peak_30179 | 533.93 | Intergenic | CYP11B2 | protein-coding | |
| MACS_peak_26738 | 533.83 | intron (NM_001166412, intron 4 of 12) | SMOC2 | protein-coding | |
| MACS_peak_31229 | 533.55 | intron (NR_163192, intron 6 of 10) | SH3GLB2 | protein-coding | |
| MACS_peak_18702 | 533.49 | exon (NM_001346120, exon 3 of 4) | SNORC | protein-coding | |
| MACS_peak_7037 | 533.02 | TTS (NR_045962) | MIR9898 | ncRNA |  |
| MACS_peak_2245 | 532.66 | Intergenic | RNVU1-6 | snRNA |  |
| MACS_peak_24646 | 532.61 | intron (NM_004553, intron 3 of 3) | NDUFS6 | protein-coding | |
| MACS_peak_6873 | 532.2 | promoter-TSS (NM_001144881) | PRICKLE1 | protein-coding | |
| MACS_peak_24381 | 531.66 | Intergenic | DUX4 | protein-coding | |
| MACS_peak_16673 | 531.01 | promoter-TSS (NM_001195256) | GFY | protein-coding | |
| MACS_peak_13564 | 530.67 | promoter-TSS (NM_001915) | CYB561 | protein-coding | |
| MACS_peak_10428 | 530.23 | intron (NM_001290341, intron 13 of 13) | CERS3-AS1 | ncRNA |  |
| MACS_peak_4340 | 530.1 | exon (NM_024875, exon 2 of 2) | SYNPO2L | protein-coding | |
| MACS_peak_3581 | 529.94 | promoter-TSS (NR_120629) | LOC101927762 | ncRNA |  |
| MACS_peak_21395 | 529 | exon (NM_001351304, exon 16 of 17) | FAM230B | ncRNA |  |
| MACS_peak_3285 | 528.97 | intron (NM_020247, intron 5 of 14) | COQ8A | protein-coding | |
| MACS_peak_5748 | 528.91 | intron (NM_139075, intron 1 of 24) | TPCN2 | protein-coding | |
| MACS_peak_11065 | 528.61 | promoter-TSS (NR_106766) | MIR6511A2 | ncRNA |  |
| MACS_peak_5893 | 528.44 | intron (NM_012309, intron 11 of 24) | SHANK2-AS3 | ncRNA |  |
| MACS_peak_9048 | 528.43 | intron (NR_146552, intron 1 of 3) | LINC02299 | ncRNA |  |
| MACS_peak_18281 | 528.3 | Intergenic | TFCP2L1 | protein-coding | |
| MACS_peak_16386 | 528.13 | Intergenic | EXOC3L2 | protein-coding | |
| MACS_peak_12645 | 527.63 | Intergenic | FLJ36000 | ncRNA |  |
| MACS_peak_3819 | 527.58 | intron (NR_120656, intron 1 of 4) | LINC01518 | ncRNA |  |
| MACS_peak_31862 | 527.26 | promoter-TSS (NM_017723) | TOR4A | protein-coding | |
| MACS_peak_10221 | 526.93 | intron (NM_001267536, intron 9 of 10) | DNM1P41 | pseudo |  |
| MACS_peak_30622 | 526.71 | TTS (NR_027422) | FAM27B | ncRNA |  |
| MACS_peak_13535 | 526.02 | promoter-TSS (NM_032043) | BRIP1 | protein-coding | |
| MACS_peak_21200 | 525.21 | Intergenic | TBX1 | protein-coding | |
| MACS_peak_7017 | 524.71 | intron (NM_005556, intron 8 of 8) | KRT7-AS | ncRNA |  |
| MACS_peak_21100 | 522.01 | intron (NR_003267, intron 12 of 12) | GGT3P | pseudo |  |
| MACS_peak_20813 | 521.71 | Intergenic | TRPM2 | protein-coding | |
| MACS_peak_19997 | 520.62 | intron (NM_001252339, intron 3 of 14) | LOC100128310 | ncRNA |  |
| MACS_peak_15866 | 519.87 | Intergenic | KCTD15 | protein-coding | |
| MACS_peak_32353 | 519.42 | Intergenic | MAGEA6 | protein-coding | |
| MACS_peak_4908 | 519.04 | Intergenic | LINC01163 | ncRNA |  |
| MACS_peak_8521 | 518.51 | exon (NM_005407, exon 2 of 2) | SALL2 | protein-coding | |
| MACS_peak_16060 | 518.47 | intron (NM_001042723, intron 100 of 104) | LOC105372397 | ncRNA |  |
| MACS_peak_4069 | 518.04 | intron (NM_001347852, intron 4 of 12) | FAM13C | protein-coding | |
| MACS_peak_19847 | 517.73 | intron (NR_034147, intron 6 of 6) | APCDD1L | protein-coding | |
| MACS_peak_24509 | 517.61 | intron (NR_147095, intron 1 of 16) | ZDHHC11B | protein-coding | |
| MACS_peak_12165 | 516.91 | non-coding (NR_036480, exon 1 of 4) | VPS9D1-AS1 | ncRNA |  |
| MACS_peak_144 | 516.5 | promoter-TSS (NM_001029885) | INTS11 | protein-coding | |
| MACS_peak_26993 | 516.38 | intron (NM_001097620, intron 2 of 8) | TMEM184A | protein-coding | |
| MACS_peak_3596 | 515.92 | intron (NM_018702, intron 3 of 9) | LINC00200 | ncRNA |  |
| MACS_peak_8221 | 515.79 | Intergenic | LINC01309 | ncRNA |  |
| MACS_peak_21569 | 515.55 | intron (NR_037839, intron 1 of 6) | CES5AP1 | pseudo |  |
| MACS_peak_14281 | 515.46 | Intergenic | UTS2R | protein-coding | |
| MACS_peak_31976 | 515.16 | Intergenic | MIR6724-4 | ncRNA |  |
| MACS_peak_20206 | 515.06 | Intergenic | MIR124-3 | ncRNA |  |
| MACS_peak_5754 | 514.88 | intron (NM_139075, intron 23 of 24) | MIR3164 | ncRNA |  |
| MACS_peak_10559 | 514.51 | Intergenic | RPUSD1 | protein-coding | |
| MACS_peak_31827 | 513.96 | intron (NM_212533, intron 10 of 48) | C9orf139 | protein-coding | |
| MACS_peak_14272 | 513.76 | Intergenic | CD7 | protein-coding | |
| MACS_peak_27870 | 513.71 | intron (NM_001281768, intron 8 of 9) | ADCY1 | protein-coding | |
| MACS_peak_32453 | 513.59 | Intergenic | GYG2P1 | pseudo |  |
| MACS_peak_31063 | 513.4 | exon (NM_001144877, exon 1 of 18) | SCAI | protein-coding | |
| MACS_peak_15208 | 513.02 | TTS (NR_104024) | MIR1238 | ncRNA |  |
| MACS_peak_20060 | 512.74 | promoter-TSS (NM_175573) | ADRM1 | protein-coding | |
| MACS_peak_24980 | 512.59 | intron (NR_027028, intron 4 of 5).2 | GUSBP1 | pseudo |  |
| MACS_peak_29 | 511.74 | Intergenic | LOC101928626 | ncRNA |  |
| MACS_peak_97 | 511.69 | Intergenic | C1orf159 | protein-coding | |
| MACS_peak_31465 | 511.13 | promoter-TSS (NM_003172) | SURF2 | protein-coding | |
| MACS_peak_2277 | 510.95 | Intergenic | LOC653513 | pseudo |  |
| MACS_peak_3621 | 510.66 | Intergenic | LOC101927824 | ncRNA |  |
| MACS_peak_5974 | 509.92 | intron (NM_001040118, intron 1 of 34) | ARAP1 | protein-coding | |
| MACS_peak_21585 | 509.85 | Intergenic | PCAT14 | ncRNA |  |
| MACS_peak_24386 | 509.84 | Intergenic | DUX4 | protein-coding | |
| MACS_peak_4286 | 509.46 | 3' UTR (NM_001168390, exon 2 of 2) | C10orf105 | protein-coding | |
| MACS_peak_25280 | 509.43 | intron (NM_005575, intron 1 of 17) | LNPEP | protein-coding | |
| MACS_peak_21682 | 509.2 | intron (NM_001284256, intron 4 of 4) | GUCD1 | protein-coding | |
| MACS_peak_18843 | 508.76 | Intergenic | KIF1A | protein-coding | |
| MACS_peak_1744 | 508.61 | promoter-TSS (NM_148905) | OSBPL9 | protein-coding | |
| MACS_peak_5009 | 508.26 | Intergenic | NKX6-2 | protein-coding | |
| MACS_peak_32039 | 507.98 | Intergenic | PLCXD1 | protein-coding | |
| MACS_peak_9296 | 507.83 | intron (NM_001242789, intron 1 of 12) | BRF1 | protein-coding | |
| MACS_peak_30185 | 506.77 | promoter-TSS (NM_002346) | LY6E-DT | ncRNA |  |
| MACS_peak_31170 | 506.7 | 3' UTR (NM_203305, exon 8 of 8) | DPM2 | protein-coding | |
| MACS_peak_26749 | 505.81 | intron (NM_003247, intron 10 of 22) | THBS2 | protein-coding | |
| MACS_peak_8202 | 505.69 | promoter-TSS (NM_033110) | GGACT | protein-coding | |
| MACS_peak_20357 | 505.57 | intron (NM_001200019, intron 2 of 5) | OPRL1 | protein-coding | |
| MACS_peak_8330 | 505.57 | Intergenic | SPACA7 | protein-coding | |
| MACS_peak_2908 | 505.47 | Intergenic | LHX9 | protein-coding | |
| MACS_peak_10432 | 505.43 | Intergenic | ASB7 | protein-coding | |
| MACS_peak_6125 | 505.4 | intron (NM_001127180, intron 31 of 48) | MYO7A | protein-coding | |
| MACS_peak_24689 | 505.13 | Intergenic | LSINCT5 | ncRNA |  |
| MACS_peak_29012 | 505.12 | Intergenic | KDM7A-DT | ncRNA |  |
| MACS_peak_15885 | 504.91 | intron (NR_027620, intron 1 of 3) | SCGB2B2 | protein-coding | |
| MACS_peak_30835 | 504.55 | Intergenic | ABCA1 | protein-coding | |
| MACS_peak_17135 | 504.44 | Intergenic | PXDN | protein-coding | |
| MACS_peak_27794 | 503.7 | intron (NM_172084, intron 12 of 15) | YKT6 | protein-coding | |
| MACS_peak_21135 | 503.34 | intron (NR_110533, intron 5 of 5) | DGCR10 | ncRNA |  |
| MACS_peak_19875 | 502.95 | intron (NM_001336, intron 2 of 5) | CTSZ | protein-coding | |
| MACS_peak_32048 | 502.69 | intron (NM_002183, intron 9 of 11) | SLC25A6 | protein-coding | |
| MACS_peak_7938 | 502.6 | promoter-TSS (NR_046414) | RB1 | protein-coding | |
| MACS_peak_12221 | 502.56 | intron (NM_001322841, intron 1 of 22) | ABR | protein-coding | |
| MACS_peak_14485 | 501.79 | Intergenic | ANKRD62 | protein-coding | |
| MACS_peak_31483 | 501.26 | intron (NM_001145320, intron 9 of 18) | ADAMTSL2 | protein-coding | |
| MACS_peak_20634 | 501.25 | intron (NM_001256295, intron 2 of 10) | ETS2 | protein-coding | |
| MACS_peak_120 | 501.22 | intron (NM_004195, intron 1 of 4) | TNFRSF18 | protein-coding | |
| MACS_peak_15332 | 500.95 | intron (NM_001145028, intron 2 of 6) | PALM3 | protein-coding | |
| MACS_peak_131 | 500.87 | Intergenic | C1QTNF12 | protein-coding | |
| MACS_peak_24564 | 500.57 | Intergenic | SLC12A7 | protein-coding | |
| MACS_peak_14895 | 500.5 | intron (NM_002067, intron 4 of 6) | GNA15 | protein-coding | |
| MACS_peak_26773 | 500.41 | intron (NR_002787, intron 2 of 2) | LOC154449 | ncRNA |  |
| MACS_peak_24011 | 500.25 | intron (NM_020777, intron 2 of 26) | MIR4274 | ncRNA |  |
| MACS_peak_29345 | 500.16 | intron (NR_130758, intron 4 of 12) | VIPR2 | protein-coding | |
| MACS_peak_10859 | 499.41 | intron (NM_001351729, intron 7 of 27) | VASN | protein-coding | |
| MACS_peak_31355 | 498.95 | promoter-TSS (NM_198180) | QRFP | protein-coding | |
| MACS_peak_31975 | 498.55 | Intergenic | RNA28SN5 | rRNA |  |
| MACS_peak_32008 | 498.25 | NA |  |  |  |
| MACS_peak_30694 | 497.58 | intron (NR_163886, intron 13 of 15) | SECISBP2 | protein-coding | |
| MACS_peak_1592 | 497.33 | 3' UTR (NM_173642, exon 5 of 5) | ZMYND12 | protein-coding | |
| MACS_peak_9558 | 496.52 | intron (NM_001353794, intron 3 of 12).3 | APBA2 | protein-coding | |
| MACS_peak_31486 | 496.23 | intron (NM_001145320, intron 17 of 18) | FAM163B | protein-coding | |
| MACS_peak_29322 | 496.12 | intron (NM_130842, intron 1 of 21) | MIR595 | ncRNA |  |
| MACS_peak_12918 | 495.99 | promoter-TSS (NM_001291466) | TBC1D3C | protein-coding | |
| MACS_peak_9176 | 495.57 | promoter-TSS (NM_001100118) | |  |  |
| MACS_peak_7464 | 495.36 | intron (NM_001080825, intron 10 of 11) | RHOF | protein-coding | |
| MACS_peak_13303 | 494.96 | promoter-TSS (NM_001145023) | SCRN2 | protein-coding | |
| MACS_peak_8771 | 494.96 | intron (NM_001102, intron 8 of 20) | ACTN1 | protein-coding | |
| MACS_peak_50 | 494.68 | intron (NM_152486, intron 8 of 13) | SAMD11 | protein-coding | |
| MACS_peak_12297 | 494.61 | intron (NM_001330058, intron 23 of 24) | OR1D5 | protein-coding | |
| MACS_peak_292 | 493.46 | Intergenic | PLCH2 | protein-coding | |
| MACS_peak_4079 | 493.15 | intron (NM_033379, intron 1 of 6) | CDK1 | protein-coding | |
| MACS_peak_24488 | 493.06 | intron (NM_007030, intron 2 of 3) | TPPP | protein-coding | |
| MACS_peak_14690 | 492.93 | intron (NM_001700, intron 2 of 4) | AZU1 | protein-coding | |
| MACS_peak_27363 | 492.91 | Intergenic | CCZ1B | protein-coding | |
| MACS_peak_168 | 492.48 | exon (NM_001039211, exon 7 of 12) | ATAD3C | protein-coding | |
| MACS_peak_2181 | 492.43 | promoter-TSS (NR_027354) | LSP1P5 | pseudo |  |
| MACS_peak_19266 | 492.4 | intron (NM_198291, intron 1 of 13) | SRC | protein-coding | |
| MACS_peak_18027 | 491.47 | intron (NR_160767, intron 1 of 3) | LOC107985911 | pseudo |  |
| MACS_peak_21301 | 491.14 | Intergenic | DGCR6L | protein-coding | |
| MACS_peak_2709 | 491.03 | Intergenic | FCGR2A | protein-coding | |
| MACS_peak_20220 | 490.94 | intron (NM_001363747, intron 3 of 6) | NKAIN4 | protein-coding | |
| MACS_peak_31554 | 490.65 | intron (NM_002957, intron 1 of 9) | RXRA | protein-coding | |
| MACS_peak_20169 | 490.16 | Intergenic | SNORA117 | snoRNA |  |
| MACS_peak_31988 | 490.13 | NA |  |  |  |
| MACS_peak_29924 | 490.1 | Intergenic | ADCY8 | protein-coding | |
| MACS_peak_30141 | 489.84 | intron (NM_001702, intron 7 of 30) | ADGRB1 | protein-coding | |
| MACS_peak_10500 | 489.77 | intron (NM_003502, intron 2 of 10) | AXIN1 | protein-coding | |
| MACS_peak_5035 | 489.34 | Intergenic | ADGRA1-AS1 | ncRNA |  |
| MACS_peak_5378 | 488.99 | Intergenic | MS4A10 | protein-coding | |
| MACS_peak_26329 | 488.86 | intron (NM_014780, intron 6 of 25) | CUL7 | protein-coding | |
| MACS_peak_24879 | 488.59 | intron (NM_001288715, intron 8 of 20) | CTNND2 | protein-coding | |
| MACS_peak_211 | 488.38 | intron (NM_001199787, intron 1 of 6) | SLC35E2A | protein-coding | |
| MACS_peak_15563 | 488.09 | intron (NM_015016, intron 2 of 26) | IL12RB1 | protein-coding | |
| MACS_peak_12864 | 487.93 | TTS (NM_032258).3 | TBC1D3K | protein-coding | |
| MACS_peak_29722 | 487.66 | intron (NM_014393, intron 1 of 11) | STAU2 | protein-coding | |
| MACS_peak_16718 | 486.76 | TTS (NR_106857) | MIR6799 | ncRNA |  |
| MACS_peak_26952 | 486.61 | Intergenic | UNCX | protein-coding | |
| MACS_peak_2668 | 486.58 | promoter-TSS (NM_001349729) | NCSTN | protein-coding | |
| MACS_peak_12896 | 486.55 | TTS (NM_001291464).2 | TBC1D3K | protein-coding | |
| MACS_peak_16597 | 486.49 | 3' UTR (NM_001080434, exon 16 of 16) | CYTH2 | protein-coding | |
| MACS_peak_29070 | 486.38 | exon (NM_005435, exon 8 of 15) | ARHGEF34P | pseudo |  |
| MACS_peak_15760 | 486.19 | promoter-TSS (NM_006627) | POP4 | protein-coding | |
| MACS_peak_14363 | 486 | intron (NR_136505, intron 2 of 2) | LINC01925 | ncRNA |  |
| MACS_peak_28495 | 485.67 | promoter-TSS (NM_001350121) | SEMA3C | protein-coding | |
| MACS_peak_10893 | 485.67 | intron (NM_002705, intron 1 of 21) | PPL | protein-coding | |
| MACS_peak_11170 | 485.53 | Intergenic | MIR3179-2 | ncRNA |  |
| MACS_peak_2423 | 485.3 | intron (NM_014856, intron 4 of 27) | DENND4B | protein-coding | |
| MACS_peak_17816 | 485.22 | Intergenic | ETAA1 | protein-coding | |
| MACS_peak_7550 | 485.08 | intron (NM_001365156, intron 1 of 2) | RFLNA | protein-coding | |
| MACS_peak_10675 | 484.92 | intron (NM_020825, intron 10 of 19) | JPT2 | protein-coding | |
| MACS_peak_31907 | 484.65 | intron (NM_001354263, intron 2 of 26) | LOC651337 | ncRNA |  |
| MACS_peak_11168 | 484.59 | Intergenic | MIR3179-1 | ncRNA |  |
| MACS_peak_21463 | 484.03 | intron (NM_001256355, intron 3 of 3) | |  |  |
| MACS_peak_29184 | 483.75 | Intergenic | ACTR3B | protein-coding | |
| MACS_peak_3311 | 483.74 | Intergenic | MIR5008 | ncRNA |  |
| MACS_peak_2367 | 483.67 | exon (NM_001025231, exon 2 of 2) | KPRP | protein-coding | |
| MACS_peak_20349 | 483.51 | promoter-TSS (NM_198723) | TCEA2 | protein-coding | |
| MACS_peak_28646 | 483.49 | TTS (NM_016188) | TFR2 | protein-coding | |
| MACS_peak_22038 | 483.41 | promoter-TSS (NM_001350055) | SH3BP1 | protein-coding | |
| MACS_peak_17143 | 482.65 | Intergenic | PXDN | protein-coding | |
| MACS_peak_27048 | 482.37 | intron (NM_001013837, intron 17 of 18) | MAD1L1 | protein-coding | |
| MACS_peak_29992 | 482.24 | intron (NM_001160372, intron 21 of 22) | KCNK9 | protein-coding | |
| MACS_peak_11163 | 482.11 | non-coding (NR_146331, exon 15 of 38) | MIR6770-1 | ncRNA |  |
| MACS_peak_6375 | 481.91 | intron (NM_001144759, intron 1 of 20) | PHLDB1 | protein-coding | |
| MACS_peak_11062 | 481.5 | Intergenic | MIR3179-1 | ncRNA |  |
| MACS_peak_397 | 481.26 | intron (NM_022114, intron 2 of 16) | MIR4251 | ncRNA |  |
| MACS_peak_32392 | 480.93 | intron (NM_005393, intron 2 of 35) | PLXNB3 | protein-coding | |
| MACS_peak_35 | 480.83 | intron (NR_033908, intron 1 of 6) | LOC100288069 | ncRNA |  |
| MACS_peak_26199 | 480.53 | intron (NM_015695, intron 1 of 12) | BRPF3 | protein-coding | |
| MACS_peak_24791 | 479.9 | Intergenic | LINC02102 | ncRNA |  |
| MACS_peak_25401 | 478.95 | exon (NM_001194956, exon 1 of 14) | MATR3 | protein-coding | |
| MACS_peak_16167 | 478.88 | intron (NM_013376, intron 1 of 1) | SERTAD1 | protein-coding | |
| MACS_peak_19791 | 478.64 | intron (NM_199171, intron 1 of 3) | PMEPA1 | protein-coding | |
| MACS_peak_494 | 478.46 | Intergenic | LINC01777 | ncRNA |  |
| MACS_peak_26267 | 478.11 | Intergenic | MOCS1 | protein-coding | |
| MACS_peak_18187 | 477.93 | non-coding (NR_046110, exon 2 of 4) | LINC01123 | ncRNA |  |
| MACS_peak_31676 | 477.73 | Intergenic | UBAC1 | protein-coding | |
| MACS_peak_6682 | 477.48 | intron (NM_016162, intron 1 of 7) | ING4 | protein-coding | |
| MACS_peak_24117 | 477.45 | Intergenic | TLR10 | protein-coding | |
| MACS_peak_7570 | 476.75 | intron (NM_001077261, intron 8 of 47) | MIR6880 | ncRNA |  |
| MACS_peak_21641 | 476.65 | promoter-TSS (NM_001355).3 | GSTT2B | protein-coding | |
| MACS_peak_10608 | 476.46 | Intergenic | C1QTNF8 | protein-coding | |
| MACS_peak_12164 | 476.35 | exon (NM_152339, exon 2 of 3) | SPATA2L | protein-coding | |
| MACS_peak_11851 | 476.26 | Intergenic | MIR5093 | ncRNA |  |
| MACS_peak_1534 | 475.81 | intron (NM_032526, intron 5 of 5) | NT5C1A | protein-coding | |
| MACS_peak_12741 | 475.68 | Intergenic | GIT1 | protein-coding | |
| MACS_peak_744 | 475.39 | exon (NM_001350235, exon 17 of 23) | PIK3CD | protein-coding | |
| MACS_peak_21959 | 475.27 | promoter-TSS (NM_012473) | TXN2 | protein-coding | |
| MACS_peak_31799 | 475.26 | promoter-TSS (NM_001135861) | PHPT1 | protein-coding | |
| MACS_peak_28246 | 474.87 | Intergenic | NSUN5 | protein-coding | |
| MACS_peak_14682 | 474.19 | intron (NM_002579, intron 6 of 8) | MISP | protein-coding | |
| MACS_peak_12505 | 473.75 | promoter-TSS (NM_001199989) | RASD1 | protein-coding | |
| MACS_peak_9477 | 473.56 | intron (NM_001365371, intron 10 of 12) | GOLGA6L7 | protein-coding | |
| MACS_peak_10604 | 473.21 | Intergenic | C1QTNF8 | protein-coding | |
| MACS_peak_20981 | 472.84 | intron (NR_132385, intron 2 of 2) | LINC01297 | ncRNA |  |
| MACS_peak_29068 | 472.77 | non-coding (NR_033942, exon 7 of 13) | CTAGE8 | protein-coding | |
| MACS_peak_22390 | 472.75 | promoter-TSS (NR_104292) | TBC1D22A | protein-coding | |
| MACS_peak_30360 | 471.93 | promoter-TSS (NM_138496) | CYHR1 | protein-coding | |
| MACS_peak_14896 | 471.92 | intron (NM_002067, intron 6 of 6) | GNA15 | protein-coding | |
| MACS_peak_3194 | 471.9 | intron (NM_016343, intron 1 of 19) | CENPF | protein-coding | |
| MACS_peak_1210 | 471.35 | promoter-TSS (NM_000403) | GALE | protein-coding | |
| MACS_peak_1906 | 471.19 | exon (NM_001199742, exon 2 of 3) | GADD45A | protein-coding | |
| MACS_peak_17131 | 470.98 | intron (NM_175722, intron 12 of 14) | TPO | protein-coding | |
| MACS_peak_6930 | 470.95 | exon (NM_001008223, exon 2 of 2) | C1QL4 | protein-coding | |
| MACS_peak_29490 | 470.66 | intron (NR_047662, intron 6 of 8) | LOC729732 | ncRNA |  |
| MACS_peak_20157 | 470.61 | Intergenic | LINC00659 | ncRNA |  |
| MACS_peak_17577 | 470.6 | promoter-TSS (NM_005633) | SOS1 | protein-coding | |
| MACS_peak_13994 | 470.55 | intron (NM_001082575, intron 2 of 14) | RBFOX3 | protein-coding | |
| MACS_peak_156 | 470.36 | TTS (NM_017900) | AURKAIP1 | protein-coding | |
| MACS_peak_14300 | 470.24 | intron (NM_006822, intron 1 of 5) | RAB40B | protein-coding | |
| MACS_peak_31855 | 470.18 | intron (NM_001177316, intron 12 of 12) | SLC34A3 | protein-coding | |
| MACS_peak_19526 | 470.14 | promoter-TSS (NR_036659) | ZNFX1 | protein-coding | |
| MACS_peak_11099 | 469.65 | intron (NM_001040114, intron 1 of 41).2 | MYH11 | protein-coding | |
| MACS_peak_29246 | 469.46 | Intergenic | LOC389602 | protein-coding | |
| MACS_peak_12936 | 469.41 | intron (NM_025248, intron 1 of 18) | SRCIN1 | protein-coding | |
| MACS_peak_12422 | 468.79 | intron (NM_001080424, intron 1 of 21) | KDM6B | protein-coding | |
| MACS_peak_8779 | 468.27 | intron (NM_020692, intron 1 of 15) | GALNT16 | protein-coding | |
| MACS_peak_21997 | 467.9 | intron (NM_001346222, intron 1 of 9) | IL2RB | protein-coding | |
| MACS_peak_12485 | 467.82 | promoter-TSS (NR_027179) | SNHG29 | ncRNA |  |
| MACS_peak_23395 | 467.39 | intron (NM_003571, intron 1 of 6) | BFSP2 | protein-coding | |
| MACS_peak_15907 | 466.72 | promoter-TSS (NM_001320912) | FXYD5 | protein-coding | |
| MACS_peak_16199 | 466.49 | intron (NM_016154, intron 5 of 7) | RAB4B | protein-coding | |
| MACS_peak_24754 | 466.41 | intron (NM_139056, intron 4 of 22) | ADAMTS16 | protein-coding | |
| MACS_peak_13608 | 465.95 | intron (NR_024386, intron 8 of 10) | ARHGAP27P1-BPTFP1-KPNA2P3 | pseudo |  |
| MACS_peak_19465 | 465.89 | intron (NM_198596, intron 5 of 20) | SULF2 | protein-coding | |
| MACS_peak_18666 | 465.64 | 3' UTR (NM_145236, exon 2 of 2) | B3GNT7 | protein-coding | |
| MACS_peak_21075 | 465.4 | Intergenic | PI4KAP1 | pseudo |  |
| MACS_peak_29388 | 464.18 | intron (NM_001346810, intron 4 of 14) | DLGAP2-AS1 | ncRNA |  |
| MACS_peak_17109 | 463.97 | Intergenic | MIR3675 | ncRNA |  |
| MACS_peak_4349 | 463.93 | intron (NR_160045, intron 12 of 17) | NDST2 | protein-coding | |
| MACS_peak_31510 | 463.75 | intron (NM_001134398, intron 13 of 29) | SARDH | protein-coding | |
| MACS_peak_5001 | 463.58 | intron (NM_005539, intron 4 of 15) | INPP5A | protein-coding | |
| MACS_peak_25934 | 463.23 | Intergenic | LINC00581 | ncRNA |  |
| MACS_peak_31710 | 463.09 | Intergenic | QSOX2 | protein-coding | |
| MACS_peak_8037 | 462.75 | intron (NM_022843, intron 1 of 1) | PCDH20 | protein-coding | |
| MACS_peak_15008 | 462.6 | promoter-TSS (NM_015015) | KDM4B | protein-coding | |
| MACS_peak_24387 | 462.46 | Intergenic | DUX4 | protein-coding | |
| MACS_peak_20894 | 461.37 | intron (NM_130445, intron 32 of 42) | MIR6815 | ncRNA |  |
| MACS_peak_21661 | 460.93 | TTS (NM_001199281) | SUSD2 | protein-coding | |
| MACS_peak_28153 | 460.59 | intron (NM_001367749, intron 1 of 4) | RABGEF1 | protein-coding | |
| MACS_peak_14622 | 460.57 | Intergenic | LINC01029 | ncRNA |  |
| MACS_peak_22727 | 460.19 | Intergenic | NUP210 | protein-coding | |
| MACS_peak_24582 | 459.78 | intron (NM_198253, intron 13 of 15) | SLC6A18 | protein-coding | |
| MACS_peak_25657 | 459.62 | intron (NR_003615, intron 1 of 5) | LOC728554 | pseudo |  |
| MACS_peak_15937 | 459.17 | exon (NM_001367856, exon 9 of 11) | ARHGAP33 | protein-coding | |
| MACS_peak_19935 | 458.94 | Intergenic | LINC01718 | ncRNA |  |
| MACS_peak_28757 | 458.91 | intron (NM_001097615, intron 8 of 8) | UPK3BL2 | protein-coding | |
| MACS_peak_15659 | 458.78 | promoter-TSS (NM_005919) | RFXANK | protein-coding | |
| MACS_peak_17105 | 458.46 | Intergenic | MIR3675 | ncRNA |  |
| MACS_peak_29330 | 458.11 | intron (NM_130843, intron 1 of 21) | PTPRN2 | protein-coding | |
| MACS_peak_16729 | 458.06 | intron (NM_007254, intron 3 of 16) | PNKP | protein-coding | |
| MACS_peak_28864 | 457.45 | Intergenic |  |  |  |
| MACS_peak_31839 | 457.43 | intron (NM_021569, intron 3 of 18) | GRIN1 | protein-coding | |
| MACS_peak_5059 | 456.93 | Intergenic | CALY | protein-coding | |
| MACS_peak_30610 | 456.43 | exon (NM_001083124, exon 4 of 4) | SPATA31A3 | protein-coding | |
| MACS_peak_4896 | 456.37 | intron (NM_001290223, intron 47 of 51) | NPS | protein-coding | |
| MACS_peak_151 | 456.19 | intron (NM_004421, intron 1 of 14) | DVL1 | protein-coding | |
| MACS_peak_28011 | 456.14 | Intergenic | FKBP9P1 | pseudo |  |
| MACS_peak_20186 | 455.89 | Intergenic | LINC01749 | ncRNA |  |
| MACS_peak_6660 | 455.83 | Intergenic | PLEKHG6 | protein-coding | |
| MACS_peak_13709 | 455.76 | intron (NM_001144952, intron 1 of 44) | SDK2 | protein-coding | |
| MACS_peak_22122 | 455.67 | Intergenic | PDGFB | protein-coding | |
| MACS_peak_31918 | 455.56 | Intergenic | MIR602 | ncRNA |  |
| MACS_peak_8777 | 454.98 | promoter-TSS (NM_001284207) | DCAF5 | protein-coding | |
| MACS_peak_12097 | 454.92 | Intergenic | CBFA2T3 | protein-coding | |
| MACS_peak_27301 | 454.68 | Intergenic | ACTB | protein-coding | |
| MACS_peak_24515 | 454.57 | intron (NM_024786, intron 1 of 12) | ZDHHC11 | protein-coding | |
| MACS_peak_31221 | 454.43 | promoter-TSS (NM_001127244) | LRRC8A | protein-coding | |
| MACS_peak_29028 | 454.04 | intron (NM_001195278, intron 2 of 3) | AGK | protein-coding | |
| MACS_peak_6074 | 453.98 | promoter-TSS (NM_001253891) | LOC283214 | ncRNA |  |
| MACS_peak_30110 | 453.72 | intron (NM_001366901, intron 13 of 13) | LINC00051 | ncRNA |  |
| MACS_peak_21826 | 453.53 | intron (NM_001318129, intron 4 of 5) | CABP7 | protein-coding | |
| MACS_peak_20160 | 453.51 | Intergenic | LINC00659 | ncRNA |  |
| MACS_peak_25757 | 453.46 | Intergenic | FOXQ1 | protein-coding | |
| MACS_peak_23530 | 453.44 | Intergenic | NAALADL2-AS1 | ncRNA |  |
| MACS_peak_79 | 452.84 | intron (NM_198576, intron 7 of 35) | AGRN | protein-coding | |
| MACS_peak_12963 | 452.53 | intron (NM_199248, intron 8 of 12) | CACNB1 | protein-coding | |
| MACS_peak_21698 | 452.53 | intron (NR_024593, intron 4 of 5) | POM121L10P | pseudo |  |
| MACS_peak_29277 | 452.51 | intron (NM_130843, intron 20 of 21) | MIR153-2 | ncRNA |  |
| MACS_peak_28262 | 452.43 | intron (NM_001077621, intron 1 of 3) | VPS37D | protein-coding | |
| MACS_peak_3082 | 452.16 | intron (NM_212503, intron 8 of 15) | CDK18 | protein-coding | |
| MACS_peak_12749 | 452.13 | Intergenic | SLC6A4 | protein-coding | |
| MACS_peak_21124 | 452.08 | Intergenic | DGCR5 | ncRNA |  |
| MACS_peak_25752 | 451.59 | promoter-TSS (NR_073064) | EXOC2 | protein-coding | |
| MACS_peak_14234 | 451.18 | intron (NM_178493, intron 8 of 10) | NOTUM | protein-coding | |
| MACS_peak_105 | 451.08 | Intergenic | LINC01342 | ncRNA |  |
| MACS_peak_21449 | 450.89 | intron (NR_003700, intron 1 of 16) | PI4KAP2 | pseudo |  |
| MACS_peak_31012 | 450.66 | promoter-TSS (NM_198469) | NDUFA8 | protein-coding | |
| MACS_peak_22705 | 450.51 | intron (NM_001134382, intron 1 of 13) | IQSEC1 | protein-coding | |
| MACS_peak_17315 | 450.23 | Intergenic | HS1BP3-IT1 | ncRNA |  |
| MACS_peak_31981 | 450.21 | Intergenic | LOC100505874 | ncRNA |  |
| MACS_peak_11066 | 450.04 | TTS (NR_106766) | LOC100288162 | ncRNA |  |
| MACS_peak_21199 | 449.82 | Intergenic | TBX1 | protein-coding | |
| MACS_peak_23768 | 449.63 | NA |  |  |  |
| MACS_peak_16716 | 449.53 | Intergenic | AP2A1 | protein-coding | |
| MACS_peak_29003 | 449.23 | 3' UTR (NM_001353368, exon 4 of 4) | CLEC2L | protein-coding | |
| MACS_peak_2540 | 448.86 | intron (NM_001304342, intron 1 of 10) | UBQLN4 | protein-coding | |
| MACS_peak_556 | 448.86 | intron (NM_001199862, intron 2 of 15) | KCNAB2 | protein-coding | |
| MACS_peak_158 | 448.66 | promoter-TSS (NM_001350497) | CCNL2 | protein-coding | |
| MACS_peak_31849 | 448.49 | intron (NM_001128228, intron 1 of 3) | SSNA1 | protein-coding | |
| MACS_peak_23792 | 448.38 | intron (NM_001317836, intron 9 of 11) | LOC100129917 | ncRNA |  |
| MACS_peak_22771 | 448.33 | Intergenic | WNT7A | protein-coding | |
| MACS_peak_27134 | 448.24 | intron (NM_025250, intron 1 of 13) | TTYH3 | protein-coding | |
| MACS_peak_89 | 447.75 | TTS (NM_001330306) | RNF223 | protein-coding | |
| MACS_peak_10497 | 447.05 | promoter-TSS (NM_006849) | PDIA2 | protein-coding | |
| MACS_peak_12900 | 446.99 | intron (NM_001369503, intron 2 of 13).2 | TBC1D3I | protein-coding | |
| MACS_peak_12043 | 446.98 | Intergenic | IL17C | protein-coding | |
| MACS_peak_17698 | 446.87 | 5' UTR (NM_001288955, exon 1 of 19) | TTC7A | protein-coding | |
| MACS_peak_75 | 446.26 | intron (NM_198576, intron 2 of 35) | AGRN | protein-coding | |
| MACS_peak_665 | 445.94 | Intergenic | UTS2 | protein-coding | |
| MACS_peak_14865 | 445.64 | intron (NM_052847, intron 3 of 4) | GADD45B | protein-coding | |
| MACS_peak_4145 | 445.46 | Intergenic | NEUROG3 | protein-coding | |
| MACS_peak_24649 | 445.44 | Intergenic | LINC02116 | ncRNA |  |
| MACS_peak_8400 | 445.35 | TTS (NM_138430) | LOC101928841 | protein-coding | |
| MACS_peak_9489 | 445.25 | intron (NM_001001413, intron 1 of 8) | GOLGA6L1 | protein-coding | |
| MACS_peak_19556 | 444.99 | intron (NM_005985, intron 1 of 2) | SNAI1 | protein-coding | |
| MACS_peak_27657 | 444.68 | Intergenic | SEPTIN7 | protein-coding | |
| MACS_peak_21385 | 444.56 | Intergenic | BCRP2 | pseudo |  |
| MACS_peak_8470 | 444.13 | Intergenic | LINC01054 | ncRNA |  |
| MACS_peak_3422 | 443.22 | Intergenic | LINC01354 | ncRNA |  |
| MACS_peak_4290 | 443.21 | exon (NM_022153, exon 6 of 7) | C10orf105 | protein-coding | |
| MACS_peak_27124 | 443.21 | intron (NM_152743, intron 1 of 13) | BRAT1 | protein-coding | |
| MACS_peak_22425 | 443.05 | Intergenic | LINC01310 | ncRNA |  |
| MACS_peak_21190 | 442.56 | Intergenic | SEPTIN5 | protein-coding | |
| MACS_peak_31894 | 442.51 | intron (NM_001098537, intron 8 of 34) | PNPLA7 | protein-coding | |
| MACS_peak_15382 | 442.46 | intron (NM_006844, intron 1 of 15) | ILVBL | protein-coding | |
| MACS_peak_5966 | 441.81 | intron (NR_161388, intron 28 of 29) | PDE2A | protein-coding | |
| MACS_peak_19088 | 441.49 | intron (NM_001363731, intron 1 of 17) | TM9SF4 | protein-coding | |
| MACS_peak_19736 | 441 | Intergenic | BMP7-AS1 | ncRNA |  |
| MACS_peak_6061 | 440.94 | intron (NM_030792, intron 1 of 16) | GDPD5 | protein-coding | |
| MACS_peak_19472 | 440.77 | Intergenic | SULF2 | protein-coding | |
| MACS_peak_17155 | 440.7 | Intergenic | LINC01250 | ncRNA |  |
| MACS_peak_21252 | 440.54 | intron (NM_022720, intron 3 of 13) | MIR1306 | ncRNA |  |
| MACS_peak_21439 | 440.16 | intron (NR_003700, intron 16 of 16) | TMEM191C | protein-coding | |
| MACS_peak_29742 | 440.01 | Intergenic | REXO1L2P | pseudo |  |
| MACS_peak_31253 | 439.93 | Intergenic | C9orf106 | ncRNA |  |
| MACS_peak_225 | 439.8 | TTS (NM_001304360) | TMEM52 | protein-coding | |
| MACS_peak_29326 | 439.73 | intron (NM_130842, intron 1 of 21) | MIR595 | ncRNA |  |
| MACS_peak_11991 | 439.55 | Intergenic | LOC400553 | ncRNA |  |
| MACS_peak_21334 | 439.36 | Intergenic | POM121L4P | pseudo |  |
| MACS_peak_14147 | 438.98 | Intergenic | NDUFAF8 | protein-coding | |
| MACS_peak_14846 | 438.98 | Intergenic | PEAK3 | protein-coding | |
| MACS_peak_27804 | 438.73 | intron (NM_172081, intron 2 of 18) | CAMK2B | protein-coding | |
| MACS_peak_6291 | 438.57 | Intergenic | MMP8 | protein-coding | |
| MACS_peak_20298 | 438.56 | intron (NR_037882, intron 6 of 37) | RTEL1 | protein-coding | |
| MACS_peak_20613 | 438.53 | intron (NR_110418, intron 1 of 5) | LOC101928269 | ncRNA |  |
| MACS_peak_20145 | 437.81 | TTS (NM_016354) | SLCO4A1-AS1 | ncRNA |  |
| MACS_peak_25786 | 437.4 | intron (NM_004332, intron 6 of 6) | TUBB2A | protein-coding | |
| MACS_peak_3981 | 437.04 | Intergenic | FAM25G | protein-coding | |
| MACS_peak_20313 | 436.86 | intron (NM_001305655, intron 1 of 5) | LIME1 | protein-coding | |
| MACS_peak_15471 | 436.76 | promoter-TSS (NR_147452) | CPAMD8 | protein-coding | |
| MACS_peak_11339 | 436.72 | promoter-TSS (NM_001243333) | SEZ6L2 | protein-coding | |
| MACS_peak_85 | 436.69 | intron (NR_148960, intron 3 of 3) | LOC100288175 | ncRNA |  |
| MACS_peak_20491 | 436.61 | Intergenic | TEKT4P2 | pseudo |  |
| MACS_peak_15176 | 436.25 | intron (NM_015719, intron 54 of 66) | OLFM2 | protein-coding | |
| MACS_peak_6104 | 436.03 | Intergenic | TSKU | protein-coding | |
| MACS_peak_2202 | 435.85 | promoter-TSS (NR_027354).2 | LSP1P5 | pseudo |  |
| MACS_peak_59 | 435.76 | promoter-TSS (NM_198317) | KLHL17 | protein-coding | |
| MACS_peak_159 | 435.48 | promoter-TSS (NM_001318485) | MRPL20 | protein-coding | |
| MACS_peak_21686 | 435.25 | intron (NR_103819, intron 2 of 2) | SNRPD3 | protein-coding | |
| MACS_peak_143 | 435.19 | intron (NM_001256463, intron 1 of 14) | INTS11 | protein-coding | |
| MACS_peak_1135 | 435.18 | intron (NM_001177520, intron 3 of 9) | ALPL | protein-coding | |
| MACS_peak_6903 | 435.18 | intron (NM_033150, intron 43 of 52) | TMEM106C | protein-coding | |
| MACS_peak_13947 | 435.1 | promoter-TSS (NM_017456) | CYTH1 | protein-coding | |
| MACS_peak_27781 | 434.94 | exon (NM_001127218, exon 6 of 11) | MIR4649 | ncRNA |  |
| MACS_peak_20482 | 434.78 | Intergenic | RNA28SN5 | rRNA |  |
| MACS_peak_1006 | 434.59 | intron (NM_014675, intron 17 of 36) | CROCC | protein-coding | |
| MACS_peak_30094 | 433.96 | Intergenic | MIR4472-1 | ncRNA |  |
| MACS_peak_15943 | 433.86 | intron (NR_104176, intron 1 of 7) | LINC01529 | ncRNA |  |
| MACS_peak_9556 | 433.68 | intron (NM_001353792, intron 1 of 13).2 | APBA2 | protein-coding | |
| MACS_peak_24917 | 433.4 | intron (NM_007118, intron 55 of 56) | SNORD170 | snoRNA |  |
| MACS_peak_1566 | 433.27 | intron (NM_001291281, intron 1 of 2) | FOXO6 | protein-coding | |
| MACS_peak_9002 | 433.26 | intron (NM_024832, intron 4 of 9) | RIN3 | protein-coding | |
| MACS_peak_19453 | 433.25 | intron (NM_001281774, intron 3 of 23) | LOC100131496 | ncRNA |  |
| MACS_peak_8246 | 432.98 | intron (NM_001846, intron 4 of 47) | MIR8073 | ncRNA |  |
| MACS_peak_30224 | 432.86 | Intergenic | MAFA-AS1 | ncRNA |  |
| MACS_peak_27108 | 432.83 | Intergenic | LFNG | protein-coding | |
| MACS_peak_16536 | 432.82 | intron (NM_015711, intron 1 of 14) | BICRA | protein-coding | |
| MACS_peak_11123 | 432.71 | intron (NR_146336, intron 20 of 34) | MIR6511A4 | ncRNA |  |
| MACS_peak_25729 | 432.69 | Intergenic | LOC100132287 | ncRNA |  |
| MACS_peak_166 | 432.6 | Intergenic | ATAD3C | protein-coding | |
| MACS_peak_20085 | 432.56 | intron (NM_031215, intron 1 of 9) | CABLES2 | protein-coding | |
| MACS_peak_19527 | 432.31 | Intergenic | SNORD12 | snoRNA |  |
| MACS_peak_31645 | 431.9 | Intergenic | LCN9 | protein-coding | |
| MACS_peak_5263 | 431.61 | intron (NM_021926, intron 1 of 3) | ALX4 | protein-coding | |
| MACS_peak_8034 | 431.61 | Intergenic | MIR3169 | ncRNA |  |
| MACS_peak_8443 | 431.13 | intron (NM_001320821, intron 14 of 25).2 | C13orf46 | protein-coding | |
| MACS_peak_7187 | 431.11 | promoter-TSS (NM_182947) | ARHGEF25 | protein-coding | |
| MACS_peak_22631 | 430.89 | non-coding (NR_037162, exon 1 of 11) | TTLL3 | protein-coding | |
| MACS_peak_31025 | 430.68 | Intergenic | OR1N1 | protein-coding | |
| MACS_peak_21339 | 430.66 | intron (NM_058004, intron 54 of 54) | TMEM191A | pseudo |  |
| MACS_peak_7745 | 430.48 | intron (NM_003565, intron 20 of 27) | PUS1 | protein-coding | |
| MACS_peak_21436 | 430.4 | 3' UTR (NM_015094, exon 3 of 3) | TMEM191C | protein-coding | |
| MACS_peak_21379 | 429.97 | exon (NM_004173, exon 4 of 5) | SLC7A4 | protein-coding | |
| MACS_peak_28308 | 429.8 | intron (NM_032421, intron 1 of 15) | CLIP2 | protein-coding | |
| MACS_peak_31585 | 429.66 | intron (NM_001278074, intron 4 of 65) | COL5A1-AS1 | ncRNA |  |
| MACS_peak_27008 | 429.46 | Intergenic | ELFN1 | protein-coding | |
| MACS_peak_26948 | 429.42 | Intergenic | UNCX | protein-coding | |
| MACS_peak_7225 | 429.42 | promoter-TSS (NM_031435) | ZFC3H1 | protein-coding | |
| MACS_peak_28399 | 429.12 | Intergenic | SPDYE11 | protein-coding | |
| MACS_peak_26042 | 428.91 | 3' UTR (NM_007293, exon 41 of 41) | CYP21A1P | pseudo |  |
| MACS_peak_7661 | 428.86 | Intergenic | PIWIL1 | protein-coding | |
| MACS_peak_1353 | 428.53 | Intergenic | MATN1-AS1 | ncRNA |  |
| MACS_peak_22447 | 428.23 | Intergenic | PIM3 | protein-coding | |
| MACS_peak_16891 | 428.08 | promoter-TSS (NM_001020818) | MYADM | protein-coding | |
| MACS_peak_27053 | 427.98 | intron (NM_001013837, intron 14 of 18) | MAD1L1 | protein-coding | |
| MACS_peak_3278 | 427.93 | Intergenic | ITPKB-IT1 | ncRNA |  |
| MACS_peak_16980 | 427.7 | intron (NM_001369799, intron 4 of 5) | COX6B2 | protein-coding | |
| MACS_peak_20629 | 427.63 | Intergenic | DYRK1A | protein-coding | |
| MACS_peak_7734 | 427.52 | intron (NM_016155, intron 1 of 9) | MMP17 | protein-coding | |
| MACS_peak_21225 | 427.45 | intron (NR_147957, intron 6 of 16) | TXNRD2 | protein-coding | |
| MACS_peak_186 | 427.38 | intron (NM_001114748, intron 2 of 3) | TMEM240 | protein-coding | |
| MACS_peak_27934 | 427.37 | intron (NM_138295, intron 1 of 56) | PKD1L1 | protein-coding | |
| MACS_peak_21377 | 427.31 | exon (NM_001159554, exon 6 of 12) | P2RX6 | protein-coding | |
| MACS_peak_20667 | 426.96 | Intergenic | FAM3B | protein-coding | |
| MACS_peak_9536 | 426.52 | exon (NM_001365373, exon 1 of 9).7 | LOC100132202 | protein-coding | |
| MACS_peak_13817 | 426.44 | Intergenic | H3F3B | protein-coding | |
| MACS_peak_20750 | 426.08 | Intergenic | CBS | protein-coding | |
| MACS_peak_5539 | 425.76 | promoter-TSS (NM_032223) | PCNX3 | protein-coding | |
| MACS_peak_21411 | 425.66 | Intergenic | RIMBP3C | protein-coding | |
| MACS_peak_3986 | 425.64 | promoter-TSS (NM_181519) | LOC102724488 | protein-coding | |
| MACS_peak_624 | 425.06 | intron (NM_001349608, intron 2 of 21) | CAMTA1 | protein-coding | |
| MACS_peak_24541 | 424.86 | intron (NM_001271082, intron 8 of 10) | NKD2 | protein-coding | |
| MACS_peak_31480 | 424.71 | intron (NM_001080483, intron 1 of 4) | MYMK | protein-coding | |
| MACS_peak_12054 | 424.68 | TTS (NM_001142864) | MIR4722 | ncRNA |  |
| MACS_peak_21705 | 424.56 | intron (NM_001098498, intron 2 of 23) | SGSM1 | protein-coding | |
| MACS_peak_11073 | 424.4 | Intergenic | PKD1P6-NPIPP1 | pseudo |  |
| MACS_peak_122 | 423.54 | exon (NM_003327, exon 6 of 7) | TNFRSF4 | protein-coding | |
| MACS_peak_10152 | 423.49 | Intergenic | LOC102724034 | ncRNA |  |
| MACS_peak_14632 | 423.41 | intron (NM_001146345, intron 3 of 4) | SLC66A2 | protein-coding | |
| MACS_peak_27766 | 422.98 | intron (NM_001014436, intron 12 of 12) | PGAM2 | protein-coding | |
| MACS_peak_29059 | 422.83 | intron (NM_001130026, intron 8 of 10) | TCAF2 | protein-coding | |
| MACS_peak_5888 | 422.82 | intron (NM_012309, intron 13 of 24) | SHANK2-AS3 | ncRNA |  |
| MACS_peak_8467 | 422.82 | Intergenic | CDC16 | protein-coding | |
| MACS_peak_2250 | 422.63 | Intergenic | RNU1-1 | snRNA |  |
| MACS_peak_2596 | 422.57 | intron (NM_001319188, intron 1 of 6) | HDGF | protein-coding | |
| MACS_peak_21902 | 422.42 | promoter-TSS (NM_006604) | RFPL3 | protein-coding | |
| MACS_peak_27002 | 422.32 | Intergenic | TFAMP1 | pseudo |  |
| MACS_peak_27572 | 422.28 | intron (NM_001145514, intron 1 of 7) | SCRN1 | protein-coding | |
| MACS_peak_30219 | 422.02 | intron (NM_052924, intron 14 of 14) | RHPN1 | protein-coding | |
| MACS_peak_31705 | 421.58 | Intergenic | LHX3 | protein-coding | |
| MACS_peak_11111 | 421.54 | Intergenic | MIR3179-2 | ncRNA |  |
| MACS_peak_25144 | 421.33 | intron (NM_183323, intron 1 of 10) | PAIP1 | protein-coding | |
| MACS_peak_20066 | 420.83 | intron (NM_005560, intron 48 of 79) | MIR4758 | ncRNA |  |
| MACS_peak_11976 | 420.49 | intron (NM_003486, intron 2 of 9) | MIR11401 | ncRNA |  |
| MACS_peak_12841 | 420.31 | exon (NM_001291465, exon 14 of 14) | TBC1D3I | protein-coding | |
| MACS_peak_11999 | 419.87 | Intergenic | LINC02182 | ncRNA |  |
| MACS_peak_30978 | 419.87 | intron (NM_001286840, intron 1 of 14) | PHF19 | protein-coding | |
| MACS_peak_12140 | 419.65 | intron (NR_045839, intron 1 of 9) | LOC101927817 | ncRNA |  |
| MACS_peak_26194 | 419.61 | exon (NM_001315, exon 1 of 12) | MAPK14 | protein-coding | |
| MACS_peak_20014 | 419.35 | exon (NM_001794, exon 16 of 16) | MIR1257 | ncRNA |  |
| MACS_peak_15446 | 418.94 | Intergenic | KLF2 | protein-coding | |
| MACS_peak_16906 | 418.89 | promoter-TSS (NM_001282349) | NDUFA3 | protein-coding | |
| MACS_peak_16489 | 418.8 | intron (NM_016457, intron 1 of 17) | PRKD2 | protein-coding | |
| MACS_peak_65 | 418.73 | intron (NM_001367552, intron 13 of 15) | PLEKHN1 | protein-coding | |
| MACS_peak_27117 | 418.68 | promoter-TSS (NR_039791) | MIR4648 | ncRNA |  |
| MACS_peak_28213 | 418.51 | Intergenic | SPDYE8P | pseudo |  |
| MACS_peak_12603 | 418.36 | Intergenic | KRT16P3 | pseudo |  |
| MACS_peak_14545 | 418.36 | promoter-TSS (NR_040113) | ANKRD20A5P | pseudo |  |
| MACS_peak_4521 | 417.87 | promoter-TSS (NM_001164647) | SFTPA1 | protein-coding | |
| MACS_peak_20919 | 417.64 | Intergenic | COL6A2 | protein-coding | |
| MACS_peak_2993 | 417.63 | intron (NM_001304331, intron 2 of 29) | PPFIA4 | protein-coding | |
| MACS_peak_30299 | 417.56 | exon (NM_201380, exon 25 of 32) | PLEC | protein-coding | |
| MACS_peak_14047 | 417.54 | intron (NM_001079804, intron 3 of 19) | GAA | protein-coding | |
| MACS_peak_4138 | 417.24 | intron (NM_001351263, intron 1 of 5) | TSPAN15 | protein-coding | |
| MACS_peak_9452 | 416.83 | Intergenic | LOC646214 | pseudo |  |
| MACS_peak_14695 | 416.24 | intron (NM_001928, intron 3 of 4) | CFD | protein-coding | |
| MACS_peak_5099 | 416.22 | Intergenic | TALDO1 | protein-coding | |
| MACS_peak_985 | 416.06 | non-coding (NR_026567, exon 10 of 11) | MIR3675 | ncRNA |  |
| MACS_peak_2397 | 415.92 | promoter-TSS (NM_005979) | S100A1 | protein-coding | |
| MACS_peak_16678 | 415.77 | intron (NM_001145396, intron 1 of 15) | ALDH16A1 | protein-coding | |
| MACS_peak_10586 | 415.74 | Intergenic | SOX8 | protein-coding | |
| MACS_peak_15879 | 415.41 | exon (NM_001353433, exon 2 of 7) | PDCD2L | protein-coding | |
| MACS_peak_11848 | 415.39 | Intergenic | MIR5093 | ncRNA |  |
| MACS_peak_21092 | 415.32 | Intergenic | RIMBP3 | protein-coding | |
| MACS_peak_10506 | 415.13 | intron (NM_021259, intron 1 of 12) | TMEM8A | protein-coding | |
| MACS_peak_21031 | 415.06 | intron (NM_015241, intron 29 of 31) | LINC00528 | ncRNA |  |
| MACS_peak_31099 | 414.86 | intron (NM_001174146, intron 2 of 7) | LMX1B | protein-coding | |
| MACS_peak_4794 | 414.56 | intron (NM_005308, intron 1 of 15) | GRK5 | protein-coding | |
| MACS_peak_18241 | 414.47 | intron (NR_024077, intron 6 of 10) | DDX11L2 | pseudo |  |
| MACS_peak_20147 | 414.42 | Intergenic | SLCO4A1-AS1 | ncRNA |  |
| MACS_peak_5025 | 414.41 | TTS (NR_125763) | LINC01167 | ncRNA |  |
| MACS_peak_21282 | 414.21 | Intergenic | MIR1286 | ncRNA |  |
| MACS_peak_24965 | 414.13 | Intergenic | LINC02223 | ncRNA |  |
| MACS_peak_30332 | 413.89 | exon (NM_015201, exon 5 of 16) | SCX | protein-coding | |
| MACS_peak_15238 | 413.85 | 3' UTR (NM_004283, exon 5 of 5) | RAB3D | protein-coding | |
| MACS_peak_5643 | 413.76 | intron (NM_001300895, intron 4 of 5) | CABP4 | protein-coding | |
| MACS_peak_5826 | 413.75 | Intergenic | FGF3 | protein-coding | |
| MACS_peak_27339 | 413.63 | intron (NM_001145118, intron 3 of 21) | GRID2IP | protein-coding | |
| MACS_peak_644 | 413.54 | intron (NM_001349608, intron 5 of 21) | CAMTA1 | protein-coding | |
| MACS_peak_10220 | 413.34 | intron (NM_001267536, intron 9 of 10) | DNM1P41 | pseudo |  |
| MACS_peak_313 | 413.21 | TTS (NM_001303013) | PANK4 | protein-coding | |
| MACS_peak_20826 | 413.18 | intron (NR_103707, intron 6 of 6) | TSPEAR-AS2 | ncRNA |  |
| MACS_peak_24154 | 412.94 | Intergenic | CWH43 | protein-coding | |
| MACS_peak_2174 | 412.27 | Intergenic | LSP1P5 | pseudo |  |
| MACS_peak_24021 | 411.72 | intron (NM_001286688, intron 5 of 14) | MIR95 | ncRNA |  |
| MACS_peak_17025 | 411.61 | intron (NM_001012478, intron 9 of 11) | EPN1 | protein-coding | |
| MACS_peak_17951 | 411.56 | Intergenic | TMSB10 | protein-coding | |
| MACS_peak_10186 | 411.35 | Intergenic | LOC440300 | pseudo |  |
| MACS_peak_12092 | 411.24 | Intergenic | CBFA2T3 | protein-coding | |
| MACS_peak_20450 | 411.13 | promoter-TSS (NM_001330065) | KCNE1B | protein-coding | |
| MACS_peak_15125 | 411.06 | exon (NM_198492, exon 5 of 9) | CLEC4G | protein-coding | |
| MACS_peak_20757 | 411.01 | Intergenic | CRYAA | protein-coding | |
| MACS_peak_394 | 410.98 | intron (NM_022114, intron 1 of 16) | MIR4251 | ncRNA |  |
| MACS_peak_12640 | 410.87 | Intergenic | FLJ36000 | ncRNA |  |
| MACS_peak_31165 | 410.68 | Intergenic | ST6GALNAC4 | protein-coding | |
| MACS_peak_12191 | 410.45 | Intergenic | LOC102723753 | pseudo |  |
| MACS_peak_26518 | 409.95 | intron (NM_198081, intron 1 of 7) | SCML4 | protein-coding | |
| MACS_peak_10692 | 409.9 | TTS (NM_005061) | MSRB1 | protein-coding | |
| MACS_peak_21299 | 409.79 | Intergenic | DGCR6L | protein-coding | |
| MACS_peak_310 | 409.67 | intron (NM_014638, intron 11 of 21).2 | PLCH2 | protein-coding | |
| MACS_peak_448 | 409.5 | intron (NM_001409, intron 3 of 36) | MEGF6 | protein-coding | |
| MACS_peak_5149 | 409.38 | intron (NR_036542, intron 1 of 22) | CARS | protein-coding | |
| MACS_peak_15189 | 409.3 | intron (NM_015956, intron 1 of 8) | MRPL4 | protein-coding | |
| MACS_peak_27246 | 409.24 | Intergenic | RBAKDN | ncRNA |  |
| MACS_peak_5652 | 409.05 | intron (NM_016366, intron 2 of 6) | CABP2 | protein-coding | |
| MACS_peak_26752 | 408.85 | Intergenic | THBS2 | protein-coding | |
| MACS_peak_5723 | 408.64 | Intergenic | GAL | protein-coding | |
| MACS_peak_26001 | 408.45 | non-coding (NR_003948, exon 3 of 4) | HCG22 | protein-coding | |
| MACS_peak_16157 | 408.44 | promoter-TSS (NM_001256441) | PLD3 | protein-coding | |
| MACS_peak_14217 | 408.42 | intron (NM_000160, intron 5 of 13) | GCGR | protein-coding | |
| MACS_peak_28405 | 408.25 | TTS (NR_003664).5 | SPDYE11 | protein-coding | |
| MACS_peak_16653 | 408.12 | promoter-TSS (NM_003660) | PPFIA3 | protein-coding | |
| MACS_peak_5565 | 407.89 | promoter-TSS (NR_073078) | CNIH2 | protein-coding | |
| MACS_peak_21720 | 407.7 | Intergenic | KIAA1671 | protein-coding | |
| MACS_peak_26561 | 407.51 | Intergenic | AKAP7 | protein-coding | |
| MACS_peak_5890 | 407.49 | intron (NM_012309, intron 13 of 24) | SHANK2-AS3 | ncRNA |  |
| MACS_peak_16510 | 407.25 | intron (NM_001127240, intron 1 of 3) | BBC3 | protein-coding | |
| MACS_peak_11604 | 407.21 | intron (NM_001004055, intron 3 of 6) | MIR328 | ncRNA |  |
| MACS_peak_24914 | 407.17 | intron (NM_007118, intron 46 of 56) | SNORD170 | snoRNA |  |
| MACS_peak_14823 | 407.09 | 5' UTR (NM_017572, exon 1 of 14) | MKNK2 | protein-coding | |
| MACS_peak_3040 | 407.04 | Intergenic | MDM4 | protein-coding | |
| MACS_peak_21270 | 406.82 | Intergenic | LOC284865 | ncRNA |  |
| MACS_peak_30252 | 406.8 | exon (NM_001100878, exon 1 of 14).2 | MROH6 | protein-coding | |
| MACS_peak_9589 | 406.58 | promoter-TSS (NM_001355476).4 | GOLGA8Q | protein-coding | |
| MACS_peak_593 | 406.37 | intron (NM_031475, intron 1 of 12) | MIR4252 | ncRNA |  |
| MACS_peak_21473 | 406.35 | 3' UTR (NM_014337, exon 20 of 20) | PPIL2 | protein-coding | |
| MACS_peak_24618 | 406.2 | Intergenic | LPCAT1 | protein-coding | |
| MACS_peak_14181 | 406.11 | intron (NM_001291324, intron 13 of 27) | MIR3186 | ncRNA |  |
| MACS_peak_15989 | 406.04 | Intergenic | LOC284412 | ncRNA |  |
| MACS_peak_26792 | 406.04 | intron (NM_020223, intron 2 of 9) | FAM20C | protein-coding | |
| MACS_peak_25798 | 405.86 | intron (NM_021945, intron 4 of 10) | PSMG4 | protein-coding | |
| MACS_peak_10151 | 405.78 | Intergenic | LOC102724034 | ncRNA |  |
| MACS_peak_10596 | 405.72 | non-coding (NR_027242, exon 4 of 4) | SSTR5 | protein-coding | |
| MACS_peak_4972 | 405.47 | intron (NM_001318878, intron 1 of 11) | STK32C | protein-coding | |
| MACS_peak_20187 | 405.24 | intron (NR_033370, intron 1 of 4) | LINC01749 | ncRNA |  |
| MACS_peak_1760 | 405.17 | intron (NR_109858, intron 2 of 10) | SLC1A7 | protein-coding | |
| MACS_peak_13209 | 405.05 | intron (NR_148207, intron 10 of 10) | ADAM11 | protein-coding | |
| MACS_peak_643 | 404.91 | intron (NM_001349608, intron 5 of 21) | LOC102725193 | ncRNA |  |
| MACS_peak_21408 | 404.84 | Intergenic | FAM230H | ncRNA |  |
| MACS_peak_30808 | 404.73 | intron (NM_052820, intron 1 of 11) | CORO2A | protein-coding | |
| MACS_peak_11781 | 404.66 | promoter-TSS (NM_178452) | HSDL1 | protein-coding | |
| MACS_peak_28902 | 404.61 | exon (NM_001458, exon 24 of 48) | FLNC-AS1 | ncRNA |  |
| MACS_peak_22629 | 404.52 | promoter-TSS (NM_001024959) | ARPC4 | protein-coding | |
| MACS_peak_14978 | 404.51 | promoter-TSS (NM_001348169) | HDGFL2 | protein-coding | |
| MACS_peak_29265 | 404.5 | promoter-TSS (NM_014671) | UBE3C | protein-coding | |
| MACS_peak_20353 | 404.39 | promoter-TSS (NR_106871) | MIR6813 | ncRNA |  |
| MACS_peak_20261 | 404.36 | intron (NM_172109, intron 1 of 7) | KCNQ2 | protein-coding | |
| MACS_peak_29312 | 403.95 | intron (NM_001308267, intron 2 of 21) | MIR595 | ncRNA |  |
| MACS_peak_31883 | 403.91 | intron (NM_001286823, intron 1 of 7) | EXD3 | protein-coding | |
| MACS_peak_2378 | 403.83 | intron (NM_001319198, intron 1 of 2) | S100A7A | protein-coding | |
| MACS_peak_13202 | 403.26 | intron (NM_000419, intron 26 of 29) | FAM171A2 | protein-coding | |
| MACS_peak_24495 | 403.14 | intron (NM_007030, intron 1 of 3) | TPPP | protein-coding | |
| MACS_peak_1788 | 403.08 | intron (NR_033142, intron 5 of 16) | NDC1 | protein-coding | |
| MACS_peak_12120 | 402.93 | Intergenic | ZNF778 | protein-coding | |
| MACS_peak_22624 | 402.76 | intron (NM_153635, intron 19 of 19) | BRPF1 | protein-coding | |
| MACS_peak_19147 | 402.68 | intron (NM_031232, intron 1 of 11) | NECAB3 | protein-coding | |
| MACS_peak_11055 | 402.44 | intron (NM_016561, intron 1 of 7) | BFAR | protein-coding | |
| MACS_peak_32010 | 401.86 | NA |  |  |  |
| MACS_peak_2926 | 401.79 | intron (NM_001252103, intron 15 of 33) | KIF21B | protein-coding | |
| MACS_peak_9632 | 401.16 | intron (NM_001282494, intron 15 of 18).3 | GOLGA8N | protein-coding | |
| MACS_peak_15584 | 400.76 | Intergenic | LRRC25 | protein-coding | |
| MACS_peak_2326 | 400.73 | promoter-TSS (NM_144618) | GABPB2 | protein-coding | |
| MACS_peak_24502 | 400.22 | intron (NM_001351303, intron 1 of 10) | ZDHHC11B | protein-coding | |
| MACS_peak_3840 | 400.07 | intron (NM_145313, intron 3 of 12) | RASGEF1A | protein-coding | |
| MACS_peak_17316 | 399.85 | Intergenic | HS1BP3-IT1 | ncRNA |  |
| MACS_peak_24579 | 399.82 | intron (NM_001003841, intron 6 of 11) | SLC6A18 | protein-coding | |
| MACS_peak_15962 | 399.64 | intron (NM_001297701, intron 7 of 7) | SYNE4 | protein-coding | |
| MACS_peak_27483 | 399.51 | promoter-TSS (NM_000905) | NPY | protein-coding | |
| MACS_peak_14613 | 399.48 | intron (NR_134646, intron 1 of 1) | LINC01898 | ncRNA |  |
| MACS_peak_32333 | 399.35 | Intergenic | MTM1 | protein-coding | |
| MACS_peak_13349 | 399.29 | TTS (NM_001135186) | PHOSPHO1 | protein-coding | |
| MACS_peak_441 | 399.26 | intron (NM_001409, intron 4 of 36) | MIR551A | ncRNA |  |
| MACS_peak_17156 | 398.99 | Intergenic | LINC01250 | ncRNA |  |
| MACS_peak_23739 | 398.99 | non-coding (NR_145451, exon 1 of 1) | LINC02012 | ncRNA |  |
| MACS_peak_21525 | 398.74 | Intergenic | POM121L1P | pseudo |  |
| MACS_peak_16162 | 398.7 | intron (NM_144685, intron 2 of 3) | HIPK4 | protein-coding | |
| MACS_peak_31724 | 398.59 | intron (NM_001145639, intron 3 of 3) | GPSM1 | protein-coding | |
| MACS_peak_29328 | 398.48 | intron (NM_130843, intron 1 of 21) | MIR595 | ncRNA |  |
| MACS_peak_17621 | 398.18 | Intergenic | LINC01819 | ncRNA |  |
| MACS_peak_855 | 397.98 | intron (NM_001316320, intron 17 of 19) | MFN2 | protein-coding | |
| MACS_peak_9292 | 397.68 | Intergenic | NUDT14 | protein-coding | |
| MACS_peak_26260 | 397.11 | intron (NM_015345, intron 1 of 24) | DAAM2 | protein-coding | |
| MACS_peak_20603 | 396.7 | promoter-TSS (NM_001127668) | KCNE1 | protein-coding | |
| MACS_peak_27513 | 396.22 | promoter-TSS (NR_136271) | LOC105375304 | ncRNA |  |
| MACS_peak_19810 | 395.97 | Intergenic | LINC01742 | ncRNA |  |
| MACS_peak_22071 | 395.73 | intron (NM_025045, intron 2 of 13) | BAIAP2L2 | protein-coding | |
| MACS_peak_14654 | 395.55 | intron (NM_033513, intron 1 of 1) | TPGS1 | protein-coding | |
| MACS_peak_26977 | 395.25 | Intergenic | MICALL2 | protein-coding | |
| MACS_peak_30220 | 395.08 | TTS (NM_052924) | RHPN1 | protein-coding | |
| MACS_peak_13401 | 394.9 | Intergenic | COL1A1 | protein-coding | |
| MACS_peak_12025 | 394.66 | intron (NM_153813, intron 2 of 9) | MIR5189 | ncRNA |  |
| MACS_peak_10600 | 394.37 | 3' UTR (NM_207419, exon 5 of 5) | C1QTNF8 | protein-coding | |
| MACS_peak_14238 | 394.37 | intron (NR_045351, intron 2 of 14) | ASPSCR1 | protein-coding | |
| MACS_peak_12831 | 394.31 | intron (NM_033315, intron 1 of 3) | RASL10B | protein-coding | |
| MACS_peak_20229 | 394.14 | exon (NM_020882, exon 11 of 36) | COL20A1 | protein-coding | |
| MACS_peak_16967 | 394.12 | promoter-TSS (NM_001161440) | PTPRH | protein-coding | |
| MACS_peak_19937 | 393.99 | Intergenic | CDH4 | protein-coding | |
| MACS_peak_3604 | 393.93 | intron (NR_033387, intron 1 of 1) | ADARB2-AS1 | ncRNA |  |
| MACS_peak_24005 | 393.91 | intron (NM_020777, intron 1 of 26) | MIR4798 | ncRNA |  |
| MACS_peak_31596 | 393.89 | intron (NM_001278074, intron 64 of 65) | MIR3689C | ncRNA |  |
| MACS_peak_30414 | 393.61 | Intergenic | TUSC1 | protein-coding | |
| MACS_peak_18742 | 393.57 | intron (NR_073043, intron 3 of 19) | IQCA1 | protein-coding | |
| MACS_peak_16627 | 393.55 | promoter-TSS (NM_016246) | HSD17B14 | protein-coding | |
| MACS_peak_14446 | 393.54 | intron (NM_006868, intron 1 of 6) | RAB31 | protein-coding | |
| MACS_peak_22804 | 393.17 | Intergenic | LINC02011 | ncRNA |  |
| MACS_peak_11398 | 392.93 | Intergenic | ZNF267 | protein-coding | |
| MACS_peak_7553 | 392.83 | intron (NM_001077261, intron 42 of 47) | MIR6880 | ncRNA |  |
| MACS_peak_25897 | 392.73 | intron (NM_006877, intron 4 of 8) | GMPR | protein-coding | |
| MACS_peak_22480 | 392.29 | intron (NM_001001794, intron 14 of 19) | PLXNB2 | protein-coding | |
| MACS_peak_5862 | 392.19 | intron (NM_005231, intron 14 of 17) | CTTN | protein-coding | |
| MACS_peak_2484 | 391.97 | non-coding (NR_045515, exon 6 of 6) | ZBTB7B | protein-coding | |
| MACS_peak_20447 | 391.9 | Intergenic | FAM243A | protein-coding | |
| MACS_peak_27244 | 391.87 | TTS (NR_015343) | RBAKDN | ncRNA |  |
| MACS_peak_24692 | 391.77 | 3' UTR (NM_001365685, exon 3 of 3) | C5orf38 | protein-coding | |
| MACS_peak_24520 | 391.76 | intron (NM_001009877, intron 8 of 15) | BRD9 | protein-coding | |
| MACS_peak_19792 | 391.71 | promoter-TSS (NM_020182) | NKILA | ncRNA |  |
| MACS_peak_18802 | 391.63 | Intergenic | COPS9 | protein-coding | |
| MACS_peak_3150 | 391.56 | intron (NM_001287754, intron 1 of 6) | TRAF3IP3 | protein-coding | |
| MACS_peak_18181 | 391.56 | promoter-TSS (NM_178584) | SOWAHC | protein-coding | |
| MACS_peak_994 | 391.47 | Intergenic | RNU1-1 | snRNA |  |
| MACS_peak_26666 | 391.43 | promoter-TSS (NR_160977) | C6orf99 | ncRNA |  |
| MACS_peak_19990 | 391.3 | intron (NM_001252339, intron 3 of 14) | LOC100128310 | ncRNA |  |
| MACS_peak_965 | 391.12 | intron (NM_153213, intron 8 of 15) | ARHGEF19 | protein-coding | |
| MACS_peak_2193 | 391.04 | Intergenic | LSP1P5 | pseudo |  |
| MACS_peak_28566 | 390.86 | Intergenic | TMEM130 | protein-coding | |
| MACS_peak_12071 | 390.77 | intron (NM_030928, intron 1 of 9) | CDT1 | protein-coding | |
| MACS_peak_9441 | 390.41 | TTS (NR_049895) | LINC01193 | ncRNA |  |
| MACS_peak_231 | 390.38 | intron (NM_001304360, intron 6 of 38) | CFAP74 | protein-coding | |
| MACS_peak_31798 | 390.36 | intron (NM_001080482, intron 1 of 2) | AJM1 | protein-coding | |
| MACS_peak_16430 | 390.3 | TTS (NM_000164) | SNRPD2 | protein-coding | |
| MACS_peak_14032 | 390.27 | Intergenic | CBX4 | protein-coding | |
| MACS_peak_121 | 390.2 | Intergenic | TNFRSF18 | protein-coding | |
| MACS_peak_26807 | 389.77 | Intergenic | LOC442497 | ncRNA |  |
| MACS_peak_18712 | 389.74 | intron (NM_001367507, intron 12 of 41) | MROH2A | protein-coding | |
| MACS_peak_28352 | 389.58 | intron (NR_027777, intron 3 of 3) | PMS2P5 | pseudo |  |
| MACS_peak_27555 | 389.57 | exon (NM_004904, exon 8 of 11) | CREB5 | protein-coding | |
| MACS_peak_2210 | 389.45 | Intergenic | RNVU1-11 | snRNA |  |
| MACS_peak_13405 | 389.33 | intron (NM_001168215, intron 1 of 5) | TMEM92 | protein-coding | |
| MACS_peak_28664 | 389.17 | intron (NM_001363494, intron 6 of 10) | TRIP6 | protein-coding | |
| MACS_peak_25218 | 389.05 | intron (NR_033968, intron 1 of 2) | GUSBP9 | pseudo |  |
| MACS_peak_16467 | 388.79 | promoter-TSS (NM_022462) | HIF3A | protein-coding | |
| MACS_peak_16122 | 388.74 | intron (NM_022835, intron 6 of 18) | PLEKHG2 | protein-coding | |
| MACS_peak_6965 | 388.5 | exon (NM_001145475, exon 4 of 8) | FAM186A | protein-coding | |
| MACS_peak_13650 | 388.22 | promoter-TSS (NM_015462) | NOL11 | protein-coding | |
| MACS_peak_9712 | 388.21 | Intergenic | MIR147B | ncRNA |  |
| MACS_peak_16751 | 388.1 | Intergenic | MYH14 | protein-coding | |
| MACS_peak_24881 | 387.51 | intron (NM_001288715, intron 2 of 20) | CTNND2 | protein-coding | |
| MACS_peak_6252 | 387.38 | intron (NM_001243270, intron 17 of 23) | ARHGAP42 | protein-coding | |
| MACS_peak_15504 | 387.31 | intron (NM_133644, intron 3 of 7) | GTPBP3 | protein-coding | |
| MACS_peak_22359 | 387.19 | intron (NR_110479, intron 1 of 4) | MIR3619 | ncRNA |  |
| MACS_peak_165 | 387.01 | Intergenic | ATAD3C | protein-coding | |
| MACS_peak_29392 | 386.95 | intron (NM_001346810, intron 8 of 14) | DLGAP2-AS1 | ncRNA |  |
| MACS_peak_5185 | 386.84 | intron (NM_001329630, intron 3 of 26) | PLEKHA7 | protein-coding | |
| MACS_peak_15998 | 386.53 | Intergenic | ZNF875 | protein-coding | |
| MACS_peak_7741 | 386.36 | intron (NM_003565, intron 3 of 27) | ULK1 | protein-coding | |
| MACS_peak_24549 | 385.97 | intron (NM_006598, intron 17 of 23).2 | MIR4635 | ncRNA |  |
| MACS_peak_285 | 385.9 | promoter-TSS (NR_125361) | RER1 | protein-coding | |
| MACS_peak_21320 | 385.83 | exon (NM_032775, exon 2 of 7) | KLHL22 | protein-coding | |
| MACS_peak_14348 | 385.58 | Intergenic | RPL23AP87 | pseudo |  |
| MACS_peak_19590 | 385.57 | Intergenic | LINC01271 | ncRNA |  |
| MACS_peak_5686 | 385.57 | exon (NM_006053, exon 12 of 15) | MIR6753 | ncRNA |  |
| MACS_peak_26048 | 385.25 | intron (NM_001002029, intron 40 of 40).3 | CYP21A2 | protein-coding | |
| MACS_peak_31109 | 385.22 | intron (NM_001190729, intron 8 of 17) | ANGPTL2 | protein-coding | |
| MACS_peak_20032 | 385.13 | Intergenic | LSM14B | protein-coding | |
| MACS_peak_12584 | 385.01 | Intergenic | SLC47A1 | protein-coding | |
| MACS_peak_10519 | 384.89 | intron (NM_001142272, intron 7 of 11) | LINC00235 | ncRNA |  |
| MACS_peak_21168 | 384.85 | promoter-TSS (NM_001363066) | CLDN5 | protein-coding | |
| MACS_peak_350 | 384.73 | Intergenic | TTC34 | protein-coding | |
| MACS_peak_20378 | 384.63 | Intergenic | LINC00266-1 | ncRNA |  |
| MACS_peak_13237 | 384.6 | intron (NM_003954, intron 11 of 15) | SPATA32 | protein-coding | |
| MACS_peak_20283 | 384.41 | promoter-TSS (NM_001319152) | FNDC11 | protein-coding | |
| MACS_peak_12211 | 384.35 | intron (NM_001205319, intron 5 of 7) | MRM3 | protein-coding | |
| MACS_peak_2662 | 384.33 | intron (NM_001001734, intron 4 of 4) | CASQ1 | protein-coding | |
| MACS_peak_8882 | 384.26 | exon (NM_014909, exon 5 of 7) | VASH1-AS1 | ncRNA |  |
| MACS_peak_2862 | 384.15 | Intergenic | LAMC1-AS1 | ncRNA |  |
| MACS_peak_16780 | 383.96 | intron (NM_001080457, intron 1 of 2) | LRRC4B | protein-coding | |
| MACS_peak_11774 | 383.96 | intron (NM_019065, intron 2 of 12) | NECAB2 | protein-coding | |
| MACS_peak_1798 | 383.72 | promoter-TSS (NM_145716) | SSBP3 | protein-coding | |
| MACS_peak_9818 | 383.23 | intron (NM_001004439, intron 11 of 29) | FEM1B | protein-coding | |
| MACS_peak_4962 | 383.09 | intron (NM_001323086, intron 22 of 23) | DPYSL4 | protein-coding | |
| MACS_peak_24099 | 383.03 | Intergenic | PACRGL | protein-coding | |
| MACS_peak_18203 | 382.97 | non-coding (NR_046110, exon 2 of 4).2 | LINC01123 | ncRNA |  |
| MACS_peak_21668 | 382.9 | intron (NR_003714, intron 5 of 6) | POM121L9P | pseudo |  |
| MACS_peak_19578 | 382.75 | Intergenic | CEBPB-AS1 | ncRNA |  |
| MACS_peak_19476 | 382.69 | Intergenic | SULF2 | protein-coding | |
| MACS_peak_3092 | 382.66 | promoter-TSS (NM_033102) | SLC45A3 | protein-coding | |
| MACS_peak_10881 | 382.6 | intron (NR_040252, intron 12 of 16) | NUDT16L1 | protein-coding | |
| MACS_peak_268 | 382.58 | intron (NM_003036, intron 1 of 6) | SKI | protein-coding | |
| MACS_peak_31587 | 382.55 | intron (NM_001278074, intron 6 of 65) | COL5A1-AS1 | ncRNA |  |
| MACS_peak_9044 | 382.48 | Intergenic | C14orf132 | protein-coding | |
| MACS_peak_145 | 382.46 | promoter-TSS (NM_152228) | TAS1R3 | protein-coding | |
| MACS_peak_2928 | 382.44 | intron (NM_001252103, intron 1 of 33) | KIF21B | protein-coding | |
| MACS_peak_28934 | 382.4 | Intergenic | LOC100128325 | ncRNA |  |
| MACS_peak_10218 | 382.38 | intron (NM_001267536, intron 9 of 10) | DNM1P41 | pseudo |  |
| MACS_peak_20405 | 382.32 | TTS (NR_152722) | LINC00319 | ncRNA |  |
| MACS_peak_13052 | 382.27 | Intergenic | KRT14 | protein-coding | |
| MACS_peak_17376 | 382.03 | exon (NM_153759, exon 2 of 19) | DNMT3A | protein-coding | |
| MACS_peak_20504 | 381.94 | Intergenic | LOC105379514 | ncRNA |  |
| MACS_peak_21256 | 381.9 | promoter-TSS (NM_182984) | RANBP1 | protein-coding | |
| MACS_peak_28722 | 381.82 | intron (NM_001202543, intron 20 of 23) | SH2B2 | protein-coding | |
| MACS_peak_28598 | 381.82 | intron (NR_134530, intron 1 of 3) | LAMTOR4 | protein-coding | |
| MACS_peak_16940 | 381.49 | Intergenic | NCR1 | protein-coding | |
| MACS_peak_32343 | 381.48 | intron (NM_001011550, intron 1 of 2) | MAGEA4 | protein-coding | |
| MACS_peak_14707 | 381.4 | intron (NM_005224, intron 3 of 8) | ARID3A | protein-coding | |
| MACS_peak_28671 | 381.21 | 3' UTR (NM_005960, exon 12 of 12) | MUC12 | protein-coding | |
| MACS_peak_14919 | 381.19 | intron (NM_016263, intron 1 of 13) | FZR1 | protein-coding | |
| MACS_peak_29037 | 381.09 | intron (NM_001363538, intron 11 of 11) | MIR11400 | ncRNA |  |
| MACS_peak_27076 | 381.05 | intron (NM_001013837, intron 5 of 18) | MAD1L1 | protein-coding | |
| MACS_peak_32234 | 380.94 | intron (NR_147172, intron 2 of 2) | DANT1 | ncRNA |  |
| MACS_peak_23696 | 380.85 | promoter-TSS (NM_005781) | TNK2 | protein-coding | |
| MACS_peak_31245 | 380.83 | Intergenic | IER5L | protein-coding | |
| MACS_peak_8043 | 380.63 | intron (NR_126409, intron 5 of 6) | LINC00376 | ncRNA |  |
| MACS_peak_29760 | 380.6 | non-coding (NR_003594, exon 1 of 1).6 | REXO1L2P | pseudo |  |
| MACS_peak_9576 | 380.5 | intron (NM_001355469, intron 1 of 18).3 | GOLGA8T | protein-coding | |
| MACS_peak_2695 | 379.93 | intron (NM_001166159, intron 6 of 12) | FCER1G | protein-coding | |
| MACS_peak_6586 | 379.57 | promoter-TSS (NM_002014) | FKBP4 | protein-coding | |
| MACS_peak_15207 | 379.53 | promoter-TSS (NR_031603) | MIR1238 | ncRNA |  |
| MACS_peak_14063 | 378.89 | intron (NM_020954, intron 3 of 16) | RNF213 | protein-coding | |
| MACS_peak_5804 | 378.83 | intron (NM_053056, intron 1 of 4) | CCND1 | protein-coding | |
| MACS_peak_22377 | 378.81 | intron (NM_014246, intron 1 of 34) | CELSR1 | protein-coding | |
| MACS_peak_15128 | 378.54 | intron (NM_001159944, intron 1 of 19) | EVI5L | protein-coding | |
| MACS_peak_18481 | 378.49 | Intergenic | TFPI | protein-coding | |
| MACS_peak_14334 | 378.39 | intron (NM_001004431, intron 2 of 3) | METRNL | protein-coding | |
| MACS_peak_2503 | 378.27 | intron (NM_001204285, intron 2 of 8) | MUC1 | protein-coding | |
| MACS_peak_30578 | 378.16 | promoter-TSS (NR_121647) | LINC01410 | ncRNA |  |
| MACS_peak_21565 | 378.09 | intron (NM_004327, intron 16 of 22) | LINC02556 | ncRNA |  |
| MACS_peak_7767 | 378.01 | intron (NM_001122636, intron 6 of 10) | GALNT9 | protein-coding | |
| MACS_peak_17433 | 377.94 | intron (NM_001253724, intron 1 of 12) | DPYSL5 | protein-coding | |
| MACS_peak_5329 | 377.89 | promoter-TSS (NM_005693) | NR1H3 | protein-coding | |
| MACS_peak_30095 | 377.51 | Intergenic | MIR4472-1 | ncRNA |  |
| MACS_peak_5474 | 377.5 | promoter-TSS (NM_032344) | TRPT1 | protein-coding | |
| MACS_peak_25365 | 377.39 | promoter-TSS (NM_032151) | PCBD2 | protein-coding | |
| MACS_peak_18808 | 377 | Intergenic | GPC1 | protein-coding | |
| MACS_peak_27857 | 376.93 | Intergenic | RAMP3 | protein-coding | |
| MACS_peak_2151 | 376.74 | Intergenic | SEC22B | protein-coding | |
| MACS_peak_8116 | 376.74 | Intergenic | LINC00430 | ncRNA |  |
| MACS_peak_16492 | 376.68 | promoter-TSS (NM_001145144) | SLC1A5 | protein-coding | |
| MACS_peak_14683 | 376.57 | intron (NM_002579, intron 8 of 8) | MISP | protein-coding | |
| MACS_peak_8011 | 376.54 | intron (NM_198441, intron 1 of 2) | PRR20D | protein-coding | |
| MACS_peak_16746 | 376.54 | TTS (NR_024214).8 | SNAR-A8 | snRNA |  |
| MACS_peak_4240 | 376.44 | intron (NM_001244889, intron 5 of 15) | SLC29A3 | protein-coding | |
| MACS_peak_13021 | 376.07 | intron (NM_001024809, intron 6 of 7) | GJD3 | protein-coding | |
| MACS_peak_17920 | 375.85 | exon (NM_001381, exon 2 of 5) | DOK1 | protein-coding | |
| MACS_peak_14008 | 375.59 | Intergenic | RBFOX3 | protein-coding | |
| MACS_peak_9186 | 375.57 | intron (NM_001080464, intron 13 of 15) | MIR203A | ncRNA |  |
| MACS_peak_26981 | 375.55 | intron (NM_182924, intron 1 of 16) | MICALL2 | protein-coding | |
| MACS_peak_9159 | 375.39 | Intergenic | LINC00605 | ncRNA |  |
| MACS_peak_20304 | 375.32 | intron (NR_037882, intron 30 of 37) | TNFRSF6B | protein-coding | |
| MACS_peak_20267 | 375.24 | Intergenic | KCNQ2 | protein-coding | |
| MACS_peak_20089 | 375.2 | intron (NM_080833, intron 2 of 13) | RBBP8NL | protein-coding | |
| MACS_peak_14351 | 374.97 | Intergenic | MGC70870 | pseudo |  |
| MACS_peak_27772 | 374.91 | Intergenic | POLM | protein-coding | |
| MACS_peak_3847 | 374.9 | intron (NM_145313, intron 1 of 12) | RASGEF1A | protein-coding | |
| MACS_peak_14531 | 374.87 | intron (NM_181482, intron 4 of 5) | MIR4526 | ncRNA |  |
| MACS_peak_30264 | 374.8 | Intergenic | ZNF707 | protein-coding | |
| MACS_peak_20150 | 374.65 | intron (NM_002531, intron 1 of 3) | NTSR1 | protein-coding | |
| MACS_peak_31199 | 374.52 | intron (NR_031672, intron 2 of 2).4 | MIR219A2 | ncRNA |  |
| MACS_peak_31160 | 374.5 | promoter-TSS (NM_001318122) | AK1 | protein-coding | |
| MACS_peak_27815 | 374.32 | exon (NM_001101648, exon 4 of 19) | NPC1L1 | protein-coding | |
| MACS_peak_8578 | 374.32 | Intergenic | GZMB | protein-coding | |
| MACS_peak_16342 | 374.27 | promoter-TSS (NM_001289166) | ZNF227 | protein-coding | |
| MACS_peak_28616 | 374.18 | promoter-TSS (NR_103728) | PVRIG2P | pseudo |  |
| MACS_peak_8509 | 373.76 | Intergenic | RNASE2 | protein-coding | |
| MACS_peak_3077 | 373.67 | promoter-TSS (NR_029893) | MIR135B | ncRNA |  |
| MACS_peak_28704 | 373.64 | Intergenic | MYL10 | protein-coding | |
| MACS_peak_5955 | 373.6 | intron (NM_001143839, intron 3 of 29) | MIR139 | ncRNA |  |
| MACS_peak_21044 | 373.57 | promoter-TSS (NM_018943) | TUBA8 | protein-coding | |
| MACS_peak_15758 | 373.43 | Intergenic | POP4 | protein-coding | |
| MACS_peak_28226 | 373.29 | Intergenic | SPDYE11 | protein-coding | |
| MACS_peak_22613 | 373.21 | intron (NR_046358, intron 11 of 12) | SSUH2 | protein-coding | |
| MACS_peak_21789 | 373.2 | promoter-TSS (NM_001284265) | CCDC117 | protein-coding | |
| MACS_peak_14387 | 372.68 | intron (NM_001242762, intron 3 of 9) | DLGAP1 | protein-coding | |
| MACS_peak_18240 | 372.46 | intron (NR_024077, intron 2 of 10) | WASH2P | pseudo |  |
| MACS_peak_31945 | 372.44 | Intergenic | LOC389831 | protein-coding | |
| MACS_peak_21276 | 372.43 | Intergenic | LINC00896 | ncRNA |  |
| MACS_peak_14702 | 372.27 | intron (NM_005224, intron 1 of 8) | ARID3A | protein-coding | |
| MACS_peak_21003 | 372.19 | promoter-TSS (NM_001163079) | TMEM121B | protein-coding | |
| MACS_peak_11124 | 372.14 | intron (NR_146336, intron 20 of 34) | MIR6511A4 | ncRNA |  |
| MACS_peak_5128 | 372.05 | intron (NM_001256630, intron 1 of 19) | BRSK2 | protein-coding | |
| MACS_peak_5776 | 372.04 | Intergenic | MYEOV | protein-coding | |
| MACS_peak_29267 | 371.8 | intron (NM_005494, intron 1 of 7) | DNAJB6 | protein-coding | |
| MACS_peak_28725 | 371.79 | intron (NM_001913, intron 14 of 22) | SH2B2 | protein-coding | |
| MACS_peak_13344 | 371.52 | promoter-TSS (NR_135674) | LOC105371814 | ncRNA |  |
| MACS_peak_12377 | 371.51 | intron (NM_001165966, intron 2 of 18) | PITPNM3 | protein-coding | |
| MACS_peak_24437 | 371.45 | intron (NM_020731, intron 3 of 11) | AHRR | protein-coding | |
| MACS_peak_30008 | 371.39 | intron (NM_001160372, intron 15 of 22) | PEG13 | ncRNA |  |
| MACS_peak_10563 | 371.22 | exon (NM_022092, exon 20 of 22) | GNG13 | protein-coding | |
| MACS_peak_14094 | 371 | intron (NM_020761, intron 9 of 33) | LOC101928855 | ncRNA |  |
| MACS_peak_15477 | 370.96 | intron (NM_001011699, intron 1 of 10) | HAUS8 | protein-coding | |
| MACS_peak_28779 | 370.93 | Intergenic | UPK3BL1 | protein-coding | |
| MACS_peak_12116 | 370.84 | intron (NM_004933, intron 7 of 13) | SLC22A31 | protein-coding | |
| MACS_peak_1954 | 370.67 | intron (NM_006769, intron 2 of 4) | LMO4 | protein-coding | |
| MACS_peak_18996 | 370.51 | intron (NM_020689, intron 12 of 16) | RIN2 | protein-coding | |
| MACS_peak_5569 | 370.44 | TTS (NM_020404) | CD248 | protein-coding | |
| MACS_peak_12762 | 370.32 | intron (NR_144394, intron 1 of 8) | SUZ12P1 | pseudo |  |
| MACS_peak_20158 | 370.31 | intron (NR_046224, intron 1 of 2) | LINC00659 | ncRNA |  |
| MACS_peak_26986 | 370.28 | intron (NM_001080453, intron 20 of 47) | INTS1 | protein-coding | |
| MACS_peak_9392 | 370.21 | NA |  |  |  |
| MACS_peak_10224 | 369.72 | intron (NM_001267536, intron 9 of 10) | DNM1P41 | pseudo |  |
| MACS_peak_24382 | 369.45 | Intergenic | DUX4 | protein-coding | |
| MACS_peak_5859 | 369.37 | intron (NM_005231, intron 5 of 17) | CTTN | protein-coding | |
| MACS_peak_14162 | 369.35 | Intergenic | LINC00482 | ncRNA |  |
| MACS_peak_29318 | 369.34 | intron (NM_130842, intron 1 of 21) | MIR595 | ncRNA |  |
| MACS_peak_4102 | 369.34 | Intergenic | JMJD1C | protein-coding | |
| MACS_peak_2387 | 369.19 | TTS (NM_014624) | S100A6 | protein-coding | |
| MACS_peak_15843 | 368.8 | intron (NM_001166056, intron 3 of 12) | PEPD | protein-coding | |
| MACS_peak_2496 | 368.8 | promoter-TSS (NM_004428) | EFNA1 | protein-coding | |
| MACS_peak_8346 | 368.77 | intron (NM_032189, intron 4 of 28) | ATP11A-AS1 | ncRNA |  |
| MACS_peak_8209 | 368.66 | intron (NM_001350749, intron 1 of 43) | NALCN | protein-coding | |
| MACS_peak_21004 | 368.48 | Intergenic | LINC01664 | ncRNA |  |
| MACS_peak_15882 | 368.44 | Intergenic | WTIP | protein-coding | |
| MACS_peak_7646 | 368.05 | Intergenic | TMEM132D-AS1 | ncRNA |  |
| MACS_peak_24855 | 368.03 | TTS (NR_104606) | LINC02212 | ncRNA |  |
| MACS_peak_13988 | 367.64 | intron (NM_001082575, intron 2 of 14) | RBFOX3 | protein-coding | |
| MACS_peak_25963 | 367.52 | promoter-TSS (NM_003509) | HIST1H2BL | protein-coding | |
| MACS_peak_11116 | 367.38 | TTS (NR_106766).2 | LOC100288162 | ncRNA |  |
| MACS_peak_7115 | 367.21 | promoter-TSS (NM_001257401) | CD63 | protein-coding | |
| MACS_peak_28684 | 367.19 | intron (NM_001084, intron 8 of 18) | PLOD3 | protein-coding | |
| MACS_peak_930 | 367.01 | intron (NM_001350151, intron 1 of 9) | FBLIM1 | protein-coding | |
| MACS_peak_13740 | 366.88 | Intergenic | CD300A | protein-coding | |
| MACS_peak_24625 | 366.77 | intron (NR_003713, intron 2 of 3) | SDHAP3 | pseudo |  |
| MACS_peak_17086 | 366.55 | 5' UTR (NM_001316978, exon 1 of 3) | ZBTB45 | protein-coding | |
| MACS_peak_646 | 366.5 | intron (NM_001349608, intron 5 of 21) | CAMTA1 | protein-coding | |
| MACS_peak_8390 | 366.34 | intron (NM_024719, intron 4 of 7) | GRTP1-AS1 | ncRNA |  |
| MACS_peak_6296 | 366.31 | Intergenic | MMP12 | protein-coding | |
| MACS_peak_5305 | 366.12 | intron (NM_052854, intron 1 of 11) | CREB3L1 | protein-coding | |
| MACS_peak_15484 | 366.01 | intron (NM_001130065, intron 31 of 39) | USE1 | protein-coding | |
| MACS_peak_12134 | 366.01 | intron (NM_001242885, intron 2 of 2) | LOC100287036 | protein-coding | |
| MACS_peak_26935 | 365.93 | Intergenic | UNCX | protein-coding | |
| MACS_peak_27777 | 365.87 | intron (NM_001129, intron 6 of 20) | MIR4649 | ncRNA |  |
| MACS_peak_14557 | 365.76 | Intergenic | CYP4F35P | pseudo |  |
| MACS_peak_21793 | 365.58 | intron (NM_032173, intron 1 of 8) | ZNRF3 | protein-coding | |
| MACS_peak_22003 | 365.55 | intron (NM_002872, intron 5 of 6) | RAC2 | protein-coding | |
| MACS_peak_28631 | 365.48 | Intergenic | NYAP1 | protein-coding | |
| MACS_peak_1182 | 365.4 | Intergenic |  |  |  |
| MACS_peak_28844 | 365.35 | Intergenic | LINC02476 | ncRNA |  |
| MACS_peak_23267 | 365.26 | promoter-TSS (NM_024610) | HSPBAP1 | protein-coding | |
| MACS_peak_6158 | 365.21 | intron (NM_001098816, intron 2 of 33) | MIR708 | ncRNA |  |
| MACS_peak_21494 | 365.21 | non-coding (NR_146277, exon 6 of 18) | TOP3B | protein-coding | |
| MACS_peak_8656 | 365.12 | Intergenic | LINC00648 | ncRNA |  |
| MACS_peak_16368 | 365.1 | intron (NM_006509, intron 1 of 10) | RELB | protein-coding | |
| MACS_peak_20495 | 364.87 | intron (NR_038328, intron 2 of 3) | TEKT4P2 | pseudo |  |
| MACS_peak_10932 | 364.79 | promoter-TSS (NM_001290097) | TMEM114 | protein-coding | |
| MACS_peak_27910 | 364.74 | intron (NM_022748, intron 3 of 30) | SNORD151 | snoRNA |  |
| MACS_peak_7828 | 364.67 | promoter-TSS (NM_183238) | ZNF605 | protein-coding | |
| MACS_peak_27878 | 364.57 | 3' UTR (NM_021116, exon 20 of 20) | SEPTIN7P2 | pseudo |  |
| MACS_peak_15302 | 364.54 | exon (NM_004907, exon 2 of 2) | IER2 | protein-coding | |
| MACS_peak_27322 | 364.32 | intron (NM_001322007, intron 13 of 13) | RSPH10B2 | protein-coding | |
| MACS_peak_18891 | 364.31 | intron (NM_005018, intron 2 of 4) | PDCD1 | protein-coding | |
| MACS_peak_3485 | 364.21 | intron (NM_006642, intron 1 of 17).2 | SDCCAG8 | protein-coding | |
| MACS_peak_28050 | 364.19 | Intergenic | MIR4283-2 | ncRNA |  |
| MACS_peak_31592 | 364.13 | intron (NM_001278074, intron 41 of 65) | MIR3689C | ncRNA |  |
| MACS_peak_5651 | 363.96 | promoter-TSS (NM_001271849) | CDK2AP2 | protein-coding | |
| MACS_peak_3444 | 363.94 | intron (NM_000081, intron 48 of 52) | GNG4 | protein-coding | |
| MACS_peak_2725 | 363.86 | Intergenic | C1orf226 | protein-coding | |
| MACS_peak_5015 | 363.79 | intron (NM_001200049, intron 44 of 57) | NKX6-2 | protein-coding | |
| MACS_peak_27012 | 363.59 | Intergenic | ELFN1 | protein-coding | |
| MACS_peak_32030 | 363.49 | Intergenic | LOC105379514 | ncRNA |  |
| MACS_peak_1544 | 363.38 | intron (NM_017646, intron 1 of 10) | TRIT1 | protein-coding | |
| MACS_peak_18376 | 363.22 | Intergenic | GPR39 | protein-coding | |
| MACS_peak_6292 | 363.18 | Intergenic | MMP10 | protein-coding | |
| MACS_peak_14350 | 363.17 | non-coding (NR_003682, exon 1 of 1) | MGC70870 | pseudo |  |
| MACS_peak_24036 | 363.06 | intron (NM_018986, intron 12 of 17) | SH3TC1 | protein-coding | |
| MACS_peak_22155 | 363 | intron (NM_001003406, intron 9 of 35) | CACNA1I | protein-coding | |
| MACS_peak_978 | 362.76 | intron (NR_026752, intron 5 of 6) | CROCCP2 | pseudo |  |
| MACS_peak_6143 | 362.66 | intron (NM_020798, intron 3 of 10) | USP35 | protein-coding | |
| MACS_peak_17233 | 362.56 | promoter-TSS (NM_005742) | PDIA6 | protein-coding | |
| MACS_peak_16981 | 362.43 | TTS (NM_001145402) | COX6B2 | protein-coding | |
| MACS_peak_22850 | 362.27 | 5' UTR (NM_002971, exon 1 of 11) | SATB1 | protein-coding | |
| MACS_peak_28393 | 362.14 | intron (NR_146079, intron 3 of 9).2 | SPDYE11 | protein-coding | |
| MACS_peak_2080 | 361.93 | promoter-TSS (NM_024494) | WNT2B | protein-coding | |
| MACS_peak_12569 | 361.85 | TTS (NR_006880) | SNORD3A | snoRNA |  |
| MACS_peak_19536 | 361.85 | intron (NM_000961, intron 4 of 9) | PTGIS | protein-coding | |
| MACS_peak_20177 | 361.77 | intron (NM_001302643, intron 8 of 13) | SLC17A9 | protein-coding | |
| MACS_peak_17340 | 361.73 | intron (NM_052920, intron 3 of 13) | KLHL29 | protein-coding | |
| MACS_peak_3944 | 361.5 | Intergenic | BMS1P1 | pseudo |  |
| MACS_peak_14297 | 361.48 | intron (NM_006822, intron 2 of 5) | MIR4525 | ncRNA |  |
| MACS_peak_5550 | 361.44 | intron (NM_001300857, intron 1 of 1) | CCDC85B | protein-coding | |
| MACS_peak_26090 | 361.03 | TTS (NR_029633).6 | RING1 | protein-coding | |
| MACS_peak_23454 | 360.99 | promoter-TSS (NM_005067) | SIAH2 | protein-coding | |
| MACS_peak_26788 | 360.92 | Intergenic | FAM20C | protein-coding | |
| MACS_peak_16983 | 360.9 | intron (NM_001267718, intron 3 of 3) | FAM71E2 | protein-coding | |
| MACS_peak_29149 | 360.84 | promoter-TSS (NM_001304419) | IQCA1L | protein-coding | |
| MACS_peak_4002 | 360.54 | intron (NR_144642, intron 8 of 9) | ARHGAP22 | protein-coding | |
| MACS_peak_438 | 360.48 | intron (NM_001409, intron 4 of 36) | MIR551A | ncRNA |  |
| MACS_peak_22178 | 360.31 | Intergenic | MCHR1 | protein-coding | |
| MACS_peak_19761 | 359.94 | intron (NM_017495, intron 3 of 3) | LOC100291105 | ncRNA |  |
| MACS_peak_20306 | 359.87 | intron (NM_003823, intron 2 of 2) | TNFRSF6B | protein-coding | |
| MACS_peak_24636 | 359.79 | Intergenic | MIR4277 | ncRNA |  |
| MACS_peak_21549 | 359.73 | intron (NM_002073, intron 2 of 2) | RSPH14 | protein-coding | |
| MACS_peak_26085 | 359.56 | exon (NM_080680, exon 16 of 66) | COL11A2 | protein-coding | |
| MACS_peak_14489 | 359.48 | Intergenic | C18orf61 | ncRNA |  |
| MACS_peak_31753 | 359.26 | non-coding (NR_135288, exon 1 of 3) | LINC01451 | ncRNA |  |
| MACS_peak_21972 | 359.2 | intron (NM_000395, intron 1 of 13) | CSF2RB | protein-coding | |
| MACS_peak_8492 | 359.06 | Intergenic | POTEG | protein-coding | |
| MACS_peak_24722 | 359.01 | intron (NM_024337, intron 3 of 3) | IRX1 | protein-coding | |
| MACS_peak_17220 | 358.71 | intron (NM_001258359, intron 2 of 4) | HPCAL1 | protein-coding | |
| MACS_peak_11944 | 358.34 | Intergenic | LOC101928708 | ncRNA |  |
| MACS_peak_21259 | 358.23 | Intergenic | ZDHHC8 | protein-coding | |
| MACS_peak_21006 | 358.1 | Intergenic | HDHD5 | protein-coding | |
| MACS_peak_3615 | 358.03 | Intergenic | LINC00701 | ncRNA |  |
| MACS_peak_14124 | 357.93 | TTS (NM_017450) | MIR657 | ncRNA |  |
| MACS_peak_16299 | 357.59 | intron (NM_001304815, intron 2 of 20) | CIC | protein-coding | |
| MACS_peak_9388 | 357.31 | NA |  |  |  |
| MACS_peak_17793 | 357.31 | non-coding (NR_120420, exon 2 of 5) | LOC100507006 | ncRNA |  |
| MACS_peak_13459 | 357.27 | intron (NM_001085430, intron 7 of 7) | C17orf67 | protein-coding | |
| MACS_peak_28825 | 357.22 | intron (NM_001363540, intron 38 of 52) | DOCK4-AS1 | ncRNA |  |
| MACS_peak_18287 | 357.11 | exon (NM_032390, exon 1 of 7) | NIFK | protein-coding | |
| MACS_peak_3251 | 357.08 | intron (NM_194442, intron 13 of 13) | LBR | protein-coding | |
| MACS_peak_31150 | 357.04 | exon (NM_001261, exon 7 of 7) | CDK9 | protein-coding | |
| MACS_peak_26595 | 357.02 | intron (NM_007044, intron 4 of 10) | RPS18P9 | pseudo |  |
| MACS_peak_23942 | 357.02 | Intergenic | ADRA2C | protein-coding | |
| MACS_peak_27520 | 356.94 | promoter-TSS (NR_038367) | HOXA1 | protein-coding | |
| MACS_peak_19484 | 356.74 | Intergenic | LINC01522 | ncRNA |  |
| MACS_peak_21154 | 356.66 | intron (NM_007098, intron 22 of 32) | SLC25A1 | protein-coding | |
| MACS_peak_20311 | 356.43 | intron (NM_181485, intron 2 of 6) | LIME1 | protein-coding | |
| MACS_peak_25747 | 356.37 | Intergenic | IRF4 | protein-coding | |
| MACS_peak_6191 | 356.3 | exon (NM_022918, exon 1 of 15) | TMEM135 | protein-coding | |
| MACS_peak_10061 | 356.29 | intron (NM_001301192, intron 2 of 4) | LINGO1 | protein-coding | |
| MACS_peak_2225 | 356.19 | intron (NR_104189, intron 2 of 3).2 | LSP1P5 | pseudo |  |
| MACS_peak_7474 | 356.18 | intron (NM_001171993, intron 3 of 15) | HPD | protein-coding | |
| MACS_peak_4266 | 356.16 | intron (NM_052836, intron 6 of 12) | CDH23-AS1 | ncRNA |  |
| MACS_peak_16030 | 356.12 | exon (NM_001039673, exon 3 of 8) | C19orf33 | protein-coding | |
| MACS_peak_24025 | 356.04 | intron (NM_001130084, intron 4 of 19) | ABLIM2 | protein-coding | |
| MACS_peak_14315 | 355.99 | intron (NM_005993, intron 17 of 38) | ZNF750 | protein-coding | |
| MACS_peak_13598 | 355.98 | intron (NM_000442, intron 14 of 15) | PECAM1 | protein-coding | |
| MACS_peak_8005 | 355.87 | intron (NM_198441, intron 1 of 2).2 | PRR20A | protein-coding | |
| MACS_peak_8115 | 355.79 | Intergenic | LINC00430 | ncRNA |  |
| MACS_peak_30604 | 355.75 | Intergenic | LOC105379252 | ncRNA |  |
| MACS_peak_29112 | 355.73 | intron (NM_001351028, intron 6 of 9) | ACTR3C | protein-coding | |
| MACS_peak_16849 | 355.31 | intron (NM_001316994, intron 1 of 8) | SPACA6 | protein-coding | |
| MACS_peak_28413 | 355.19 | non-coding (NR_033322, exon 1 of 11) | NSUN5P1 | pseudo |  |
| MACS_peak_1939 | 355.19 | promoter-TSS (NM_032184) | SYDE2 | protein-coding | |
| MACS_peak_17806 | 355.06 | promoter-TSS (NM_004161) | RAB1A | protein-coding | |
| MACS_peak_27914 | 355.05 | intron (NM_022748, intron 1 of 30) | TNS3 | protein-coding | |
| MACS_peak_5091 | 354.95 | promoter-TSS (NM_007183) | PKP3 | protein-coding | |
| MACS_peak_20063 | 354.91 | exon (NM_005560, exon 64 of 80) | ADRM1 | protein-coding | |
| MACS_peak_31870 | 354.74 | intron (NM_017820, intron 19 of 21) | NRARP | protein-coding | |
| MACS_peak_27005 | 354.49 | Intergenic | TFAMP1 | pseudo |  |
| MACS_peak_30314 | 354.37 | exon (NM_001134374, exon 3 of 4) | SMPD5 | pseudo |  |
| MACS_peak_7009 | 354.31 | intron (NM_182507, intron 2 of 8) | KRT80 | protein-coding | |
| MACS_peak_5389 | 353.85 | intron (NM_001079808, intron 4 of 8) | PGA4 | protein-coding | |
| MACS_peak_30522 | 353.81 | Intergenic | FAM95B1 | ncRNA |  |
| MACS_peak_8182 | 353.8 | Intergenic | DOCK9-DT | ncRNA |  |
| MACS_peak_3178 | 353.76 | 5' UTR (NM_001040619, exon 1 of 4) | ATF3 | protein-coding | |
| MACS_peak_2308 | 353.69 | intron (NR_073513, intron 6 of 13) | MIR6878 | ncRNA |  |
| MACS_peak_7993 | 353.69 | Intergenic | MIR5007 | ncRNA |  |
| MACS_peak_30674 | 353.37 | intron (NR_149022, intron 3 of 3) | SPATA31E1 | protein-coding | |
| MACS_peak_17145 | 353.33 | intron (NM_015025, intron 21 of 24) | PXDN | protein-coding | |
| MACS_peak_24377 | 353.32 | non-coding (NR_121644, exon 1 of 1) | DUX4 | protein-coding | |
| MACS_peak_12259 | 353.3 | intron (NM_178568, intron 1 of 1) | LOC105371485 | ncRNA |  |
| MACS_peak_27807 | 353.29 | Intergenic | CAMK2B | protein-coding | |
| MACS_peak_31872 | 353.26 | intron (NM_017820, intron 19 of 21) | NRARP | protein-coding | |
| MACS_peak_12914 | 353.19 | Intergenic | TBC1D3E | protein-coding | |
| MACS_peak_13630 | 353.03 | intron (NM_002737, intron 3 of 16) | PRKCA-AS1 | ncRNA |  |
| MACS_peak_20760 | 353.01 | Intergenic | LINC00322 | ncRNA |  |
| MACS_peak_23499 | 352.98 | Intergenic | MIR1263 | ncRNA |  |
| MACS_peak_10516 | 352.89 | intron (NM_001370401, intron 6 of 14).2 | RAB11FIP3 | protein-coding | |
| MACS_peak_18714 | 352.79 | intron (NM_018410, intron 1 of 8) | HJURP | protein-coding | |
| MACS_peak_16416 | 352.54 | intron (NM_003370, intron 10 of 12) | VASP | protein-coding | |
| MACS_peak_28731 | 352.4 | TTS (NR_036245) | MIR4285 | ncRNA |  |
| MACS_peak_32445 | 352.39 | Intergenic | WASIR1 | ncRNA |  |
| MACS_peak_25001 | 352.29 | Intergenic | LINC02211 | ncRNA |  |
| MACS_peak_29400 | 352.24 | intron (NM_001308153, intron 12 of 29).2 | ARHGEF10 | protein-coding | |
| MACS_peak_27798 | 352.09 | intron (NM_172081, intron 5 of 18) | YKT6 | protein-coding | |
| MACS_peak_18127 | 351.89 | Intergenic | TMEM131 | protein-coding | |
| MACS_peak_10660 | 351.88 | intron (NM_001365310, intron 1 of 1) | LOC105371045 | protein-coding | |
| MACS_peak_23827 | 351.71 | Intergenic | FGFRL1 | protein-coding | |
| MACS_peak_683 | 351.51 | exon (NM_012102, exon 19 of 24) | SLC45A1 | protein-coding | |
| MACS_peak_15661 | 351.27 | intron (NM_004386, intron 1 of 14) | NCAN | protein-coding | |
| MACS_peak_11142 | 351.1 | Intergenic | XYLT1 | protein-coding | |
| MACS_peak_10664 | 351.09 | intron (NM_001287, intron 7 of 24) | CCDC154 | protein-coding | |
| MACS_peak_24545 | 351.04 | intron (NM_006598, intron 22 of 23).2 | MIR4635 | ncRNA |  |
| MACS_peak_7423 | 350.97 | intron (NM_002859, intron 1 of 10) | PXN | protein-coding | |
| MACS_peak_9461 | 350.87 | Intergenic | GOLGA8EP | pseudo |  |
| MACS_peak_1313 | 350.85 | Intergenic | FGR | protein-coding | |
| MACS_peak_15587 | 350.79 | Intergenic | SSBP4 | protein-coding | |
| MACS_peak_17079 | 350.69 | Intergenic | MIR4754 | ncRNA |  |
| MACS_peak_137 | 350.6 | 3' UTR (NM_030649, exon 24 of 24) | MIR6726 | ncRNA |  |
| MACS_peak_27593 | 350.59 | exon (NM_032222, exon 17 of 18) | AQP1 | protein-coding | |
| MACS_peak_20302 | 350.51 | intron (NR_037882, intron 12 of 37) | TNFRSF6B | protein-coding | |
| MACS_peak_10024 | 350.36 | Intergenic | ODF3L1 | protein-coding | |
| MACS_peak_5421 | 350.32 | intron (NM_001271686, intron 1 of 8) | RAB3IL1 | protein-coding | |
| MACS_peak_28860 | 350.27 | Intergenic | LOC101928254 | ncRNA |  |
| MACS_peak_9878 | 350.26 | intron (NM_014249, intron 7 of 7) | NR2E3 | protein-coding | |
| MACS_peak_30250 | 349.94 | 3' UTR (NM_001100878, exon 14 of 14).2 | MROH6 | protein-coding | |
| MACS_peak_12890 | 349.9 | intron (NM_198839, intron 4 of 59).2 | ACACA | protein-coding | |
| MACS_peak_27869 | 349.69 | intron (NM_001281768, intron 6 of 9) | ADCY1 | protein-coding | |
| MACS_peak_4192 | 349.59 | intron (NM_014431, intron 1 of 19) | PALD1 | protein-coding | |
| MACS_peak_12444 | 349.49 | intron (NM_001256012, intron 1 of 42) | MYH10 | protein-coding | |
| MACS_peak_5170 | 349.33 | Intergenic | CSNK2A3 | protein-coding | |
| MACS_peak_13154 | 349.23 | Intergenic | RNU2-1 | snRNA |  |
| MACS_peak_10549 | 348.95 | intron (NM_024042, intron 2 of 3) | METRN | protein-coding | |
| MACS_peak_4224 | 348.91 | Intergenic | LINC02622 | ncRNA |  |
| MACS_peak_12910 | 348.9 | promoter-TSS (NM_001291465).15 | TBC1D3C | protein-coding | |
| MACS_peak_32325 | 348.75 | Intergenic | HSFX1 | protein-coding | |
| MACS_peak_15300 | 348.62 | promoter-TSS (NM_004907) | STX10 | protein-coding | |
| MACS_peak_11294 | 348.52 | intron (NM_145659, intron 1 of 4) | IL27 | protein-coding | |
| MACS_peak_6007 | 348.44 | intron (NM_032871, intron 1 of 10) | RELT | protein-coding | |
| MACS_peak_1837 | 348.43 | intron (NM_015306, intron 1 of 67) | USP24 | protein-coding | |
| MACS_peak_24151 | 348.41 | Intergenic | CWH43 | protein-coding | |
| MACS_peak_2971 | 348.34 | intron (NM_001017403, intron 3 of 17) | LGR6 | protein-coding | |
| MACS_peak_10644 | 348.28 | Intergenic | UBE2I | protein-coding | |
| MACS_peak_16370 | 348.24 | intron (NR_103529, intron 5 of 20) | CLASRP | protein-coding | |
| MACS_peak_18205 | 348.16 | intron (NM_001123363, intron 10 of 23).2 | RGPD5 | protein-coding | |
| MACS_peak_20995 | 348.11 | Intergenic | GAB4 | protein-coding | |
| MACS_peak_11696 | 348.1 | intron (NM_001170720, intron 1 of 7) | BCAR1 | protein-coding | |
| MACS_peak_30680 | 348.08 | intron (NR_149022, intron 1 of 3) | LOC497256 | ncRNA |  |
| MACS_peak_21067 | 347.89 | intron (NR_003563, intron 14 of 14) | TMEM191B | protein-coding | |
| MACS_peak_16759 | 347.73 | intron (NR_110912, intron 2 of 3) | KCNC3 | protein-coding | |
| MACS_peak_22603 | 347.58 | Intergenic | MIR4790 | ncRNA |  |
| MACS_peak_5679 | 347.58 | intron (NM_030930, intron 7 of 10) | UNC93B1 | protein-coding | |
| MACS_peak_14219 | 347.45 | intron (NM_207368, intron 3 of 4) | MCRIP1 | protein-coding | |
| MACS_peak_21911 | 347.34 | intron (NM_003490, intron 6 of 13) | TIMP3 | protein-coding | |
| MACS_peak_16160 | 347.32 | intron (NM_001031696, intron 9 of 12) | MIR6796 | ncRNA |  |
| MACS_peak_19762 | 347.24 | intron (NM_017495, intron 3 of 3) | LOC100291105 | ncRNA |  |
| MACS_peak_7402 | 347.2 | intron (NM_001346490, intron 1 of 21) | TAOK3 | protein-coding | |
| MACS_peak_27405 | 347.19 | intron (NR_156701, intron 8 of 14) | SCIN | protein-coding | |
| MACS_peak_18609 | 346.98 | Intergenic | ASIC4 | protein-coding | |
| MACS_peak_31802 | 346.91 | intron (NM_153200, intron 1 of 3) | EDF1 | protein-coding | |
| MACS_peak_23362 | 346.89 | promoter-TSS (NM_001204888) | RAB43 | protein-coding | |
| MACS_peak_9469 | 346.85 | Intergenic | GOLGA8EP | pseudo |  |
| MACS_peak_28727 | 346.78 | 3' UTR (NM_181500, exon 23 of 23) | SH2B2 | protein-coding | |
| MACS_peak_10804 | 346.64 | Intergenic | CASP16P | pseudo |  |
| MACS_peak_25566 | 346.27 | promoter-TSS (NM_004417) | DUSP1 | protein-coding | |
| MACS_peak_22123 | 346.25 | intron (NM_002608, intron 2 of 6) | PDGFB | protein-coding | |
| MACS_peak_7080 | 346.2 | intron (NM_014620, intron 1 of 3) | MIR615 | ncRNA |  |
| MACS_peak_29625 | 346.05 | promoter-TSS (NM_001277971) | POMK | protein-coding | |
| MACS_peak_7471 | 346.05 | intron (NM_001353345, intron 15 of 15) | SETD1B | protein-coding | |
| MACS_peak_22128 | 345.99 | Intergenic | SNORD83B | snoRNA |  |
| MACS_peak_1262 | 345.98 | promoter-TSS (NR_037481) | STMN1 | protein-coding | |
| MACS_peak_28209 | 345.9 | Intergenic | SPDYE11 | protein-coding | |
| MACS_peak_14771 | 345.64 | intron (NM_138924, intron 2 of 4) | GAMT | protein-coding | |
| MACS_peak_22933 | 345.39 | promoter-TSS (NM_005808) | ITGA9-AS1 | ncRNA |  |
| MACS_peak_7462 | 345.27 | intron (NM_001080825, intron 4 of 11) | TMEM120B | protein-coding | |
| MACS_peak_14262 | 345.11 | intron (NM_001367828, intron 3 of 16) | CCDC57 | protein-coding | |
| MACS_peak_31697 | 345.09 | Intergenic | TMEM250 | protein-coding | |
| MACS_peak_6517 | 344.95 | intron (NM_015232, intron 1 of 12) | IQSEC3 | protein-coding | |
| MACS_peak_1240 | 344.88 | intron (NM_001320672, intron 4 of 6) | MIR6731 | ncRNA |  |
| MACS_peak_16502 | 344.81 | intron (NM_001321086, intron 5 of 7) | NPAS1 | protein-coding | |
| MACS_peak_31257 | 344.7 | intron (NR_120686, intron 2 of 3) | LINC01503 | ncRNA |  |
| MACS_peak_19143 | 344.4 | intron (NM_031232, intron 8 of 11) | C20orf144 | protein-coding | |
| MACS_peak_20762 | 344.28 | intron (NR_103713, intron 1 of 2) | LINC00322 | ncRNA |  |
| MACS_peak_14096 | 344.26 | intron (NM_001163034, intron 13 of 29) | LOC101928855 | ncRNA |  |
| MACS_peak_15408 | 344.21 | Intergenic | CYP4F8 | protein-coding | |
| MACS_peak_2508 | 344.2 | TTS (NM_005698) | FAM189B | protein-coding | |
| MACS_peak_21123 | 344 | Intergenic | DGCR5 | ncRNA |  |
| MACS_peak_16954 | 343.92 | intron (NM_001271618, intron 6 of 21) | EPS8L1 | protein-coding | |
| MACS_peak_16681 | 343.81 | promoter-TSS (NM_012423) | RPL13A | protein-coding | |
| MACS_peak_8766 | 343.65 | intron (NM_001244701, intron 2 of 2) | ZFP36L1 | protein-coding | |
| MACS_peak_5994 | 343.44 | Intergenic | ARHGEF17 | protein-coding | |
| MACS_peak_27576 | 343.36 | intron (NM_006092, intron 6 of 13) | NOD1 | protein-coding | |
| MACS_peak_15832 | 343.2 | Intergenic | CEBPA | protein-coding | |
| MACS_peak_544 | 343.06 | intron (NR_111987, intron 16 of 32) | MIR4689 | ncRNA |  |
| MACS_peak_553 | 342.95 | intron (NM_001199860, intron 1 of 15) | KCNAB2 | protein-coding | |
| MACS_peak_22137 | 342.94 | intron (NM_153497, intron 1 of 10) | TAB1 | protein-coding | |
| MACS_peak_28632 | 342.94 | promoter-TSS (NM_173564) | NYAP1 | protein-coding | |
| MACS_peak_14504 | 342.91 | Intergenic | LINC01882 | ncRNA |  |
| MACS_peak_14641 | 342.83 | Intergenic | LINC01002 | ncRNA |  |
| MACS_peak_2624 | 342.5 | Intergenic | ETV3 | protein-coding | |
| MACS_peak_22117 | 342.47 | Intergenic | APOBEC3H | protein-coding | |
| MACS_peak_8377 | 342.23 | intron (NM_001008895, intron 5 of 19) | CUL4A | protein-coding | |
| MACS_peak_14716 | 341.94 | intron (NM_001033026, intron 2 of 10) | TMEM259 | protein-coding | |
| MACS_peak_22513 | 341.85 | Intergenic | GGT3P | pseudo |  |
| MACS_peak_1298 | 341.72 | exon (NM_004672, exon 13 of 29) | MAP3K6 | protein-coding | |
| MACS_peak_7765 | 341.65 | intron (NM_001122636, intron 9 of 10) | GALNT9 | protein-coding | |
| MACS_peak_609 | 341.59 | intron (NM_198681, intron 1 of 21) | PLEKHG5 | protein-coding | |
| MACS_peak_10511 | 341.48 | intron (NM_014700, intron 1 of 13).2 | RAB11FIP3 | protein-coding | |
| MACS_peak_15712 | 341.45 | TTS (NR_110427) | LOC101929124 | ncRNA |  |
| MACS_peak_5729 | 341.36 | intron (NM_004923, intron 6 of 9) | GAL | protein-coding | |
| MACS_peak_27045 | 341.34 | intron (NM_001013837, intron 17 of 18) | MAD1L1 | protein-coding | |
| MACS_peak_21739 | 341.29 | intron (NM_182492, intron 1 of 3) | LRP5L | protein-coding | |
| MACS_peak_21160 | 341.13 | intron (NM_003325, intron 1 of 24) | HIRA | protein-coding | |
| MACS_peak_14729 | 341.08 | intron (NM_014963, intron 23 of 31) | GPX4 | protein-coding | |
| MACS_peak_20778 | 341.07 | intron (NM_003681, intron 1 of 10) | PDXK | protein-coding | |
| MACS_peak_14711 | 340.94 | intron (NM_024100, intron 2 of 9) | WDR18 | protein-coding | |
| MACS_peak_4635 | 340.86 | intron (NM_001290295, intron 1 of 24) | SORBS1 | protein-coding | |
| MACS_peak_9588 | 340.86 | intron (NM_001282484, intron 1 of 18).2 | GOLGA8R | protein-coding | |
| MACS_peak_6409 | 340.71 | exon (NM_002855, exon 6 of 6) | NECTIN1 | protein-coding | |
| MACS_peak_2156 | 340.66 | non-coding (NR_157049, exon 1 of 5) | NOTCH2NLR | pseudo |  |
| MACS_peak_14310 | 340.64 | intron (NM_005993, intron 11 of 38) | ZNF750 | protein-coding | |
| MACS_peak_24910 | 340.62 | intron (NM_007118, intron 7 of 56) | TRIO | protein-coding | |
| MACS_peak_16977 | 340.52 | intron (NR_104071, intron 6 of 6) | TMEM150B | protein-coding | |
| MACS_peak_6384 | 340.5 | promoter-TSS (NR_039713) | BCL9L | protein-coding | |
| MACS_peak_5334 | 340.34 | intron (NM_001080547, intron 4 of 4) | MYBPC3 | protein-coding | |
| MACS_peak_3252 | 340.19 | intron (NM_001008493, intron 1 of 14) | ENAH | protein-coding | |
| MACS_peak_9382 | 340.12 | NA |  |  |  |
| MACS_peak_9431 | 340.07 | intron (NR_027411, intron 13 of 17) | GOLGA8CP | pseudo |  |
| MACS_peak_996 | 340.05 | Intergenic | RNU1-2 | snRNA |  |
| MACS_peak_9393 | 340.01 | NA |  |  |  |
| MACS_peak_5387 | 339.85 | intron (NM_001079807, intron 4 of 8) | PGA3 | protein-coding | |
| MACS_peak_21224 | 339.78 | intron (NR_147957, intron 7 of 16) | TXNRD2 | protein-coding | |
| MACS_peak_20230 | 339.78 | intron (NM_020882, intron 15 of 35) | COL20A1 | protein-coding | |
| MACS_peak_4216 | 339.78 | Intergenic | PCBD1 | protein-coding | |
| MACS_peak_16637 | 339.54 | TTS (NM_138764) | FTL | protein-coding | |
| MACS_peak_12903 | 339.47 | promoter-TSS (NM_001291465).5 | TBC1D3C | protein-coding | |
| MACS_peak_32384 | 339.31 | intron (NM_001395, intron 1 of 3) | DUSP9 | protein-coding | |
| MACS_peak_24434 | 339.29 | intron (NM_020731, intron 2 of 11) | AHRR | protein-coding | |
| MACS_peak_13543 | 339.21 | intron (NR_027486, intron 13 of 13) | TBC1D3P2 | pseudo |  |
| MACS_peak_37 | 339.05 | intron (NR_047524, intron 2 of 3) | LINC01128 | ncRNA |  |
| MACS_peak_10415 | 339.04 | promoter-TSS (NM_001284417) | LYSMD4 | protein-coding | |
| MACS_peak_11090 | 338.94 | intron (NM_001040113, intron 41 of 42) | NDE1 | protein-coding | |
| MACS_peak_26353 | 338.9 | Intergenic | MRPS18A | protein-coding | |
| MACS_peak_458 | 338.88 | intron (NM_001204185, intron 1 of 12) | TP73 | protein-coding | |
| MACS_peak_28707 | 338.65 | Intergenic | MYL10 | protein-coding | |
| MACS_peak_21383 | 338.39 | non-coding (NR_027006, exon 3 of 11) | LRRC74B | protein-coding | |
| MACS_peak_8008 | 338.36 | intron (NM_198441, intron 1 of 2).3 | PRR20C | protein-coding | |
| MACS_peak_4598 | 338.19 | promoter-TSS (NM_001099338) | NUTM2A | protein-coding | |
| MACS_peak_21243 | 337.97 | promoter-TSS (NM_001322146) | TANGO2 | protein-coding | |
| MACS_peak_5957 | 337.86 | intron (NM_001143839, intron 1 of 29) | MIR139 | ncRNA |  |
| MACS_peak_11648 | 337.67 | intron (NM_001322117, intron 2 of 18) | PDPR | protein-coding | |
| MACS_peak_17453 | 337.64 | exon (NM_004341, exon 37 of 44) | SLC30A3 | protein-coding | |
| MACS_peak_29358 | 337.6 | Intergenic | OR4F21 | protein-coding | |
| MACS_peak_5590 | 337.45 | promoter-TSS (NM_031492) | RBM4B | protein-coding | |
| MACS_peak_20269 | 337.29 | intron (NM_001958, intron 6 of 7) | EEF1A2 | protein-coding | |
| MACS_peak_28142 | 337.25 | Intergenic | GS1-124K5.4 | ncRNA |  |
| MACS_peak_22754 | 337.23 | 5' UTR (NM_001165035, exon 1 of 18) | FBLN2 | protein-coding | |
| MACS_peak_21414 | 337.22 | Intergenic | RIMBP3C | protein-coding | |
| MACS_peak_12031 | 337.2 | intron (NM_153813, intron 3 of 9) | LOC100128882 | ncRNA |  |
| MACS_peak_21262 | 336.94 | exon (NM_001365892, exon 2 of 9) | CCDC188 | protein-coding | |
| MACS_peak_23893 | 336.93 | intron (NM_002938, intron 4 of 7) | FAM193A | protein-coding | |
| MACS_peak_31867 | 336.88 | intron (NM_017820, intron 19 of 21) | NRARP | protein-coding | |
| MACS_peak_19151 | 336.78 | intron (NM_176812, intron 4 of 4) | CHMP4B | protein-coding | |
| MACS_peak_16326 | 336.64 | intron (NM_006297, intron 12 of 16) | ZNF575 | protein-coding | |
| MACS_peak_21938 | 336.62 | Intergenic | RASD2 | protein-coding | |
| MACS_peak_31648 | 336.55 | exon (NM_020822, exon 1 of 31) | KCNT1 | protein-coding | |
| MACS_peak_3629 | 336.49 | Intergenic | LOC105376360 | ncRNA |  |
| MACS_peak_31460 | 336.23 | promoter-TSS (NM_020469) | ABO | protein-coding | |
| MACS_peak_21916 | 336.2 | intron (NM_003490, intron 2 of 13) | SYN3 | protein-coding | |
| MACS_peak_2154 | 336.17 | intron (NR_144517, intron 4 of 41) | LOC100996724 | pseudo |  |
| MACS_peak_10449 | 336.04 | Intergenic | CHSY1 | protein-coding | |
| MACS_peak_9683 | 335.97 | promoter-TSS (NM_001198588) | JMJD7-PLA2G4B | protein-coding | |
| MACS_peak_3940 | 335.96 | promoter-TSS (NM_031912) | SYT15 | protein-coding | |
| MACS_peak_29979 | 335.88 | intron (NM_152888, intron 22 of 64) | COL22A1 | protein-coding | |
| MACS_peak_23999 | 335.84 | promoter-TSS (NM_020777) | SORCS2 | protein-coding | |
| MACS_peak_13568 | 335.63 | intron (NM_030779, intron 5 of 13) | KCNH6 | protein-coding | |
| MACS_peak_30274 | 335.56 | promoter-TSS (NM_198488) | FAM83H-AS1 | ncRNA |  |
| MACS_peak_29162 | 335.49 | Intergenic | CRYGN | protein-coding | |
| MACS_peak_16385 | 335.43 | exon (NM_138568, exon 6 of 10) | EXOC3L2 | protein-coding | |
| MACS_peak_551 | 335.23 | intron (NM_172130, intron 1 of 14) | KCNAB2 | protein-coding | |
| MACS_peak_15939 | 335.21 | promoter-TSS (NM_052948) | ARHGAP33 | protein-coding | |
| MACS_peak_23483 | 335.11 | intron (NM_001369781, intron 1 of 5) | MLF1 | protein-coding | |
| MACS_peak_27270 | 335.06 | exon (NM_001080495, exon 7 of 30) | TNRC18 | protein-coding | |
| MACS_peak_2412 | 334.93 | promoter-TSS (NR_126565) | LOC343052 | pseudo |  |
| MACS_peak_19366 | 334.86 | promoter-TSS (NR_037419) | MIR3646 | ncRNA |  |
| MACS_peak_11609 | 334.77 | intron (NM_001323627, intron 8 of 11) | TPPP3 | protein-coding | |
| MACS_peak_17475 | 334.7 | Intergenic | FOSL2 | protein-coding | |
| MACS_peak_1034 | 334.66 | intron (NR_137287, intron 4 of 29) | ARHGEF10L | protein-coding | |
| MACS_peak_30044 | 334.62 | Intergenic | LINC01300 | ncRNA |  |
| MACS_peak_13376 | 334.54 | Intergenic | DLX4 | protein-coding | |
| MACS_peak_18 | 334.52 | Intergenic | OR4F3 | protein-coding | |
| MACS_peak_7755 | 334.49 | intron (NM_175066, intron 2 of 14) | DDX51 | protein-coding | |
| MACS_peak_10722 | 334.43 | intron (NM_020764, intron 10 of 19) | CASKIN1 | protein-coding | |
| MACS_peak_3610 | 334.36 | intron (NM_018702, intron 1 of 9) | ADARB2 | protein-coding | |
| MACS_peak_12449 | 334.21 | intron (NM_004822, intron 2 of 6) | NTN1 | protein-coding | |
| MACS_peak_12901 | 333.88 | promoter-TSS (NM_001291465).19 | TBC1D3H | protein-coding | |
| MACS_peak_21 | 333.81 | Intergenic | LOC100132062 | ncRNA |  |
| MACS_peak_10651 | 333.75 | TTS (NM_001276414) | GNPTG | protein-coding | |
| MACS_peak_21894 | 333.67 | promoter-TSS (NR_040114) | AP1B1P1 | pseudo |  |
| MACS_peak_9241 | 333.38 | Intergenic | LINC02280 | ncRNA |  |
| MACS_peak_10198 | 333.23 | Intergenic | LOC103171574 | ncRNA |  |
| MACS_peak_30061 | 333.12 | intron (NR_102364, intron 3 of 26) | MROH5 | protein-coding | |
| MACS_peak_7910 | 333.01 | intron (NM_001370329, intron 1 of 9) | ELF1 | protein-coding | |
| MACS_peak_20950 | 332.88 | Intergenic | FRG1FP | pseudo |  |
| MACS_peak_28523 | 332.75 | promoter-TSS (NM_001145306) | LOC101927497 | ncRNA |  |
| MACS_peak_16437 | 332.75 | intron (NM_001329633, intron 1 of 1) | FBXO46 | protein-coding | |
| MACS_peak_12856 | 332.71 | intron (NM_001369503, intron 13 of 13) | TBC1D3G | protein-coding | |
| MACS_peak_14327 | 332.56 | Intergenic | B3GNTL1 | protein-coding | |
| MACS_peak_14345 | 332.53 | Intergenic | RPL23AP87 | pseudo |  |
| MACS_peak_17635 | 332.45 | Intergenic | LINC02580 | ncRNA |  |
| MACS_peak_28063 | 332.45 | Intergenic | LOC105375297 | ncRNA |  |
| MACS_peak_27990 | 332.43 | intron (NM_001346941, intron 1 of 21) | EGFR | protein-coding | |
| MACS_peak_26677 | 332.32 | intron (NM_020133, intron 8 of 8) | AGPAT4-IT1 | ncRNA |  |
| MACS_peak_24419 | 332.29 | intron (NM_001267557, intron 2 of 3) | PDCD6 | protein-coding | |
| MACS_peak_21712 | 332.27 | Intergenic | TMEM211 | protein-coding | |
| MACS_peak_2468 | 332.26 | Intergenic | KCNN3 | protein-coding | |
| MACS_peak_24370 | 332.03 | Intergenic | LINC01099 | ncRNA |  |
| MACS_peak_16547 | 332.02 | exon (NM_015710, exon 7 of 13) | SNORD23 | snoRNA |  |
| MACS_peak_21783 | 331.89 | promoter-TSS (NM_001284278) | PITPNB | protein-coding | |
| MACS_peak_1641 | 331.89 | intron (NM_018150, intron 1 of 14) | RNF220 | protein-coding | |
| MACS_peak_578 | 331.81 | TTS (NM_001024598) | HES3 | protein-coding | |
| MACS_peak_28000 | 331.8 | 3' UTR (NM_001284282, exon 5 of 5) | VOPP1 | protein-coding | |
| MACS_peak_12544 | 331.74 | promoter-TSS (NR_146075) | KRT17P2 | pseudo |  |
| MACS_peak_10094 | 331.74 | promoter-TSS (NM_001102668) | PSMA4 | protein-coding | |
| MACS_peak_27982 | 331.72 | exon (NM_182595, exon 1 of 1) | POM121L12 | protein-coding | |
| MACS_peak_14170 | 331.71 | intron (NM_001291324, intron 1 of 27) | MIR4740 | ncRNA |  |
| MACS_peak_4483 | 331.65 | intron (NM_020338, intron 1 of 24) | ZMIZ1 | protein-coding | |
| MACS_peak_13509 | 331.64 | Intergenic | LOC653653 | pseudo |  |
| MACS_peak_19646 | 331.41 | intron (NM_006045, intron 9 of 27) | ATP9A | protein-coding | |
| MACS_peak_7321 | 331.22 | promoter-TSS (NR_104099) | VPS29 | protein-coding | |
| MACS_peak_15818 | 331.13 | intron (NM_019849, intron 7 of 10) | SLC7A10 | protein-coding | |
| MACS_peak_20467 | 331.1 | intron (NR_003287, intron 1 of 2) | MIR663A | ncRNA |  |
| MACS_peak_18957 | 330.88 | promoter-TSS (NM_001206491) | MAVS | protein-coding | |
| MACS_peak_9258 | 330.79 | 3' UTR (NM_022489, exon 23 of 23) | ADSSL1 | protein-coding | |
| MACS_peak_4213 | 330.78 | promoter-TSS (NM_003901) | SGPL1 | protein-coding | |
| MACS_peak_31789 | 330.74 | promoter-TSS (NM_001039374) | CCDC183 | protein-coding | |
| MACS_peak_13723 | 330.69 | Intergenic | RPL38 | protein-coding | |
| MACS_peak_21554 | 330.68 | intron (NM_001349878, intron 4 of 10) | RAB36 | protein-coding | |
| MACS_peak_23092 | 330.64 | intron (NR_134919, intron 1 of 19) | MST1R | protein-coding | |
| MACS_peak_23161 | 330.62 | intron (NM_015512, intron 34 of 77) | BAP1 | protein-coding | |
| MACS_peak_4654 | 330.61 | intron (NM_001206528, intron 8 of 14) | MIR3085 | ncRNA |  |
| MACS_peak_12955 | 330.57 | Intergenic | LINC02079 | ncRNA |  |
| MACS_peak_29110 | 330.5 | promoter-TSS (NM_001351027) | ACTR3C | protein-coding | |
| MACS_peak_4151 | 330.36 | Intergenic | FAM241B | protein-coding | |
| MACS_peak_30253 | 330.34 | intron (NM_001363146, intron 7 of 11) | NAPRT | protein-coding | |
| MACS_peak_3605 | 330.3 | intron (NM_018702, intron 1 of 9) | ADARB2-AS1 | ncRNA |  |
| MACS_peak_10870 | 330.22 | intron (NM_145253, intron 2 of 2) | UBALD1 | protein-coding | |
| MACS_peak_7805 | 330.21 | intron (NM_001142641, intron 4 of 16) | MIR6763 | ncRNA |  |
| MACS_peak_24609 | 330.16 | intron (NM_024830, intron 3 of 13) | MIR6075 | ncRNA |  |
| MACS_peak_9262 | 330.14 | Intergenic | SIVA1 | protein-coding | |
| MACS_peak_28056 | 330.01 | Intergenic | MIR4283-2 | ncRNA |  |
| MACS_peak_2239 | 330.01 | intron (NM_007053, intron 1 of 5) | CD160 | protein-coding | |
| MACS_peak_7738 | 329.97 | intron (NM_016155, intron 9 of 9) | MMP17 | protein-coding | |
| MACS_peak_32134 | 329.95 | Intergenic | EFNB1 | protein-coding | |
| MACS_peak_23800 | 329.88 | exon (NM_006651, exon 3 of 4) | LOC100129917 | ncRNA |  |
| MACS_peak_29132 | 329.82 | intron (NM_001160110, intron 8 of 13) | NOS3 | protein-coding | |
| MACS_peak_28138 | 329.79 | intron (NM_000048, intron 14 of 16) | ASL | protein-coding | |
| MACS_peak_14862 | 329.7 | Intergenic | GADD45B | protein-coding | |
| MACS_peak_25807 | 329.67 | promoter-TSS (NM_001166010) | ECI2 | protein-coding | |
| MACS_peak_28850 | 329.66 | intron (NM_001167940, intron 1 of 29) | CADPS2 | protein-coding | |
| MACS_peak_28625 | 329.62 | Intergenic | C7orf61 | protein-coding | |
| MACS_peak_31522 | 329.44 | intron (NM_001134398, intron 1 of 29) | VAV2 | protein-coding | |
| MACS_peak_10830 | 329.38 | intron (NM_004380, intron 30 of 30) | TRAP1 | protein-coding | |
| MACS_peak_10346 | 329.33 | promoter-TSS (NM_018668) | VPS33B | protein-coding | |
| MACS_peak_16029 | 329.33 | exon (NM_033520, exon 2 of 4) | C19orf33 | protein-coding | |
| MACS_peak_22244 | 329.29 | intron (NR_027779, intron 2 of 11) | TTLL1 | protein-coding | |
| MACS_peak_27280 | 329.27 | Intergenic | TNRC18 | protein-coding | |
| MACS_peak_26080 | 329.19 | intron (NM_080679, intron 51 of 62).3 | COL11A2 | protein-coding | |
| MACS_peak_6295 | 329.05 | Intergenic | MMP12 | protein-coding | |
| MACS_peak_9976 | 328.93 | intron (NM_001099436, intron 7 of 15) | MIR6882 | ncRNA |  |
| MACS_peak_10532 | 328.9 | intron (NM_021168, intron 1 of 5) | RAB40C | protein-coding | |
| MACS_peak_26299 | 328.71 | intron (NM_001300805, intron 2 of 3) | MDFI | protein-coding | |
| MACS_peak_15385 | 328.67 | intron (NM_000435, intron 17 of 32) | MIR6795 | ncRNA |  |
| MACS_peak_10080 | 328.57 | Intergenic | LOC440292 | pseudo |  |
| MACS_peak_30562 | 328.56 | Intergenic | FAM27C | ncRNA |  |
| MACS_peak_15952 | 328.55 | exon (NM_001321831, exon 6 of 12) | NFKBID | protein-coding | |
| MACS_peak_19945 | 328.51 | intron (NM_001794, intron 2 of 15) | CDH4 | protein-coding | |
| MACS_peak_24713 | 328.44 | Intergenic | LINC01377 | ncRNA |  |
| MACS_peak_15403 | 328.4 | promoter-TSS (NM_005858) | AKAP8 | protein-coding | |
| MACS_peak_9256 | 328.28 | exon (NM_022489, exon 12 of 23) | ADSSL1 | protein-coding | |
| MACS_peak_31185 | 328.2 | intron (NM_005564, intron 2 of 6) | LCN2 | protein-coding | |
| MACS_peak_9401 | 328.13 | Intergenic | CHEK2P2 | pseudo |  |
| MACS_peak_191 | 328.07 | intron (NR_147951, intron 2 of 2) | FNDC10 | protein-coding | |
| MACS_peak_4478 | 327.92 | intron (NM_020338, intron 1 of 24) | ZMIZ1 | protein-coding | |
| MACS_peak_7527 | 327.72 | intron (NR_136910, intron 2 of 8) | KMT5A | protein-coding | |
| MACS_peak_20271 | 327.69 | intron (NM_001958, intron 1 of 7) | EEF1A2 | protein-coding | |
| MACS_peak_11229 | 327.56 | exon (NM_001135865, exon 7 of 7) | OTOAP1 | pseudo |  |
| MACS_peak_22913 | 327.48 | Intergenic | TRIM71 | protein-coding | |
| MACS_peak_12931 | 327.47 | intron (NM_001199417, intron 23 of 23).2 | ARHGAP23 | protein-coding | |
| MACS_peak_12849 | 327.4 | 3' UTR (NM_001291465, exon 14 of 14).5 | TBC1D3I | protein-coding | |
| MACS_peak_6361 | 327.37 | intron (NM_020693, intron 3 of 32) | CEP164 | protein-coding | |
| MACS_peak_23950 | 327.28 | promoter-TSS (NR_130740) | FAM86EP | pseudo |  |
| MACS_peak_8541 | 327.18 | promoter-TSS (NR_046052) | THTPA | protein-coding | |
| MACS_peak_31420 | 327.14 | intron (NM_032536, intron 2 of 7) | NTNG2 | protein-coding | |
| MACS_peak_21881 | 327.1 | intron (NM_014338, intron 1 of 8) | PISD | protein-coding | |
| MACS_peak_17881 | 326.98 | non-coding (NR_138476, exon 15 of 15) | EMX1 | protein-coding | |
| MACS_peak_31531 | 326.89 | intron (NM_017588, intron 1 of 13) | WDR5 | protein-coding | |
| MACS_peak_2940 | 326.84 | Intergenic | TNNT2 | protein-coding | |
| MACS_peak_11029 | 326.68 | intron (NM_032167, intron 15 of 20) | SNX29 | protein-coding | |
| MACS_peak_27021 | 326.53 | intron (NM_001128636, intron 1 of 2) | ELFN1 | protein-coding | |
| MACS_peak_31654 | 326.46 | intron (NM_001272003, intron 2 of 30) | KCNT1 | protein-coding | |
| MACS_peak_20898 | 326.43 | promoter-TSS (NM_001352512) | SLC19A1 | protein-coding | |
| MACS_peak_9297 | 326.32 | intron (NM_001242789, intron 1 of 12) | BRF1 | protein-coding | |
| MACS_peak_21211 | 326.22 | intron (NM_053004, intron 3 of 7) | RTL10 | protein-coding | |
| MACS_peak_20775 | 326.15 | TTS (NR_152722).2 | LINC00319 | ncRNA |  |
| MACS_peak_22147 | 326.12 | Intergenic | CACNA1I | protein-coding | |
| MACS_peak_12984 | 326.04 | intron (NM_001005862, intron 4 of 29) | ERBB2 | protein-coding | |
| MACS_peak_31215 | 325.97 | exon (NM_001318015, exon 2 of 5) | ZDHHC12 | protein-coding | |
| MACS_peak_983 | 325.91 | Intergenic | RNU1-4 | snRNA |  |
| MACS_peak_19076 | 325.85 | intron (NM_001012644, intron 2 of 6) | DUSP15 | protein-coding | |
| MACS_peak_2956 | 325.74 | intron (NM_012134, intron 1 of 2) | SHISA4 | protein-coding | |
| MACS_peak_30283 | 325.72 | TTS (NR_030633) | MIR937 | ncRNA |  |
| MACS_peak_29310 | 325.28 | intron (NM_001308267, intron 2 of 21) | MIR595 | ncRNA |  |
| MACS_peak_2611 | 325.24 | intron (NM_001080471, intron 4 of 22) | PEAR1 | protein-coding | |
| MACS_peak_28078 | 325.24 | Intergenic | ZNF716 | protein-coding | |
| MACS_peak_31493 | 325.21 | intron (NM_000787, intron 7 of 11) | DBH-AS1 | ncRNA |  |
| MACS_peak_27311 | 325.2 | Intergenic | FSCN1 | protein-coding | |
| MACS_peak_16793 | 325.04 | intron (NM_016148, intron 15 of 23) | SHANK1 | protein-coding | |
| MACS_peak_18781 | 325.01 | intron (NM_006037, intron 6 of 26) | MGC16025 | ncRNA |  |
| MACS_peak_19137 | 324.92 | Intergenic | SNTA1 | protein-coding | |
| MACS_peak_4305 | 324.87 | intron (NM_014767, intron 11 of 11) | SPOCK2 | protein-coding | |
| MACS_peak_5226 | 324.85 | Intergenic | ARL14EP | protein-coding | |
| MACS_peak_32163 | 324.85 | intron (NM_016120, intron 1 of 3) | RLIM | protein-coding | |
| MACS_peak_28769 | 324.81 | intron (NM_001277335, intron 15 of 19).2 | POLR2J3 | protein-coding | |
| MACS_peak_11428 | 324.63 | intron (NM_182493, intron 5 of 12) | MYLK3 | protein-coding | |
| MACS_peak_342 | 324.63 | Intergenic | TTC34 | protein-coding | |
| MACS_peak_173 | 324.55 | intron (NM_031921, intron 7 of 15) | ATAD3B | protein-coding | |
| MACS_peak_12907 | 324.54 | intron (NM_001001418, intron 7 of 13) | TBC1D3D | protein-coding | |
| MACS_peak_11813 | 324.49 | Intergenic | ZDHHC7 | protein-coding | |
| MACS_peak_22518 | 324.49 | Intergenic | GGT3P | pseudo |  |
| MACS_peak_14176 | 324.33 | intron (NM_001291324, intron 2 of 27) | MIR3186 | ncRNA |  |
| MACS_peak_21232 | 324.33 | intron (NM_001135162, intron 1 of 5) | COMT | protein-coding | |
| MACS_peak_1651 | 324.27 | intron (NM_001319957, intron 5 of 13) | SNORD145 | snoRNA |  |
| MACS_peak_7985 | 324.26 | Intergenic | MIR759 | ncRNA |  |
| MACS_peak_6259 | 324.25 | Intergenic | ARHGAP42 | protein-coding | |
| MACS_peak_7999 | 324.24 | 5' UTR (NM_001130407, exon 1 of 3) | PRR20E | protein-coding | |
| MACS_peak_16462 | 324.21 | Intergenic | IGFL2-AS1 | ncRNA |  |
| MACS_peak_13266 | 324.1 | intron (NM_016835, intron 6 of 13).2 | STH | protein-coding | |
| MACS_peak_4180 | 323.92 | promoter-TSS (NM_207119) | LRRC20 | protein-coding | |
| MACS_peak_12905 | 323.9 | intron (NM_001369501, intron 2 of 11).3 | TBC1D3D | protein-coding | |
| MACS_peak_3723 | 323.84 | promoter-TSS (NM_001321007) | TRDMT1 | protein-coding | |
| MACS_peak_20292 | 323.64 | intron (NM_012384, intron 2 of 9) | GMEB2 | protein-coding | |
| MACS_peak_2502 | 323.55 | exon (NM_001044392, exon 3 of 5) | MUC1 | protein-coding | |
| MACS_peak_14393 | 323.55 | Intergenic | DLGAP1 | protein-coding | |
| MACS_peak_15629 | 323.5 | intron (NM_000095, intron 4 of 18) | COMP | protein-coding | |
| MACS_peak_16250 | 323.39 | intron (NM_198977, intron 2 of 27) | ARHGEF1 | protein-coding | |
| MACS_peak_29045 | 323.39 | promoter-TSS (NM_001282876) | TMEM139 | protein-coding | |
| MACS_peak_22385 | 323.35 | intron (NM_015124, intron 2 of 18) | GRAMD4 | protein-coding | |
| MACS_peak_20976 | 323.21 | Intergenic | POTEH | protein-coding | |
| MACS_peak_2667 | 323.15 | non-coding (NR_028106, exon 1 of 4) | DCAF8 | protein-coding | |
| MACS_peak_17225 | 323.02 | Intergenic | ODC1-DT | ncRNA |  |
| MACS_peak_18970 | 323.02 | Intergenic | LINC01713 | ncRNA |  |
| MACS_peak_31284 | 323 | Intergenic | NTMT1 | protein-coding | |
| MACS_peak_9069 | 322.95 | intron (NM_001144995, intron 1 of 5) | CCDC85C | protein-coding | |
| MACS_peak_12877 | 322.82 | Intergenic | MRM1 | protein-coding | |
| MACS_peak_23811 | 322.81 | intron (NM_005255, intron 1 of 27) | GAK | protein-coding | |
| MACS_peak_1585 | 322.67 | intron (NR_038261, intron 2 of 3) | HIVEP3 | protein-coding | |
| MACS_peak_20021 | 322.56 | intron (NM_003185, intron 14 of 14) | MIR1257 | ncRNA |  |
| MACS_peak_22555 | 322.31 | Intergenic | LOC102724728 | pseudo |  |
| MACS_peak_16105 | 321.81 | exon (NM_001014831, exon 10 of 11) | NCCRP1 | protein-coding | |
| MACS_peak_17456 | 321.58 | promoter-TSS (NM_001201459) | ZNF513 | protein-coding | |
| MACS_peak_29139 | 321.42 | promoter-TSS (NM_001199692) | CDK5 | protein-coding | |
| MACS_peak_8476 | 321.4 | Intergenic | DUXAP10 | pseudo |  |
| MACS_peak_26818 | 321.36 | Intergenic | LOC442497 | ncRNA |  |
| MACS_peak_22324 | 321.33 | intron (NM_006486, intron 14 of 16) | LINC01589 | ncRNA |  |
| MACS_peak_17076 | 321.28 | promoter-TSS (NR_136527) | LOC105372483 | ncRNA |  |
| MACS_peak_27282 | 321.27 | Intergenic | LOC100129484 | protein-coding | |
| MACS_peak_208 | 321.24 | exon (NM_001313896, exon 18 of 20) | MMP23A | pseudo |  |
| MACS_peak_28395 | 321.1 | intron (NR_146079, intron 3 of 9).2 | SPDYE8P | pseudo |  |
| MACS_peak_12665 | 321.06 | Intergenic | MIR4522 | ncRNA |  |
| MACS_peak_14758 | 321.04 | intron (NM_001280, intron 3 of 6) | CIRBP-AS1 | ncRNA |  |
| MACS_peak_4019 | 321.02 | Intergenic | TMEM273 | protein-coding | |
| MACS_peak_21510 | 320.86 | promoter-TSS (NM_007128) | VPREB1 | protein-coding | |
| MACS_peak_18415 | 320.85 | Intergenic | GPD2 | protein-coding | |
| MACS_peak_4555 | 320.71 | intron (NM_030927, intron 4 of 8) | LOC101929574 | ncRNA |  |
| MACS_peak_21180 | 320.7 | Intergenic | LINC00895 | ncRNA |  |
| MACS_peak_25347 | 320.58 | Intergenic | IRF1 | protein-coding | |
| MACS_peak_502 | 320.53 | intron (NM_018836, intron 1 of 5) | AJAP1 | protein-coding | |
| MACS_peak_22743 | 320.52 | promoter-TSS (NM_024923) | NUP210 | protein-coding | |
| MACS_peak_19816 | 320.51 | Intergenic | LINC01742 | ncRNA |  |
| MACS_peak_8572 | 320.5 | exon (NM_001363682, exon 2 of 10) | NFATC4 | protein-coding | |
| MACS_peak_11884 | 320.5 | intron (NR_161452, intron 2 of 4) | MIR1910 | ncRNA |  |
| MACS_peak_2312 | 320.32 | exon (NM_001288607, exon 6 of 20) | MIR4257 | ncRNA |  |
| MACS_peak_11966 | 320.29 | intron (NM_020655, intron 2 of 4) | JPH3 | protein-coding | |
| MACS_peak_12712 | 320.23 | intron (NM_004295, intron 1 of 6) | TRAF4 | protein-coding | |
| MACS_peak_25920 | 320.2 | Intergenic | LOC105374960 | ncRNA |  |
| MACS_peak_3925 | 320.18 | Intergenic | FAM25BP | pseudo |  |
| MACS_peak_16407 | 320.16 | intron (NM_001369412, intron 1 of 9) | MIR6088 | ncRNA |  |
| MACS_peak_11051 | 320.1 | Intergenic | LINC02130 | ncRNA |  |
| MACS_peak_1163 | 320 | Intergenic | MIR4418 | ncRNA |  |
| MACS_peak_8447 | 319.96 | intron (NM_001320821, intron 5 of 25).2 | RASA3 | protein-coding | |
| MACS_peak_29463 | 319.95 | Intergenic | PRSS55 | protein-coding | |
| MACS_peak_20081 | 319.95 | Intergenic | LAMA5 | protein-coding | |
| MACS_peak_11664 | 319.94 | intron (NM_001270974, intron 63 of 85) | VAC14 | protein-coding | |
| MACS_peak_7996 | 319.86 | Intergenic | PRR20B | protein-coding | |
| MACS_peak_30344 | 319.73 | intron (NM_001363122, intron 2 of 4) | SLC52A2 | protein-coding | |
| MACS_peak_16781 | 319.73 | intron (NM_001080457, intron 1 of 2) | LRRC4B | protein-coding | |
| MACS_peak_15457 | 319.58 | intron (NM_006387, intron 2 of 16) | CHERP | protein-coding | |
| MACS_peak_8762 | 319.38 | promoter-TSS (NR_135816) | LOC100996664 | ncRNA |  |
| MACS_peak_207 | 319.35 | non-coding (NR_002946, exon 1 of 6).2 | MMP23A | pseudo |  |
| MACS_peak_24096 | 319.33 | intron (NM_004334, intron 1 of 8) | BST1 | protein-coding | |
| MACS_peak_597 | 319.31 | intron (NM_031475, intron 10 of 12) | TNFRSF25 | protein-coding | |
| MACS_peak_7097 | 319.29 | intron (NM_015481, intron 2 of 6) | ZNF385A | protein-coding | |
| MACS_peak_15621 | 319.27 | intron (NM_001345982, intron 2 of 3) | KLHL26 | protein-coding | |
| MACS_peak_27135 | 319.27 | intron (NM_025250, intron 5 of 13) | TTYH3 | protein-coding | |
| MACS_peak_21287 | 319.08 | intron (NM_023004, intron 1 of 1) | RTN4R | protein-coding | |
| MACS_peak_307 | 319.02 | intron (NM_014638, intron 5 of 21).2 | PLCH2 | protein-coding | |
| MACS_peak_31857 | 318.87 | promoter-TSS (NR_046340) | STPG3 | protein-coding | |
| MACS_peak_2676 | 318.81 | 3' UTR (NM_001184879, exon 8 of 8) | SLAMF6 | protein-coding | |
| MACS_peak_19602 | 318.79 | intron (NM_001290268, intron 6 of 21) | MIR1302-5 | ncRNA |  |
| MACS_peak_23613 | 318.71 | Intergenic | MASP1 | protein-coding | |
| MACS_peak_26563 | 318.71 | Intergenic | ARG1 | protein-coding | |
| MACS_peak_24600 | 318.67 | intron (NM_001044, intron 4 of 14).2 | SLC6A3 | protein-coding | |
| MACS_peak_9315 | 318.61 | Intergenic | TMEM121 | protein-coding | |
| MACS_peak_19564 | 318.6 | promoter-TSS (NM_001257398) | UBE2V1 | protein-coding | |
| MACS_peak_28282 | 318.48 | Intergenic | ELN | protein-coding | |
| MACS_peak_7587 | 318.47 | Intergenic | NCOR2 | protein-coding | |
| MACS_peak_5629 | 318.36 | intron (NM_001166212, intron 1 of 2) | CLCF1 | protein-coding | |
| MACS_peak_1218 | 318.34 | TTS (NM_021258) | MYOM3 | protein-coding | |
| MACS_peak_26776 | 318.23 | Intergenic | PSMB1 | protein-coding | |
| MACS_peak_7186 | 318.23 | promoter-TSS (NM_178502) | DTX3 | protein-coding | |
| MACS_peak_14232 | 318.05 | intron (NM_001330523, intron 3 of 6) | PYCR1 | protein-coding | |
| MACS_peak_20164 | 317.95 | non-coding (NR_102430, exon 1 of 3) | OGFR-AS1 | ncRNA |  |
| MACS_peak_22634 | 317.94 | Intergenic | CIDEC | protein-coding | |
| MACS_peak_2982 | 317.89 | intron (NM_177402, intron 1 of 8) | SYT2 | protein-coding | |
| MACS_peak_15442 | 317.85 | Intergenic | KLF2 | protein-coding | |
| MACS_peak_14235 | 317.78 | exon (NM_178493, exon 6 of 11) | NOTUM | protein-coding | |
| MACS_peak_3707 | 317.77 | intron (NM_031453, intron 1 of 4) | FAM107B | protein-coding | |
| MACS_peak_2420 | 317.69 | exon (NM_001367466, exon 21 of 28) | GATAD2B | protein-coding | |
| MACS_peak_30227 | 317.66 | Intergenic | MAFA-AS1 | ncRNA |  |
| MACS_peak_31031 | 317.65 | TTS (NR_104603) | CRB2 | protein-coding | |
| MACS_peak_24919 | 317.6 | Intergenic | SNORD170 | snoRNA |  |
| MACS_peak_10691 | 317.24 | Intergenic | HS3ST6 | protein-coding | |
| MACS_peak_2482 | 317.19 | intron (NR_049765, intron 2 of 3) | ZBTB7B | protein-coding | |
| MACS_peak_32103 | 317.05 | intron (NM_001098411, intron 1 of 4) | GAGE2B | protein-coding | |
| MACS_peak_1464 | 317.05 | Intergenic | CSF3R | protein-coding | |
| MACS_peak_23413 | 317.04 | Intergenic | CLDN18 | protein-coding | |
| MACS_peak_13896 | 316.94 | intron (NM_006640, intron 3 of 10) | SEPTIN9 | protein-coding | |
| MACS_peak_7960 | 316.81 | promoter-TSS (NR_002605) | DLEU2 | ncRNA |  |
| MACS_peak_13767 | 316.78 | promoter-TSS (NM_030630) | HID1 | protein-coding | |
| MACS_peak_5121 | 316.78 | intron (NM_001304359, intron 15 of 48) | MUC5AC | protein-coding | |
| MACS_peak_6089 | 316.72 | Intergenic | WNT11 | protein-coding | |
| MACS_peak_23880 | 316.68 | promoter-TSS (NM_024511) | HAUS3 | protein-coding | |
| MACS_peak_24513 | 316.59 | intron (NM_024786, intron 4 of 12) | ZDHHC11 | protein-coding | |
| MACS_peak_28341 | 316.53 | intron (NM_000265, intron 8 of 10) | NCF1 | protein-coding | |
| MACS_peak_2937 | 316.5 | exon (NM_001367841, exon 17 of 25) | IGFN1 | protein-coding | |
| MACS_peak_15495 | 316.43 | exon (NM_001278444, exon 3 of 8) | ANKLE1 | protein-coding | |
| MACS_peak_27089 | 316.34 | Intergenic | SNX8 | protein-coding | |
| MACS_peak_16352 | 316.26 | TTS (NR_107052) | MIR8085 | ncRNA |  |
| MACS_peak_16903 | 316.18 | intron (NM_031897, intron 1 of 1) | CACNG6 | protein-coding | |
| MACS_peak_7508 | 316.16 | exon (NM_001300801, exon 13 of 25) | MIR4304 | ncRNA |  |
| MACS_peak_19055 | 316.05 | Intergenic | MLLT10P1 | pseudo |  |
| MACS_peak_1550 | 315.99 | Intergenic | KCNQ4 | protein-coding | |
| MACS_peak_29021 | 315.97 | Intergenic | DENND2A | protein-coding | |
| MACS_peak_19979 | 315.71 | intron (NM_001252339, intron 1 of 14) | LOC100128310 | ncRNA |  |
| MACS_peak_2394 | 315.49 | promoter-TSS (NR_133645) | S100A16 | protein-coding | |
| MACS_peak_26284 | 315.43 | Intergenic | LINC01276 | ncRNA |  |
| MACS_peak_21130 | 315.33 | intron (NR_045121, intron 1 of 2) | DGCR5 | ncRNA |  |
| MACS_peak_27223 | 315.28 | intron (NR_157345, intron 3 of 16) | MIR4656 | ncRNA |  |
| MACS_peak_29008 | 315.22 | intron (NM_001366537, intron 4 of 11) | PARP12 | protein-coding | |
| MACS_peak_4410 | 315.18 | intron (NM_001322838, intron 1 of 29) | KCNMA1 | protein-coding | |
| MACS_peak_10187 | 315.09 | Intergenic | LOC440300 | pseudo |  |
| MACS_peak_2196 | 315.08 | Intergenic | LSP1P5 | pseudo |  |
| MACS_peak_13843 | 315.05 | intron (NM_022066, intron 1 of 17) | SPHK1 | protein-coding | |
| MACS_peak_19199 | 314.87 | intron (NR_119376, intron 23 of 42) | FER1L4 | pseudo |  |
| MACS_peak_5797 | 314.87 | Intergenic | LINC01488 | ncRNA |  |
| MACS_peak_14807 | 314.69 | Intergenic | KLF16 | protein-coding | |
| MACS_peak_16158 | 314.69 | intron (NM_012268, intron 2 of 12) | MIR6796 | ncRNA |  |
| MACS_peak_26141 | 314.68 | intron (NM_001256809, intron 1 of 8) | GRM4 | protein-coding | |
| MACS_peak_20216 | 314.68 | Intergenic | BIRC7 | protein-coding | |
| MACS_peak_21453 | 314.67 | Intergenic | PI4KAP2 | pseudo |  |
| MACS_peak_27352 | 314.58 | promoter-TSS (NM_001278559) | ZNF316 | protein-coding | |
| MACS_peak_9792 | 314.49 | intron (NR_027654, intron 4 of 4) | SMAD6 | protein-coding | |
| MACS_peak_18402 | 314.36 | 5' UTR (NM_001177663, exon 1 of 35) | RIF1 | protein-coding | |
| MACS_peak_20126 | 314.36 | promoter-TSS (NR_029676) | MIR133A2 | ncRNA |  |
| MACS_peak_24092 | 314.32 | Intergenic | WDR1 | protein-coding | |
| MACS_peak_13213 | 314.28 | intron (NM_001318933, intron 7 of 26) | ADAM11 | protein-coding | |
| MACS_peak_31776 | 314.22 | intron (NM_152421, intron 2 of 4) | SNORA17B | snoRNA |  |
| MACS_peak_25436 | 314.03 | Intergenic | PCDH1 | protein-coding | |
| MACS_peak_29549 | 314.03 | intron (NM_152272, intron 9 of 10) | CHMP7 | protein-coding | |
| MACS_peak_17061 | 314 | promoter-TSS (NM_152474) | C19orf18 | protein-coding | |
| MACS_peak_6515 | 313.94 | Intergenic | IQSEC3 | protein-coding | |
| MACS_peak_3211 | 313.93 | Intergenic | ZC3H11B | protein-coding | |
| MACS_peak_21666 | 313.87 | Intergenic | POM121L9P | pseudo |  |
| MACS_peak_10203 | 313.86 | Intergenic | LOC103171574 | ncRNA |  |
| MACS_peak_259 | 313.83 | Intergenic | FAAP20 | protein-coding | |
| MACS_peak_15650 | 313.69 | intron (NM_178526, intron 1 of 7) | SLC25A42 | protein-coding | |
| MACS_peak_30208 | 313.68 | 3' UTR (NM_173832, exon 3 of 3) | GLI4 | protein-coding | |
| MACS_peak_14953 | 313.53 | intron (NM_015897, intron 1 of 10) | PIAS4 | protein-coding | |
| MACS_peak_3441 | 313.36 | promoter-TSS (NM_001098722) | GNG4 | protein-coding | |
| MACS_peak_7760 | 313.3 | Intergenic | LINC02361 | ncRNA |  |
| MACS_peak_17882 | 313.26 | intron (NM_001330402, intron 12 of 12) | EMX1 | protein-coding | |
| MACS_peak_20255 | 313.18 | intron (NM_172107, intron 11 of 16) | KCNQ2 | protein-coding | |
| MACS_peak_15330 | 313.02 | intron (NM_004843, intron 10 of 13) | PALM3 | protein-coding | |
| MACS_peak_77 | 312.94 | promoter-TSS (NM_001364727) | AGRN | protein-coding | |
| MACS_peak_32227 | 312.92 | intron (NR_147172, intron 2 of 2) | DANT1 | ncRNA |  |
| MACS_peak_31137 | 312.92 | Intergenic | NIBAN2 | protein-coding | |
| MACS_peak_14158 | 312.86 | intron (NR_038080, intron 1 of 3) | LINC00482 | ncRNA |  |
| MACS_peak_13156 | 312.78 | promoter-TSS (NR_002716).10 | RNU2-1 | snRNA |  |
| MACS_peak_15468 | 312.72 | exon (NM_015260, exon 2 of 20) | SIN3B | protein-coding | |
| MACS_peak_12616 | 312.61 | intron (NM_152914, intron 1 of 2) | NATD1 | protein-coding | |
| MACS_peak_2498 | 312.61 | promoter-TSS (NM_001122837) | SLC50A1 | protein-coding | |
| MACS_peak_9545 | 312.47 | Intergenic | LOC101928042 | ncRNA |  |
| MACS_peak_15053 | 312.35 | intron (NR_034166, intron 1 of 4) | NDUFA11 | protein-coding | |
| MACS_peak_16296 | 312.34 | promoter-TSS (NM_001301035) | ERF | protein-coding | |
| MACS_peak_24132 | 312.33 | intron (NM_001270880, intron 1 of 5) | GNPDA2 | protein-coding | |
| MACS_peak_23847 | 312.14 | Intergenic | FAM53A | protein-coding | |
| MACS_peak_605 | 312.13 | intron (NM_001042663, intron 2 of 21) | PLEKHG5 | protein-coding | |
| MACS_peak_22326 | 311.98 | intron (NM_006486, intron 16 of 16) | LINC01589 | ncRNA |  |
| MACS_peak_26228 | 311.86 | Intergenic | MIR4462 | ncRNA |  |
| MACS_peak_3991 | 311.82 | intron (NM_001278794, intron 1 of 1).2 | NPY4R | protein-coding | |
| MACS_peak_19805 | 311.65 | Intergenic | LINC01742 | ncRNA |  |
| MACS_peak_11880 | 311.64 | exon (NM_014615, exon 4 of 16) | GINS2 | protein-coding | |
| MACS_peak_20256 | 311.6 | intron (NM_172107, intron 10 of 16) | KCNQ2 | protein-coding | |
| MACS_peak_18867 | 311.46 | intron (NM_014808, intron 14 of 26) | MIR3133 | ncRNA |  |
| MACS_peak_13204 | 311.23 | Intergenic | FZD2 | protein-coding | |
| MACS_peak_28273 | 311.19 | Intergenic | CLDN4 | protein-coding | |
| MACS_peak_17557 | 311.17 | promoter-TSS (NM_001322373) | CEBPZOS | protein-coding | |
| MACS_peak_13393 | 311.17 | intron (NM_032595, intron 7 of 9) | SAMD14 | protein-coding | |
| MACS_peak_21676 | 311.15 | promoter-TSS (NR_103543) | ADORA2A | protein-coding | |
| MACS_peak_25583 | 311.14 | Intergenic | LINC01411 | ncRNA |  |
| MACS_peak_31146 | 311.12 | intron (NM_001252334, intron 3 of 11) | SH2D3C | protein-coding | |
| MACS_peak_31162 | 311.08 | promoter-TSS (NM_000476) | AK1 | protein-coding | |
| MACS_peak_14536 | 310.91 | promoter-TSS (NM_001308263) | FAM210A | protein-coding | |
| MACS_peak_31971 | 310.87 | Intergenic | LOC100507412 | ncRNA |  |
| MACS_peak_7131 | 310.82 | promoter-TSS (NM_079423) | MYL6 | protein-coding | |
| MACS_peak_8489 | 310.8 | Intergenic | DUXAP10 | pseudo |  |
| MACS_peak_2983 | 310.61 | non-coding (NR_046326, exon 1 of 2) | PCAT6 | ncRNA |  |
| MACS_peak_14916 | 310.57 | intron (NM_001136503, intron 1 of 3) | SMIM24 | protein-coding | |
| MACS_peak_21291 | 310.53 | Intergenic | RTN4R | protein-coding | |
| MACS_peak_7753 | 310.33 | intron (NM_015409, intron 45 of 52) | SNORA49 | snoRNA |  |
| MACS_peak_19890 | 310.29 | Intergenic | EDN3 | protein-coding | |
| MACS_peak_11416 | 310.12 | Intergenic | ENPP7P13 | pseudo |  |
| MACS_peak_32230 | 310.1 | intron (NR_147172, intron 2 of 2) | DANT1 | ncRNA |  |
| MACS_peak_22026 | 310.04 | intron (NR_029413, intron 3 of 6) | MFNG | protein-coding | |
| MACS_peak_20333 | 310.02 | promoter-TSS (NR_031735) | MIR647 | ncRNA |  |
| MACS_peak_194 | 309.89 | intron (NM_080875, intron 8 of 19) | MMP23B | protein-coding | |
| MACS_peak_24790 | 309.77 | promoter-TSS (NR_136739) | SRD5A1 | protein-coding | |
| MACS_peak_549 | 309.73 | intron (NM_001199861, intron 1 of 15) | KCNAB2 | protein-coding | |
| MACS_peak_25088 | 309.42 | Intergenic | SLC1A3 | protein-coding | |
| MACS_peak_30257 | 309.38 | 3' UTR (NM_032862, exon 1 of 1).2 | TIGD5 | protein-coding | |
| MACS_peak_21770 | 309.36 | Intergenic | LINC02554 | ncRNA |  |
| MACS_peak_8274 | 309.32 | Intergenic | ANKRD10 | protein-coding | |
| MACS_peak_28755 | 309.3 | Intergenic | RASA4B | protein-coding | |
| MACS_peak_5044 | 309.19 | intron (NM_152643, intron 5 of 29) | KNDC1 | protein-coding | |
| MACS_peak_20975 | 309.15 | Intergenic | OR11H1 | protein-coding | |
| MACS_peak_14657 | 309.1 | intron (NM_004359, intron 1 of 4) | CDC34 | protein-coding | |
| MACS_peak_21563 | 309.04 | intron (NM_004327, intron 4 of 22) | FBXW4P1 | pseudo |  |
| MACS_peak_7 | 309.04 | Intergenic | LOC729737 | ncRNA |  |
| MACS_peak_12587 | 308.99 | Intergenic | ALDH3A2 | protein-coding | |
| MACS_peak_17847 | 308.94 | promoter-TSS (NM_012476) | VAX2 | protein-coding | |
| MACS_peak_29676 | 308.83 | Intergenic | LINC01606 | ncRNA |  |
| MACS_peak_21268 | 308.72 | Intergenic | LOC284865 | ncRNA |  |
| MACS_peak_15329 | 308.59 | TTS (NM_001311197) | IL27RA | protein-coding | |
| MACS_peak_2830 | 308.51 | intron (NM_005819, intron 6 of 7) | KIAA1614-AS1 | ncRNA |  |
| MACS_peak_20345 | 308.46 | Intergenic | SOX18 | protein-coding | |
| MACS_peak_30333 | 308.4 | intron (NM_015201, intron 3 of 15) | SCX | protein-coding | |
| MACS_peak_26937 | 308.33 | Intergenic | UNCX | protein-coding | |
| MACS_peak_20492 | 308.31 | TTS (NR_038327) | TEKT4P2 | pseudo |  |
| MACS_peak_26564 | 308.09 | Intergenic | CCN2 | protein-coding | |
| MACS_peak_22134 | 307.96 | promoter-TSS (NM_145738) | SYNGR1 | protein-coding | |
| MACS_peak_6480 | 307.95 | intron (NM_001143820, intron 3 of 9) | ETS1-AS1 | ncRNA |  |
| MACS_peak_18947 | 307.93 | promoter-TSS (NM_000490) | AVP | protein-coding | |
| MACS_peak_5147 | 307.88 | Intergenic | PHLDA2 | protein-coding | |
| MACS_peak_7572 | 307.84 | intron (NM_001077261, intron 5 of 47) | NCOR2 | protein-coding | |
| MACS_peak_5328 | 307.53 | promoter-TSS (NM_001357016) | NR1H3 | protein-coding | |
| MACS_peak_28323 | 307.53 | intron (NM_016328, intron 1 of 26) | GTF2IRD1 | protein-coding | |
| MACS_peak_27355 | 307.35 | intron (NR_002217, intron 5 of 5) | RSPH10B | protein-coding | |
| MACS_peak_32420 | 307.33 | TTS (NM_001330067).2 | OPN1MW3 | protein-coding | |
| MACS_peak_31362 | 307.27 | Intergenic | FIBCD1 | protein-coding | |
| MACS_peak_17664 | 307.16 | intron (NR_147194, intron 1 of 2) | LINC01833 | ncRNA |  |
| MACS_peak_27775 | 307.03 | 5' UTR (NM_001129, exon 1 of 21) | AEBP1 | protein-coding | |
| MACS_peak_10351 | 307 | Intergenic | SLCO3A1 | protein-coding | |
| MACS_peak_28007 | 306.92 | Intergenic | VOPP1 | protein-coding | |
| MACS_peak_19747 | 306.91 | intron (NM_001719, intron 2 of 6) | BMP7-AS1 | ncRNA |  |
| MACS_peak_12028 | 306.79 | intron (NM_153813, intron 3 of 9) | MIR5189 | ncRNA |  |
| MACS_peak_4614 | 306.78 | TTS (NR_024467) | PCGF5 | protein-coding | |
| MACS_peak_5028 | 306.75 | Intergenic | LINC01168 | ncRNA |  |
| MACS_peak_24477 | 306.63 | Intergenic | MIR4456 | ncRNA |  |
| MACS_peak_27218 | 306.56 | intron (NM_001037165, intron 8 of 8) | AP5Z1 | protein-coding | |
| MACS_peak_2946 | 306.55 | TTS (NM_012396) | PHLDA3 | protein-coding | |
| MACS_peak_30055 | 306.51 | 3' UTR (NM_007079, exon 4 of 4).2 | PTP4A3 | protein-coding | |
| MACS_peak_30834 | 306.46 | intron (NM_005502, intron 1 of 49) | ABCA1 | protein-coding | |
| MACS_peak_11282 | 306.44 | intron (NM_001024401, intron 1 of 3) | SBK1 | protein-coding | |
| MACS_peak_3231 | 306.39 | Intergenic | TLR5 | protein-coding | |
| MACS_peak_26540 | 306.31 | Intergenic | HS3ST5 | protein-coding | |
| MACS_peak_630 | 306.23 | intron (NM_001349608, intron 2 of 21) | CAMTA1 | protein-coding | |
| MACS_peak_16801 | 305.98 | promoter-TSS (NR_003068) | SNORD88A | snoRNA |  |
| MACS_peak_5718 | 305.97 | Intergenic | GAL | protein-coding | |
| MACS_peak_6712 | 305.94 | intron (NM_080548, intron 1 of 15) | PTPN6 | protein-coding | |
| MACS_peak_28099 | 305.91 | Intergenic | MIR4283-1 | ncRNA |  |
| MACS_peak_32100 | 305.9 | promoter-TSS (NM_021123).2 | GAGE12F | protein-coding | |
| MACS_peak_6084 | 305.87 | Intergenic | WNT11 | protein-coding | |
| MACS_peak_856 | 305.87 | 5' UTR (NM_014874, exon 1 of 19) | MFN2 | protein-coding | |
| MACS_peak_31854 | 305.85 | exon (NM_080877, exon 8 of 13) | SLC34A3 | protein-coding | |
| MACS_peak_20316 | 305.85 | intron (NM_001369741, intron 4 of 4) | SLC2A4RG | protein-coding | |
| MACS_peak_5392 | 305.82 | intron (NM_014224, intron 4 of 8) | PGA5 | protein-coding | |
| MACS_peak_11155 | 305.71 | TTS (NR_106766).4 | LOC100288162 | ncRNA |  |
| MACS_peak_1899 | 305.66 | Intergenic | LEPR | protein-coding | |
| MACS_peak_24499 | 305.65 | intron (NR_147095, intron 15 of 16) | TPPP | protein-coding | |
| MACS_peak_12372 | 305.62 | Intergenic | WSCD1 | protein-coding | |
| MACS_peak_30341 | 305.48 | Intergenic | SCRT1 | protein-coding | |
| MACS_peak_1668 | 305.42 | promoter-TSS (NM_001166292) | PTCH2 | protein-coding | |
| MACS_peak_15856 | 305.41 | intron (NM_001127895, intron 3 of 4) | CHST8 | protein-coding | |
| MACS_peak_16749 | 305.4 | promoter-TSS (NR_024214).10 | SNAR-A14 | snRNA |  |
| MACS_peak_10154 | 305.35 | Intergenic | LOC102724034 | ncRNA |  |
| MACS_peak_12020 | 305.23 | promoter-TSS (NR_049821) | MIR5189 | ncRNA |  |
| MACS_peak_17795 | 305.21 | Intergenic | MIR4433B | ncRNA |  |
| MACS_peak_31183 | 305.12 | Intergenic | LCN2 | protein-coding | |
| MACS_peak_20219 | 304.99 | intron (NM_001363747, intron 4 of 6) | MIR3196 | ncRNA |  |
| MACS_peak_15015 | 304.95 | intron (NM_001370094, intron 6 of 10) | KDM4B | protein-coding | |
| MACS_peak_27619 | 304.94 | intron (NM_001322059, intron 1 of 17) | PDE1C | protein-coding | |
| MACS_peak_10406 | 304.91 | intron (NM_145728, intron 2 of 3) | SYNM | protein-coding | |
| MACS_peak_9572 | 304.89 | intron (NM_001282472, intron 1 of 18).3 | GOLGA8J | protein-coding | |
| MACS_peak_30543 | 304.85 | exon (NM_001145196, exon 4 of 4) | SPATA31A6 | protein-coding | |
| MACS_peak_14774 | 304.75 | Intergenic | APC2 | protein-coding | |
| MACS_peak_13581 | 304.7 | 3' UTR (NM_001039933, exon 6 of 6) | CD79B | protein-coding | |
| MACS_peak_10598 | 304.67 | Intergenic | SSTR5 | protein-coding | |
| MACS_peak_15544 | 304.67 | intron (NM_001161358, intron 27 of 27) | B3GNT3 | protein-coding | |
| MACS_peak_7580 | 304.66 | intron (NM_001077261, intron 1 of 47) | NCOR2 | protein-coding | |
| MACS_peak_9345 | 304.61 | Intergenic | ELK2AP | pseudo |  |
| MACS_peak_8002 | 304.59 | exon (NM_001130407, exon 2 of 3).3 | PRR20C | protein-coding | |
| MACS_peak_27030 | 304.41 | intron (NR_120508, intron 1 of 1) | ELFN1-AS1 | ncRNA |  |
| MACS_peak_28441 | 304.37 | exon (NM_001291831, exon 13 of 16) | HSPB1 | protein-coding | |
| MACS_peak_1966 | 304.28 | Intergenic | CDC7 | protein-coding | |
| MACS_peak_7271 | 304.25 | Intergenic | LOC101929058 | ncRNA |  |
| MACS_peak_31356 | 304.24 | intron (NM_001145106, intron 5 of 7) | QRFP | protein-coding | |
| MACS_peak_20374 | 304.17 | Intergenic | PCMTD2 | protein-coding | |
| MACS_peak_30260 | 304.1 | promoter-TSS (NM_001317783).2 | TSTA3 | protein-coding | |
| MACS_peak_21212 | 303.9 | intron (NM_053004, intron 2 of 7) | RTL10 | protein-coding | |
| MACS_peak_21744 | 303.88 | Intergenic | LRP5L | protein-coding | |
| MACS_peak_29065 | 303.87 | exon (NM_001206941, exon 2 of 8) | TCAF1 | protein-coding | |
| MACS_peak_29295 | 303.8 | intron (NM_130843, intron 10 of 21) | LOC100506585 | ncRNA |  |
| MACS_peak_10544 | 303.7 | intron (NM_153350, intron 1 of 5) | FBXL16 | protein-coding | |
| MACS_peak_19782 | 303.69 | intron (NR_136660, intron 6 of 7) | ZBP1 | protein-coding | |
| MACS_peak_12719 | 303.66 | promoter-TSS (NM_001330170) | FLOT2 | protein-coding | |
| MACS_peak_27907 | 303.55 | intron (NM_022748, intron 4 of 30) | SNORD151 | snoRNA |  |
| MACS_peak_31829 | 303.54 | 3' UTR (NM_207511, exon 3 of 3) | FUT7 | protein-coding | |
| MACS_peak_30152 | 303.54 | Intergenic | ARC | protein-coding | |
| MACS_peak_25089 | 303.48 | Intergenic | SLC1A3 | protein-coding | |
| MACS_peak_18350 | 303.43 | Intergenic | PRSS40B | pseudo |  |
| MACS_peak_18893 | 303.34 | intron (NR_110220, intron 2 of 3) | LINC01237 | ncRNA |  |
| MACS_peak_16593 | 303.31 | intron (NM_017457, intron 1 of 11) | CYTH2 | protein-coding | |
| MACS_peak_11971 | 303.29 | Intergenic | LOC102724467 | ncRNA |  |
| MACS_peak_9391 | 303.11 | NA |  |  |  |
| MACS_peak_22025 | 303.04 | intron (NR_029413, intron 4 of 6) | MFNG | protein-coding | |
| MACS_peak_21422 | 303.03 | Intergenic | RIMBP3B | protein-coding | |
| MACS_peak_19804 | 302.94 | Intergenic | LINC01742 | ncRNA |  |
| MACS_peak_10242 | 302.8 | Intergenic | LOC101929479 | pseudo |  |
| MACS_peak_11722 | 302.7 | Intergenic | CDYL2 | protein-coding | |
| MACS_peak_17726 | 302.64 | promoter-TSS (NM_001281494) | MSH6 | protein-coding | |
| MACS_peak_4126 | 302.62 | intron (NM_015634, intron 2 of 6) | KIF1BP | protein-coding | |
| MACS_peak_109 | 302.5 | Intergenic | TTLL10 | protein-coding | |
| MACS_peak_2607 | 302.49 | intron (NM_002529, intron 1 of 16) | NTRK1 | protein-coding | |
| MACS_peak_4525 | 302.41 | promoter-TSS (NM_001355263) | NUTM2E | protein-coding | |
| MACS_peak_26554 | 302.37 | Intergenic | HDDC2 | protein-coding | |
| MACS_peak_15729 | 302.36 | intron (NR_110759, intron 5 of 9) | LOC100420587 | pseudo |  |
| MACS_peak_5591 | 302.34 | intron (NM_006946, intron 30 of 36) | RBM4B | protein-coding | |
| MACS_peak_778 | 302.27 | intron (NM_001079843, intron 2 of 20) | CASZ1 | protein-coding | |
| MACS_peak_5661 | 302.24 | intron (NM_080658, intron 1 of 7) | ACY3 | protein-coding | |
| MACS_peak_29276 | 302.17 | intron (NM_130843, intron 21 of 21) | MIR153-2 | ncRNA |  |
| MACS_peak_24708 | 302.14 | Intergenic | LINC01377 | ncRNA |  |
| MACS_peak_12085 | 302.05 | intron (NM_005187, intron 1 of 11) | LOC100129697 | protein-coding | |
| MACS_peak_4862 | 302.03 | Intergenic | FAM53B-AS1 | ncRNA |  |
| MACS_peak_27961 | 301.96 | intron (NM_001350816, intron 5 of 17) | GRB10 | protein-coding | |
| MACS_peak_31494 | 301.92 | exon (NM_000787, exon 10 of 12) | DBH-AS1 | ncRNA |  |
| MACS_peak_9920 | 301.86 | Intergenic | INSYN1-AS1 | ncRNA |  |
| MACS_peak_9886 | 301.83 | promoter-TSS (NM_001206799) | PKM | protein-coding | |
| MACS_peak_5002 | 301.75 | intron (NM_005539, intron 8 of 15) | INPP5A | protein-coding | |
| MACS_peak_29314 | 301.69 | intron (NM_130842, intron 1 of 21) | MIR595 | ncRNA |  |
| MACS_peak_17313 | 301.68 | Intergenic | HS1BP3-IT1 | ncRNA |  |
| MACS_peak_28869 | 301.59 | Intergenic | SND1 | protein-coding | |
| MACS_peak_9833 | 301.59 | intron (NM_001324091, intron 1 of 4) | GLCE | protein-coding | |
| MACS_peak_17966 | 301.48 | intron (NM_198843, intron 5 of 11) | SFTPB | protein-coding | |
| MACS_peak_28957 | 301.43 | intron (NM_001105543, intron 3 of 3) | PLXNA4 | protein-coding | |
| MACS_peak_7874 | 301.41 | Intergenic | GTF3A | protein-coding | |
| MACS_peak_27026 | 301.25 | intron (NM_001128636, intron 2 of 2) | ELFN1-AS1 | ncRNA |  |
| MACS_peak_30689 | 301.22 | promoter-TSS (NM_001161625) | NXNL2 | protein-coding | |
| MACS_peak_3834 | 301.18 | intron (NM_020630, intron 1 of 18) | RET | protein-coding | |
| MACS_peak_25107 | 301.16 | promoter-TSS (NM_001205301) | EGFLAM | protein-coding | |
| MACS_peak_1070 | 301.05 | 5' UTR (NM_013945, exon 1 of 8) | PAX7 | protein-coding | |
| MACS_peak_17696 | 300.9 | intron (NM_144949, intron 1 of 1) | SOCS5 | protein-coding | |
| MACS_peak_1941 | 300.8 | Intergenic | BCL10 | protein-coding | |
| MACS_peak_13177 | 300.77 | TTS (NR_136406) | LINC01976 | ncRNA |  |
| MACS_peak_8439 | 300.68 | intron (NM_001320821, intron 21 of 25).2 | C13orf46 | protein-coding | |
| MACS_peak_6421 | 300.61 | Intergenic | TRIM29 | protein-coding | |
| MACS_peak_15605 | 300.6 | Intergenic | KXD1 | protein-coding | |
| MACS_peak_22101 | 300.6 | Intergenic | SUN2 | protein-coding | |
| MACS_peak_10634 | 300.5 | Intergenic | TPSD1 | protein-coding | |
| MACS_peak_3037 | 300.5 | intron (NM_001278517, intron 1 of 7) | MDM4 | protein-coding | |
| MACS_peak_12954 | 300.37 | intron (NR_073384, intron 5 of 7) | MIR6779 | ncRNA |  |
| MACS_peak_9437 | 300.35 | Intergenic | GOLGA8CP | pseudo |  |
| MACS_peak_10217 | 300.13 | intron (NM_001267536, intron 9 of 10) | DNM1P41 | pseudo |  |
| MACS_peak_24619 | 300.02 | Intergenic | LPCAT1 | protein-coding | |
| MACS_peak_29582 | 300.02 | intron (NR_026826, intron 11 of 14) | EXTL3 | protein-coding | |
| MACS_peak_239 | 300 | promoter-TSS (NM_001350803) | PRKCZ | protein-coding | |
| MACS_peak_17346 | 300 | Intergenic | ATAD2B | protein-coding | |

Table S6. Overlapped genes obtained from intersection

of RNA-seq and ChIRP-seq dataset

| **Gene Name** | **Fold Change** | **p-value** | **q-value** |
| --- | --- | --- | --- |
| CPN2 | 0.006853512 | 5.07661E-09 | 6.08654E-07 |
| ASTL | 0.009384567 | 2.28309E-07 | 1.65941E-05 |
| CTCFL | 0.01033079 | 5.4473E-07 | 3.54966E-05 |
| C1orf189 | 0.01897859 | 1.12969E-05 | 0.000519109 |
| GLT6D1 | 0.019461093 | 7.16854E-05 | 0.002429909 |
| CABP4 | 0.019761591 | 1.87636E-05 | 0.000785785 |
| KRT36 | 0.021100412 | 0.000158888 | 0.004655177 |
| TMEM150B | 0.022935958 | 4.22791E-05 | 0.001558964 |
| SLC6A4 | 0.024113175 | 0.000290176 | 0.007563597 |
| TNFRSF4 | 0.027374534 | 0.000142854 | 0.004276554 |
| ACTN3 | 0.028124827 | 0.000833844 | 0.017317713 |
| SLC6A3 | 0.029729948 | 2.07413E-14 | 6.27133E-12 |
| ACTL7B | 0.030837102 | 0.000250545 | 0.006668543 |
| TEX35 | 0.031634718 | 0.001456907 | 0.026270944 |
| MYBPC3 | 0.031674715 | 0.00141135 | 0.025583678 |
| ABCG5 | 0.031737079 | 0.001755858 | 0.029659289 |
| ADAM30 | 0.037974162 | 0.00089856 | 0.018234137 |
| LRRC43 | 0.038907105 | 0.004393787 | 0.056773742 |
| SPATA32 | 0.042219393 | 5.9609E-11 | 1.10636E-08 |
| NLRP12 | 0.042869685 | 0.001610806 | 0.027958855 |
| THBS2 | 0.046029169 | 0.011284274 | 0.10517269 |
| DLK1 | 0.046145759 | 0.011389134 | 0.105632475 |
| TAS1R2 | 0.046944781 | 0.00261393 | 0.039438524 |
| MMP12 | 0.050465472 | 0.01763247 | 0.14126533 |
| MCHR1 | 0.050538825 | 0.016608233 | 0.135941127 |
| NPHS1 | 0.050609346 | 0.01569364 | 0.131444017 |
| KLHL40 | 0.050764401 | 0.014936844 | 0.127445706 |
| GNLY | 0.050800487 | 0.016909806 | 0.137368323 |
| ZBP1 | 0.054070273 | 8.78167E-16 | 3.12963E-13 |
| LRTM2 | 0.05473893 | 0.005863283 | 0.069035139 |
| HRH3 | 0.056132915 | 0.023690592 | 0.170073034 |
| FAM186A | 0.056391513 | 0.024056958 | 0.171069184 |
| GRK1 | 0.0590473 | 1.50245E-11 | 2.98869E-09 |
| PLA2G2F | 0.061693125 | 0.008627992 | 0.088193368 |
| TEX101 | 0.063146583 | 0.035615346 | 0.215597222 |
| CIDEA | 0.063146583 | 0.035615346 | 0.215597222 |
| WFIKKN2 | 0.063164802 | 0.049151508 | 0.259025299 |
| DNAJB8 | 0.063174411 | 0.035664465 | 0.21567015 |
| UNCX | 0.063310903 | 0.037201174 | 0.220120295 |
| GYS2 | 0.063417013 | 0.036093567 | 0.217135909 |
| CABP7 | 0.063444107 | 0.034364945 | 0.211541472 |
| FAT3 | 0.063527493 | 0.037594113 | 0.221282436 |
| TSSK2 | 0.063527493 | 0.037594113 | 0.221282436 |
| HK3 | 0.065746016 | 0.011555218 | 0.106714589 |
| ZNF750 | 0.070485343 | 0.016684691 | 0.136271828 |
| DPEP1 | 0.070594849 | 0.000248835 | 0.006646585 |
| LOC102724265 | 0.076744456 | 0.004590588 | 0.058508839 |
| STPG3 | 0.07703023 | 0.004368278 | 0.056686372 |
| MASP1 | 0.082427524 | 0.030414325 | 0.198447892 |
| OIT3 | 0.083527825 | 3.13895E-07 | 2.2072E-05 |
| LRRC14B | 0.085710626 | 5.60128E-07 | 3.63434E-05 |
| GPR52 | 0.091162053 | 0.011433328 | 0.105912412 |
| RPS6KL1 | 0.091830124 | 0.001574193 | 0.027608644 |
| NPC1L1 | 0.100280396 | 6.04161E-14 | 1.75648E-11 |
| STAB1 | 0.100431388 | 5.95644E-08 | 5.34037E-06 |
| SSTR3 | 0.102835957 | 0.01719053 | 0.138501689 |
| FOXE3 | 0.103905557 | 3.16081E-06 | 0.000175037 |
| GSC2 | 0.115860677 | 0.000193868 | 0.005417565 |
| PDE6G | 0.116692081 | 1.21855E-07 | 9.59479E-06 |
| HIPK4 | 0.127994496 | 0.000283565 | 0.007442595 |
| SPIB | 0.132365896 | 4.91526E-10 | 7.74051E-08 |
| POU5F2 | 0.137683578 | 0.018006104 | 0.143801519 |
| OSM | 0.148024905 | 0.008115369 | 0.085140975 |
| STPG4 | 0.152720326 | 8.05739E-20 | 4.68506E-17 |
| LGALS4 | 0.161462193 | 3.09557E-13 | 7.93201E-11 |
| IQCA1L | 0.163299457 | 0.040814695 | 0.233460671 |
| TTLL10 | 0.16823245 | 1.20825E-10 | 2.07572E-08 |
| NR5A1 | 0.169723836 | 0.002107392 | 0.033785318 |
| LDLRAD4 | 0.173268083 | 0.001566388 | 0.027567697 |
| RSPH6A | 0.177966681 | 0.013981475 | 0.122321722 |
| MYLK2 | 0.179776616 | 8.74983E-17 | 3.77943E-14 |
| KIF5A | 0.185081488 | 0.000191273 | 0.005384953 |
| GFRA3 | 0.185953836 | 0.01855216 | 0.147074752 |
| WFIKKN1 | 0.189086144 | 0.00262414 | 0.039474379 |
| PLEKHD1 | 0.190662359 | 7.39609E-06 | 0.000365406 |
| CD7 | 0.190785686 | 0.008544521 | 0.087815134 |
| C20orf204 | 0.197337883 | 3.12697E-09 | 3.99122E-07 |
| KRT14 | 0.197678396 | 0.009960696 | 0.097719532 |
| AZU1 | 0.204001907 | 0.011578079 | 0.106860442 |
| SRL | 0.204255428 | 4.08568E-05 | 0.001521362 |
| ORM1 | 0.20449658 | 0.049595924 | 0.260434589 |
| MUC2 | 0.214731571 | 0.010111311 | 0.098725382 |
| MYO1G | 0.215777849 | 2.19003E-05 | 0.00089726 |
| FAM71E2 | 0.215846968 | 4.08217E-05 | 0.001521362 |
| NPIPB6 | 0.219513744 | 0.006659012 | 0.074309161 |
| SCG2 | 0.220478079 | 0.019467094 | 0.151002324 |
| ARC | 0.22237939 | 1.47183E-36 | 5.5628E-33 |
| MYH11 | 0.225785932 | 0.000441097 | 0.010652568 |
| GOLGA6B | 0.228448366 | 0.000811188 | 0.016985506 |
| ASIC4 | 0.23050538 | 0.004454487 | 0.057250931 |
| PECAM1 | 0.248890778 | 0.038119996 | 0.222734198 |
| ABO | 0.25736484 | 0.041645264 | 0.237114943 |
| WDR97 | 0.272331291 | 0.001870698 | 0.0310101 |
| TEX29 | 0.277875386 | 1.09972E-05 | 0.000506878 |
| C8G | 0.285031377 | 4.75687E-06 | 0.000249702 |
| CEP295NL | 0.285710052 | 9.6889E-06 | 0.000453489 |
| CHD5 | 0.285909069 | 1.43921E-12 | 3.1997E-10 |
| BAIAP2L2 | 0.290281198 | 2.07291E-05 | 0.000856236 |
| GOLGA6A | 0.291849487 | 0.012228414 | 0.111166065 |
| BHLHE41 | 0.294297653 | 4.38764E-09 | 5.34939E-07 |
| KCNN3 | 0.303488744 | 0.000504253 | 0.011892818 |
| PERM1 | 0.30619079 | 0.011065207 | 0.104199771 |
| ASGR1 | 0.308196883 | 1.59768E-05 | 0.000682044 |
| EMC10 | 0.309370061 | 0.030153555 | 0.197513625 |
| NTRK1 | 0.310425022 | 0.014374618 | 0.124539771 |
| C7orf61 | 0.311715893 | 0.012868276 | 0.115553466 |
| CKM | 0.313506342 | 0.032549359 | 0.206150487 |
| TMEM240 | 0.3177419 | 0.00775746 | 0.082184495 |
| GPT | 0.32127559 | 0.031754881 | 0.203287844 |
| RSPH10B | 0.335084812 | 0.003283978 | 0.046183426 |
| ZDHHC11B | 0.33975544 | 1.17342E-09 | 1.67356E-07 |
| SAP25 | 0.342443204 | 1.07727E-07 | 8.6368E-06 |
| NPIPA7 | 0.345723273 | 5.82052E-06 | 0.000294296 |
| SIK1 | 0.346871946 | 0.03133623 | 0.201849651 |
| SCRT1 | 0.347917771 | 0.042664424 | 0.240134311 |
| HMCN2 | 0.348082128 | 0.030317373 | 0.198243099 |
| IQCN | 0.354502539 | 3.14166E-09 | 3.99122E-07 |
| SPDEF | 0.35504501 | 0.019356595 | 0.150633805 |
| MUC20 | 0.359541119 | 4.98775E-06 | 0.000259123 |
| COL20A1 | 0.36241628 | 2.70297E-07 | 1.93666E-05 |
| CHRNA4 | 0.363408379 | 0.029304233 | 0.193374683 |
| LCN12 | 0.364496853 | 2.67978E-06 | 0.000151734 |
| TPPP | 0.36620103 | 3.96028E-10 | 6.30227E-08 |
| CLCN1 | 0.372533046 | 0.002394177 | 0.037276172 |
| RASD1 | 0.37520054 | 5.2051E-13 | 1.26921E-10 |
| LHX3 | 0.38456642 | 1.16012E-05 | 0.000526688 |
| TCAP | 0.390461977 | 0.019311337 | 0.150633805 |
| ALPK3 | 0.393646053 | 3.08433E-12 | 6.47623E-10 |
| SPOCK2 | 0.395532666 | 1.44441E-21 | 1.03984E-18 |
| LDB3 | 0.400189884 | 0.027790099 | 0.187140634 |
| DRICH1 | 0.403228953 | 0.003303385 | 0.046284126 |
| SNAI1 | 0.404608155 | 4.411E-27 | 6.66856E-24 |
| LEKR1 | 0.408599118 | 0.000149184 | 0.0044397 |
| SEMA4A | 0.414491393 | 1.0122E-10 | 1.77936E-08 |
| PTCH2 | 0.415178501 | 0.001386601 | 0.025321288 |
| PRDM12 | 0.415593125 | 0.048288626 | 0.25697403 |
| IL2RB | 0.419644068 | 0.031174007 | 0.201577687 |
| SSUH2 | 0.426315022 | 0.005319976 | 0.064756353 |
| ADHFE1 | 0.436163113 | 0.020146658 | 0.154272109 |
| REC8 | 0.438028104 | 0.001008848 | 0.019884957 |
| GADD45B | 0.444251097 | 2.48589E-33 | 6.26361E-30 |
| POU2F2 | 0.451421571 | 0.00184122 | 0.030593415 |
| NR1D1 | 0.477667614 | 2.31566E-13 | 6.25145E-11 |
| B3GALT4 | 0.493747256 | 0.000654517 | 0.014466349 |
| ATP1A2 | 0.4942084 | 0.002556125 | 0.038876752 |
| NGFR | 0.49725905 | 0.002087373 | 0.033528182 |
| TEX22 | 0.501874377 | 0.04467642 | 0.247405902 |
| SLC4A5 | 0.505648722 | 0.00188533 | 0.031141566 |
| LTB | 0.510758448 | 0.013007058 | 0.11649331 |
| SBK2 | 0.526778899 | 0.009398804 | 0.094162437 |
| DEPP1 | 0.528878361 | 5.20407E-07 | 3.42066E-05 |
| ATAD3C | 0.535290113 | 5.82852E-05 | 0.002052773 |
| THOC6 | 0.538341204 | 7.77351E-27 | 1.06836E-23 |
| IER5L | 0.549539826 | 2.02139E-25 | 2.18282E-22 |
| PTPRN2 | 0.550513022 | 1.0355E-05 | 0.000480204 |
| CSKMT | 0.552573669 | 0.017959302 | 0.14357944 |
| MYL5 | 0.555690528 | 0.007480302 | 0.080090088 |
| ADORA2A | 0.56768284 | 3.00631E-12 | 6.40133E-10 |
| MUC12 | 0.568759926 | 0.027241473 | 0.184514602 |
| ATF3 | 0.56938664 | 1.40747E-31 | 3.03973E-28 |
| PKD1L1 | 0.57580696 | 2.77184E-07 | 1.97664E-05 |
| OTOF | 0.577313144 | 0.031744274 | 0.203287844 |
| GGTLC2 | 0.581554276 | 0.024966114 | 0.175226421 |
| C12orf57 | 0.581904589 | 1.81277E-22 | 1.52252E-19 |
| FCGBP | 0.585970295 | 0.025782078 | 0.17808241 |
| EDN2 | 0.589193185 | 0.006758945 | 0.074913289 |
| EFCAB6 | 0.592048856 | 0.026055166 | 0.179209279 |
| SNAI3 | 0.599867898 | 0.002056321 | 0.033106995 |
| TNXB | 0.600307991 | 0.00095028 | 0.018903072 |
| SLC34A3 | 0.6009918 | 0.031556953 | 0.202666955 |
| AHRR | 0.61679232 | 9.92645E-10 | 1.44562E-07 |
| CREB5 | 0.618292075 | 2.30672E-05 | 0.000939975 |
| SPTBN5 | 0.62703919 | 0.014147776 | 0.123348372 |
| COX6B2 | 0.631737571 | 0.007803856 | 0.082618129 |
| PFKFB4 | 0.633849849 | 1.31604E-05 | 0.000578367 |
| NUTM2E | 0.633953686 | 0.001275828 | 0.023960209 |
| GADD45A | 0.639676553 | 9.394E-06 | 0.000441051 |
| SPACA6 | 0.643742719 | 0.007543166 | 0.080706006 |
| ASIC3 | 0.646341693 | 0.000546231 | 0.012558326 |
| SYNGAP1 | 0.650485709 | 2.5684E-06 | 0.000145974 |
| GNB1L | 0.651081879 | 4.09755E-08 | 3.80041E-06 |
| NPIPB12 | 0.653741243 | 0.033891079 | 0.210169911 |
| ZDHHC11 | 0.655163733 | 7.01742E-13 | 1.65765E-10 |
| NOTCH2NLA | 0.662237323 | 0.002550624 | 0.038832155 |
| PDE2A | 0.664690231 | 0.00091898 | 0.018450394 |
| KIRREL2 | 3.265998897 | 0.012273787 | 0.111444515 |
